# Spaceflight Changes the Production and Bioactivity of Secondary Metabolites in *Beauveria bassiana*

**DOI:** 10.3390/toxins14080555

**Published:** 2022-08-15

**Authors:** Youdan Zhang, Xiaochen Zhang, Jieming Zhang, Shaukat Ali, Jianhui Wu

**Affiliations:** 1 Key Laboratory of Bio-Pesticide Innovation and Application, Guangzhou 510642, China; 2 Engineering Research Center of Biological Control, Ministry of Education and Guangdong Province, South China Agricultural University, Guangzhou 510642, China

**Keywords:** space exposure, entomopathogenic fungi, pathogenicity, secondary metabolites

## Abstract

Studies on microorganism response spaceflight date back to 1960. However, nothing conclusive is known concerning the effects of spaceflight on virulence and environmental tolerance of entomopathogenic fungi; thus, this area of research remains open to further exploration. In this study, the entomopathogenic fungus *Beauveria bassiana* (strain SB010) was exposed to spaceflight (ChangZheng 5 space shuttle during 5 May 2020 to 8 May 2020) as a part of the Key Research and Development Program of Guangdong Province, China, in collaboration with the China Space Program. The study revealed significant differences between the secondary metabolite profiles of the wild isolate (SB010) and the spaceflight-exposed isolate (BHT021, BH030, BHT098) of *B. bassiana*. Some of the secondary metabolites/toxins, including enniatin A2, brevianamide F, macrosporin, aphidicolin, and diacetoxyscirpenol, were only produced by the spaceflight-exposed isolate (BHT021, BHT030). The study revealed increased insecticidal activities for of crude protein extracts of *B. bassiana* spaceflight mutants (BHT021 and BH030, respectively) against *Megalurothrips usitatus* 5 days post application when compared crude protein extracts of the wild isolate (SB010). The data obtained support the idea of using space mutation as a tool for development/screening of fungal strains producing higher quantities of secondary metabolites, ultimately leading to increased toxicity/virulence against the target insect host.

## 1. Introduction

An increase in space exploration has resulted in an increase in studies on understanding the changes in physiology of living organisms under spaceflight conditions [1]. Spaceflight conditions include long-term exposure to microgravity, radiation, and isolation [2]. The higher costs, limited number of launches, and complexity of experimental design under space habitats are the main difficulties in performing studies under true spaceflight conditions, and consequently, many studies have also been performed under ground-based simulated microgravity [3]. Different investigations have explored the effects of space conditions on plants, animals, as well as microorganisms. However, microorganisms can be considered strong candidates to study the responses to variations in environmental conditions because of their rapid life cycle, easy handling, and stability [4,5]. The explorations regarding influences of spaceflight on growth and metabolism of microorganisms have few significant implications. Firstly, microorganisms can impact (positively or negatively) human, animal and plant life [6]. Secondly, they are known for producing secondary metabolites, which are used as medicine, biopesticides, plant growth-promoting agents, etc. [7,8]. Several studies have explained the relationship between changed environmental conditions during spaceflight and variations in morphological as well as physiological characteristics of microorganisms, including germination, virulence, host pathogen relationship, secondary metabolite production, and gene expression [2,5,9].

Metabolism is the sum of all reactions in a living cell required for maintenance, development, and division. Microbial metabolism is comprised of primary metabolites (the intracellular molecules that enable growth and proliferation) and secondary metabolites (predominantly extracellular molecules that facilitate a microbe’s interaction and adaptation with its environment) [10,11]. Secondary metabolites produced by microorganisms are predominantly low-molecular-weight extracellular compounds [12]. They are usually produced in late-exponential and stationary phases and are not directly associated with growth, development, or division of microorganisms [10]. These specialized products are most notable for their use in healthcare settings as antimicrobial, antiparasitic, and pest-control agents [13,14,15,16,17]. Many of the intermediates in primary metabolism are precursors of secondary metabolites, and cells have evolved complex molecular switches linking primary and secondary metabolic pathways. These include high expression of the secondary metabolism genes at specific times in the cell cycle and controlling the flow of primary metabolites (carbon and nitrogen) through different pathways by feedback regulation [12,13,18]. Thus far, studies on secondary metabolism have focused on only a few microorganisms (mainly *Streptomycetes*, *Escherichia coli*, and *Bacillus*) and are mostly limited to one or a few metabolites per study. These studies have suggested altered secondary metabolite production levels, but the specific responses have been unique to each species. Furthermore, previous studies have been either limited to already-known metabolites or have focused on microorganisms that are already well-known metabolite producers. However, space vehicles hold diverse species whose behaviors are unstudied and could have responses under microgravity beyond prediction. Additionally, understanding microbes at a global metabolomics level could provide more comprehensive knowledge about the overall responses exhibited under microgravity.

Filamentous fungi are a major group of microorganisms critical to the production of different commercial enzymes, biopesticides, and organic compounds [19,20]. Several species of filamentous fungi belonging to the phylum Ascomycota (Subkingdom Dikarya) are known for their pathogenicity against insects [21]. Fungi belonging to genus *Beauveria* (Hypocreales: Cordycipitaceae) are one of the most common insect pathogenic fungal species. *Beauveria* species are known to cause widespread epizootics of insect populations because of their saprophytic behavior [22]. Studies regarding responses of microorganisms to spaceflight date back to 1960, but the responses to microgravity and its analogs have been investigated in a few microorganisms (bacteria as well as fungi), showing plausible but conflicting results for cellular growth rates and secondary metabolism under spaceflight and simulated microgravity experiments [23,24,25,26]. To date, limited concrete studies on the influences/utilization of spaceflight on production of secondary metabolites by entomopathogenic fungi make this area of research open for detailed investigations.

In this study, the entomopathogenic fungus *Beauveria bassiana* (SB010) was sent to the Taingong space station for exposure to spaceflight conditions on the ChangZheng 5 space shuttle during 5 May 2020 to 8 May 2020 as a part of the Key Research and Development Program of Guangdong Province, China, in collaboration with the China Space Program. The aims of this study were to (i) extract and characterize the changes in mycelial protein extract/secondary metabolites profiles of different *B. bassiana* spaceflight mutants and (ii) undertake toxicity assays of mycelial extract/secondary metabolites *B. bassiana* spaceflight mutants against *Megalurothrips usitatus* (Thysanoptera: Thripidae).

## 2. Results

**Figure 1:**
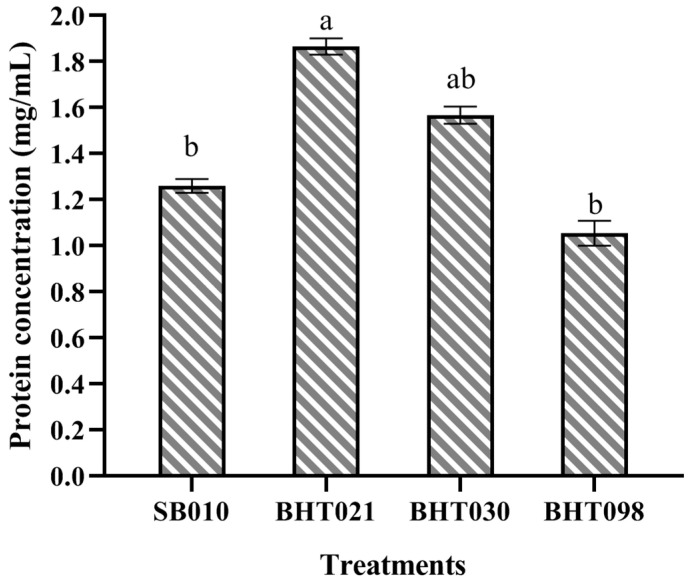
Total protein content of the crude protein extracts of the wild isolate (SB010) and spaceflight mutants (SCPH0535) of *Beauveria bassiana*. Error bars indicate standard error of means. Bars having different letters are significantly different from each other (Tukey’s test at 5% level of significance).

Secondary metabolite production was quantified from the ethyl acetate extracts of wild isolate (SB010) and spaceflight mutants (BHT021, BHT030, BHT098) of *B. bassiana*. The total protein concentration of ethyl acetate extract differed significantly among all four isolates (F_3,8_ = 41.32, *p* < 0.001). The highest protein contents were obtained from the spaceflight mutant BHT021, with a mean value of 1.87 ± 0.035 mg/mL. For the wild isolate (SB010), the concentration of protein observed was 1.26 ± 0.03 mg/mL. The lowest protein content was produced by the space mutant BHT098, with a mean value of 1.05 ± 0.05 mg/mL (Figure 1).

**Figure 2:**
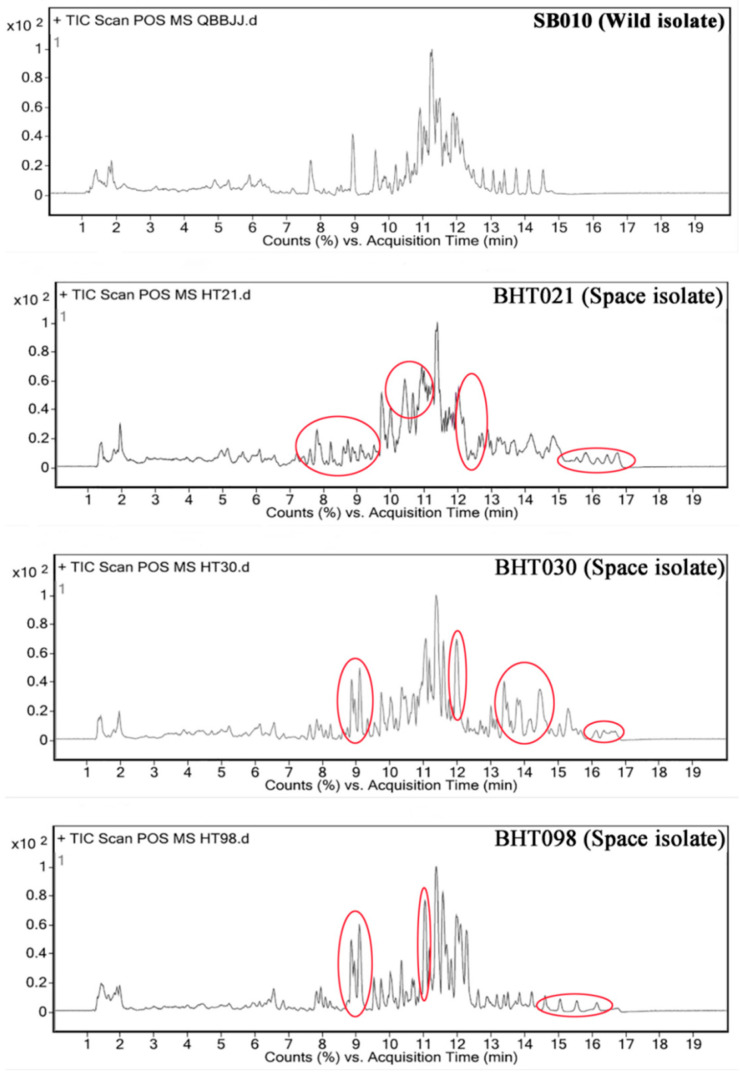
LC-MS analysis of the crude protein extracts from the wild isolate and spaceflight mutants of *Beauveria bassiana*. The indicated peaks (circle) show the variation in secondary metabolite profile of spaceflight mutants from the wild isolates.

As determined by LC-MS analysis, several differences were observed between the metabolite profiles of the four isolates (Figure 2; Supplementary file S1). A section of peaks between retention time of 8–9 min and 14.5–17 min was evident in the LC-MS profile of the spaceflight-exposed mutants (BHT021, BHT030, and BHT098) when compared with the wild isolate (SB010). In addition, a broader pattern of peaks between retention time of 10–11 min was observed for the spaceflight mutant BHT021 when compared with the wild isolate (SB010). Analysis of fragmentation pattern of peaks from the LC-MS profiles of the four isolates revealed the production of 43, 79, 44, and 47 secondary metabolites known for insecticidal activity by the SB010, BHT021, BHT030, and BHT098, respectively (Supplementary file S1). Some of the secondary metabolites/toxins, including enniatin A2, brevianamide F, macrosporin, aphidicolin, and diacetoxyscirpenol, were only produced by the spaceflight mutants BHT021 and HT030 (Supplementary file S1).

**Figure 3:**
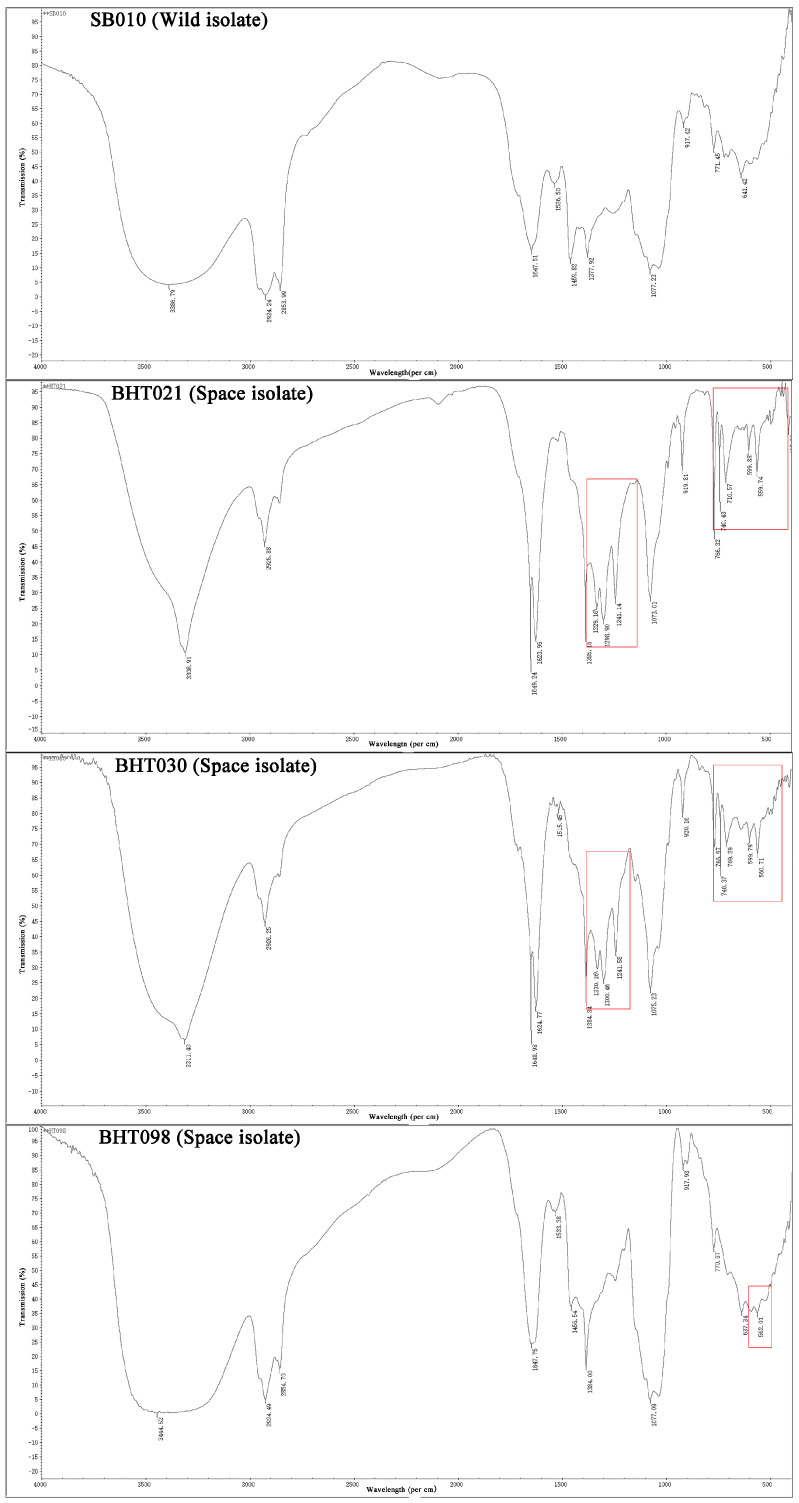
Fourier transformation infrared (FTIR) spectroscopy of the crude protein extracts of wild isolate (SB010) and spaceflight mutants (BHT021, BHT030, and BHT098) of *Beauveria bassiana*. The indicated peaks (circle) show the variation in secondary metabolite profile of spaceflight mutants from the wild isolates.

The FTIR analysis of ethyl acetate extracts of *B. bassiana* wild isolate (SB010) and spaceflight mutants (BHT021,BHT030, and BHT098) showed marked variations for functional groups of secondary metabolites (Figure 3). The FTIR profiles of the wild isolate (SB010) showed a broad peak at 3386.79 cm^−1^ (due to overlap of O-H or N-H stretching) along with sharp peaks at 2924.24 cm^−1^ (aliphatics (C-H), methylene functional group), 1647.51 cm^−1^ (carbonyl stretch amide linkage), and 641.42 cm^−1^ (aromatics).

In the case of *B. bassiana* spaceflight mutant (BHT021), the O-H or N-H stretching was observed at 3308.91 cm^−1^. The methylene (C-H) peak was observed at 2955.88 cm^−1^, whereas amide linkage peaks were observed at 1649.24 and 1623.96 cm^−1^, respectively. Furthermore, four sharp peaks of ester (C-O) functional group were observed at 1385.16, 1329.16, 1298.90, and 1241.14 cm^−1^, respectively. Five strong peaks representing the presence of aromatics were observed between 766.32–559.74 cm^−1^.

The FTIR profiles of *B. bassiana* spaceflight mutant (SB030) showed a broad peak at 3311.40 cm^−1^ (due to overlap of O-H or N-H stretching) along with peaks at 2924.24 cm^−1^ (aliphatics (C-H), methylene functional group), 1648.98 cm^−1^ (carbonyl stretch amide linkage). Furthermore, four sharp peaks of ester (C-O) functional group were observed at 1384.34, 1330.16, 1306.46 and 1241.58 cm^−1^, respectively. Five strong peaks representing the presence of aromatics were observed between 766.66–559.74 cm^−1^ (Figure 3).

In the case of *B. bassiana* spaceflight mutant (BHT098), the O-H or N-H stretching was observed at 34.44.52 cm^−1^. The methylene (C-H) peak was observed at 2924.49 cm^−1^, whereas amide linkage peaks were observed at 1647.75 cm^−1^, respectively. Furthermore, a single sharp peak of ester (C-O) functional group was observed at 138,400 cm^−1^. Two strong peaks representing the presence of aromatics were observed at 637.34 and 562.01 cm^−1^ (Figure 3).

The results showed the presence of additional ester (C-O) and aromatic functional groups in ethyl acetate extracts of the *B. bassiana* spaceflight mutants when compared to the FTIR profile of *B. bassiana* wild isolate (SB010).

### 2.1. Nuclear Magnetic Resonance (NMR)

**Figure 4:**
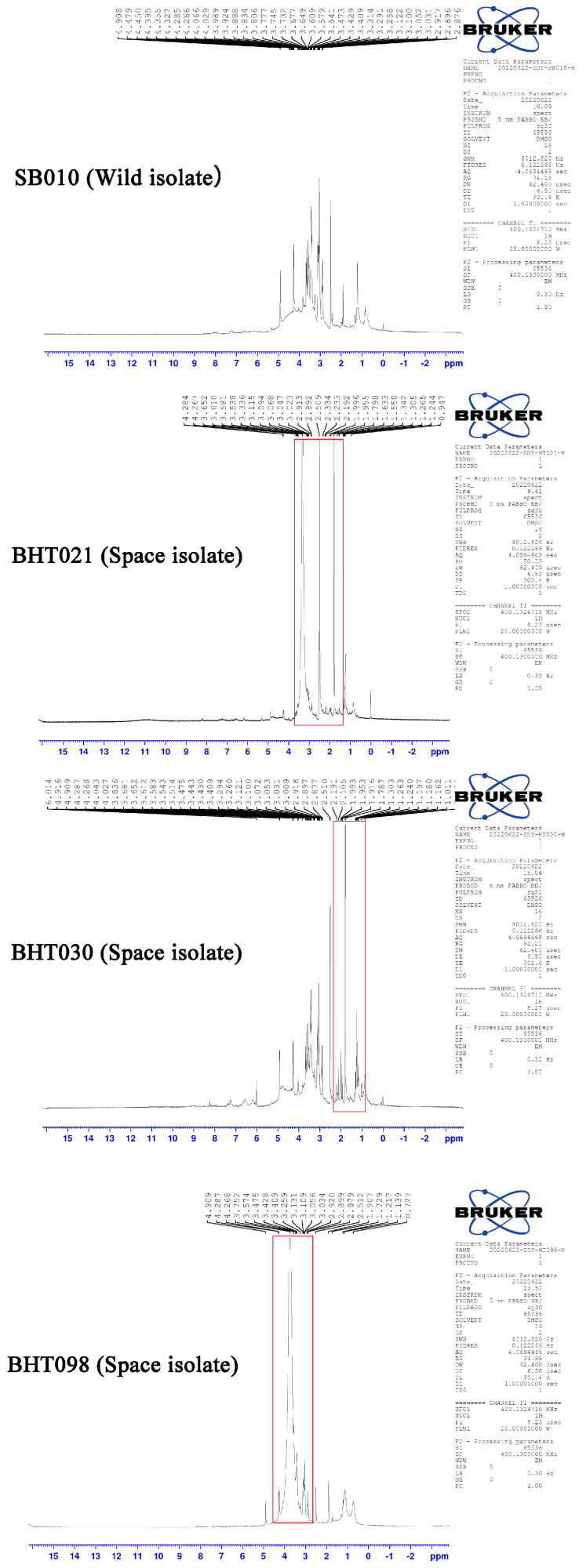
Nuclear magnetic resonance (NMR) resonance analysis of the crude protein extracts of wild isolate (SB010) and spaceflight mutants (BHT021, BHT030, and BHT098) of *Beauveria bassiana*. The indicated peaks (circle) show the variation in secondary metabolite profile of spaceflight mutants from the wild isolates.

The characterization of protein extracts of *B. bassiana* wild isolate SB010 through NMR analysis revealed bands of high resonance in allylic, pyrrolidine ring and NeCH (2.0–2.6 ppm and 2.7–3.5 ppm), and ether linkage (3.5–4.3 ppm) regions. The NMR spectrograms of metabolic compounds produced by *B. bassiana* space mutants (BHT021, BHT030, and BHT098) showed high-resonance bands in aliphatic (0.9–1.4 ppm), allylic, pyrrolidine ring (2.0–2.6 ppm), NeCH (2.1–3.5 ppm), and ether linkage (3.5–4.3 ppm) regions (Figure 4).

### 2.2. Toxicity of Metabolites against M. usitatus

The insecticidal activities of crude protein extracts from *B. bassiana* wild isolate (SB010) and space mutants (BHT021, BHT030, and BHT098) applied at different concentrations against *M. usitatus* adults differed significantly among different isolates and their concentrations (F_15,48_ = 23.81; *p* = 0.0034). The different concentrations of the crude protein extracts of *B. bassiana* spaceflight mutants (BHT021 and BHT030) had higher efficacy against *M. usitatus* adults 5 days post application compared to the wild isolate (SB010) (Table 1). The observed mortality (%) of *M. usitatus* adults following treatment with different concentration of the crude protein extracts of spaceflight mutants (BHT098) were lower than the mortality values observed for the crude protein extracts of wild isolate (SB010).

### 2.3. Transmission Electron Microscopic Examination of M. usitatus Midgut following Treatment with Mycelial Extracts from B. bassina Wild Isolate and Space Mutants

**Figure 5:**
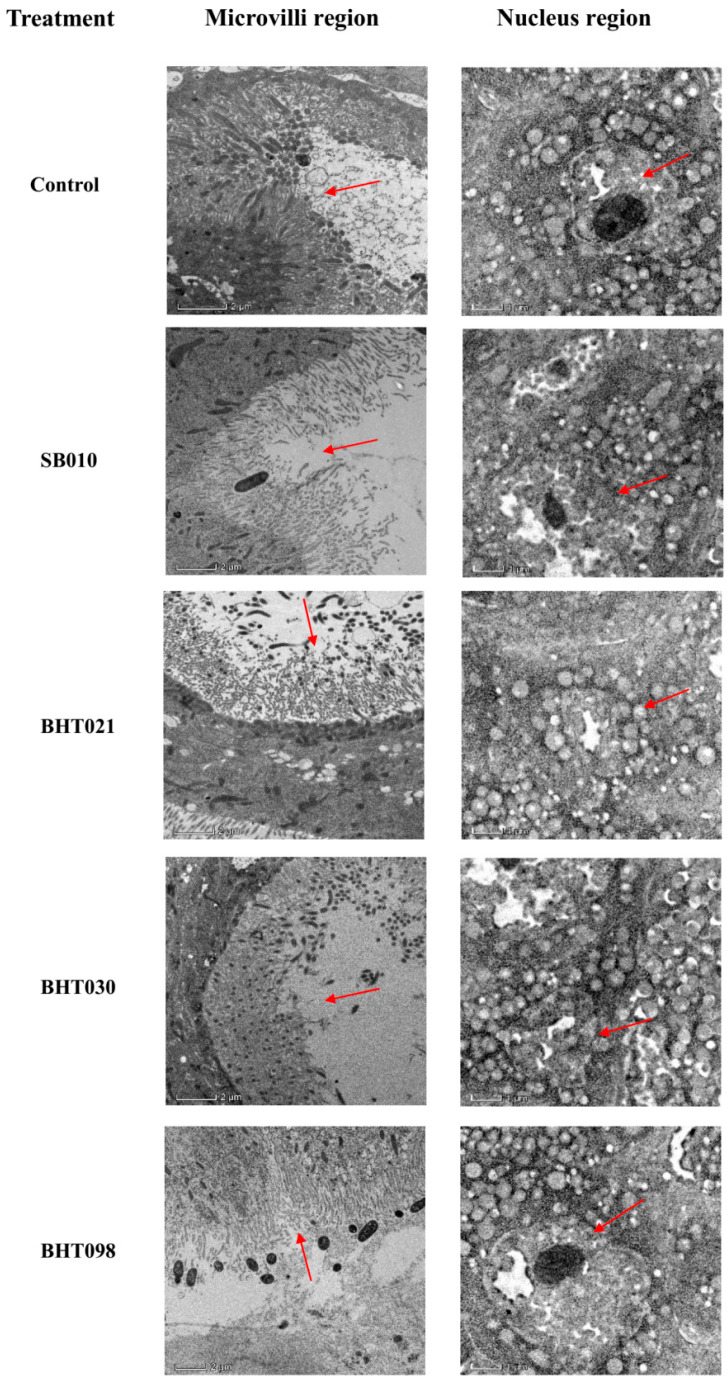
Transmission electron microscopic examination of *M. usitatus* following treatment with mycelial extracts from *Beauveria bassiana* wild isolate and space mutants. Arrow marks points with variations in *M. usitatus* midgut anatomy following different treatment.

The ultrastructural changes of the fat body and somatic cells of the treated group were observed by transmission electron microscopy after feeding the toxin for 72 h compared with the control. The microvilli of the midgut cells in the control were abundant and neatly arranged with a fenestrated morphology, and after 72 h of toxin feeding, the microvilli of the midgut of adult thrips were swollen, shortened and thinned, vacuolated, and partially dislodged (Figure 5). Compared with the control, the microvilli in the lumen of the midgut cells of *M. usitatus* treated with mycelial extracts of space mutants (BHT012 and BHT030) were sparse, disorganized, and vacuolated, while some of them were even completely lost. The nuclei of the blank group were flat, but after treatment with mycelial extracts of space mutants (BHT021 and BHT030), the fat body cells of *M. usitatus* adults were severely damaged, and their nuclei appeared expanded, folded, and detached, and the lipid droplet membrane structure became transparent and vacuolated (Figure 5).

## 3. Discussion

Studies investigating the influences of spaceflight on secondary metabolite production by microorganisms would be interesting, as the biosynthesis and concentration of microbial secondary metabolites are sensitive to extracellular environmental cues (nutrients availability, temperature, and osmotic stress) [27,28]. The results showed significant changes in total yield and secondary metabolite profiles of *B. bassiana* spaceflight mutants and the wild isolate. These findings are consistent with Lam et al. [29], who observed increased production of actinomycin D by *Streptomyces plicatus* WC56452 after spaceflight on the U.S. Space Shuttle mission STS-80. Similarly, Luo et al. [30] observed 13–18% higher production of nikkomycins by *Streptomyces ansochromogenus* during 15 days of spaceflight. In a different study, Nikkomycins-producing strains of *Streptomyces ansochromogenus* were investigated to understand the biological response to production onboard a satellite for 15 days. The production of Nikkomycins in nearly all strains was reported to be increased by 13–18%, with increases specifically in Nikkomycin X and Z [2,30]. Similarly, a study on *Cupriavidus metallidurans* under simulated microgravity showed increased production of the polyester polymer poly-b-hydroxybutyrate after 24 h but not after 48 h compared with ground controls [7]. All these results suggest that effects of microgravity on secondary metabolism may be specific depending on the strain, growth condition, pathway utilized, or time course analyzed.

Based on already-reported data and the results of this study as well as complexity of regulatory mechanisms of secondary metabolites production, the observed changes in secondary metabolite profiles of *B. bassiana* in response to spaceflight exposure can be strain, growth media, and secondary metabolism production pathway, depicting an inconsistent trend. These changes are induced by the indirect physical influences of spaceflight (such as variations in fluid dynamics or the extracellular transport of metabolites) [2]. Furthermore, different extracellular environmental cues can induce or enhance secondary metabolisms in different microorganisms, followed by the transfer of extracellular stresses to the downstream responsive genes by a cascade of complex signal transduction steps [29]. Therefore, although all of the *B. bassiana* space flight mutants went through same sort of extracellular stress, the amount and number of secondary metabolites produced by each mutant were different. In short, the changes in secondary metabolites production in *B. bassiana* following spaceflight exposure were induced by the variations in micro-environments around the fungal cells [2]. However, space vehicles hold diverse species whose behaviors are unstudied and could have responses under microgravity beyond prediction. Additionally, understanding microbes at a global metabolomics level could provide more comprehensive knowledge about the overall responses exhibited under microgravity since entomopathogenic fungi offer a wealth of potential for the discovery of new and important microbial products for pest control. Broadening the horizon of fungal species and understanding the altered levels under microgravity could offer unique advantages for the biopesticides industry. There remains much to discover about the nature of diverse secondary metabolisms in such stressful environments of spaceflight. The changes of environmental factors, such as temperature, oxygen availability, and diffusion limitations, under microgravity can provide a condition that can be harnessed in the best way possible to be used for engineered microorganisms to generate useful metabolites. Therefore, understanding the specific cause-and-effect mechanisms of fungal responses to microgravity at the molecular level could provide ground-breaking discoveries for space applications and biopesticides development. However, in order to highlight the microbial responses to microgravity for long periods of time, different technologies (such as low-shear-modeled microgravity (LSMMG)) have been developed to mimic real microgravity [31], and these technologies can be used more often (to mimic microgravity and ground level) to induce higher production of secondary metabolites by insect pathogenic fungi.

The examination of the biological activities of fungal crude protein extracts from *B. bassiana* wild isolate SB010 and space mutants (BHT021, BHT030, and BHT098), applied at different concentrations against *M. usitatus* adults, showed significantly different rates of insect mortality. The crude protein extracts of *B. bassiana* space mutants BHT021 and BHT030 (used at 100 μg/mL) induced higher mortality of *M. usitatus* adults when compared with the mortality caused by *B. bassiana* wild isolate SB010. On the other hand, *M. usitatus* mortality observed in response to treatment with crude protein extracts of *B. bassiana* space mutant BHT098 was lower than mortality caused by *B. bassiana* wild isolate SB010. Our results are similar to the already-reported previous studies on changes to microbial pathogens (*Bacillus subtilis; Escherichia coli; Pseudomonas aeruginosa; Staphylococcus aureus*) induced by spaceflight [29]. The transmission electron microscopy of the midgut of fourth instar *M. usitatus* adults showed nuclei enlargement, folding of nuclear membrane, and detachment of microvilli from midgut cells. The secretion of secondary metabolites is important for pathogenic processes, and variations in secondary metabolite profiles of spaceflight mutants compared to the wild isolate may have led to variations in biological activities of crude protein extracts against *M. usitatus*. These data further explain that increased secondary metabolism can increase the virulence of microorganisms [2].

## 4. Conclusions

The results obtained from our studies provide substantial evidence that spaceflight exposure can alter the secondary metabolites production and biological activities of *B. bassiana*, which can serve as a baseline information for the studies on the effects of microgravity on insect pathogenic fungi. However, the specific reasons or mechanisms regulating the above-mentioned changes are unclear. Therefore, further studies on corresponding gene expression/regulation and characterization of micro-environments around the fungal cells should be emphasized in near future for identification as well as application of secondary metabolites produced by *B. bassiana*.

## 5. Materials and Methods

### 5.1. Insect Cultures

*M. usitatus* adults were reared on cowpea pods following the method of Espinosa et al. [32] and Du et al. [33] for multiple generations in an artificial climate chamber (Model PYX-400Q-A, Shaoguan City Keli Instrument Co., Ltd., Ningbo, China). Freshly emerged *M. usitatus* adult females were used in subsequent studies.

### 5.2. Fungal Preparations

*Beauveria bassiana* isolate SB010 (deposited at Guangdong Microbiological Research Centre repository under accession number GDMCC NO. 60359) was used for the study. The fugal isolate was cultured on potato dextrose agar (PDA) plates for 15 d followed by preparation of a basal conidial concentration (1 × 10^8^ conidia/mL) using the method of Ali et al. [34].

### 5.3. Exposure of Beauveria bassiana to Spaceflight Conditions

One milliliter of *B. bassiana* conidial suspension (1 × 10^6^ conidia/mL) grown on PDA broth was individually loaded into polypropylene (PE) centrifuge tubes, which were then sealed with parafilm M (Bemis, Neenah, WI, USA). Four PE centrifuge tubes were exposed to simulated microgravity in a 3D rotating experimental device (temperature: 20 °C; speed: 9 rpm/min) for 72 h at Shenzhou Space Biology Science and Technology Corporation, Ltd., Beijing, China.

Polypropylene centrifuge tubes (4 tubes) having *B. bassiana* conidial suspension were exposed to spaceflight conditions by the following method. The PE tube samples were pooled and placed into experimental boxes. The experimental boxes were placed in the ChangZheng 5 space shuttle. The samples were then flown to space within the shuttle during 5 May 2020 to 8 May 2020. The samples stayed in higher earth orbit (altitude 3000–8000 km) for 67 h and faced the Van Allen radiation belt (high-energy particle radiation belt) several times during the spaceflight. The sample box was retrieved from the returning space capsule and opened after 10 days, with the PE tubes being stored at −20 °C until further use.

Aliquots (200 µL) of *B. bassiana* conidial suspension exposed to spaceflight conditions were cultured on PDA plates (15 plates each) following the method of Zhao et al. [35]. The fastest-growing colonies from spaceflight-exposed *B. bassiana* conidial suspensions were selected and named as BHT021, BHT030, and BHT098.The fugal isolate was cultured on PDA plates for 15 d followed by preparation of a basal conidial concentration (1 × 10^8^ conidia/mL) using the method of Ali et al. [34].

### 5.4. Production and Characterization of Crude Protein Extracts of Wild Isolate (SB010) and Spaceflight Mutants (BHT021, BHT030, and BHT098) of Beauveria bassiana

Sterilized growth medium, 100 mL, (containing/L: glucose 30 g, yeast extract 3 g, KH_2_PO 0.39 g, Na_2_HPO_4_·12H_2_O 1.42 g, NH_4_NO_3_ 0.70 g, and KCl 1.00 g), added to Erlenmeyer flasks (250 mL), was inoculated with 5 mL of conidial suspension (1 × 10^8^ conidia/mL) of wild isolate (SB010) and spaceflight mutants (BHT021, BHT030, and BHT098) of *B. bassiana* followed by incubation at 150 rpm and 27 °C for 5 days. After 5 days of growth, cultures were centrifuged in an Eppendorf 5804R centrifuge (Eppendorf, Framingham, MA, USA) at 10,000 rpm, 4 °C for 10 min, and the resultant supernatant was extracted with ethyl acetate (1: 1 *v*/*v* ratio) following Wu et al. [36]. Three individual samples were run for each treatment as biological replicate.

The total protein concentration of the extracts was quantified by Bradfords’ method using bovine albumin serum as standard [37].

The liquid chromatography–mass spectrophotometry (LC-MS) analysis of obtained protein extracts was carried out by the method of Wu et al. [36] using LC Agilent 1200 LC-MS/MS system. The detailed description of LC-MS protocol can be seen in the supplementary materials (Supplementary file 2).

Fourier-transformed infrared spectroscopy analysis was performed by using MIR8035 FTIR spectrometer (Thermo Fisher Scientific, Germany). All measurements were made at a resolution of 4 cm^−1^ over a frequency range of 400 to 4000 cm^−1^. The liquid sample was loaded directly, and the spectra were recorded at room temperature.

Nuclear magnetic resonance (NMR) was performed using a Bruker advance III-HD 600 NMR spectrometer (Bruker, Karlsruhe, Germany) by following the method of Wu et al. [36].

### 5.5. Toxicity of Crude Protein Extracts of Wild Isolate (SB010, and Spaceflight Mutants (BHT021, BHT030, and BHT098) of Beauveria bassiana against Megalurothrips usitatus

The concentration mortality response of crude protein extracts of wild isolate (SB010) and spaceflight mutants (BHT021, BHT030, and BHT098) of *B. bassiana* against *M. usitatus* adult females was studied by following the method Du et al. [26]. Briefly, centrifuge tubes (9 mL) and bean pods (1 cm) were individually immersed in different crude protein extracts of different concentrations (100, 75, 50, 25, and 12.5 µg/mL) followed by drying under sterile conditions. Centrifuge tubes and bean pods immersed in ddH_2_O with 0.05% Tween-80 served as control. Adult females of *M. usitatus* (100 individuals) were inoculated to treated bean pods with camel hair brush, and bean pods were placed in a treated centrifuge tube. Each centrifuge tube was sealed with a cotton plug to prevent the thrips from escaping and was placed at 26 ± 1 °C, 70 ± 5% R.H., and 16:8 L:D photoperiod. The insects were observed on a daily basis for 5 days to record mortality data as outlined by Du et al. [33]. The treatments were repeated three times with fresh batches of insects.

Changes in the appearance of the infected *M. usitatus* midgut were directly monitored at 5 days post treatment under a JEM1011 transmission electron microscope (Nikon Co. Ltd., Tokyo, Japan) following Du et al. [26]. The treated *M. usitatus* adults were sampled at 5 d post treatment and were dissected under stereo microscope (Stemi 508, Carl-ZEISS, Jena, Germany) to obtain midgut samples. The samples were fixed overnight in 2.5% glutaraldehyde + 2% paraformaldehyde solution at 4 °C followed by rinsing with PBS buffer (0.1 M). The samples were then stained overnight with 1% uranyl acetate at 4 °C followed by dehydration with gradient concentrations of ethanol. The dehydrated tissues were embedded in silica gel blocks, and sections were cut using automatic microwave tissue processing instrument (EM AMW, Leica Microsystems, Wetzlar, Germany) and cryo-ultramicrotome (EM UC7/FC7, Leica Microsystems, Wetzlar, Germany).

### 5.6. Data Analysis

Data regarding total protein concentration were subjected to ANOVA-1. Means were compared by Tukey’s HSD test (*p* < 0.05). Mortality (%) data were arcsine transformed before further analysis. The mortality (%) data were subjected to ANOVA-2, and significance between means was also tested by Tukey’s HSD test (*p* < 0.05). SAS 9.2 was used for all statistical analysis [38].

## Figures and Tables

**Table 1:** Dose–mortality responses of *M. usitatus* adults to crude protein extracts of wild isolate (SB010) and spaceflight mutants (BHT021, BHT030, and BHT098) of *Beauveria bassiana*.

Treatments	Concentration (μg/mL)	Mortality (%)
**SB010**	ddH_2_O	5.00 j
	100	88.33 ab
	75	80.00 c
	50	73.33 d
	25	65.00 ef
	12.5	50.00 h
**BHT021**	ddH_2_O	5.00 j
	100	100.00 a
	75	91.67 ab
	50	83.33 bc
	25	70.00 e
	12.5	63.33 f
**BHT030**	ddH_2_O	5.00
	100	95.00 a
	75	83.33
	50	75.00 d
	25	63.33 f
	12.5	56.67 g
**BHT098**	ddH_2_O	5.00 j
	100	81.67 c
	75	73.33 d
	50	61.67 f
	25	55.00 g
	12.5	46.67 i

The difference between the means (±SE) followed by various letters is significant (Tukey’s *p* < 0.05).

## Data Availability

The raw data supporting the conclusion will be made available by the corresponding author on request.

## Supplementary Materials

The following supporting information can be downloaded at: https://www.mdpi.com/article/10.3390/toxins14080555/s1, Supplementary file S1: Detailed LCMS analysis of the crude protein extracts from wild isolate and spaceflight mutants of *Beauveria bassiana*. Supplementary file S2: Supplementary methods.
