# Supplementary material for: Spaceflight Changes the Production and Bioactivity of Secondary Metabolites in Beauveria bassiana
Source: Toxins (Basel). 2022 Aug 15;14(8):555. doi: 10.3390/toxins14080555 (PMC9416017; doi:10.3390/toxins14080555)
Supplement: Supplementary file 1 [file toxins-14-00555-s001.zip › Supplementary file S2.pdf]

Liquid chromatography-mass spectrophotometry (LC-MS) analysis

The LC-MS analysis was performed by using LC-MS/MS system, consisting of a LC Agilent 1200 using a binary pump and an automatic injector, and coupled to a 3200 QTRAP® AB SCIEX equipped with a Turbo-V™ source (electrospray ionization) interface. The chromatographic separation of the compounds was conducted at 24 ± 1°C on a reverse-phase analytical column C_{18} (3 μm, 150 × 2 mm ID) and a guard-column C_{18} (4 x 2 mm, ID; 3 μm). Mobile phases were as follows: methanol (0.1% acetic acid and 5 mM ammonium acetate) as Phase A and water (0.1% acetic acid and 5 mM ammonium acetate) as Phase B. The following gradient was used: equilibration at 90% B for 2 min, from 80% to 20% B in 3 min, 20% B for 1 min, from 20% to 10% B in 2 min, 10% B for 6 min, from 10% to 0% B in 3 min, 100% A for 1 min, from 100% to 50% A in 3 min, return to initial conditions in 2 min, and maintain at initial conditions for 2 min. The flow rate was 0.25 mL min^{-1} in all steps. Total run time was 21 min. The injection volume was 20 μL. In regards to mycotoxin analysis, the QTRAP System was used as selected reaction monitoring (SRM). The Turbo-V™ source was used in positive mode with the following settings for source/gas parameters: vacuum gauge (10e-5 Torr) 3.1, curtain gas (CUR) 20, ion spray voltage (IS) 5500, source temperature (TEM) 450°C, ion source gas 1 (GS1), and ion source gas 2 (GS2) 50. The entrance potential (EP) was the same for all analytes, i.e. 10 V. Acquisition and processing data were performed using Analyst® software, version 1.5.2 (AB SCIEX, Concord, ON, Canada).

Fourier transformed infrared spectroscopy (FTIR)

Fourier transformed infrared spectroscopy analysis was performed by using MIR8035 FTIR spectrometer (Thermo Fisher, Germany). All measurements were made at a resolution of 4 cm^{-1} over a frequency range of 400 to 4000 cm^{-1}. The liquid sample was loaded directly, and the spectra were recorded at room temperature.

Nuclear magnetic resonance (NMR)

Nuclear magnetic resonance (NMR) was performed using a Bruker advance III- HD 600 NMR spectrometer (Bruker, Karlsruhe, Germany) by following the method of Wang et al. [32].
