# Supplementary material for: Spaceflight Changes the Production and Bioactivity of Secondary Metabolites in Beauveria bassiana
Source: Toxins (Basel). 2022 Aug 15;14(8):555. doi: 10.3390/toxins14080555 (PMC9416017; doi:10.3390/toxins14080555)
Supplement: Supplementary file 1 [file toxins-14-00555-s001.zip › Supplemnetary file S1.pdf]

Qualitative Compound Report

**Data File**	POS MS QBBJJ.d	**Sample Name**	QBBJJ
**Sample Type**	Sample	**Position**	P1-C1
**Instrument Name**	Instrument 1	**User Name**	
**Acq Method**	Mycotoxins POS.m	**Acquired Time**	4/26/2022 10:01:41 AM
**IRM Calibration Status**	Success	**DA Method**	default.m
**Comment**			

**Sample Group**		**Info.**	
**Stream Name**	LC 1	**Acquisition SW Version**	6200 series TOF/6500 series Q-TOF B.06.01 (B6157)

Fragmentor Voltage 135 **Collision Energy** 0 **Ionization Mode** ESI

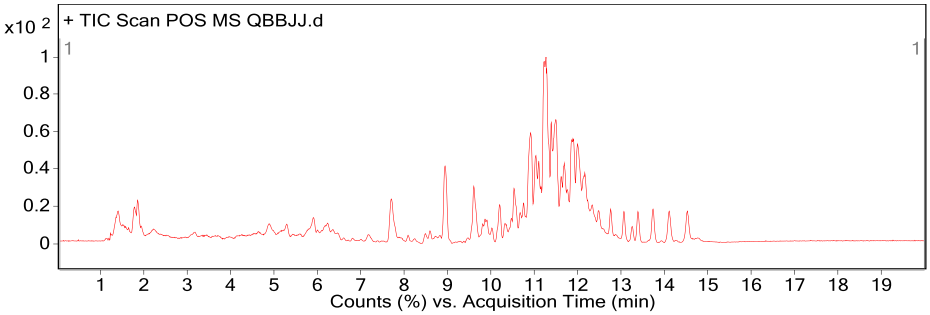

Fragmentor Voltage **Collision Energy** **Ionization Mode**

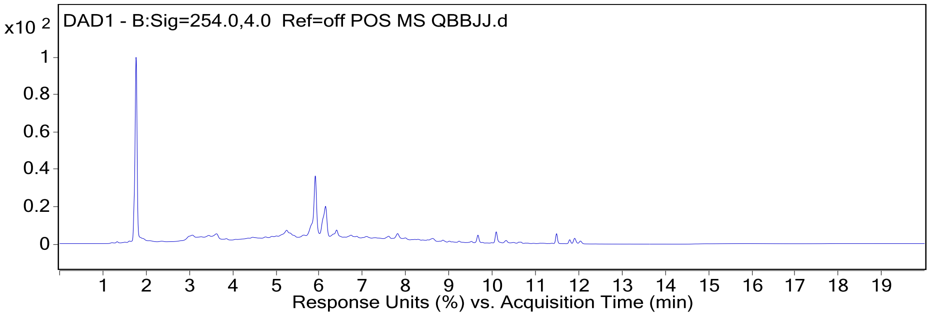

Fragmentor Voltage 135 **Collision Energy** 0 **Ionization Mode** ESI

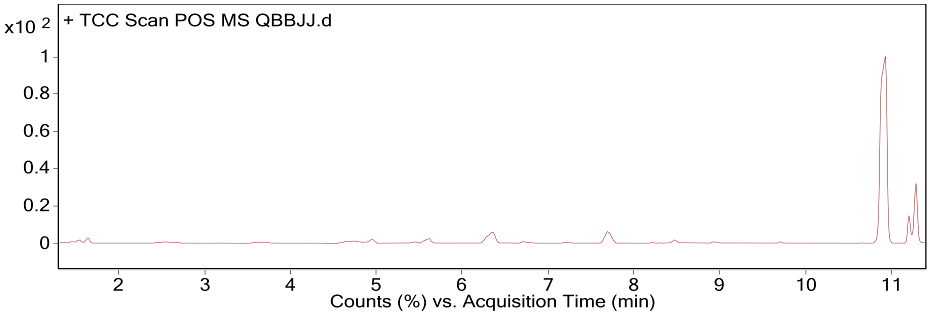

Compound Table

Compound Label	RT	Mass	Name	Formula	Tgt Mass	Diff (ppm)	SignificantIonMz
Cpd 1: Fusarinolic acid	1.35	195.0893	Fusarinolic acid	C10 H13 N O3	195.0895	-1.31	213.1231
Cpd 2: Chlamydospordiol	1.44	228.0995	Chlamydospordiol	C11 H16 O5	228.0998	-1.26	246.1333
Cpd 3: Fusarinolic acid	1.45	195.0892	Fusarinolic acid	C10 H13 N O3	195.0895	-1.57	213.1231

Cpd 4: Cerulenin	1.53	223.1206	Cerulenin	C12 H17 N O3	223.1208	-0.97	241.1544
Cpd 5: Fusarinolic acid	1.63	195.0892	Fusarinolic acid	C10 H13 N O3	195.0895	-1.83	213.123
Cpd 6: Cerulenin	1.64	223.1207	Cerulenin	C12 H17 N O3	223.1208	-0.69	241.1545
Cpd 7: Cerulenin	1.79	223.1207	Cerulenin	C12 H17 N O3	223.1208	-0.42	241.1546
Cpd 8: Terrein	2.55	154.0628	Terrein	C8 H10 O3	154.063	-1.2	172.0966
Cpd 9: Pyrenocine A	2.7	208.073	Pyrenocine A	C11 H12 O4	208.0736	-2.48	226.1069
Cpd 10: 5-Methyl-mellein	3.13	192.0787	5-Methyl-mellein	C11 H12 O3	192.0786	0.34	210.1125
Cpd 11: 5-Methyl-mellein	3.58	192.0784	5-Methyl-mellein	C11 H12 O3	192.0786	-1.1	210.1123
Cpd 12: Terrein	3.68	154.0628	Terrein	C8 H10 O3	154.063	-1.1	172.0966
Cpd 13: Austdiol	4.3	236.0683	Austdiol	C12 H12 O5	236.0685	-0.79	254.1021
Cpd 14: Fusaric acid	4.63	179.0946	Fusaric acid	C10 H13 N O2	179.0946	-0.28	197.1284
Cpd 15: Fusaric acid	4.69	179.0947	Fusaric acid	C10 H13 N O2	179.0946	0.28	197.1285
Cpd 16: Fusaric acid	4.75	179.0946	Fusaric acid	C10 H13 N O2	179.0946	-0.3	197.1284
Cpd 17: Fusaric acid	4.95	179.0948	Fusaric acid	C10 H13 N O2	179.0946	0.68	197.1286
Cpd 18: Cerulenin	5.17	223.1209	Cerulenin	C12 H17 N O3	223.1208	0.21	241.1547
Cpd 19: Infectopyrone	5.45	264.0996	Infectopyrone	C14 H16 O5	264.0998	-0.51	282.1335
Cpd 20: 5-Methyl-mellein	5.46	192.0785	5-Methyl-mellein	C11 H12 O3	192.0786	-0.47	210.1124
Cpd 21: Fusaric acid	5.6	179.0948	Fusaric acid	C10 H13 N O2	179.0946	0.7	180.1021
Cpd 22: Enniatin K1	6.31	625.391	Enniatin K1	C32 H55 N3 O9	625.3938	-4.58	648.3803
Cpd 23: Brevianamid F	6.35	283.1324	Brevianamid F	C16 H17 N3 O2	283.1321	1.28	284.1397
Cpd 24: Pyrenocine A	6.56	208.0735	Pyrenocine A	C11 H12 O4	208.0736	-0.3	226.1073
Cpd 25: Enniatin K1	6.71	625.3911	Enniatin K1	C32 H55 N3 O9	625.3938	-4.39	648.3803
Cpd 26: Fusarinolic acid	6.78	195.0896	Fusarinolic acid	C10 H13 N O3	195.0895	0.37	196.0969
Cpd 27: DAS / Diacetoxyscirpenol	7.22	366.1678	DAS / Diacetoxyscirpenol	C19 H26 O7	366.1679	-0.27	384.2016
Cpd 28: Fusaric acid	7.7	179.0949	Fusaric acid	C10 H13 N O2	179.0946	1.42	180.1022
Cpd 29: beta-Zearalenol	7.76	320.1622	beta-Zearalenol	C18 H24 O5	320.1624	-0.6	338.196
Cpd 30: 15-Hydroxyculmorone	7.93	252.1725	15-Hydroxyculmorone	C15 H24 O3	252.1725	-0.04	270.2064
Cpd 31: Fusaric acid	8.22	179.0945	Fusaric acid	C10 H13 N O2	179.0946	-0.6	180.1018
Cpd 32: Culmorin	8.3	238.1929	Culmorin	C15 H26 O2	238.1933	-1.78	256.2267
Cpd 33: Palitantin	8.34	254.1526	Palitantin	C14 H22 O4	254.1518	3.29	272.1865
Cpd 34: FS-4	8.46	250.1568	FS-4	C15 H22 O3	250.1569	-0.27	268.1906
Cpd 35: Culmorin	8.49	238.1933	Culmorin	C15 H26 O2	238.1933	0.18	256.2271
Cpd 36: Deepoxy deoxynivalenol	8.94	280.1308	Deepoxy deoxynivalenol	C15 H20 O5	280.1311	-1.03	281.1381
Cpd 37: Paspaline	9.71	421.2957	Paspaline	C28 H39 N O2	421.2981	-5.76	439.3308
Cpd 38: Ionomycin	10.66	708.5209	Ionomycin	C41 H72 O9	708.5176	4.57	731.5101
Cpd 39: 2-Amino-14,16-dimethyloctadecan-3-ol	10.84	313.334	2-Amino-14,16-dimethyloctadecan-3-ol	C20 H43 N O	313.3345	-1.62	336.3232
Cpd 40: Beauvericin	10.91	783.4055	Beauvericin	C45 H57 N3 O9	783.4095	-5.02	801.4396
Cpd 41: Sirolimus (Rapamycin)	11.21	913.5589	Sirolimus (Rapamycin)	C51 H79 N O13	913.5551	4.13	931.5931
Cpd 42: Sirolimus (Rapamycin)	11.29	913.5585	Sirolimus (Rapamycin)	C51 H79 N O13	913.5551	3.68	931.5925
Cpd 43: Sirolimus (Rapamycin)	11.38	913.5561	Sirolimus (Rapamycin)	C51 H79 N O13	913.5551	1	931.5899

Compound Label	Name	*m/z*	RT	Algorithm	Mass
Cpd 1: Fusarinolic acid	**Fusarinolic acid**	213.1231	1.35	Find by Molecular Feature	195.0893

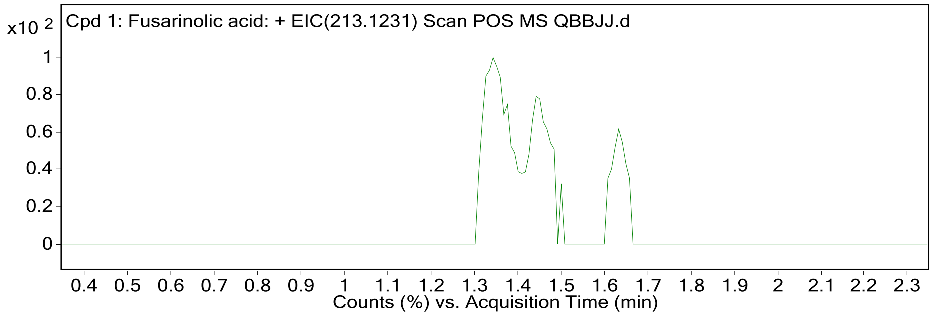

MFE MS Zoomed Spectrum

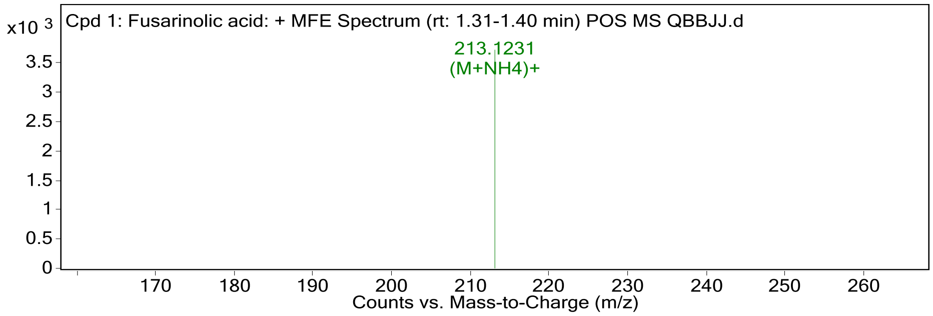

Compound Label	Name	*m/z*	RT	Algorithm	Mass
Cpd 2: Chlamydospordiol	**Chlamydospordiol**	246.1333	1.44	Find by Molecular Feature	228.0995

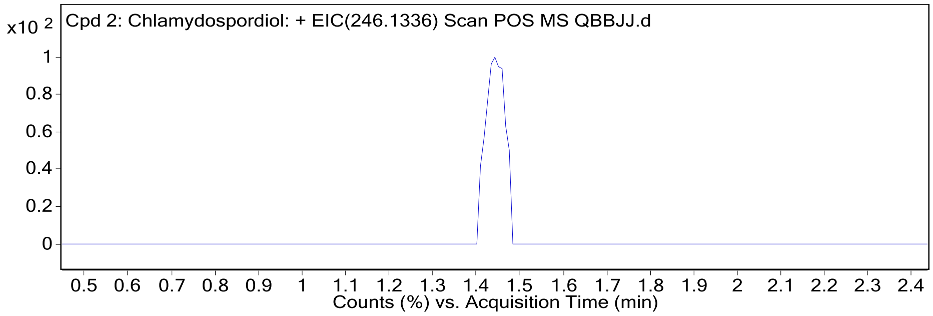

MFE MS Zoomed Spectrum

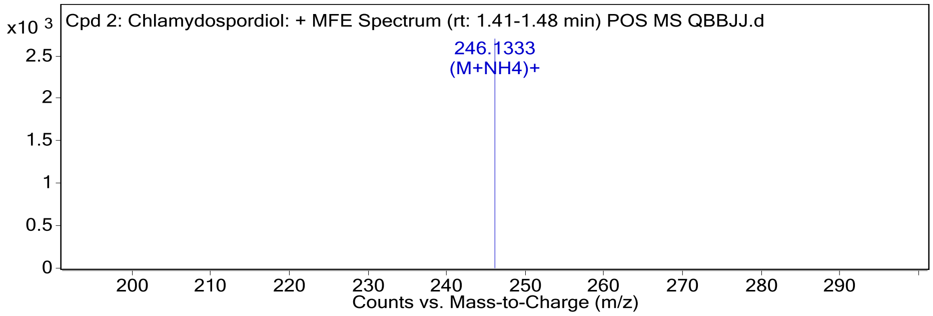

Compound Label	Name	*m/z*	RT	Algorithm	Mass
Cpd 3: Fusarinolic acid	**Fusarinolic acid**	213.1231	1.45	Find by Molecular Feature	195.0892

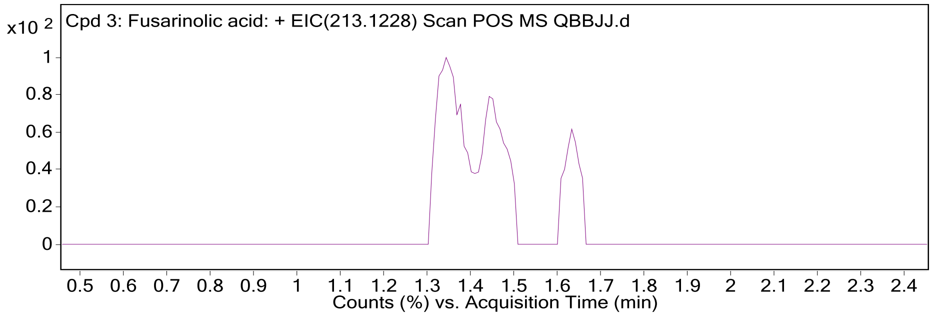

MFE MS Zoomed Spectrum

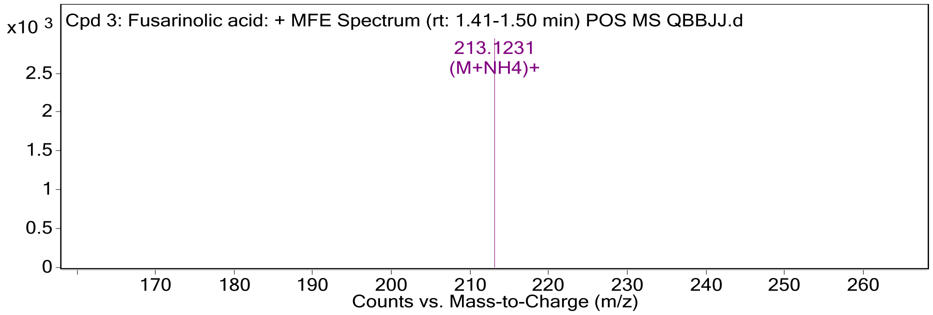

Compound Label	Name	*m/z*	RT	Algorithm	Mass
Cpd 4: Cerulenin	**Cerulenin**	241.1544	1.53	Find by Molecular Feature	223.1206

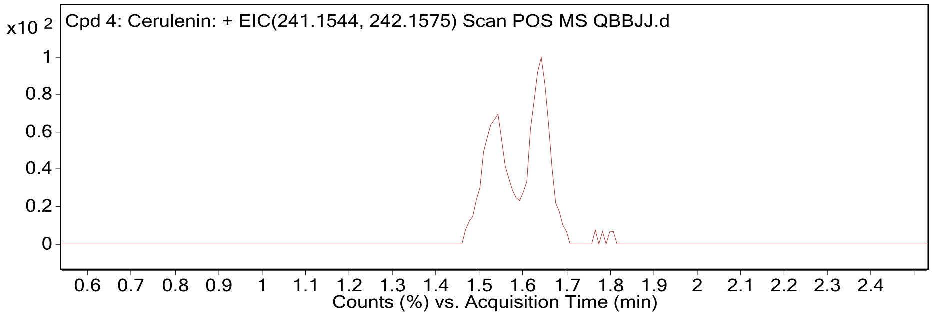

MFE MS Zoomed Spectrum

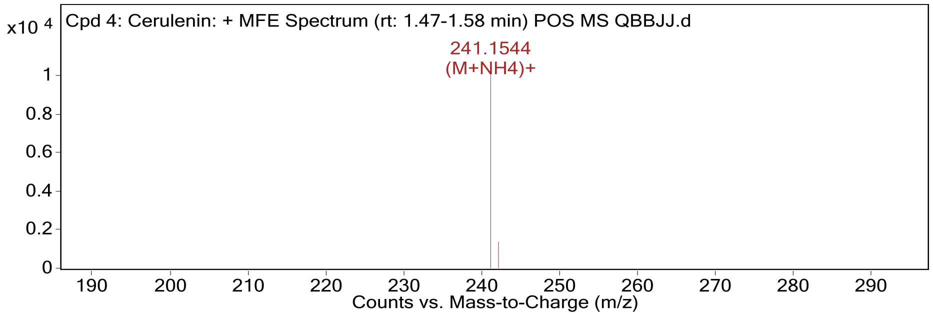

Compound Label	Name	*m/z*	RT	Algorithm	Mass

Cpd 5: Fusarinolic acid	**Fusarinolic acid**	213.123	1.63	Find by Molecular Feature	195.0892

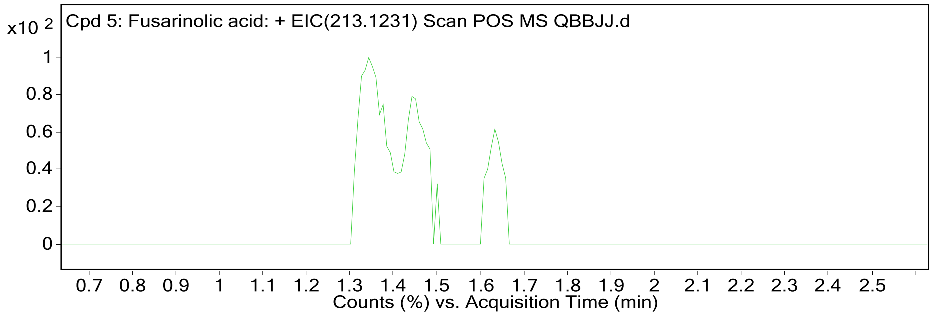

MFE MS Zoomed Spectrum

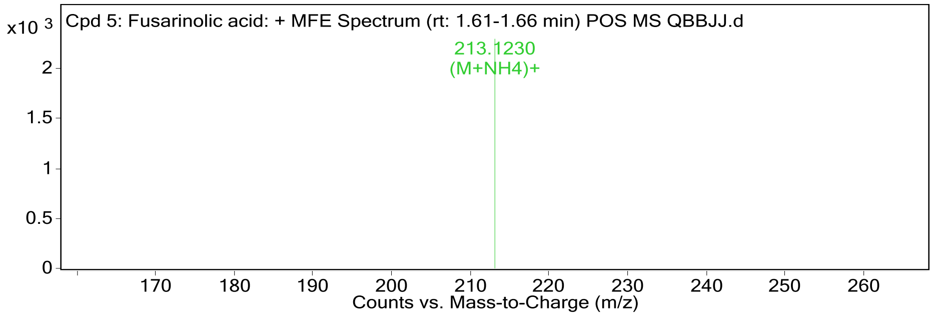

Compound Label	Name	*m/z*	RT	Algorithm	Mass
Cpd 6: Cerulenin	**Cerulenin**	241.1545	1.64	Find by Molecular Feature	223.1207

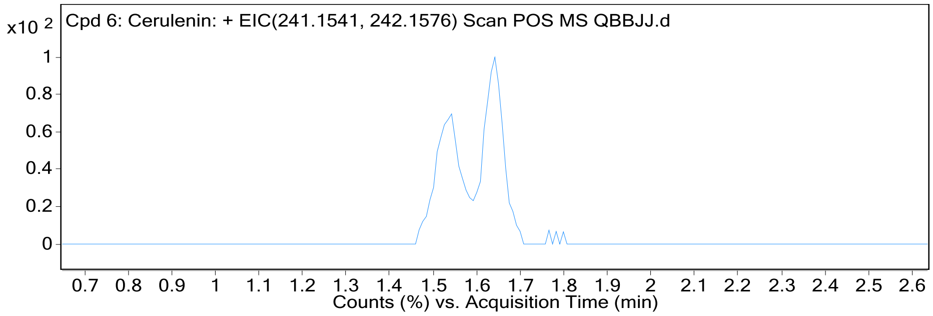

MFE MS Zoomed Spectrum

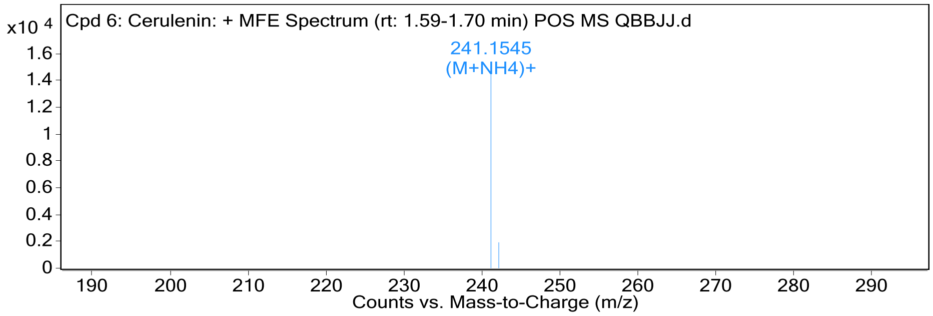

Qualitative Compound Report

Compound Label	Name	*m/z*	RT	Algorithm	Mass
Cpd 7: Cerulenin	**Cerulenin**	241.1546	1.79	Find by Molecular Feature	223.1207

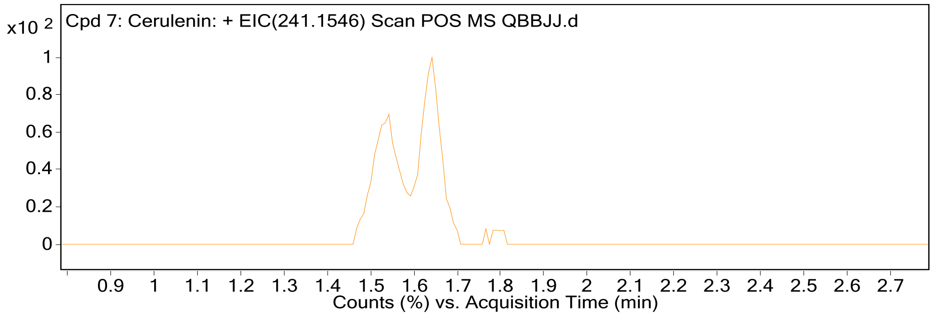

MFE MS Zoomed Spectrum

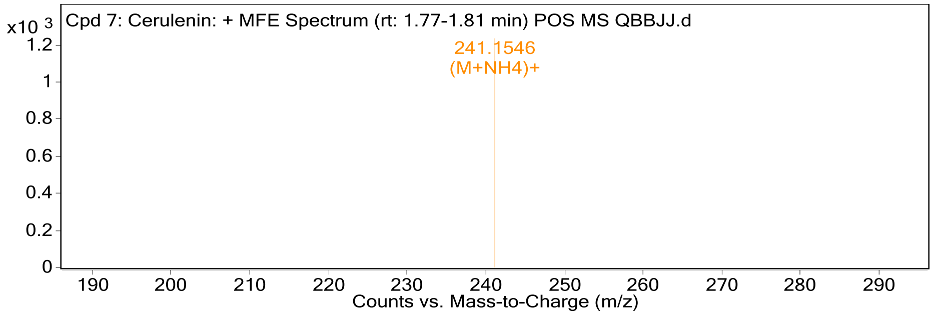

Compound Label	Name	*m/z*	RT	Algorithm	Mass
Cpd 8: Terrein	**Terrein**	172.0966	2.55	Find by Molecular Feature	154.0628

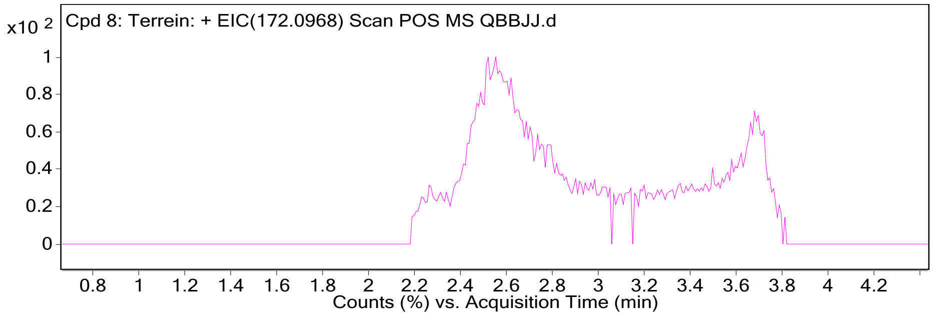

MFE MS Zoomed Spectrum

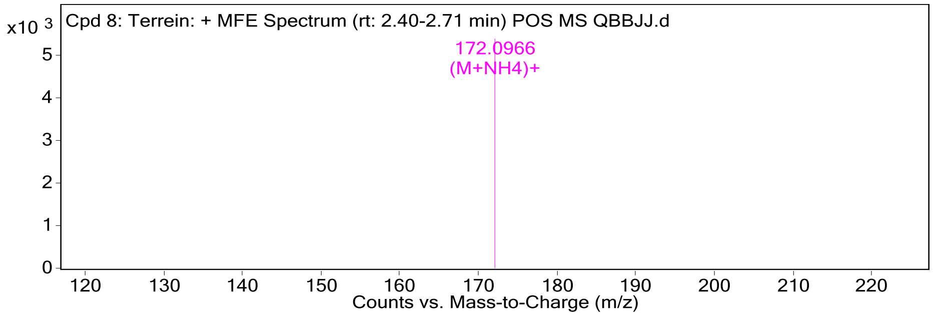

Compound Label	Name	*m/z*	RT	Algorithm	Mass
Cpd 9: Pyrenocine A	**Pyrenocine A**	226.1069	2.7	Find by Molecular Feature	208.073

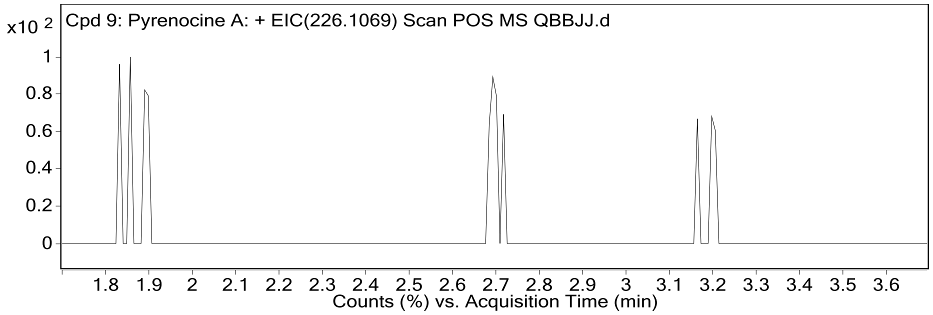

MFE MS Zoomed Spectrum

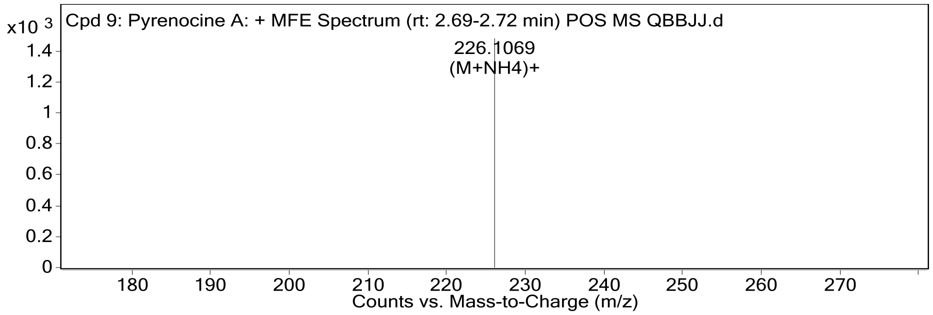

Compound Label	Name	*m/z*	RT	Algorithm	Mass
Cpd 10: 5-Methyl-mellein	**5-Methyl-mellein**	210.1125	3.13	Find by Molecular Feature	192.0787

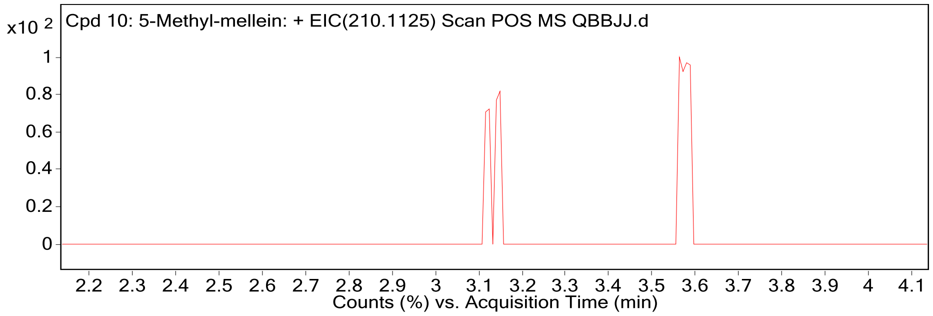

MFE MS Zoomed Spectrum

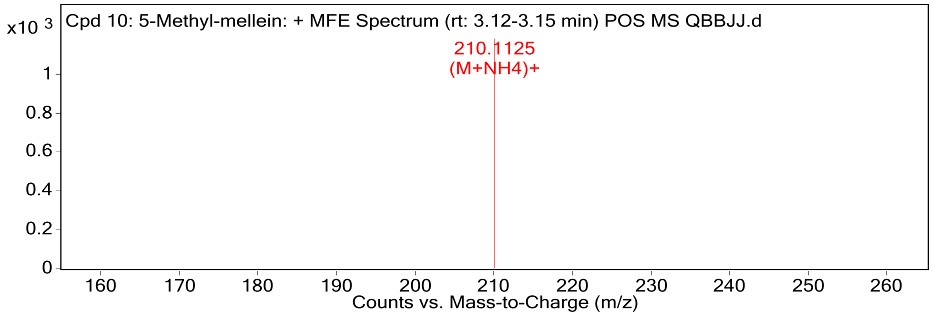

Qualitative Compound Report

Compound Label	Name	*m/z*	RT	Algorithm	Mass
Cpd 11: 5-Methyl-mellein	**5-Methyl-mellein**	210.1123	3.58	Find by Molecular Feature	192.0784

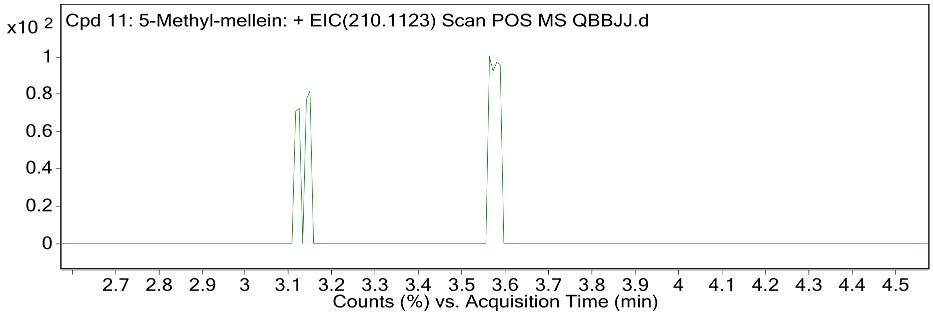

MFE MS Zoomed Spectrum

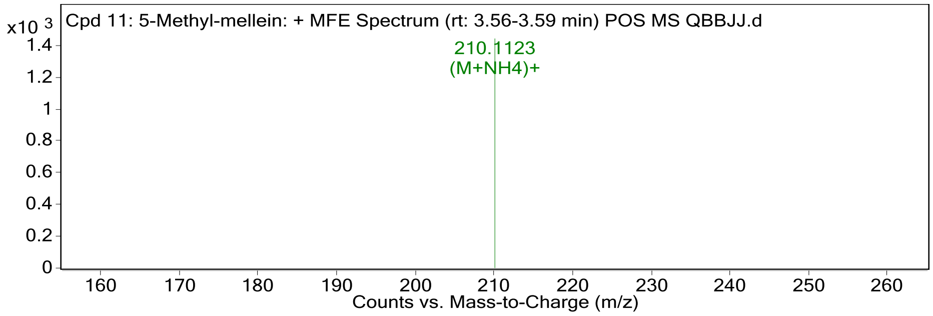

Compound Label	Name	*m/z*	RT	Algorithm	Mass
Cpd 12: Terrein	**Terrein**	172.0966	3.68	Find by Molecular Feature	154.0628

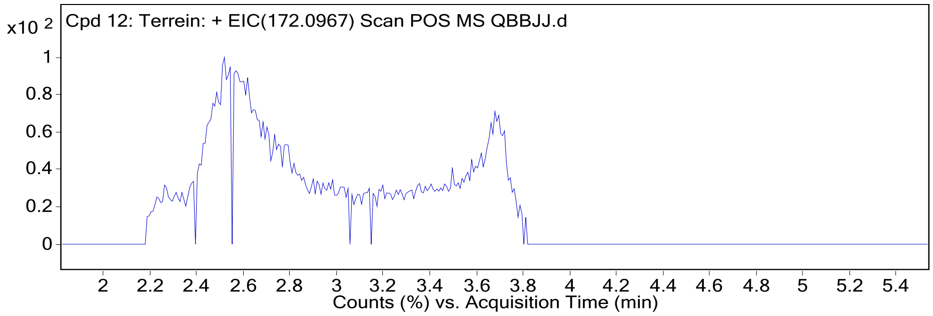

MFE MS Zoomed Spectrum

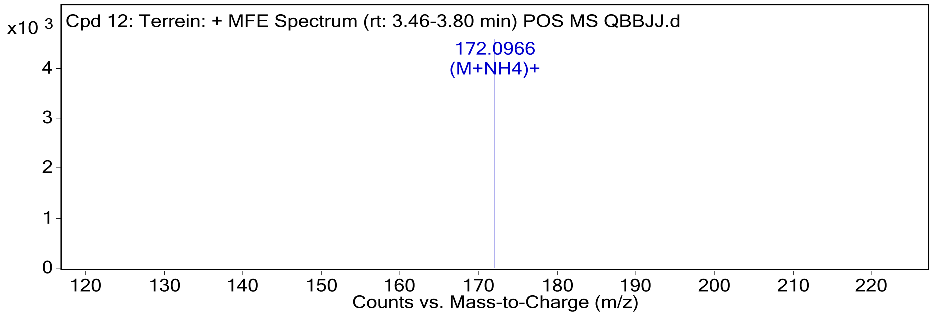

Qualitative Compound Report

Compound Label	Name	*m/z*	RT	Algorithm	Mass
Cpd 13: Austdiol	**Austdiol**	254.1021	4.3	Find by Molecular Feature	236.0683

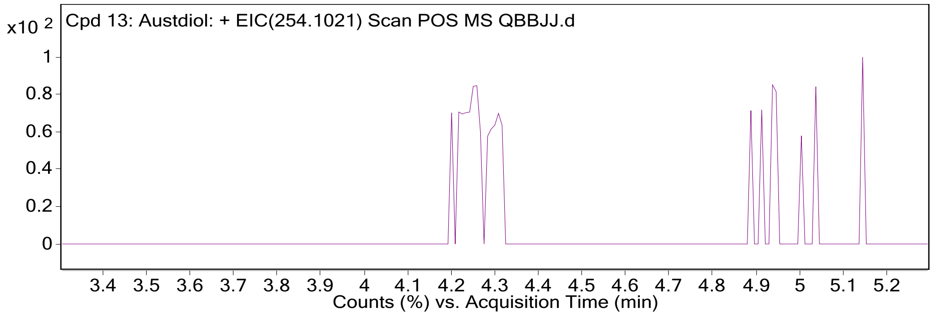

MFE MS Zoomed Spectrum

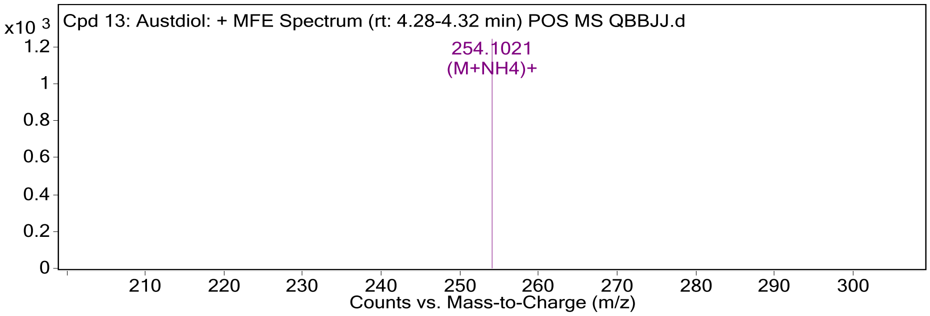

Compound Label	Name	*m/z*	RT	Algorithm	Mass
Cpd 14: Fusaric acid	**Fusaric acid**	197.1284	4.63	Find by Molecular Feature	179.0946

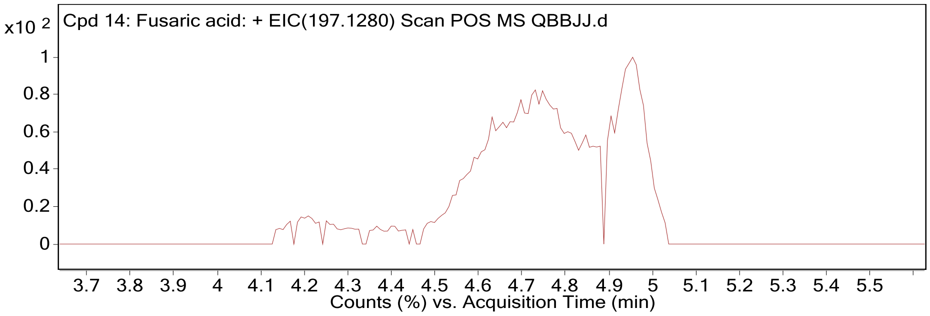

MFE MS Zoomed Spectrum

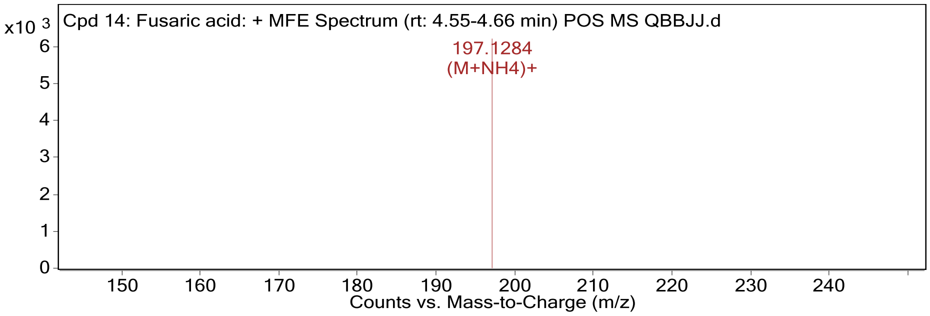

Compound Label	Name	*m/z*	RT	Algorithm	Mass
Cpd 15: Fusaric acid	**Fusaric acid**	197.1285	4.69	Find by Molecular Feature	179.0947

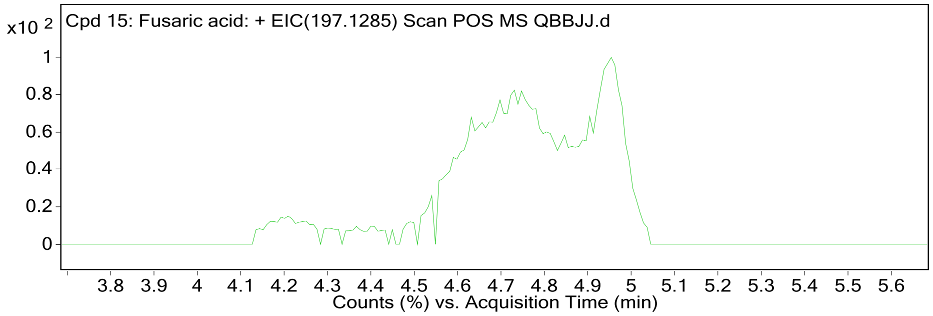

MFE MS Zoomed Spectrum

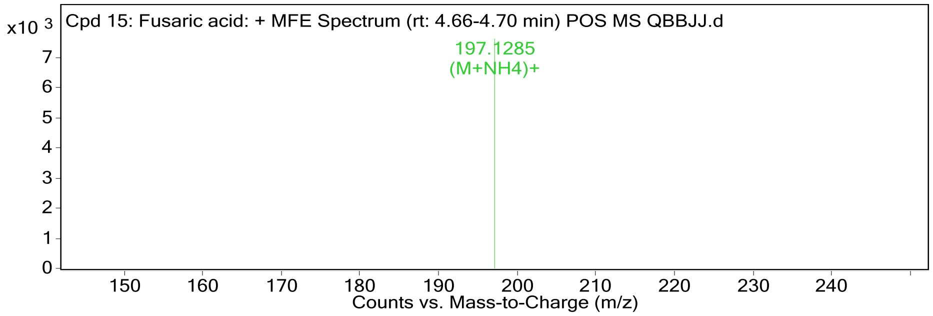

Compound Label	Name	*m/z*	RT	Algorithm	Mass
Cpd 16: Fusaric acid	**Fusaric acid**	197.1284	4.75	Find by Molecular Feature	179.0946

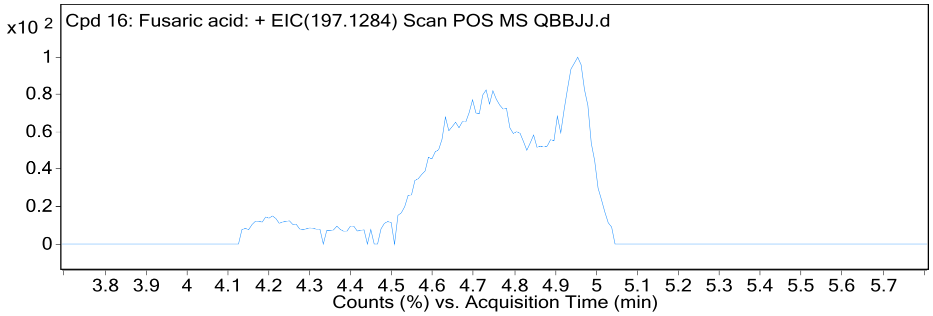

MFE MS Zoomed Spectrum

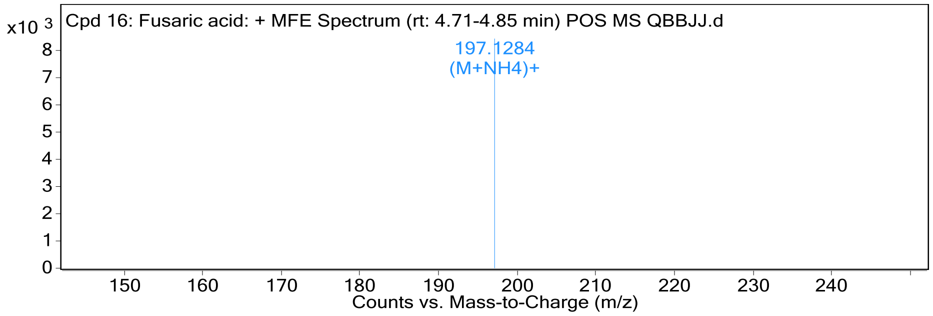

Compound Label	Name	*m/z*	RT	Algorithm	Mass
Cpd 17: Fusaric acid	**Fusaric acid**	197.1286	4.95	Find by Molecular Feature	179.0948

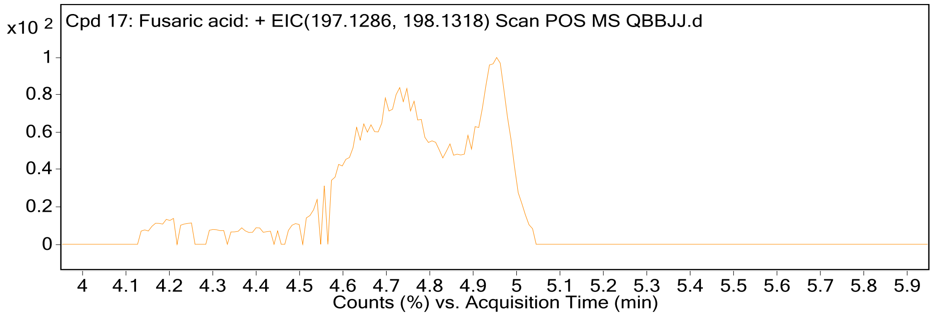

MFE MS Zoomed Spectrum

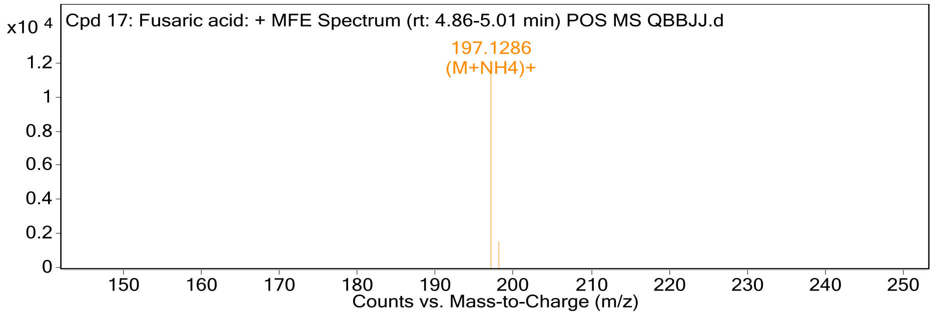

Compound Label	Name	*m/z*	RT	Algorithm	Mass
Cpd 18: Cerulenin	**Cerulenin**	241.1547	5.17	Find by Molecular Feature	223.1209

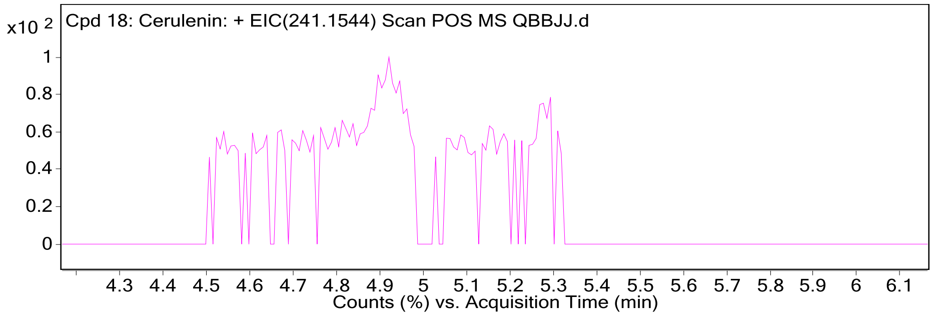

MFE MS Zoomed Spectrum

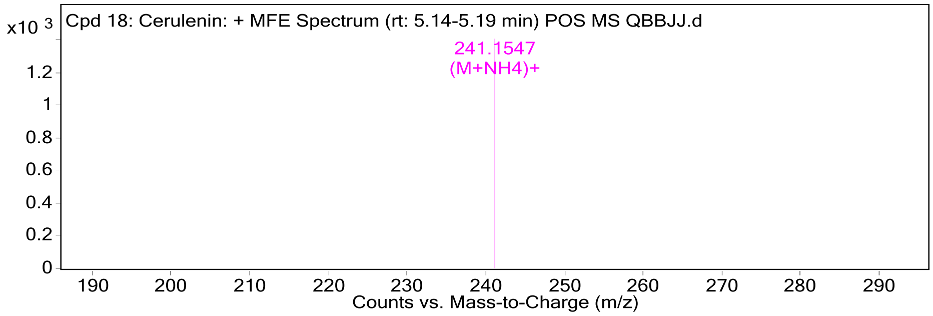

Compound Label	Name	*m/z*	RT	Algorithm	Mass

Cpd 19: Infectopyrone	**Infectopyrone**	282.1335	5.45	Find by Molecular Feature	264.0996

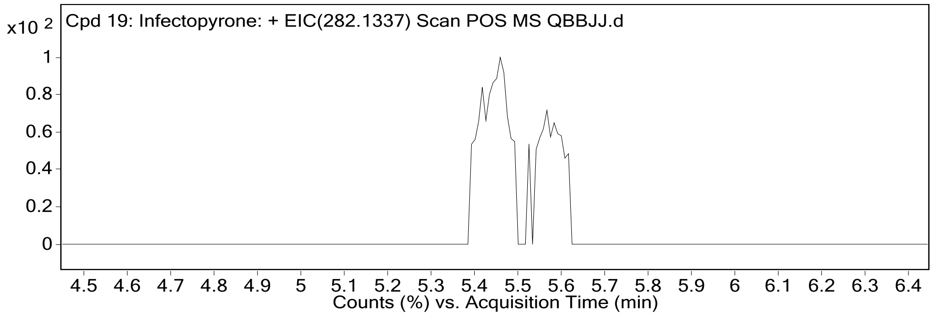

MFE MS Zoomed Spectrum

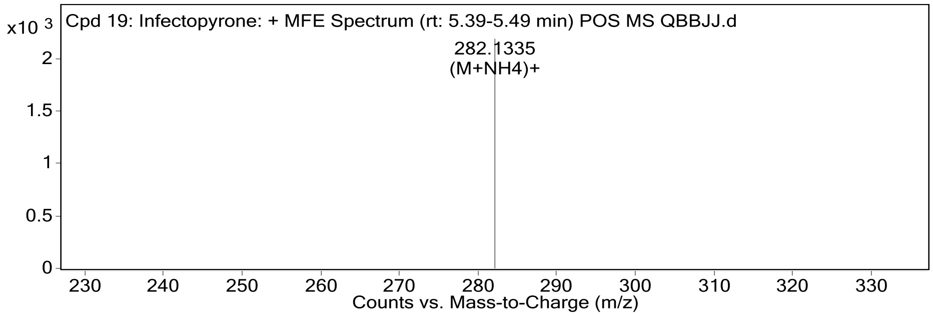

Compound Label	Name	*m/z*	RT	Algorithm	Mass
Cpd 20: 5-Methyl-mellein	**5-Methyl-mellein**	210.1124	5.46	Find by Molecular Feature	192.0785

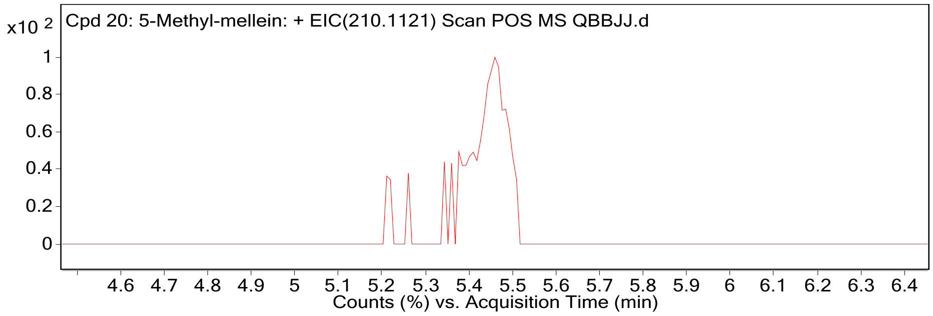

MFE MS Zoomed Spectrum

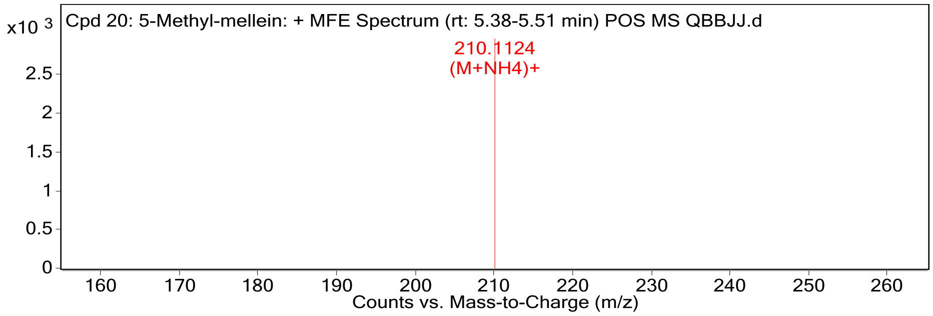

Qualitative Compound Report

Compound Label	Name	*m/z*	RT	Algorithm	Mass
Cpd 21: Fusaric acid	**Fusaric acid**	180.1021	5.6	Find by Molecular Feature	179.0948

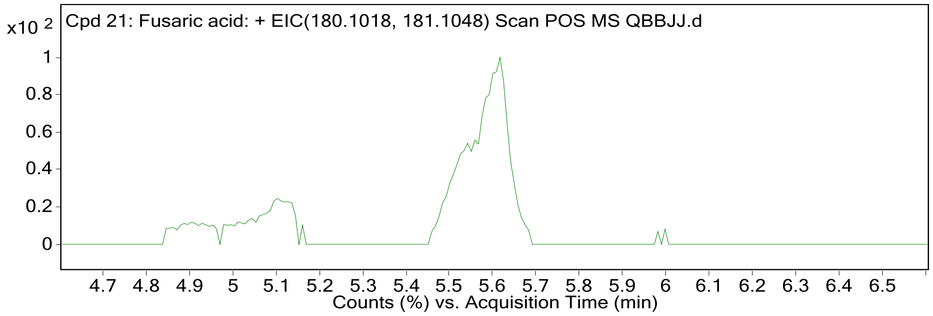

MFE MS Zoomed Spectrum

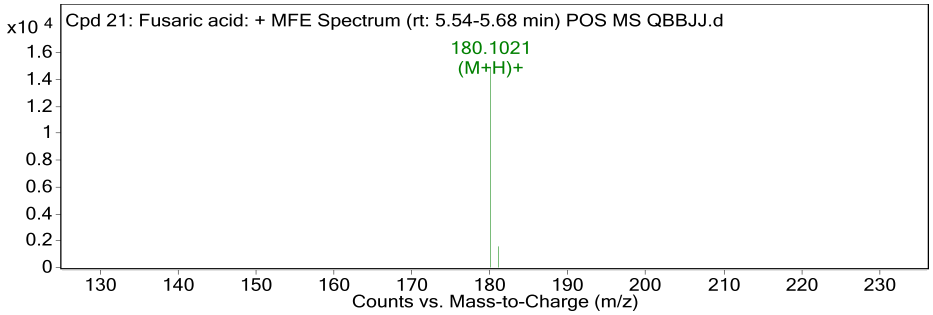

Compound Label	Name	*m/z*	RT	Algorithm	Mass
Cpd 22: Enniatin K1	**Enniatin K1**	648.3803	6.31	Find by Molecular Feature	625.391

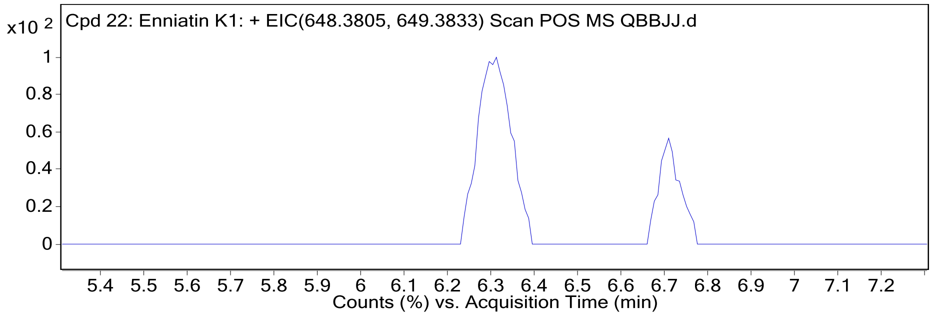

MFE MS Zoomed Spectrum

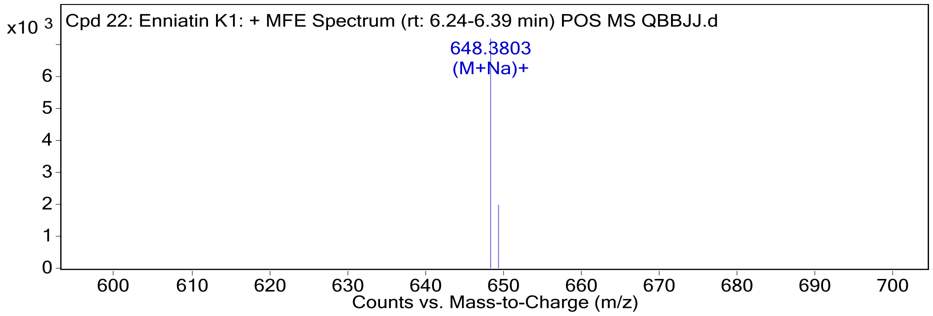

Qualitative Compound Report

Compound Label	Name	*m/z*	RT	Algorithm	Mass
Cpd 23: Brevianamid F	**Brevianamid F**	284.1397	6.35	Find by Molecular Feature	283.1324

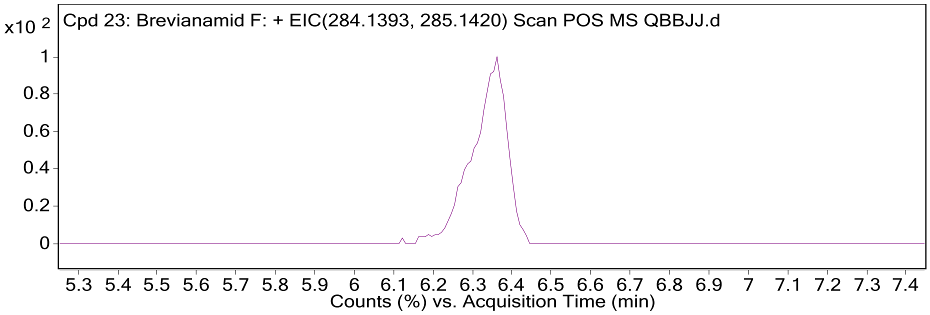

MFE MS Zoomed Spectrum

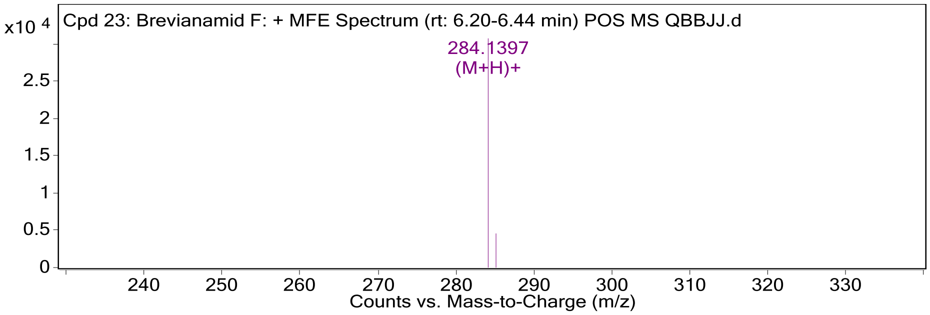

Compound Label	Name	*m/z*	RT	Algorithm	Mass
Cpd 24: Pyrenocine A	**Pyrenocine A**	226.1073	6.56	Find by Molecular Feature	208.0735

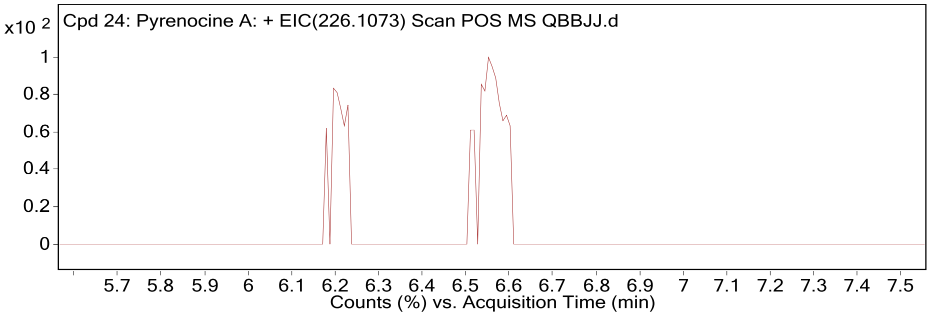

MFE MS Zoomed Spectrum

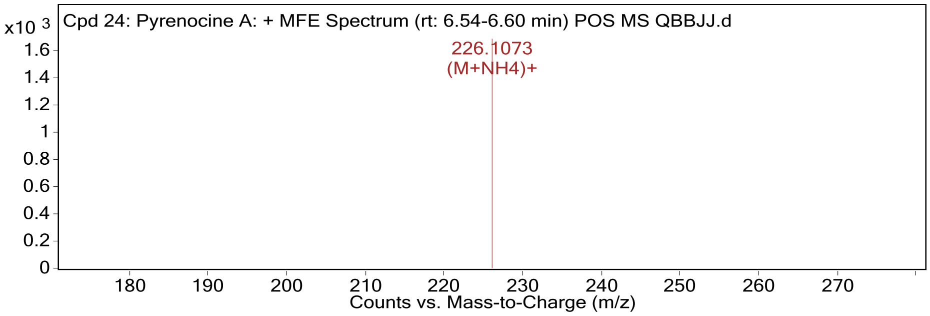

Compound Label	Name	*m/z*	RT	Algorithm	Mass
Cpd 25: Enniatin K1	**Enniatin K1**	648.3803	6.71	Find by Molecular Feature	625.3911

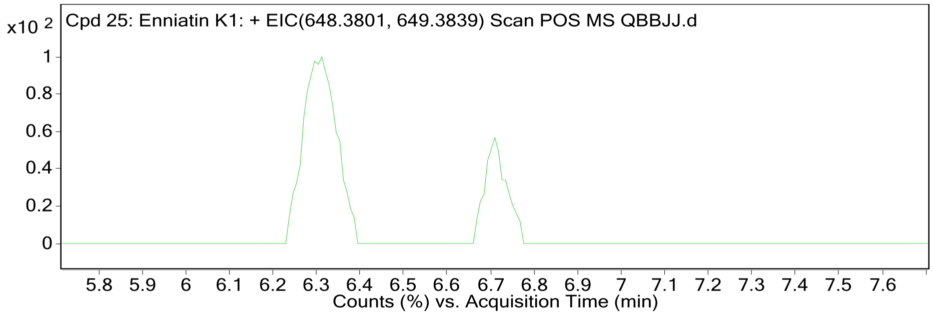

MFE MS Zoomed Spectrum

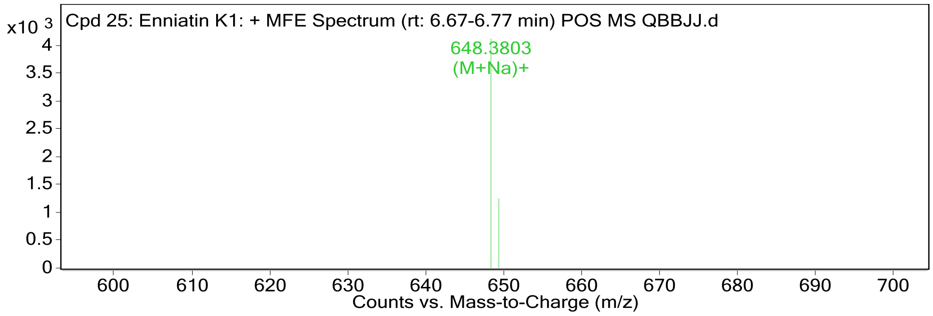

Compound Label	Name	*m/z*	RT	Algorithm	Mass
Cpd 26: Fusarinolic acid	**Fusarinolic acid**	196.0969	6.78	Find by Molecular Feature	195.0896

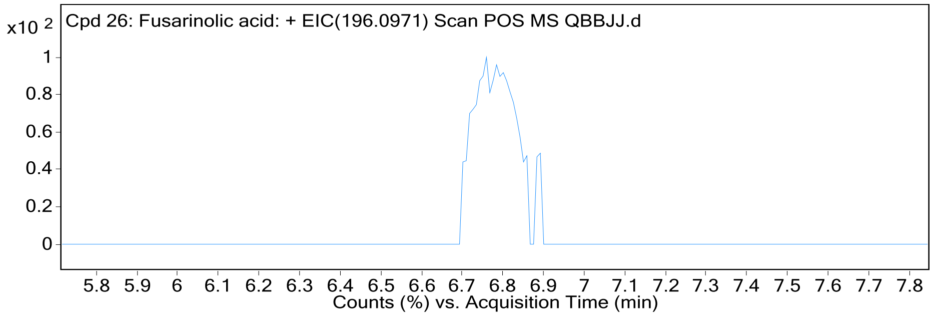

MFE MS Zoomed Spectrum

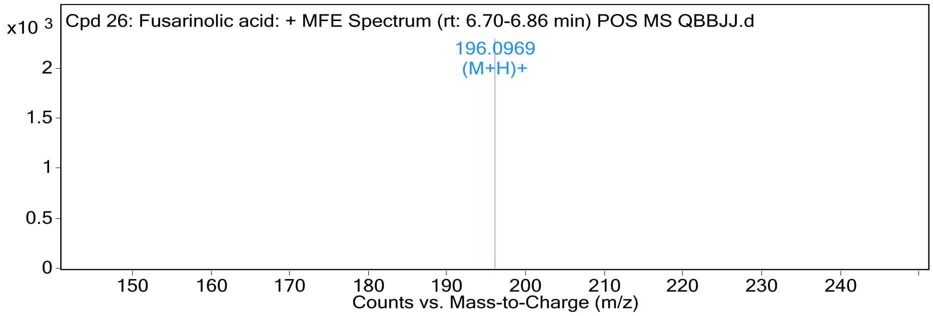

Qualitative Compound Report

Compound Label	Name	*m/z*	RT	Algorithm	Mass
Cpd 27: DAS / Diacetoxyscirpenol	**DAS / Diacetoxyscirpenol**	384.2016	7.22	Find by Molecular Feature	366.1678

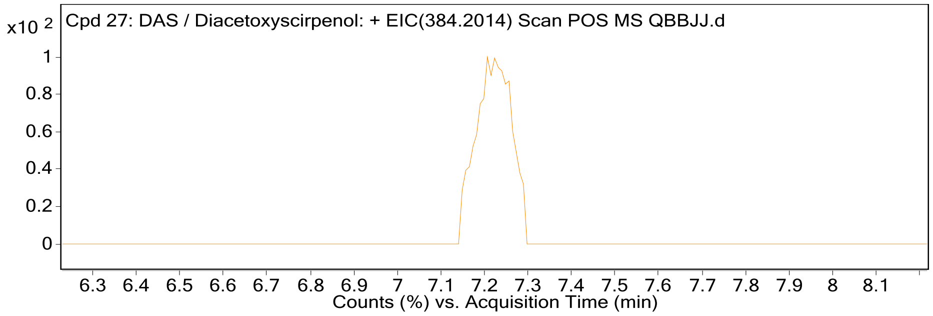

MFE MS Zoomed Spectrum

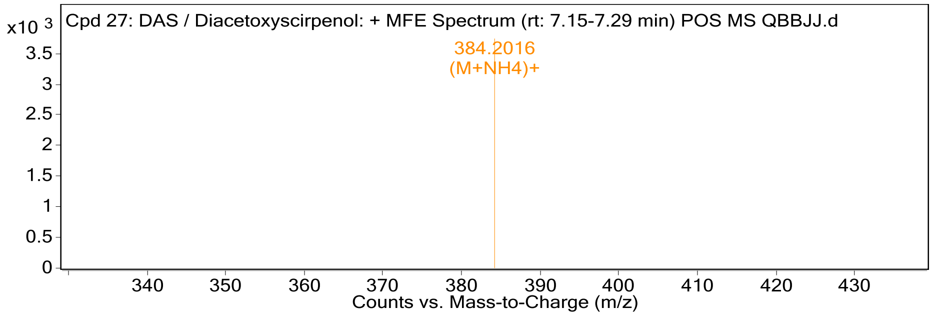

Compound Label	Name	*m/z*	RT	Algorithm	Mass
Cpd 28: Fusaric acid	**Fusaric acid**	180.1022	7.7	Find by Molecular Feature	179.0949

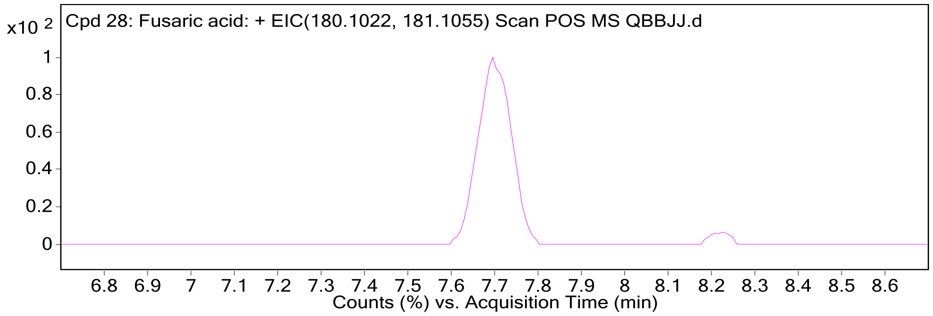

MFE MS Zoomed Spectrum

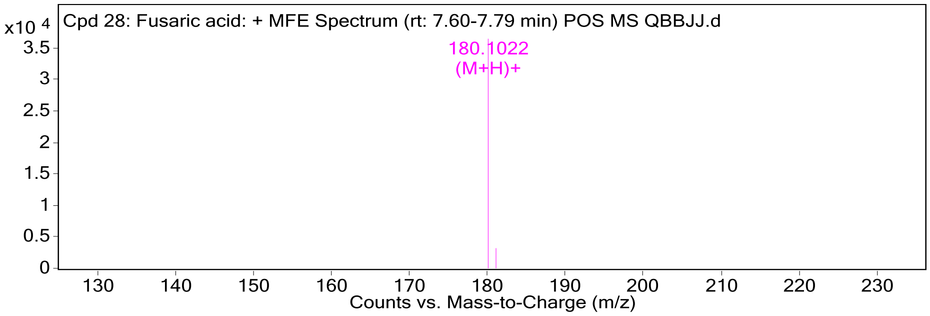

Compound Label	Name	*m/z*	RT	Algorithm	Mass
Cpd 29: beta-Zearalenol	**beta-Zearalenol**	338.196	7.76	Find by Molecular Feature	320.1622

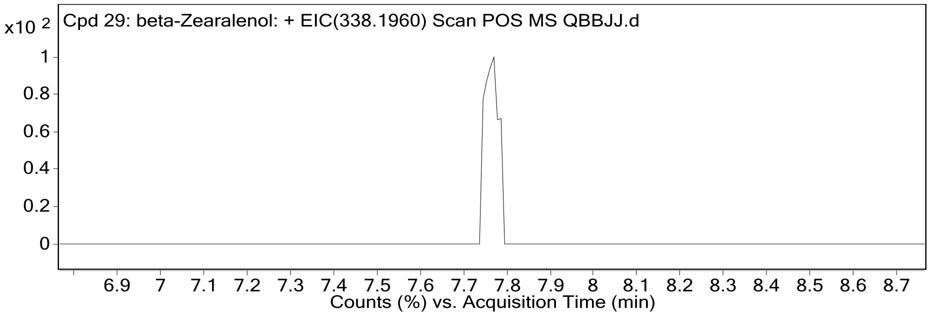

MFE MS Zoomed Spectrum

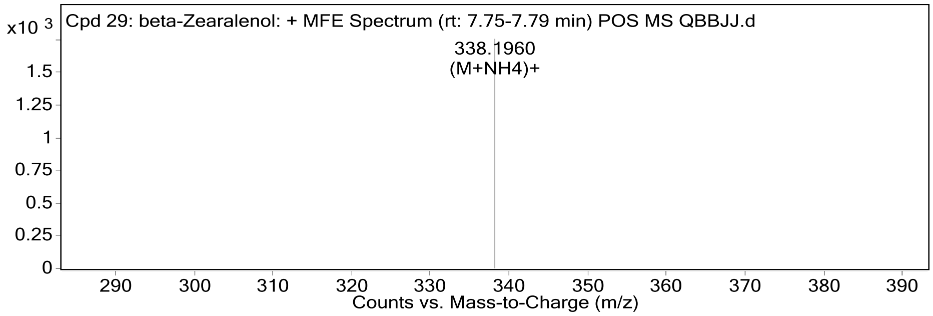

Compound Label	Name	*m/z*	RT	Algorithm	Mass
Cpd 30: 15-Hydroxyculmorone	**15-Hydroxyculmorone**	270.2064	7.93	Find by Molecular Feature	252.1725

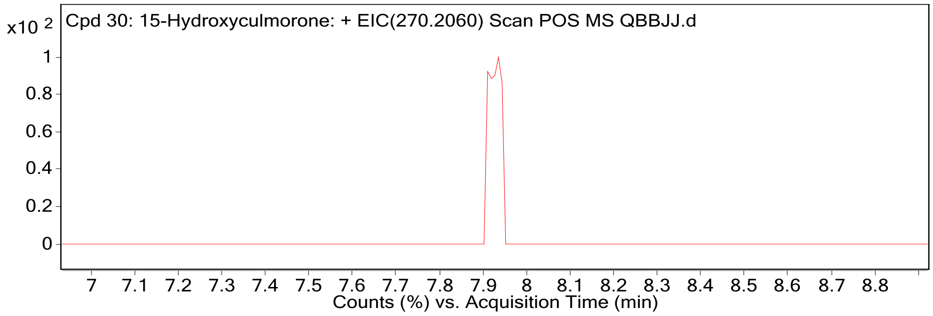

MFE MS Zoomed Spectrum

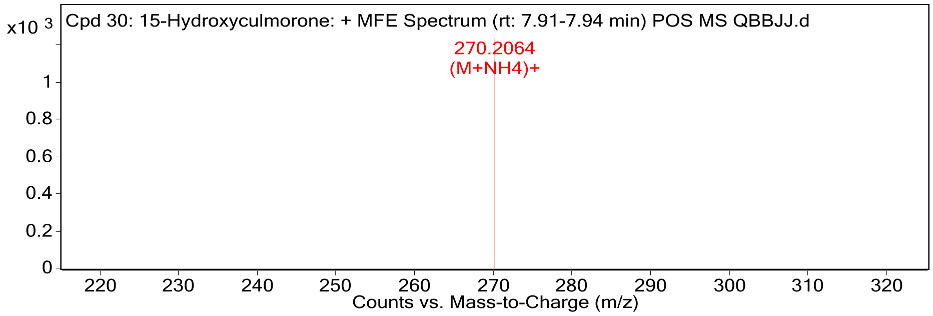

Compound Label	Name	*m/z*	RT	Algorithm	Mass
Cpd 31: Fusaric acid	**Fusaric acid**	180.1018	8.22	Find by Molecular Feature	179.0945

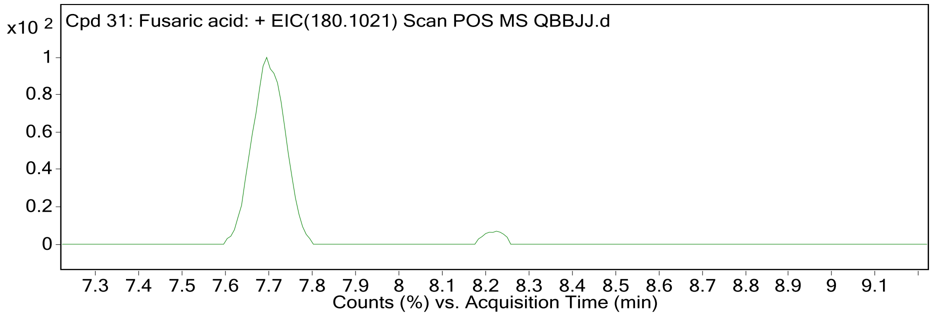

MFE MS Zoomed Spectrum

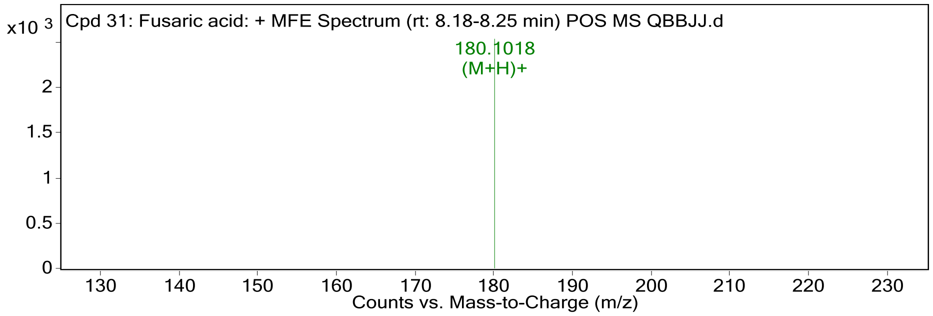

Compound Label	Name	*m/z*	RT	Algorithm	Mass
Cpd 32: Culmorin	**Culmorin**	256.2267	8.3	Find by Molecular Feature	238.1929

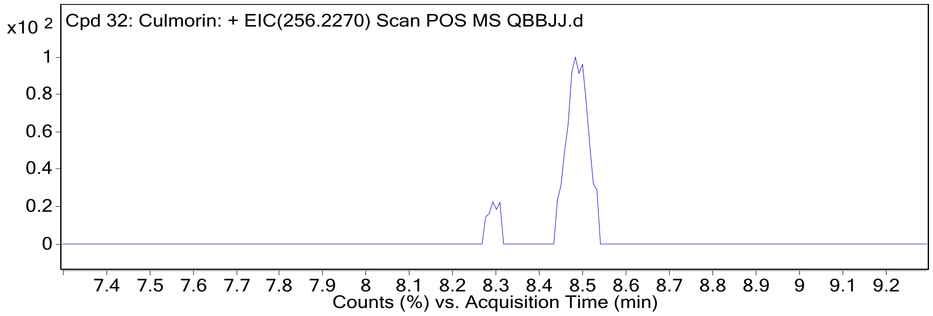

MFE MS Zoomed Spectrum

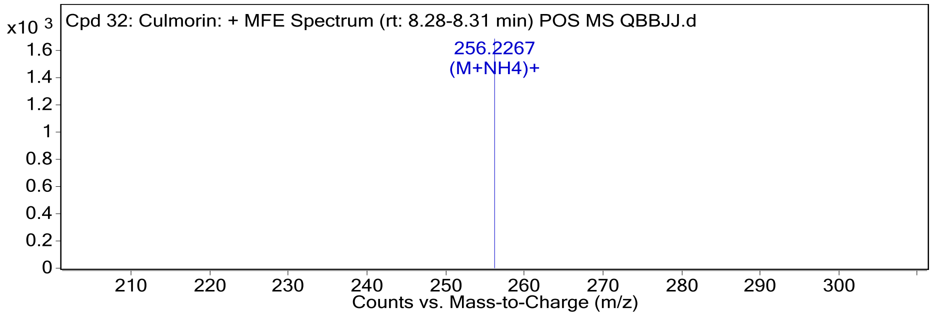

Compound Label	Name	*m/z*	RT	Algorithm	Mass
Cpd 33: Palitantin	**Palitantin**	272.1865	8.34	Find by Molecular Feature	254.1526

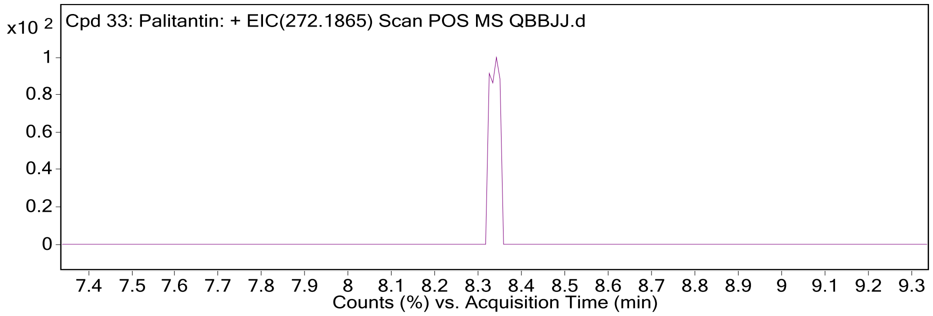

MFE MS Zoomed Spectrum

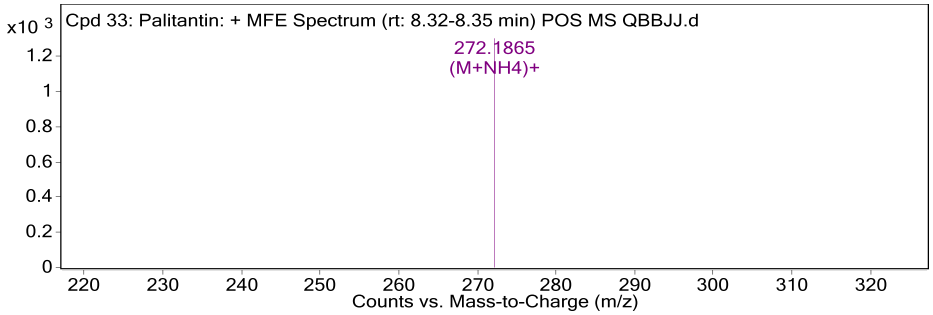

Compound Label	Name	*m/z*	RT	Algorithm	Mass
Cpd 34: FS-4	**FS-4**	268.1906	8.46	Find by Molecular Feature	250.1568

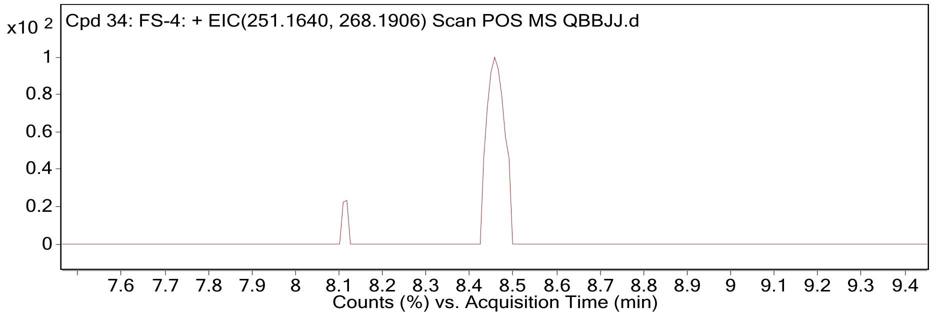

MFE MS Zoomed Spectrum

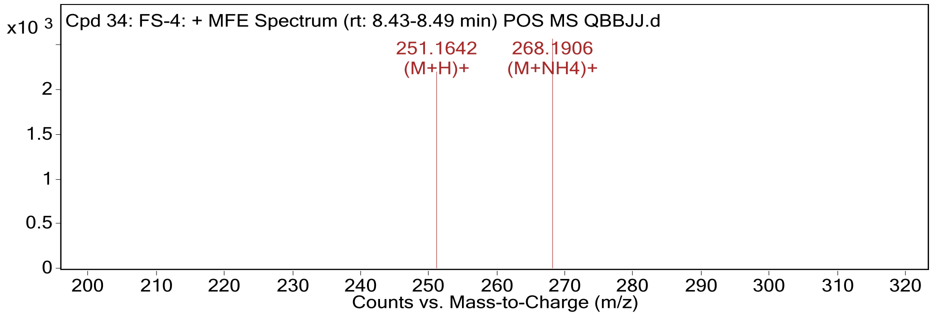

Compound Label	Name	*m/z*	RT	Algorithm	Mass

Cpd 35: Culmorin	**Culmorin**	256.2271	8.49	Find by Molecular Feature	238.1933

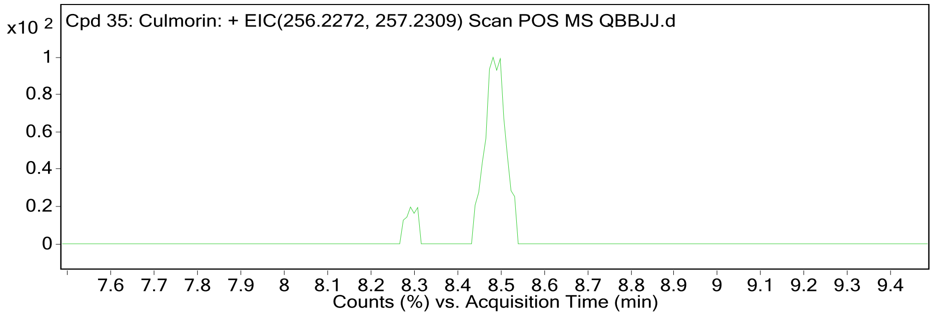

MFE MS Zoomed Spectrum

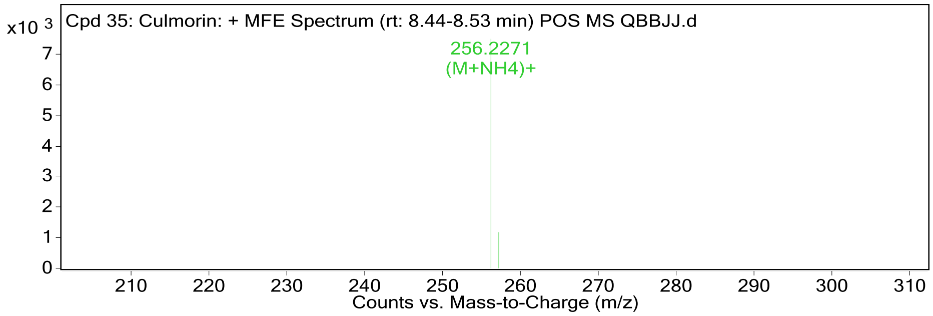

Compound Label	Name	*m/z*	RT	Algorithm	Mass
Cpd 36: Deepoxy deoxynivalenol	**Deepoxy deoxynivalenol**	281.1381	8.94	Find by Molecular Feature	280.1308

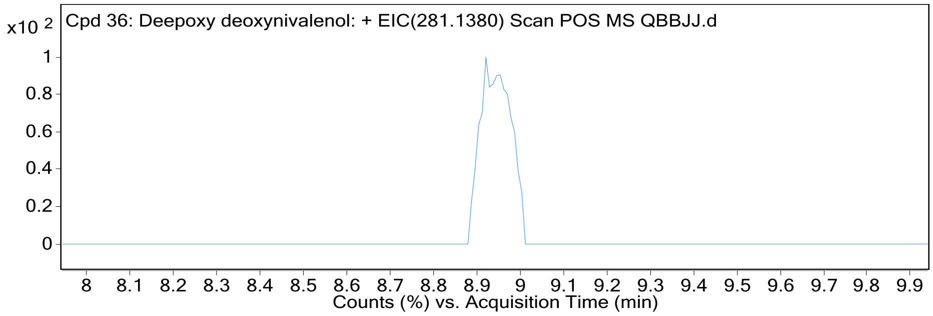

MFE MS Zoomed Spectrum

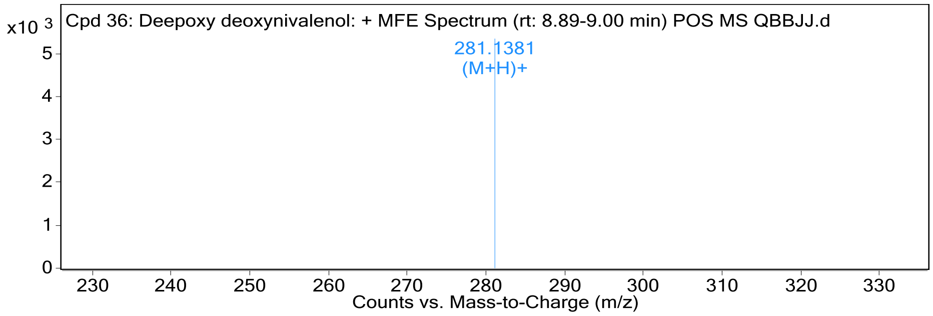

Qualitative Compound Report

Compound Label	Name	*m/z*	RT	Algorithm	Mass
Cpd 37: Paspaline	**Paspaline**	439.3308	9.71	Find by Molecular Feature	421.2957

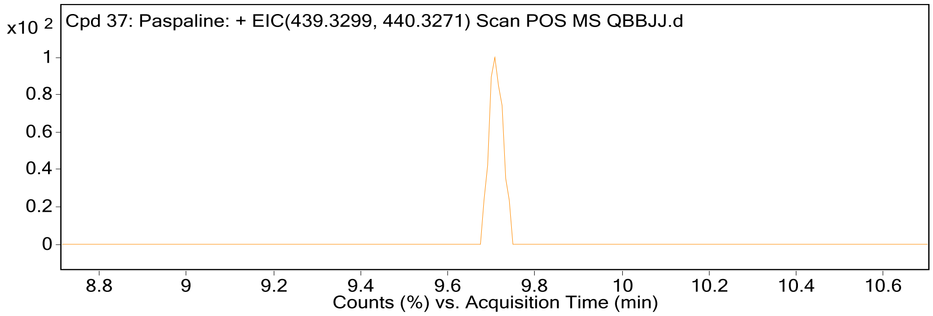

MFE MS Zoomed Spectrum

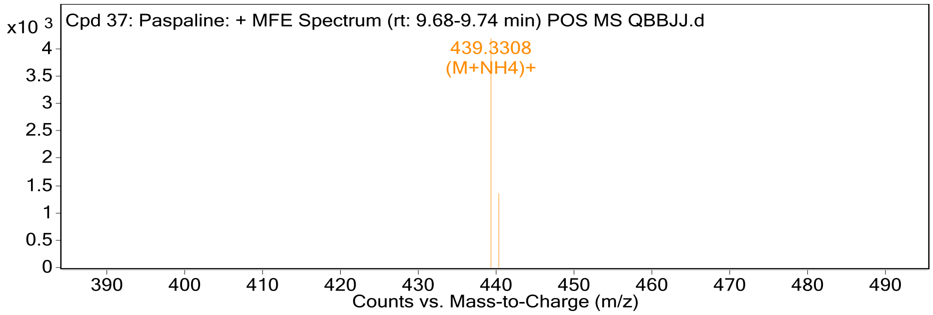

Compound Label	Name	*m/z*	RT	Algorithm	Mass
Cpd 38: Ionomycin	**Ionomycin**	731.5101	10.66	Find by Molecular Feature	708.5209

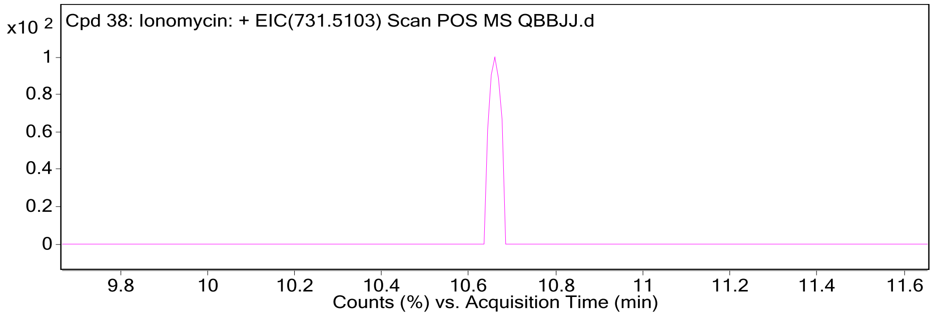

MFE MS Zoomed Spectrum

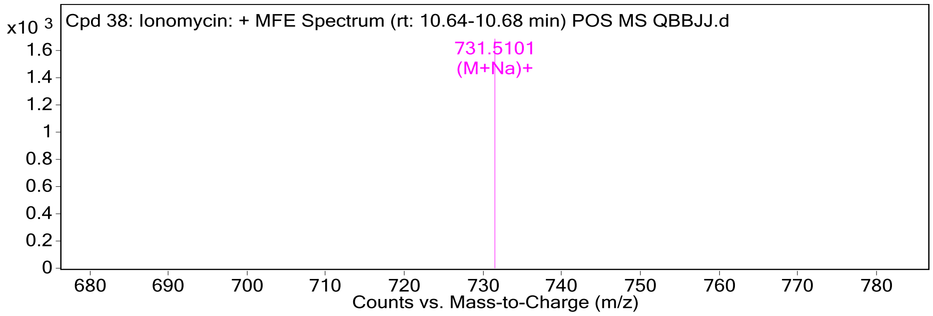

Qualitative Compound Report

Compound Label	Name	*m/z*	RT	Algorithm	Mass
Cpd 39: 2-Amino-14,16-dimethyloctadecan-3-ol	**2-Amino-14,16-dimethyloctadecan-3-**	336.3232	10.84	Find by Molecular Feature	313.334

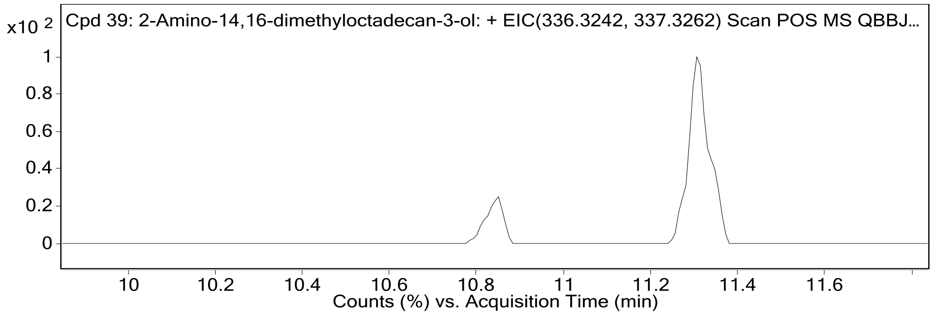

MFE MS Zoomed Spectrum

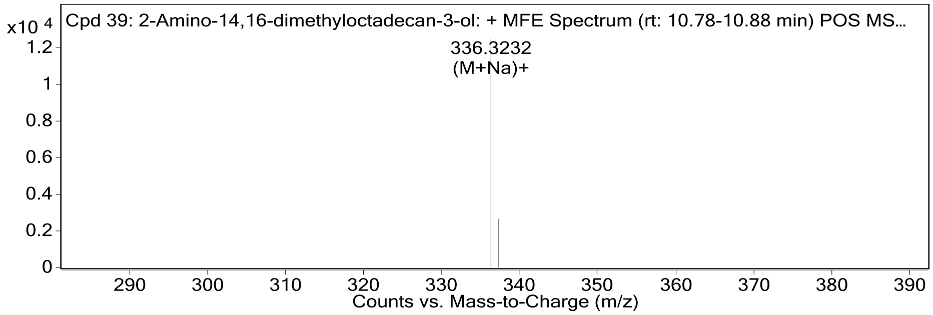

Compound Label	Name	*m/z*	RT	Algorithm	Mass
Cpd 40: Beauvericin	**Beauvericin**	801.4396	10.91	Find by Molecular Feature	783.4055

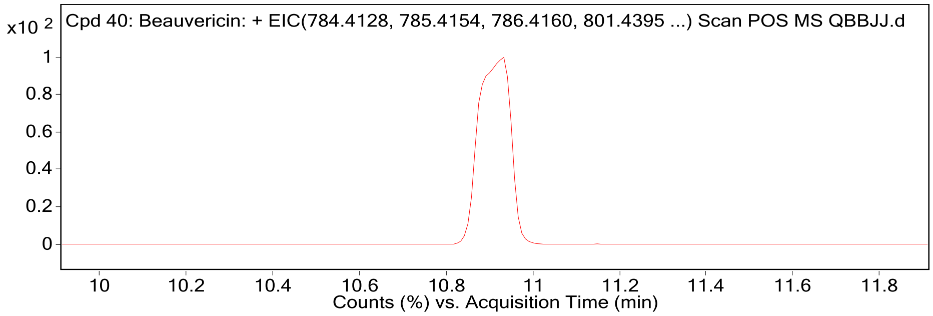

MFE MS Zoomed Spectrum

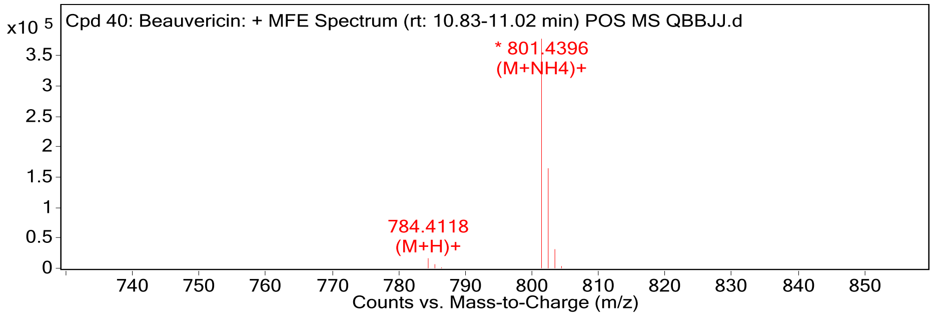

Compound Label	Name	*m/z*	RT	Algorithm	Mass
Cpd 41: Sirolimus (Rapamycin)	**Sirolimus (Rapamycin)**	931.5931	11.21	Find by Molecular Feature	913.5589

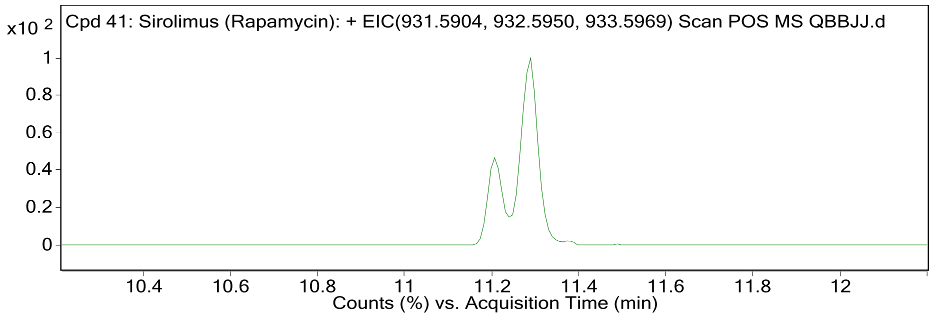

MFE MS Zoomed Spectrum

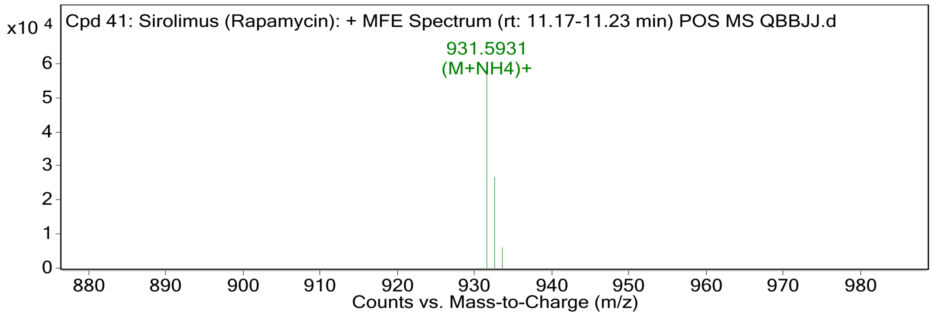

Compound Label	Name	*m/z*	RT	Algorithm	Mass
Cpd 42: Sirolimus (Rapamycin)	**Sirolimus (Rapamycin)**	931.5925	11.29	Find by Molecular Feature	913.5585

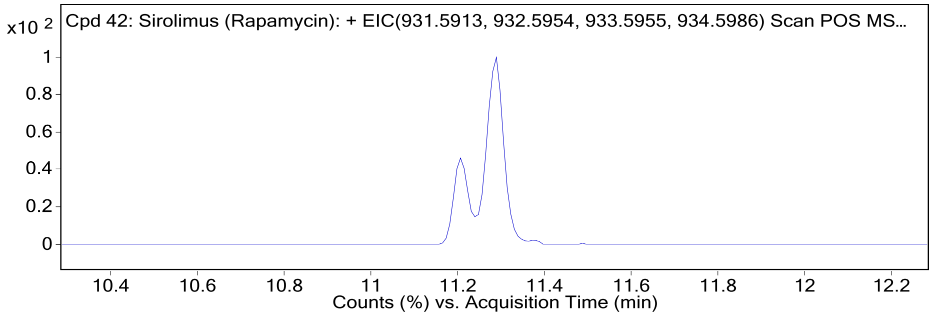

MFE MS Zoomed Spectrum

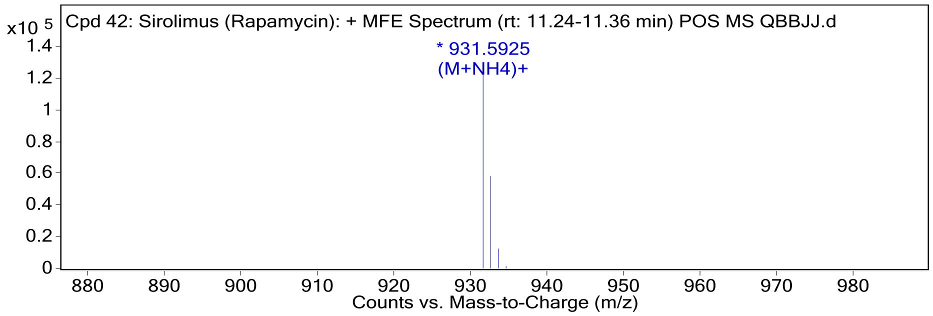

Compound Label	Name	*m/z*	RT	Algorithm	Mass
Cpd 43: Sirolimus (Rapamycin)	**Sirolimus (Rapamycin)**	931.5899	11.38	Find by Molecular Feature	913.5561

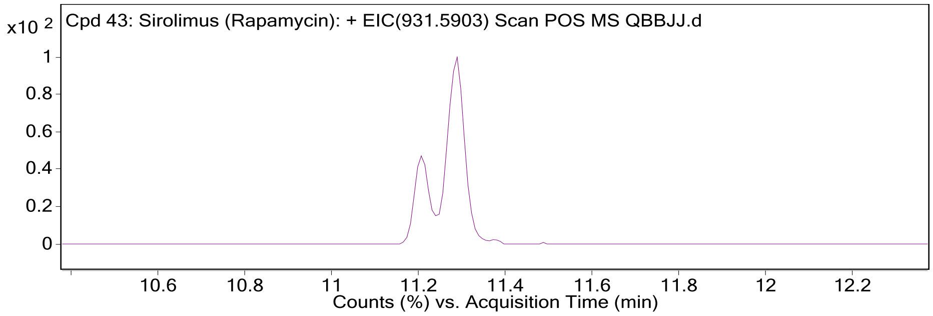

MFE MS Zoomed Spectrum

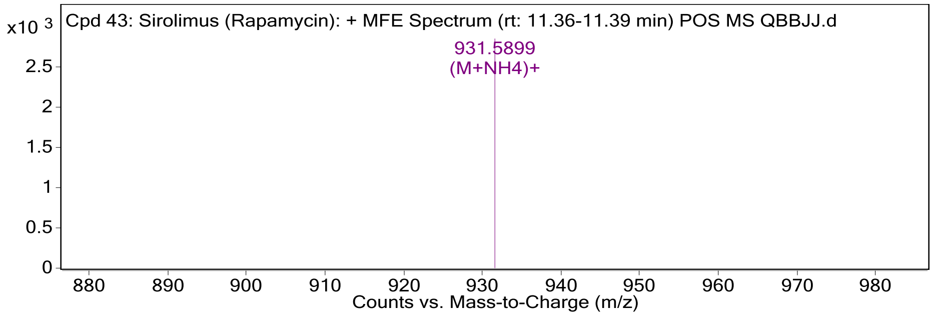

--- End Of Report ---

Qualitative Compound Report

**Data File**	POS MS HT21.d	**Sample Name**	HT21
**Sample Type**	Sample	**Position**	P1-A2
**Instrument Name**	Instrument 1	**User Name**	
**Acq Method**	Mycotoxins POS.m	**Acquired Time**	6/15/2022 11:20:35 AM
**IRM Calibration Status**	Success	**DA Method**	scau default.m
**Comment**			

**Sample Group**		**Info.**	
**Stream Name**	LC 1	**Acquisition SW Version**	6200 series TOF/6500 series Q-TOF B.06.01 (B6157)

Fragmentor Voltage 130 **Collision Energy** 0 **Ionization Mode** ESI

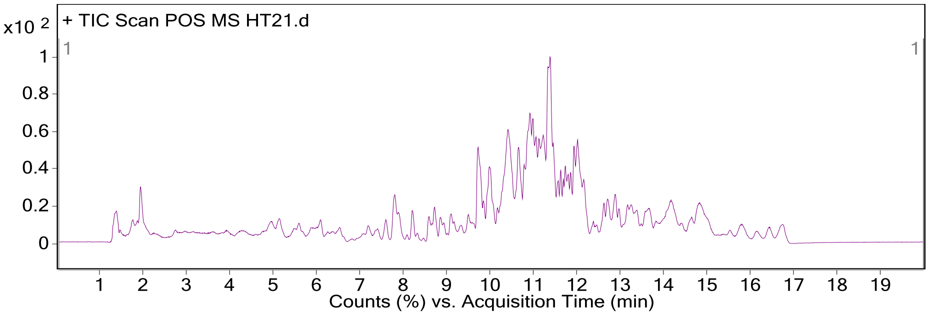

Fragmentor Voltage 130 **Collision Energy** 0 **Ionization Mode** ESI

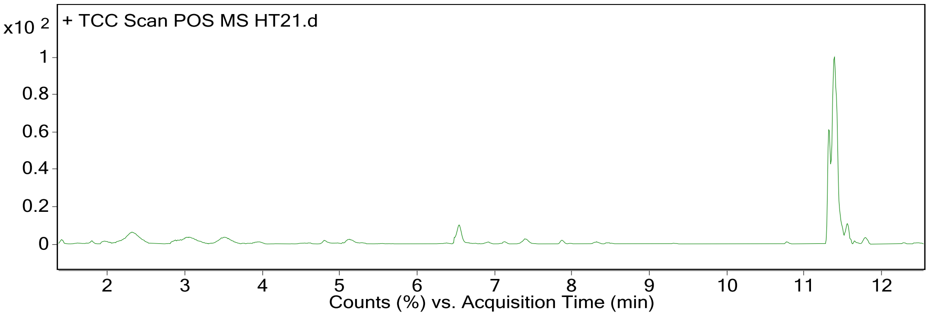

Compound Table

Compound Label	RT	Mass	Name	Formula	Tgt Mass	Diff (ppm)	SignificantIonMz
Cpd 1: Fusarinolic acid	1.4	195.0895	Fusarinolic acid	C10 H13 N O3	195.0895	-0.13	213.1233
Cpd 2: Terrein	1.42	154.0629	Terrein	C8 H10 O3	154.063	-0.88	172.0967
Cpd 3: Chlamydospordiol	1.63	228.0996	Chlamydospordiol	C11 H16 O5	228.0998	-0.58	246.1335
Cpd 4: Cerulenin	1.8	223.1211	Cerulenin	C12 H17 N O3	223.1208	1.25	241.1549
Cpd 5: Fusarinolic acid	1.8	195.0895	Fusarinolic acid	C10 H13 N O3	195.0895	-0.31	213.1233
Cpd 6: Fusarinolic acid	1.95	195.0883	Fusarinolic acid	C10 H13 N O3	195.0895	-6.59	196.0971
Cpd 7: 5-Methyl-mellein	1.95	192.0788	5-Methyl-mellein	C11 H12 O3	192.0786	0.92	210.1126
Cpd 8: DON / Deoxynivalenol	2	296.1263	DON / Deoxynivalenol	C15 H20 O6	296.126	1.18	314.1602
Cpd 9: Asperlactone	2.06	184.0737	Asperlactone	C9 H12 O4	184.0736	0.59	202.1075
Cpd 10: Asperlactone	2.07	184.0738	Asperlactone	C9 H12 O4	184.0736	1.15	185.081
Cpd 11: 10,11-Dehydrocurvularin	2.13	290.1156	10,11-Dehydrocurvularin	C16 H18 O5	290.1154	0.67	308.1494
Cpd 12: Austdiol	2.15	236.0685	Austdiol	C12 H12 O5	236.0685	0.13	254.1023
Cpd 13: [U-13C]-NIV / Nivalenol	2.18	327.1707	[U-13C]-NIV / Nivalenol	[13C]15 H20 O7	327.1712	-1.47	350.16
Cpd 14: Dechlorogriseofulvin	2.19	318.1104	Dechlorogriseofulvin	C17 H18 O6	318.1103	0.16	336.1442

Cpd 15: Decarestrictine D	2.33	216.0999	Decarestrictine D	C10 H16 O5	216.0998	0.65	234.1337
Cpd 16: Tenuazonic acid	2.34	197.1052	Tenuazonic acid	C10 H15 N O3	197.1052	0.07	198.1125
Cpd 17: 10,11-Dehydrocurvularin	2.94	290.1155	10,11-Dehydrocurvularin	C16 H18 O5	290.1154	0.28	308.1493
Cpd 18: Pyrenocine A	3.03	208.0735	Pyrenocine A	C11 H12 O4	208.0736	-0.37	226.1073
Cpd 19: Terrein	3.04	154.0631	Terrein	C8 H10 O3	154.063	0.61	172.0969
Cpd 20: Austdiol	3.13	236.0685	Austdiol	C12 H12 O5	236.0685	0.01	254.1023
Cpd 21: [U-13C]-NIV / Nivalenol	3.17	327.1706	[U-13C]-NIV / Nivalenol	[13C]15 H20 O7	327.1712	-2.03	350.1598
Cpd 22: Antimycin	3.27	250.0839	Antimycin	C13 H14 O5	250.0841	-0.77	268.1178
Cpd 23: 10,11-Dehydrocurvularin	3.51	290.1156	10,11-Dehydrocurvularin	C16 H18 O5	290.1154	0.68	308.1495
Cpd 24: Rubrofusarin	3.51	272.0686	Rubrofusarin	C15 H12 O5	272.0685	0.33	290.1024
Cpd 25: Pyrenocine A	3.54	208.0734	Pyrenocine A	C11 H12 O4	208.0736	-0.76	226.1072
Cpd 26: Decarestrictine D	3.58	216.0996	Decarestrictine D	C10 H16 O5	216.0998	-0.97	234.1334
Cpd 27: Decarestrictine D	3.75	216.1	Decarestrictine D	C10 H16 O5	216.0998	1.24	234.1339
Cpd 28: Austdiol	3.76	236.0685	Austdiol	C12 H12 O5	236.0685	-0.03	254.1023
Cpd 29: Decarestrictine D	3.93	216.0998	Decarestrictine D	C10 H16 O5	216.0998	0.27	234.1337
Cpd 30: Terrein	3.96	154.0632	Terrein	C8 H10 O3	154.063	1.15	172.097
Cpd 31: Asterric acid	4.15	348.0846	Asterric acid	C17 H16 O8	348.0845	0.15	366.1184
Cpd 32: Pyrenocine A	4.38	208.0738	Pyrenocine A	C11 H12 O4	208.0736	1.04	226.1076
Cpd 33: Fusaric acid	4.52	179.0947	Fusaric acid	C10 H13 N O2	179.0946	0.5	197.1285
Cpd 34: Austdiol	4.54	236.0686	Austdiol	C12 H12 O5	236.0685	0.34	254.1024
Cpd 35: Fusaric acid	4.59	179.0946	Fusaric acid	C10 H13 N O2	179.0946	-0.04	197.1284
Cpd 36: 10,11-Dehydrocurvularin	4.62	290.1155	10,11-Dehydrocurvularin	C16 H18 O5	290.1154	0.33	308.1493
Cpd 37: 10,11-Dehydrocurvularin	4.8	290.1156	10,11-Dehydrocurvularin	C16 H18 O5	290.1154	0.53	308.1495
Cpd 38: Fusaric acid	4.91	179.0947	Fusaric acid	C10 H13 N O2	179.0946	0.59	197.1286
Cpd 39: Austdiol	5.09	236.0684	Austdiol	C12 H12 O5	236.0685	-0.29	254.1022
Cpd 40: Pyrenocine A	5.09	208.0736	Pyrenocine A	C11 H12 O4	208.0736	-0.04	226.1074
Cpd 41: Oosporein	5.13	306.0379	Oosporein	C14 H10 O8	306.0376	1.12	307.0452
Cpd 42: Asterric acid	5.14	348.0849	Asterric acid	C17 H16 O8	348.0845	1.07	366.1187
Cpd 43: Fusaric acid	5.17	179.0949	Fusaric acid	C10 H13 N O2	179.0946	1.27	197.1287
Cpd 44: Tenuazonic acid	5.29	197.1052	Tenuazonic acid	C10 H15 N O3	197.1052	0.01	198.1125
Cpd 45: Cerulenin	5.31	223.1209	Cerulenin	C12 H17 N O3	223.1208	0.17	241.1547
Cpd 46: Infectopyrone	5.56	264.0997	Infectopyrone	C14 H16 O5	264.0998	-0.37	282.1335
Cpd 47: Pyrenocine A	6.3	208.0734	Pyrenocine A	C11 H12 O4	208.0736	-0.66	226.1072
Cpd 48: Pyrenocine A	6.38	208.0737	Pyrenocine A	C11 H12 O4	208.0736	0.8	226.1076
Cpd 49: Brevianamid F	6.54	283.1322	Brevianamid F	C16 H17 N3 O2	283.1321	0.52	284.1395
Cpd 50: Paspalic acid	6.65	268.121	Paspalic acid	C16 H16 N2 O2	268.1212	-0.63	286.1548
Cpd 51: Pyrenocine A	6.74	208.0737	Pyrenocine A	C11 H12 O4	208.0736	0.44	226.1075
Cpd 52: Infectopyrone	6.89	264.1006	Infectopyrone	C14 H16 O5	264.0998	3.28	282.1345
Cpd 53: Anisomycin	6.93	265.1319	Anisomycin	C14 H19 N O4	265.1314	1.81	266.1392
Cpd 54: Macrosporin	7.12	284.0672	Macrosporin	C16 H12 O5	284.0685	-4.6	307.0564
Cpd 55: Aphidicolin	7.13	338.2438	Aphidicolin	C20 H34 O4	338.2457	-5.53	377.2072
Cpd 56: DAS / Diacetoxyscirpenol	7.39	366.1663	DAS / Diacetoxyscirpenol	C19 H26 O7	366.1679	-4.2	384.2002
Cpd 57: Rugulosuvine	7.41	333.1463	Rugulosuvine	C20 H19 N3 O2	333.1477	-4.3	334.1536
Cpd 58: Antibiotic L696,474	7.44	477.2898	Antibiotic L696,474	C30 H39 N O4	477.2879	3.87	516.253
Cpd 59: beta-Zearalenol	7.87	320.1607	beta-Zearalenol	C18 H24 O5	320.1624	-5.08	338.1946
Cpd 60: Isofusidienol A	7.98	300.0623	Isofusidienol A	C16 H12 O6	300.0634	-3.74	318.0961
Cpd 61: [U-13C]-ZEN / Zearalenone	8.27	336.2079	[U-13C]-ZEN / Zearalenone	[13C]18 H22 O5	336.2071	2.2	359.1971
Cpd 62: Fusaric acid	8.32	179.0939	Fusaric acid	C10 H13 N O2	179.0946	-4.29	180.1011
Cpd 63: Culmorin	8.45	238.1924	Culmorin	C15 H26 O2	238.1933	-3.66	256.2262
Cpd 64: Doxycycline	8.5	444.1518	Doxycycline	C22 H24 N2 O8	444.1533	-3.3	462.1856
Cpd 65: Stachybotrylactam	9.32	385.227	Stachybotrylactam	C23 H31 N O4	385.2253	4.35	403.2608
Cpd 66: FB6 / Fumonisin B6	10.78	721.3897	FB6 / Fumonisin B6	C34 H59 N O15	721.3885	1.67	722.3972
Cpd 67: Ionomycin	10.79	708.5202	Ionomycin	C41 H72 O9	708.5176	3.62	731.5094
Cpd 68: Sirolimus (Rapamycin)	11.32	913.5581	Sirolimus (Rapamycin)	C51 H79 N O13	913.5551	3.2	931.5915
Cpd 69: Sirolimus (Rapamycin)	11.4	913.5587	Sirolimus (Rapamycin)	C51 H79 N O13	913.5551	3.94	931.5926
Cpd 70: Tryptoquialanine	11.49	518.1811	Tryptoquialanine	C27 H26 N4 O7	518.1802	1.89	536.2158

Cpd 71: Myriocin	11.51	401.2783	Myriocin	C21 H39 N O6	401.2777	1.36	419.3121
Cpd 72: Sirolimus (Rapamycin)	11.56	913.5585	Sirolimus (Rapamycin)	C51 H79 N O13	913.5551	3.68	931.5925
Cpd 73: [U-13C]-FB1 / Fumonisin B1	11.65	755.4989	[U-13C]-FB1 / Fumonisin B1	[13C]34 H59 N O15	755.5025	-4.78	773.5327
Cpd 74: Sirolimus (Rapamycin)	11.66	913.5578	Sirolimus (Rapamycin)	C51 H79 N O13	913.5551	2.9	931.5916
Cpd 75: Sirolimus (Rapamycin)	11.78	913.5574	Sirolimus (Rapamycin)	C51 H79 N O13	913.5551	2.47	931.5913
Cpd 76: [U-13C]-FB2 / Fumonisin B2	11.78	739.5049	[U-13C]-FB2 / Fumonisin B2	[13C]34 H59 N O14	739.5076	-3.67	757.5384
Cpd 77: Fusapyrone	11.81	606.3791	Fusapyrone	C34 H54 O9	606.3768	3.84	607.3863
Cpd 78: Sirolimus (Rapamycin)	12.29	913.5567	Sirolimus (Rapamycin)	C51 H79 N O13	913.5551	1.7	931.5909
Cpd 79: Sirolimus (Rapamycin)	12.46	913.5586	Sirolimus (Rapamycin)	C51 H79 N O13	913.5551	3.76	931.5924

Compound Label	Name	*m/z*	RT	Algorithm	Mass
Cpd 1: Fusarinolic acid	**Fusarinolic acid**	213.1233	1.4	Find by Molecular Feature	195.0895

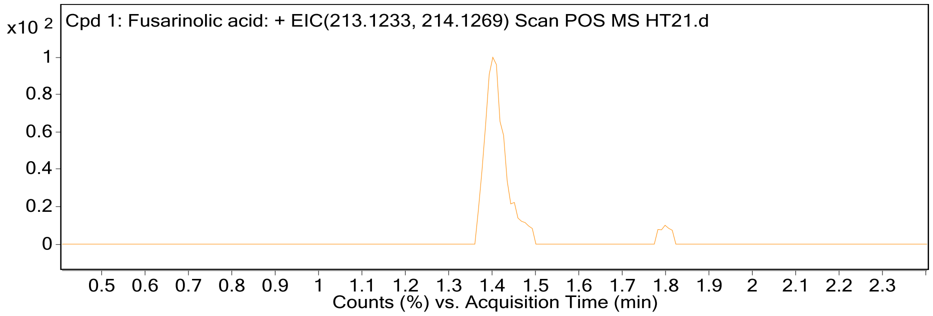

MFE MS Zoomed Spectrum

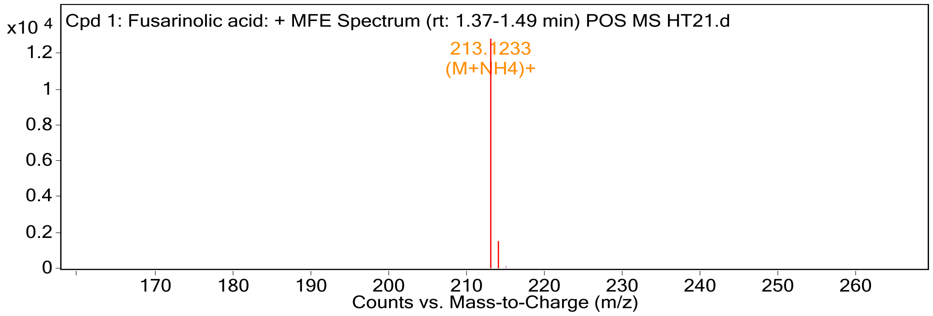

Compound Label	Name	*m/z*	RT	Algorithm	Mass
Cpd 2: Terrein	**Terrein**	172.0967	1.42	Find by Molecular Feature	154.0629

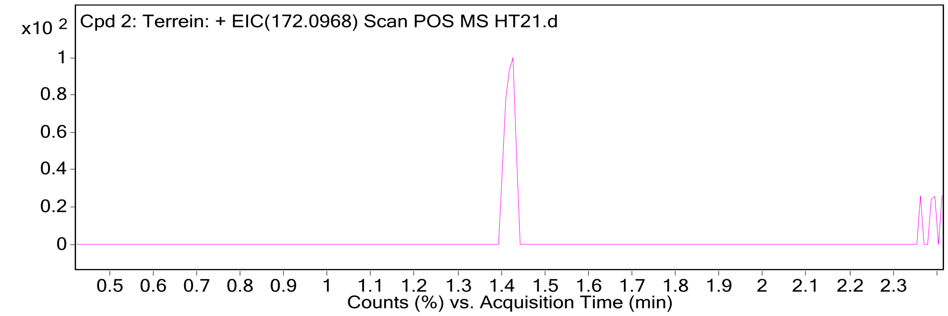

MFE MS Zoomed Spectrum

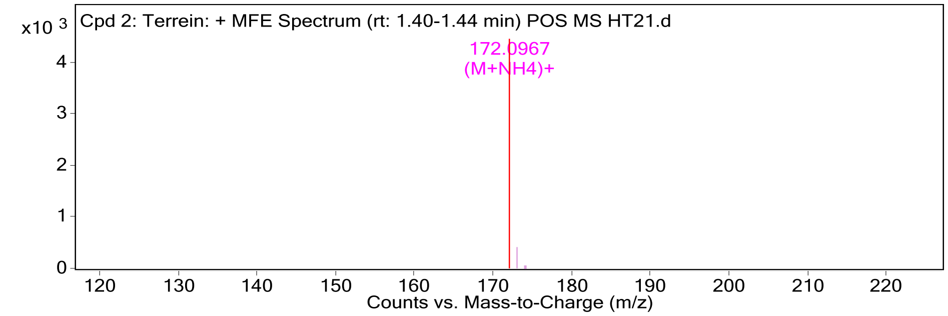

Compound Label	Name	*m/z*	RT	Algorithm	Mass
Cpd 3: Chlamydospordiol	**Chlamydospordiol**	246.1335	1.63	Find by Molecular Feature	228.0996

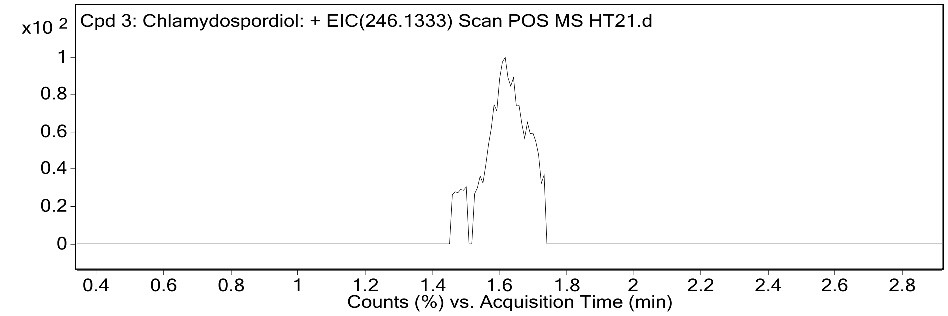

MFE MS Zoomed Spectrum

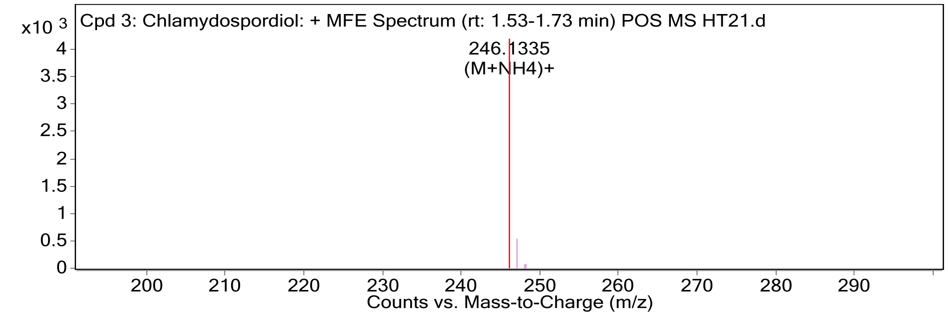

Compound Label	Name	*m/z*	RT	Algorithm	Mass
Cpd 4: Cerulenin	**Cerulenin**	241.1549	1.8	Find by Molecular Feature	223.1211

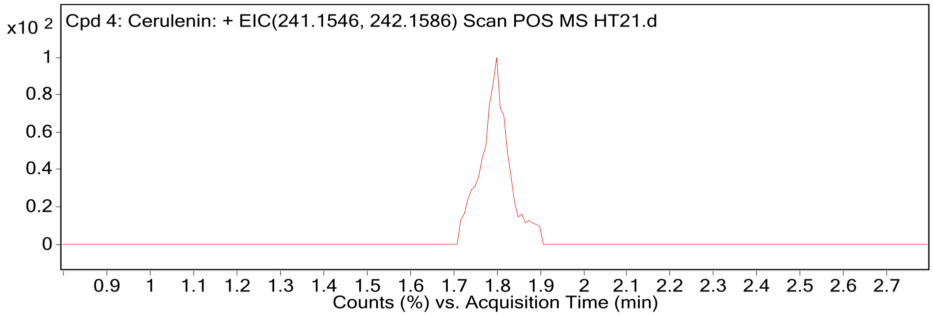

MFE MS Zoomed Spectrum

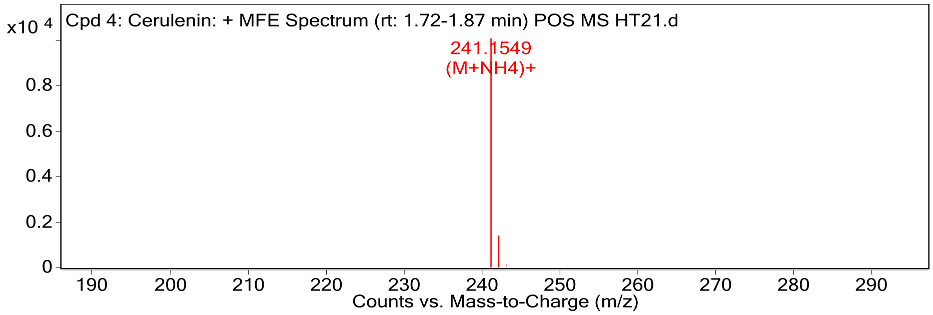

Compound Label	Name	*m/z*	RT	Algorithm	Mass
Cpd 5: Fusarinolic acid	**Fusarinolic acid**	213.1233	1.8	Find by Molecular Feature	195.0895

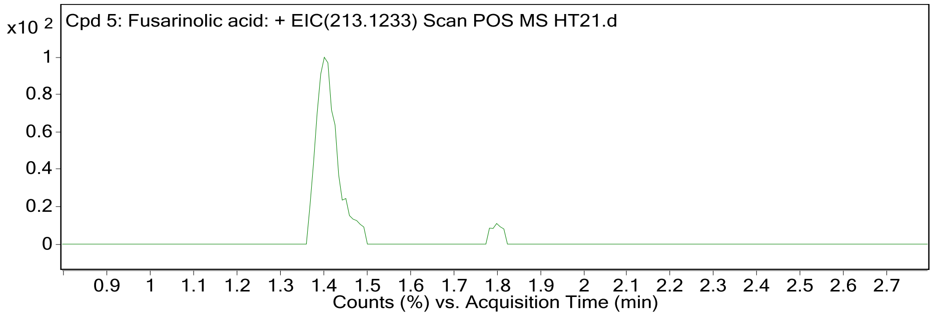

MFE MS Zoomed Spectrum

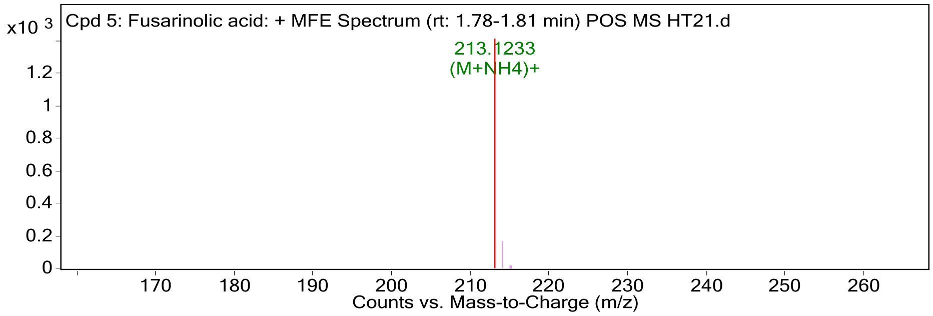

Compound Label	Name	*m/z*	RT	Algorithm	Mass

Cpd 6: Fusarinolic acid	**Fusarinolic acid**	196.0971	1.95	Find by Molecular Feature	195.0883

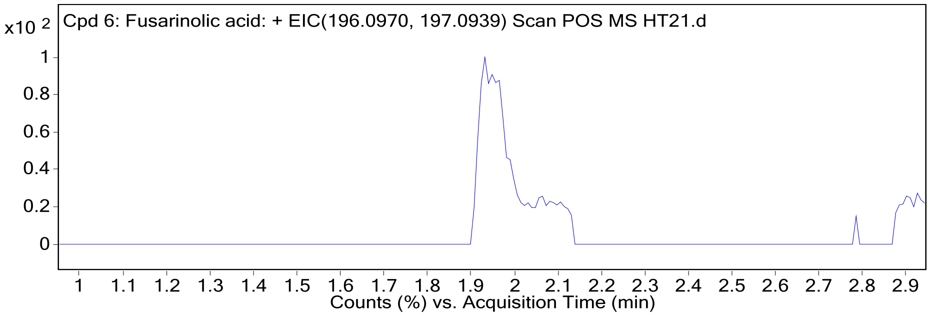

MFE MS Zoomed Spectrum

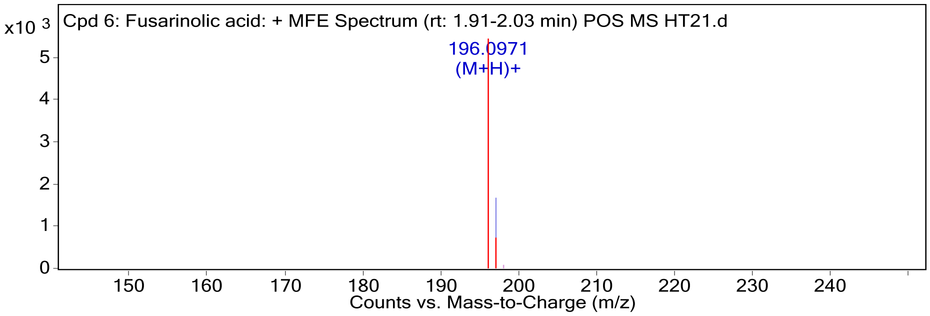

Compound Label	Name	*m/z*	RT	Algorithm	Mass
Cpd 7: 5-Methyl-mellein	**5-Methyl-mellein**	210.1126	1.95	Find by Molecular Feature	192.0788

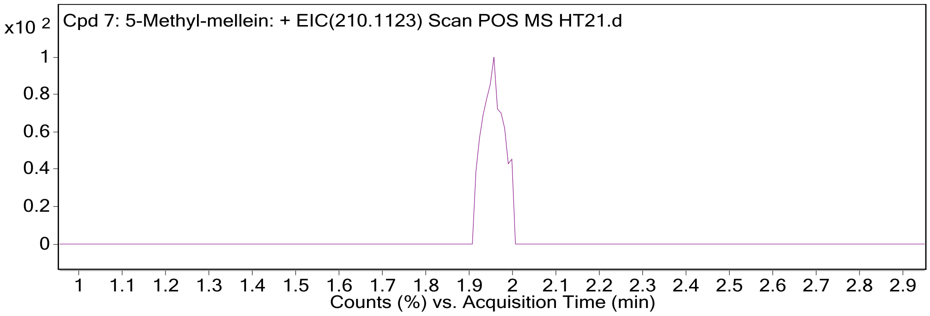

MFE MS Zoomed Spectrum

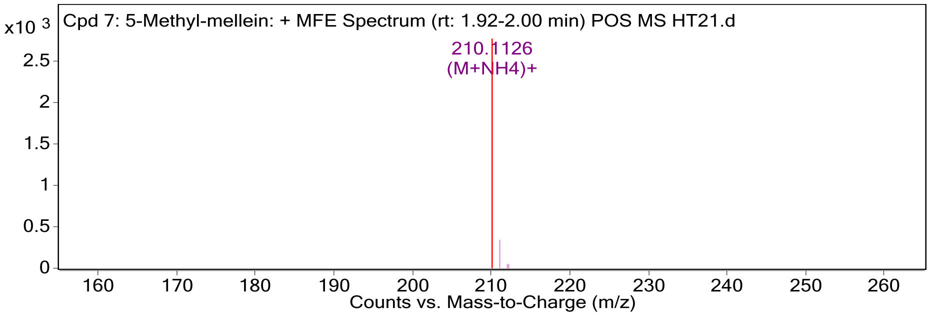

Qualitative Compound Report

Compound Label	Name	*m/z*	RT	Algorithm	Mass
Cpd 8: DON / Deoxynivalenol	**DON / Deoxynivalenol**	314.1602	2	Find by Molecular Feature	296.1263

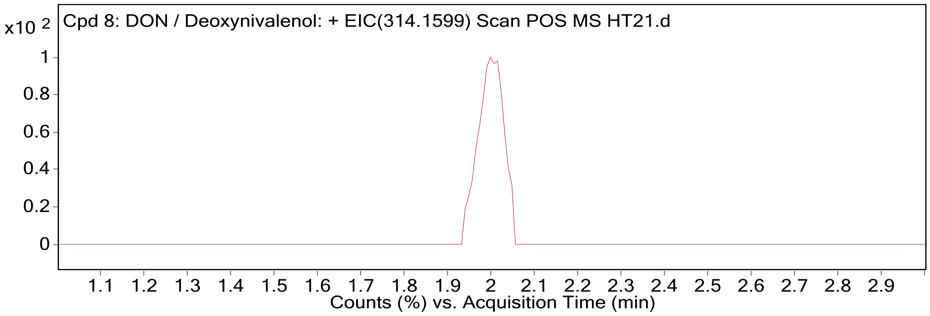

MFE MS Zoomed Spectrum

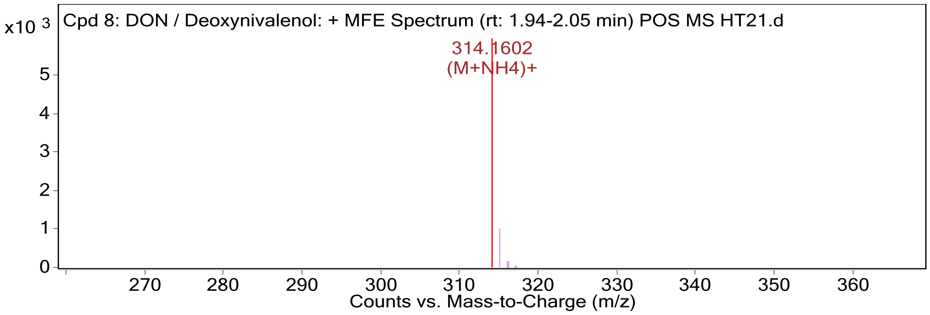

Compound Label	Name	*m/z*	RT	Algorithm	Mass
Cpd 9: Asperlactone	**Asperlactone**	202.1075	2.06	Find by Molecular Feature	184.0737

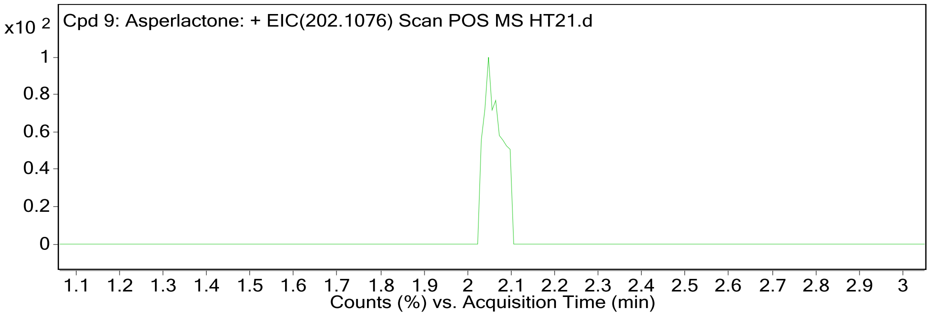

MFE MS Zoomed Spectrum

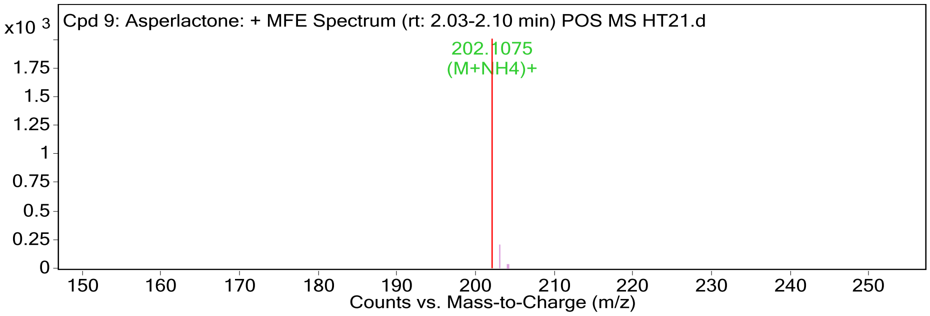

Qualitative Compound Report

Compound Label	Name	*m/z*	RT	Algorithm	Mass
Cpd 10: Asperlactone	**Asperlactone**	185.081	2.07	Find by Molecular Feature	184.0738

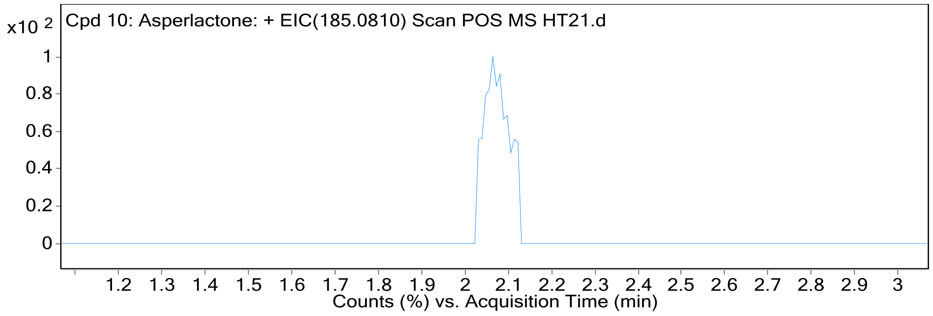

MFE MS Zoomed Spectrum

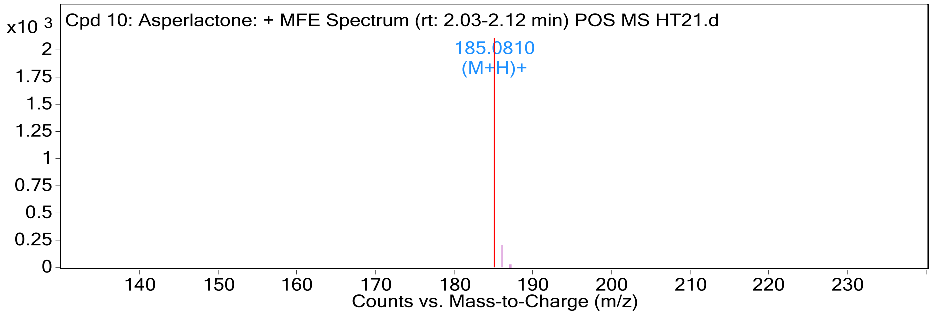

Compound Label	Name	*m/z*	RT	Algorithm	Mass
Cpd 11: 10,11-Dehydrocurvularin	**10,11-Dehydrocurvularin**	308.1494	2.13	Find by Molecular Feature	290.1156

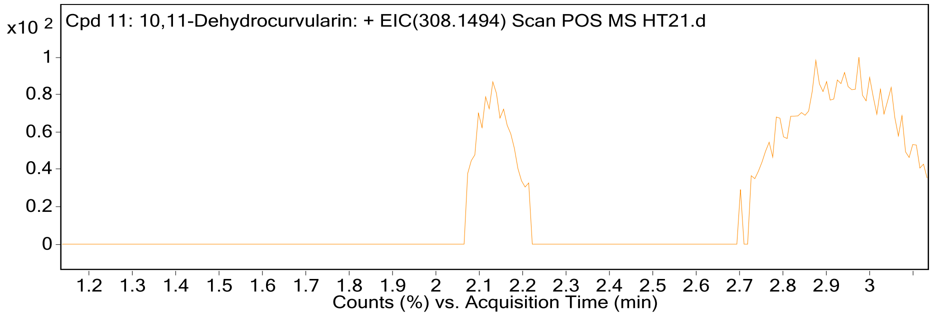

MFE MS Zoomed Spectrum

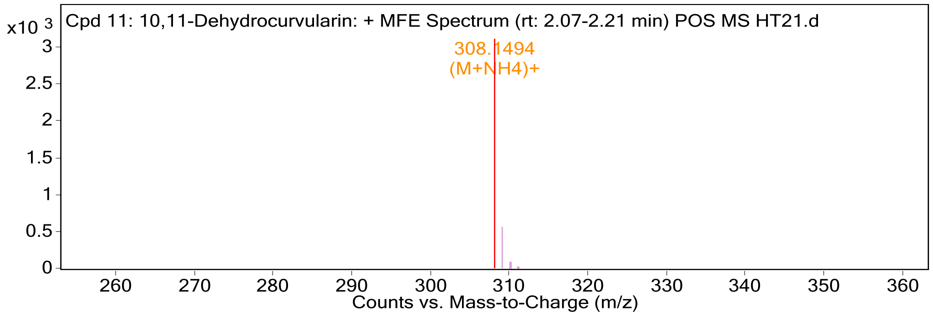

Qualitative Compound Report

Compound Label	Name	*m/z*	RT	Algorithm	Mass
Cpd 12: Austdiol	**Austdiol**	254.1023	2.15	Find by Molecular Feature	236.0685

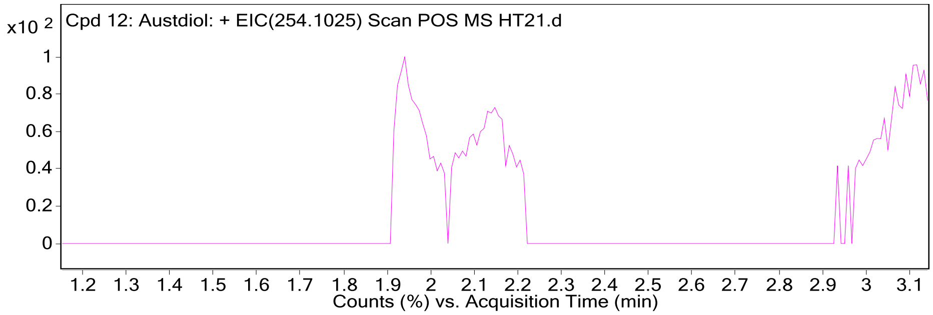

MFE MS Zoomed Spectrum

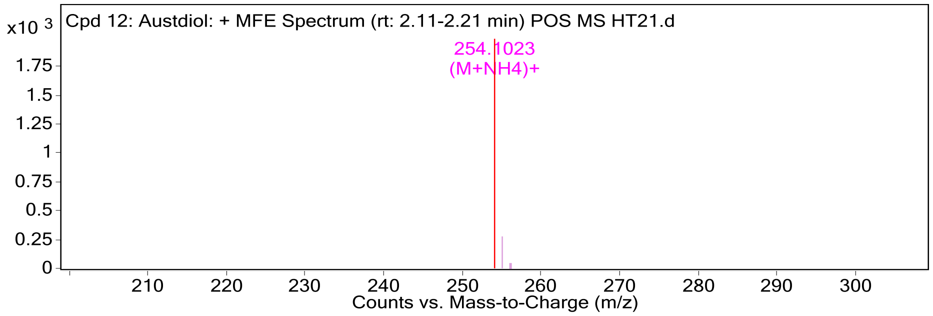

Compound Label	Name	*m/z*	RT	Algorithm	Mass
Cpd 13: [U-13C]-NIV / Nivalenol	**[U-13C]-NIV / Nivalenol**	350.16	2.18	Find by Molecular Feature	327.1707

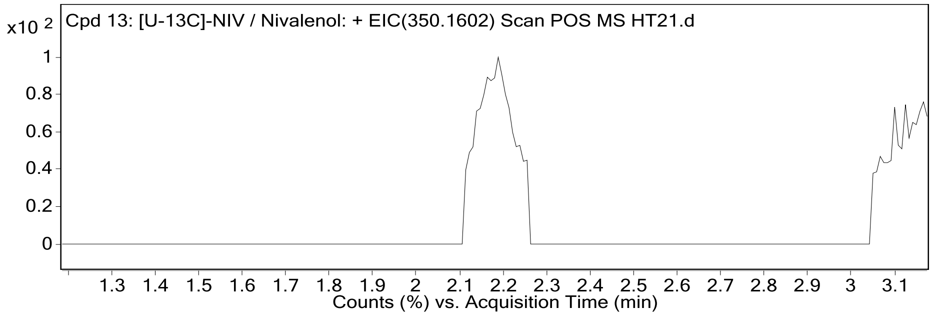

MFE MS Zoomed Spectrum

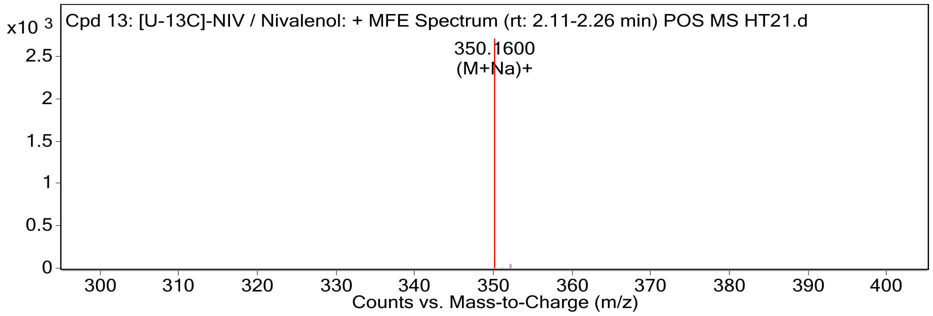

Compound Label	Name	*m/z*	RT	Algorithm	Mass
Cpd 14: Dechlorogriseofulvin	**Dechlorogriseofulvin**	336.1442	2.19	Find by Molecular Feature	318.1104

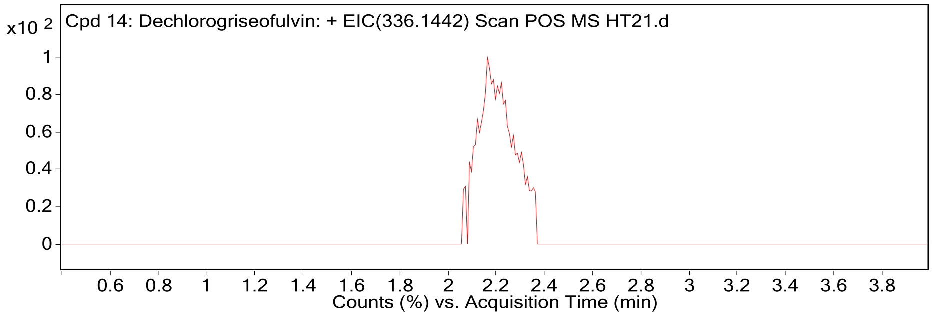

MFE MS Zoomed Spectrum

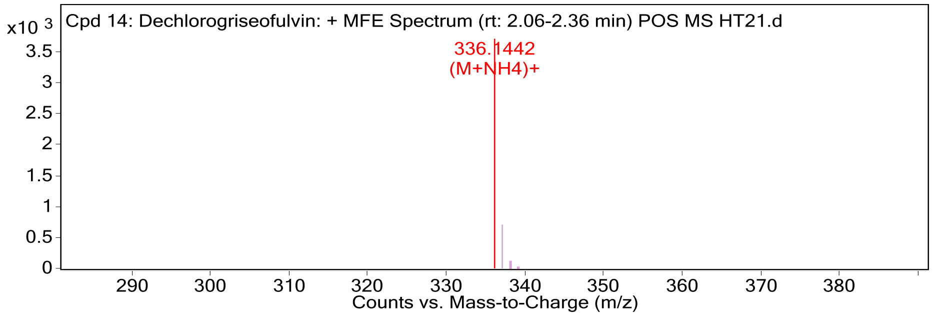

Compound Label	Name	*m/z*	RT	Algorithm	Mass
Cpd 15: Decarestrictine D	**Decarestrictine D**	234.1337	2.33	Find by Molecular Feature	216.0999

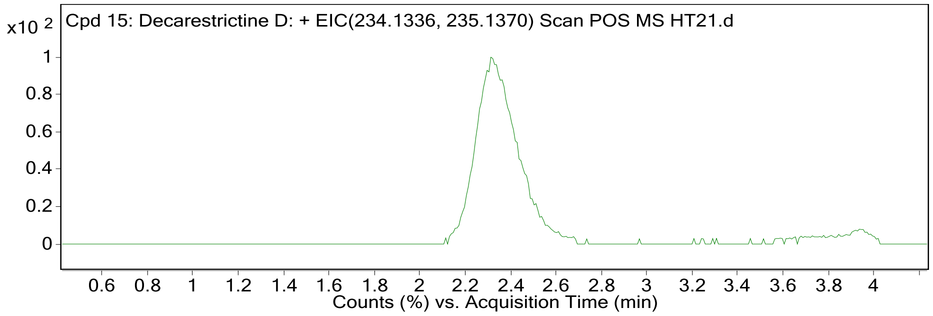

MFE MS Zoomed Spectrum

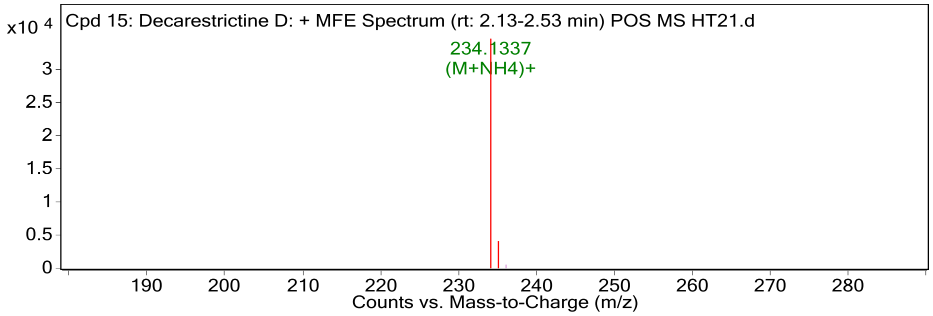

Compound Label	Name	*m/z*	RT	Algorithm	Mass
Cpd 16: Tenuazonic acid	**Tenuazonic acid**	198.1125	2.34	Find by Molecular Feature	197.1052

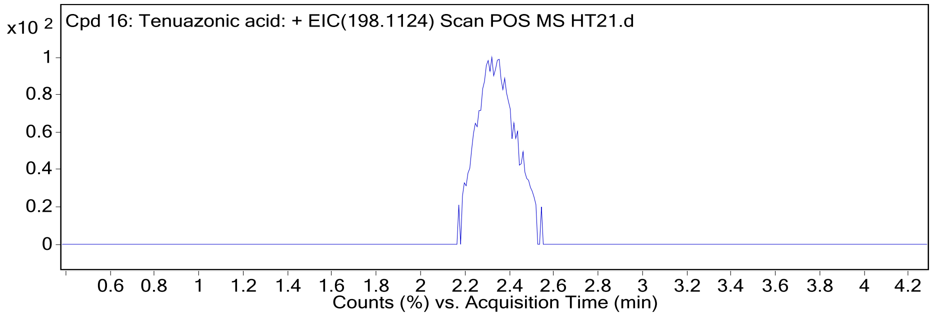

MFE MS Zoomed Spectrum

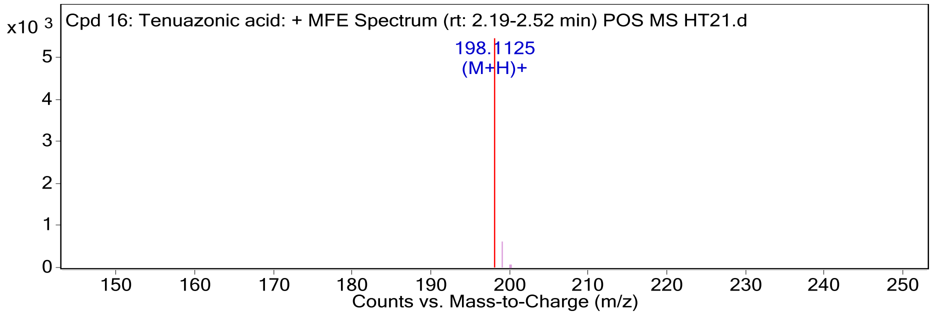

Compound Label	Name	*m/z*	RT	Algorithm	Mass
Cpd 17: 10,11-Dehydrocurvularin	**10,11-Dehydrocurvularin**	308.1493	2.94	Find by Molecular Feature	290.1155

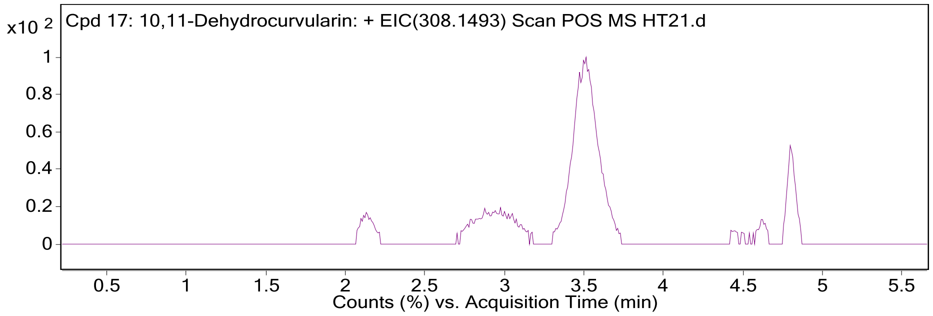

MFE MS Zoomed Spectrum

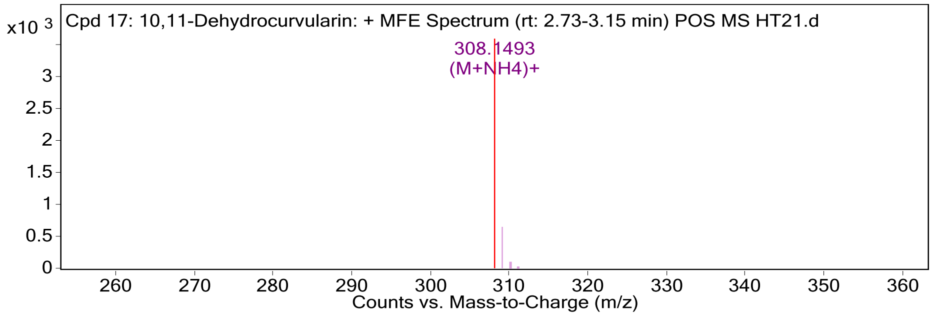

Compound Label	Name	*m/z*	RT	Algorithm	Mass

Cpd 18: Pyrenocine A	**Pyrenocine A**	226.1073	3.03	Find by Molecular Feature	208.0735

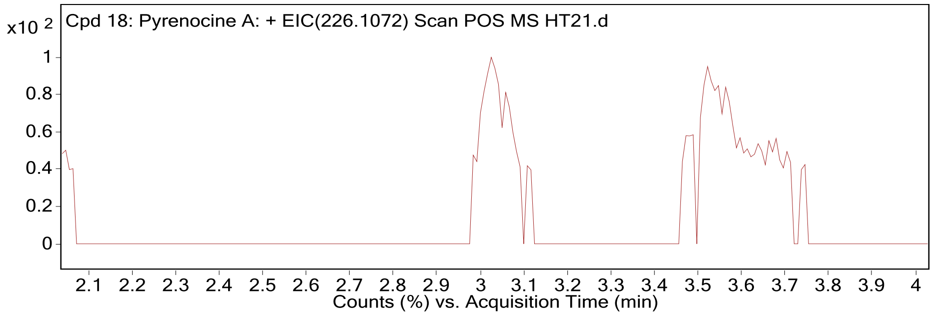

MFE MS Zoomed Spectrum

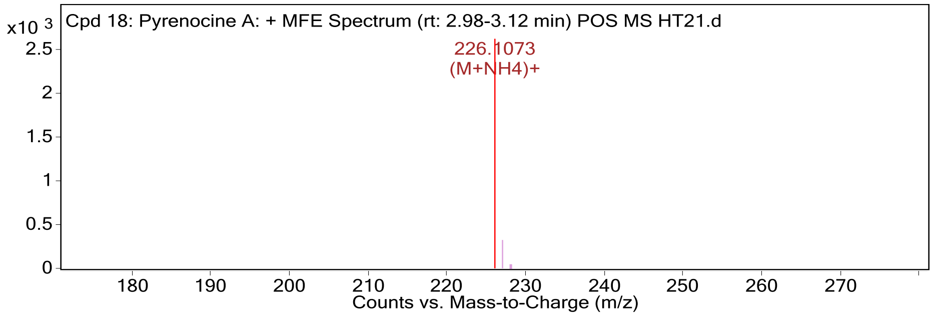

Compound Label	Name	*m/z*	RT	Algorithm	Mass
Cpd 19: Terrein	**Terrein**	172.0969	3.04	Find by Molecular Feature	154.0631

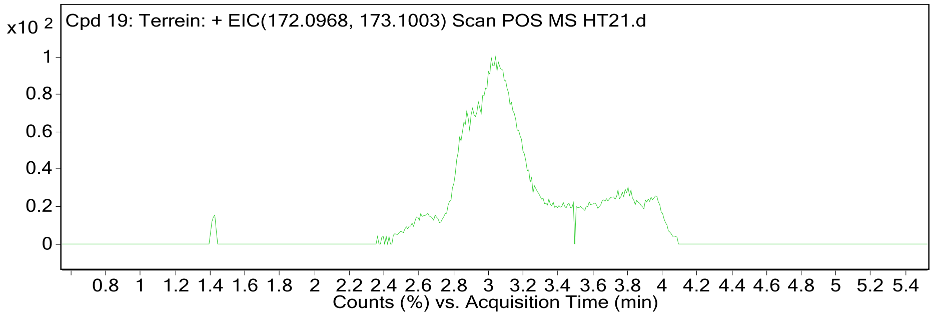

MFE MS Zoomed Spectrum

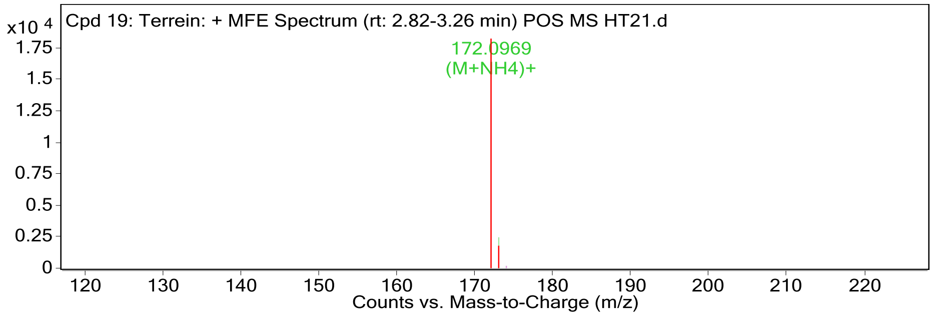

**Compound Label**	**Name**	***m/z***	**RT**	**Algorithm**	**Mass**
Cpd 20: Austdiol	**Austdiol**	254.1023	3.13	Find by Molecular Feature	236.0685

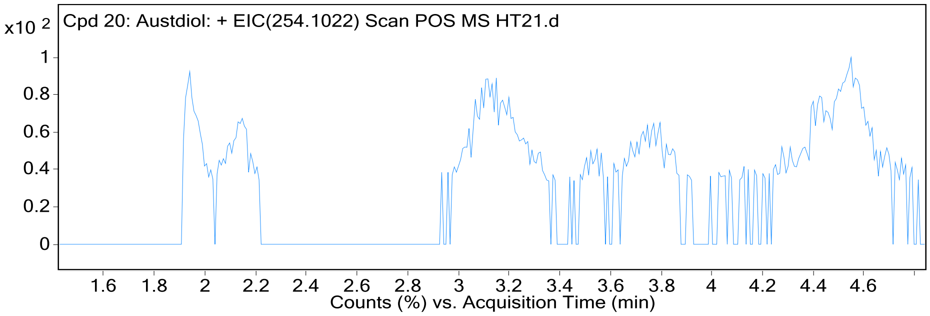

MFE MS Zoomed Spectrum

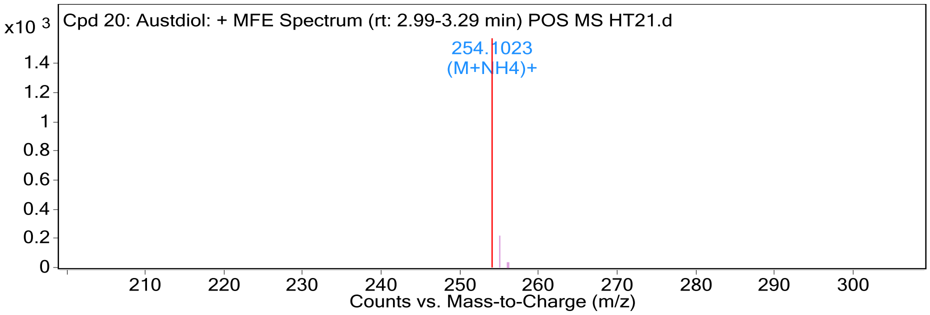

**Compound Label**	**Name**	***m/z***	**RT**	**Algorithm**	**Mass**
Cpd 21: [U-13C]-NIV / Nivalenol	**[U-13C]-NIV / Nivalenol**	350.1598	3.17	Find by Molecular Feature	327.1706

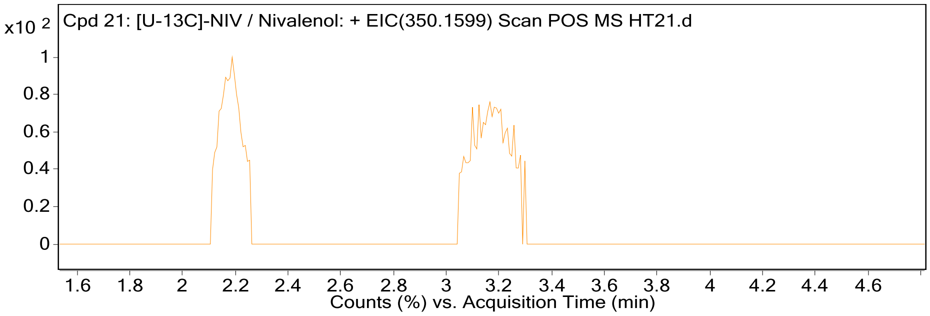

MFE MS Zoomed Spectrum

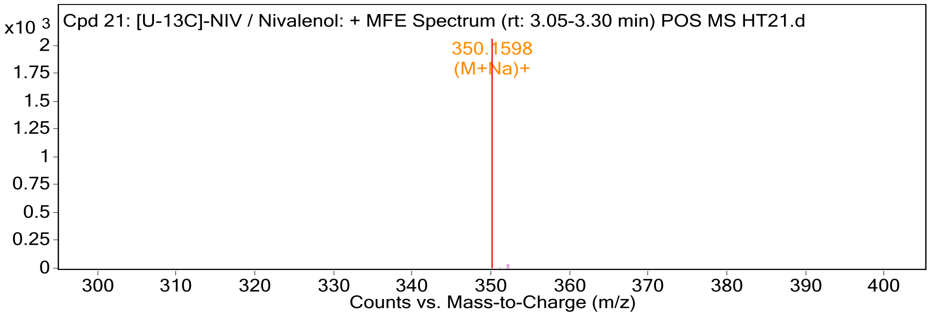

Compound Label	Name	*m/z*	RT	Algorithm	Mass
Cpd 22: Antimycin	**Antimycin**	268.1178	3.27	Find by Molecular Feature	250.0839

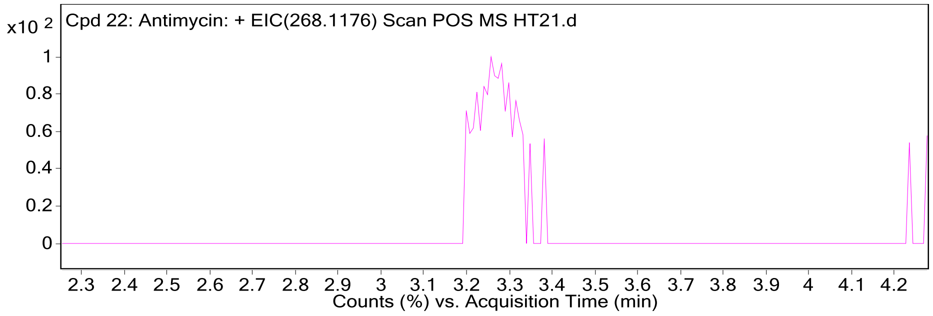

MFE MS Zoomed Spectrum

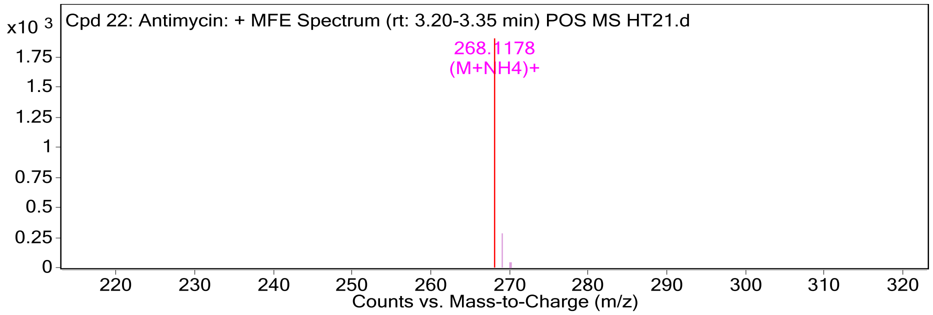

Compound Label	Name	*m/z*	RT	Algorithm	Mass
Cpd 23: 10,11-Dehydrocurvularin	**10,11-Dehydrocurvularin**	308.1495	3.51	Find by Molecular Feature	290.1156

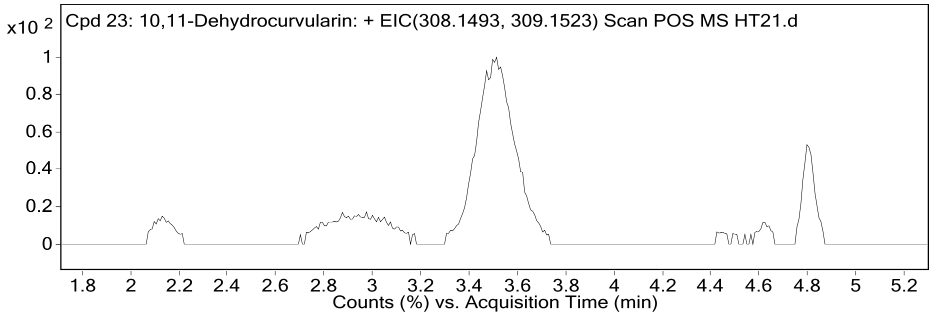

MFE MS Zoomed Spectrum

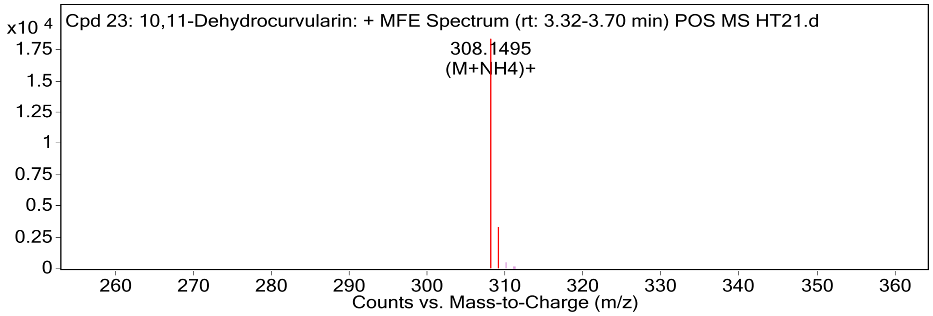

Compound Label	Name	*m/z*	RT	Algorithm	Mass
Cpd 24: Rubrofusarin	**Rubrofusarin**	290.1024	3.51	Find by Molecular Feature	272.0686

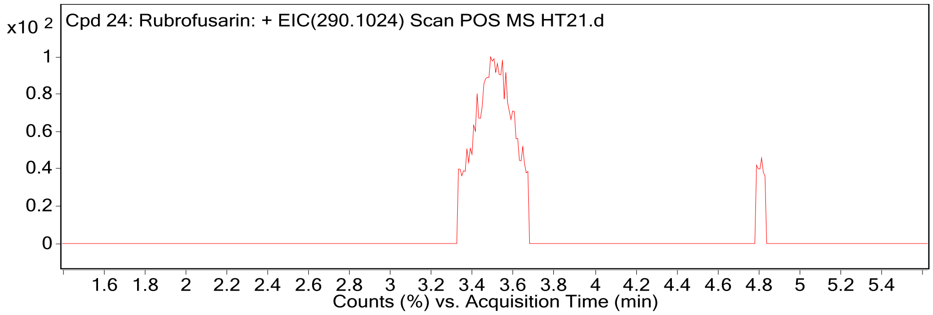

MFE MS Zoomed Spectrum

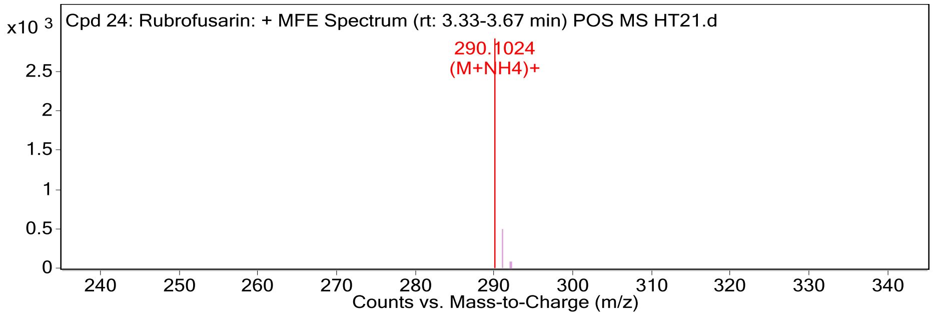

Compound Label	Name	*m/z*	RT	Algorithm	Mass
Cpd 25: Pyrenocine A	**Pyrenocine A**	226.1072	3.54	Find by Molecular Feature	208.0734

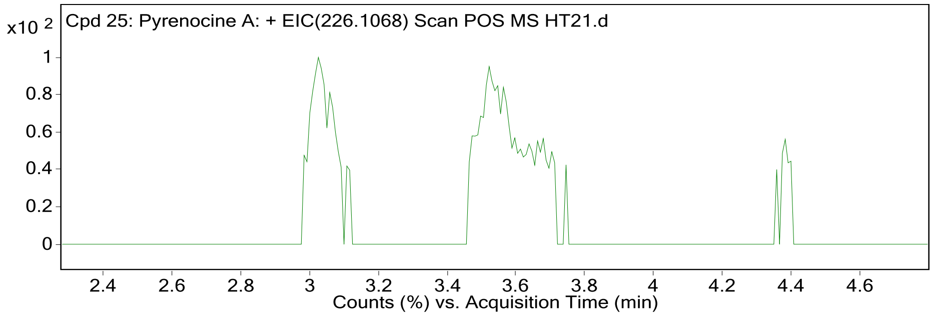

MFE MS Zoomed Spectrum

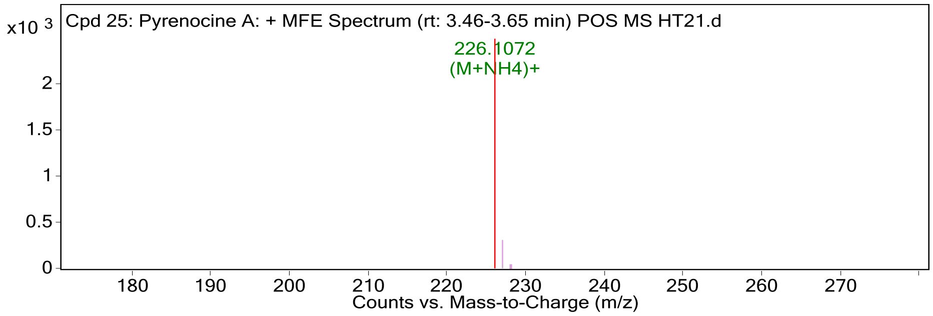

Compound Label	Name	*m/z*	RT	Algorithm	Mass
Cpd 26: Decarestrictine D	**Decarestrictine D**	234.1334	3.58	Find by Molecular Feature	216.0996

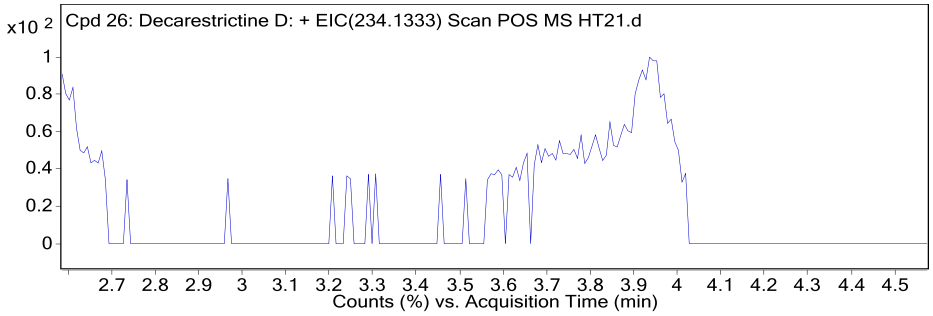

MFE MS Zoomed Spectrum

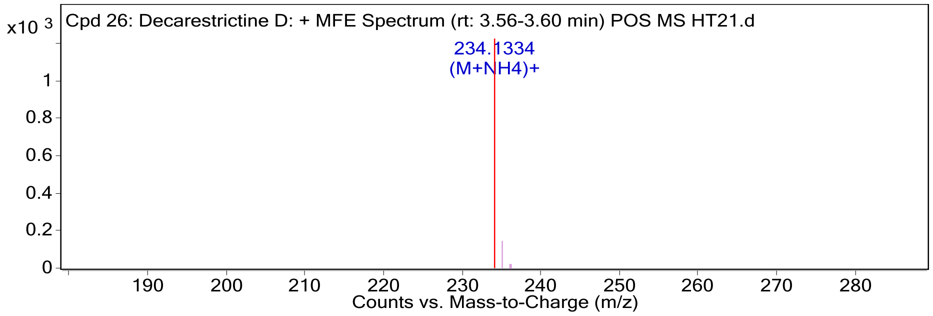

Compound Label	Name	*m/z*	RT	Algorithm	Mass
Cpd 27: Decarestrictine D	**Decarestrictine D**	234.1339	3.75	Find by Molecular Feature	216.1

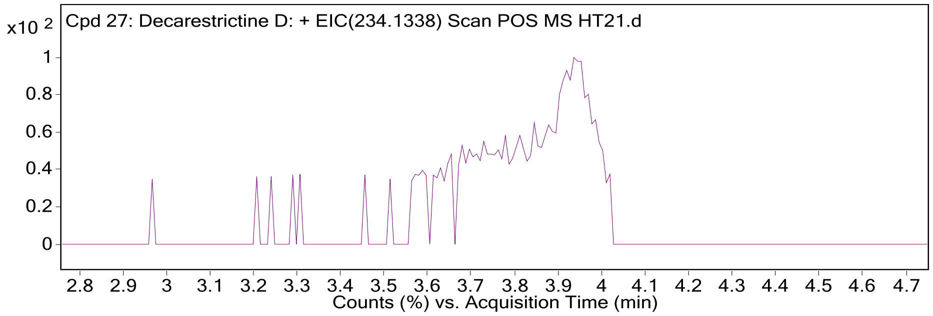

MFE MS Zoomed Spectrum

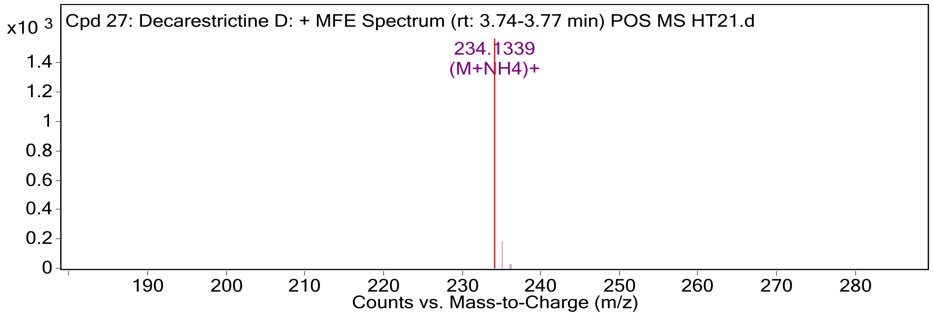

Compound Label	Name	*m/z*	RT	Algorithm	Mass
Cpd 28: Austdiol	**Austdiol**	254.1023	3.76	Find by Molecular Feature	236.0685

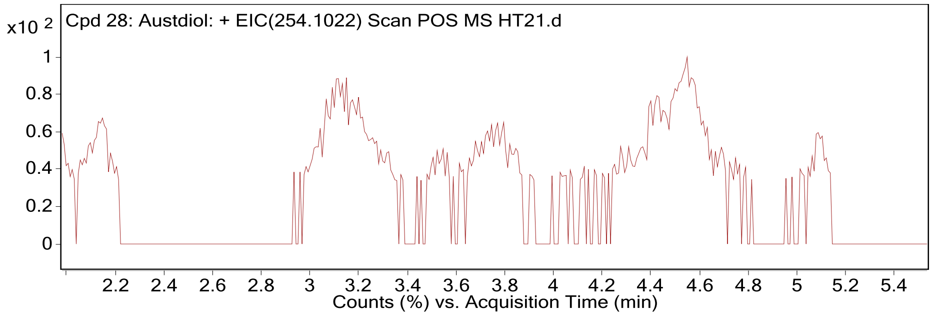

MFE MS Zoomed Spectrum

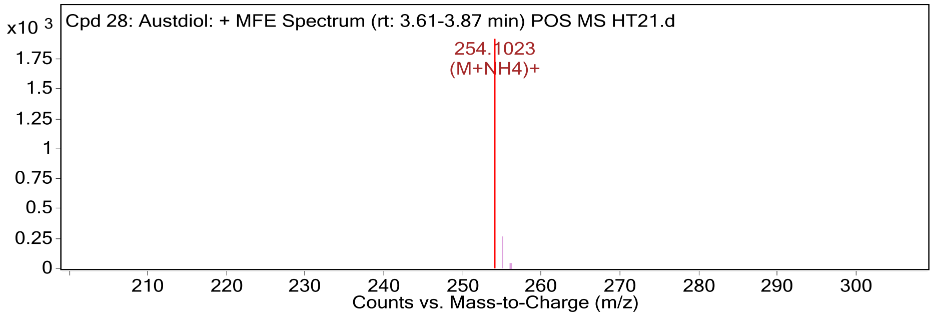

Compound Label	Name	*m/z*	RT	Algorithm	Mass
Cpd 29: Decarestrictine D	**Decarestrictine D**	234.1337	3.93	Find by Molecular Feature	216.0998

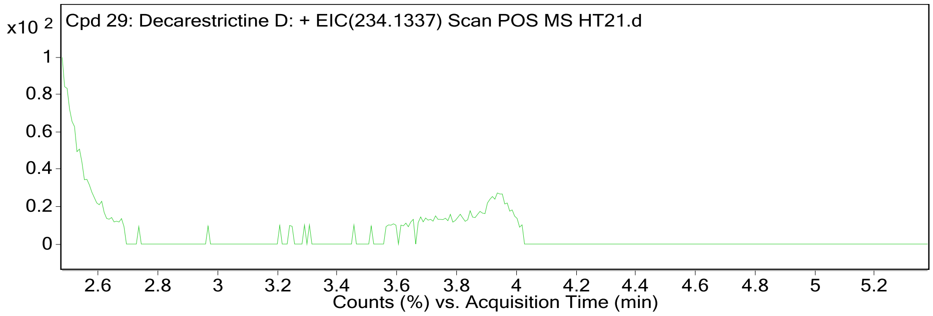

MFE MS Zoomed Spectrum

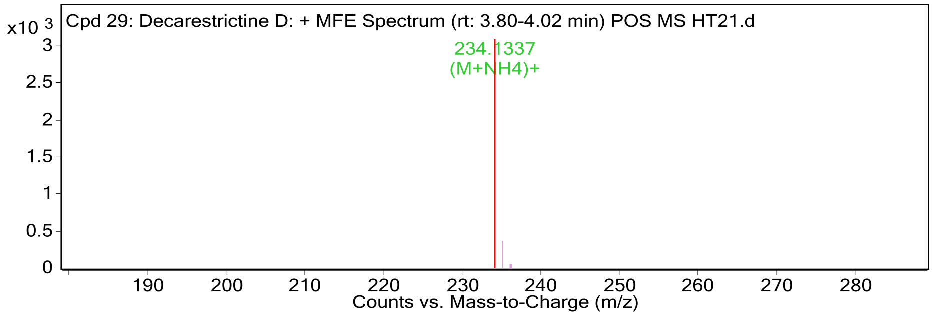

Compound Label	Name	*m/z*	RT	Algorithm	Mass
Cpd 30: Terrein	**Terrein**	172.097	3.96	Find by Molecular Feature	154.0632

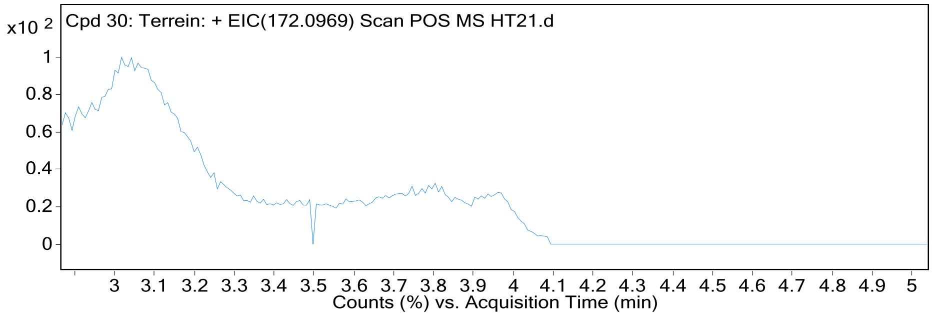

MFE MS Zoomed Spectrum

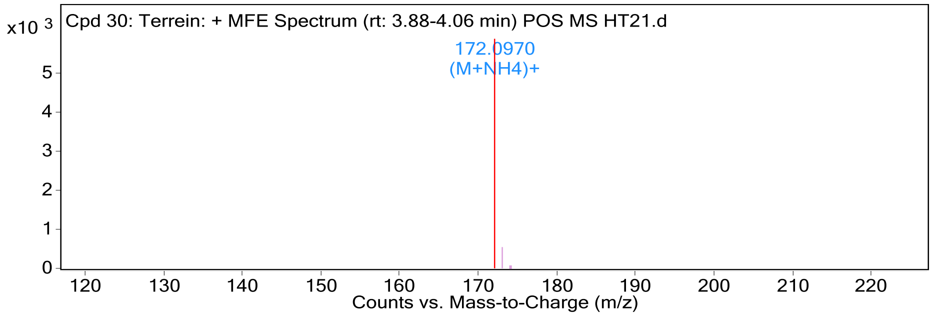

Compound Label	Name	*m/z*	RT	Algorithm	Mass
Cpd 31: Asterric acid	**Asterric acid**	366.1184	4.15	Find by Molecular Feature	348.0846

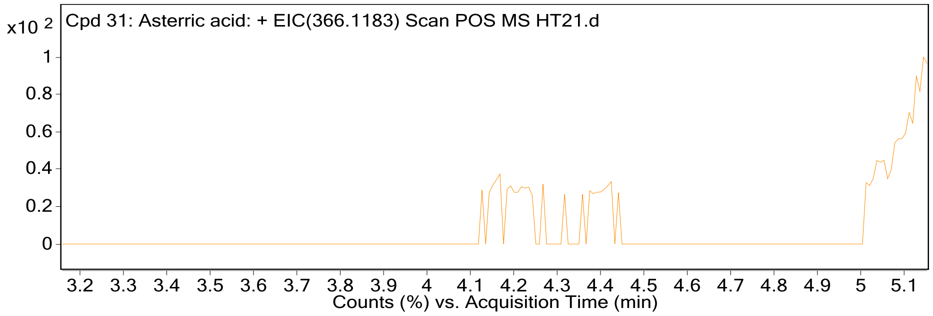

MFE MS Zoomed Spectrum

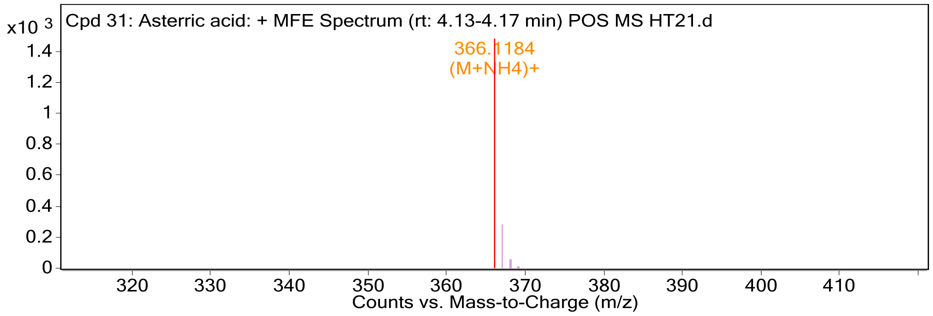

Compound Label	Name	*m/z*	RT	Algorithm	Mass
Cpd 32: Pyrenocine A	**Pyrenocine A**	226.1076	4.38	Find by Molecular Feature	208.0738

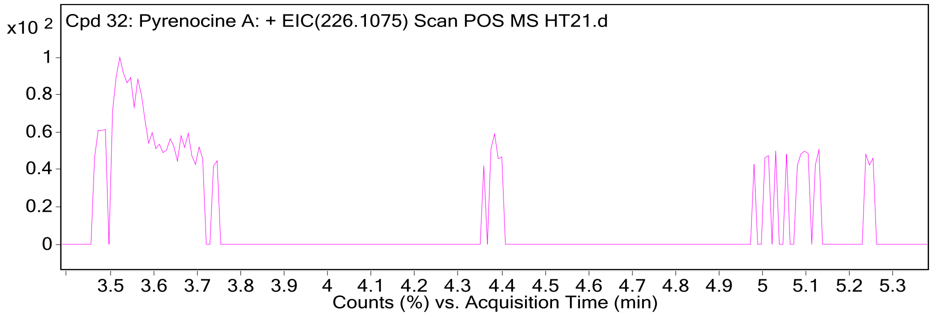

MFE MS Zoomed Spectrum

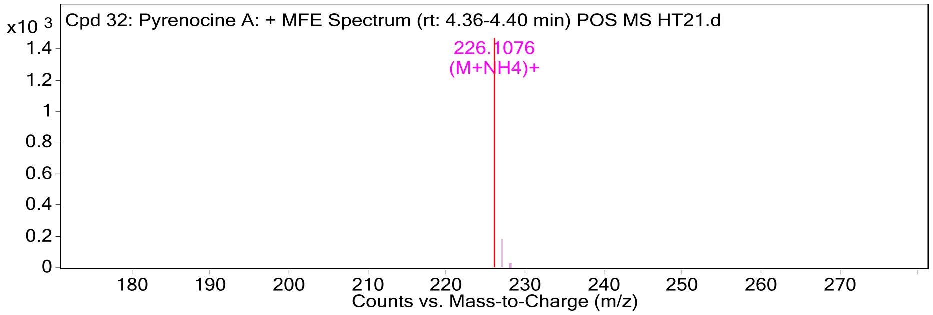

**Compound Label**	**Name**	***m/z***	**RT**	**Algorithm**	**Mass**
Cpd 33: Fusaric acid	**Fusaric acid**	197.1285	4.52	Find by Molecular Feature	179.0947

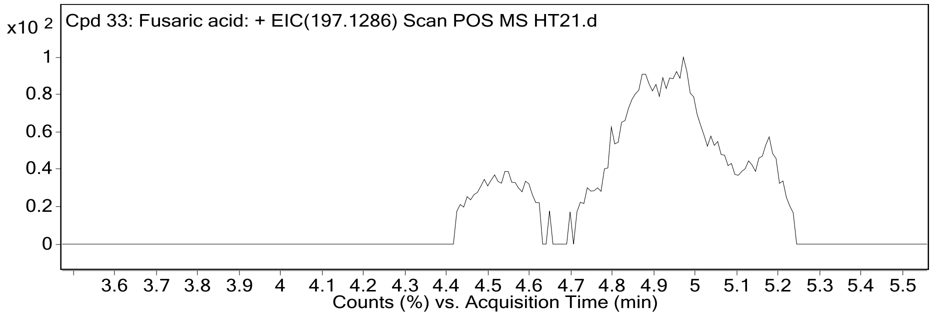

MFE MS Zoomed Spectrum

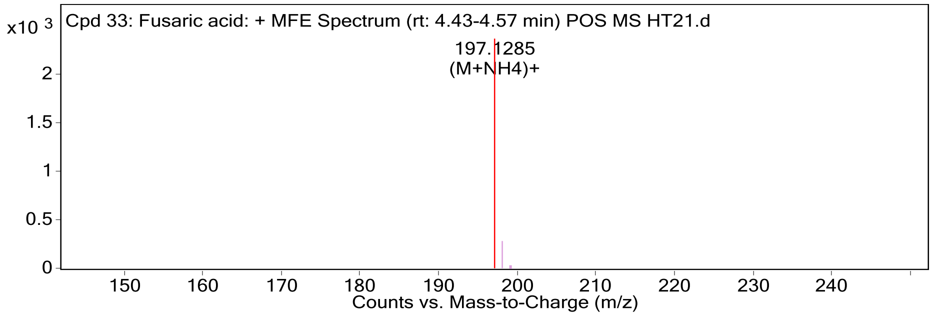

**Compound Label**	**Name**	***m/z***	**RT**	**Algorithm**	**Mass**

Cpd 34: Austdiol	**Austdiol**	254.1024	4.54	Find by Molecular Feature	236.0686

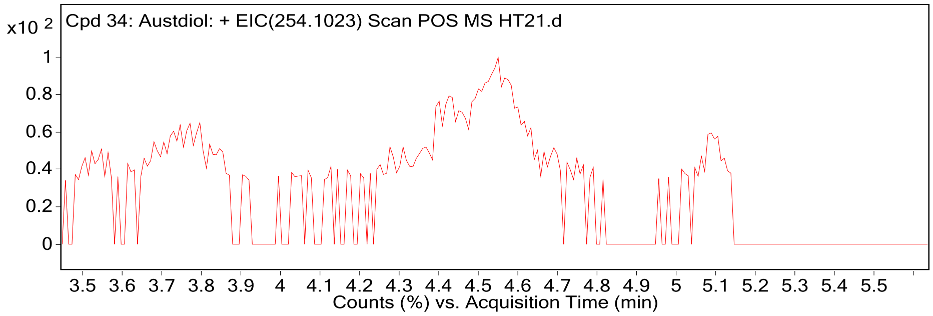

MFE MS Zoomed Spectrum

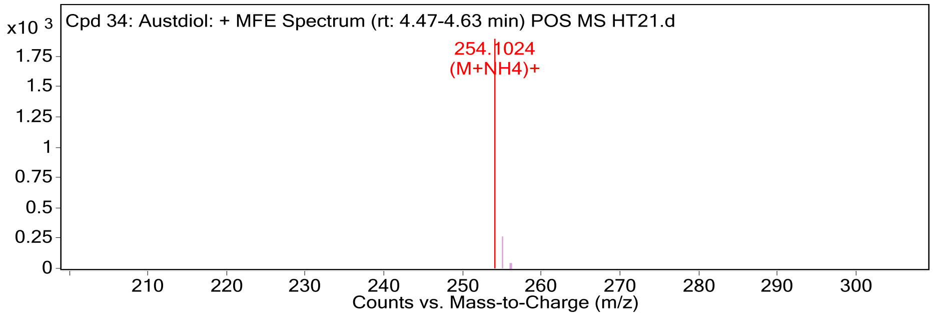

**Compound Label**	**Name**	***m/z***	**RT**	**Algorithm**	**Mass**
Cpd 35: Fusaric acid	**Fusaric acid**	197.1284	4.59	Find by Molecular Feature	179.0946

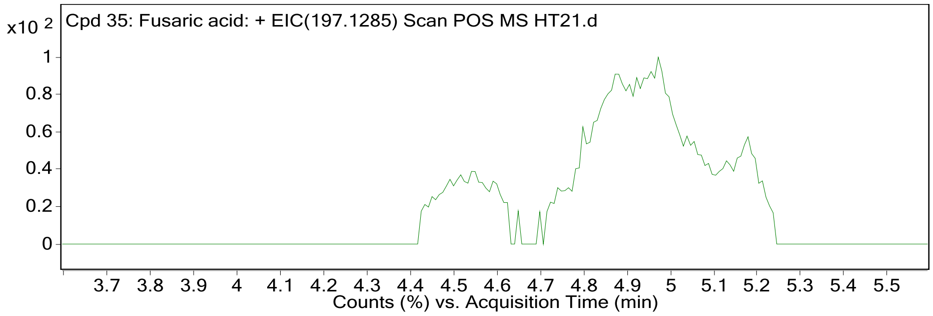

MFE MS Zoomed Spectrum

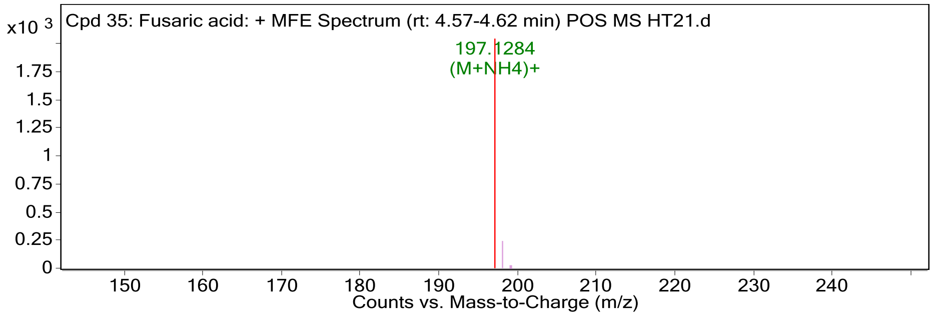

Compound Label	Name	*m/z*	RT	Algorithm	Mass
Cpd 36: 10,11-Dehydrocurvularin	**10,11-Dehydrocurvularin**	308.1493	4.62	Find by Molecular Feature	290.1155

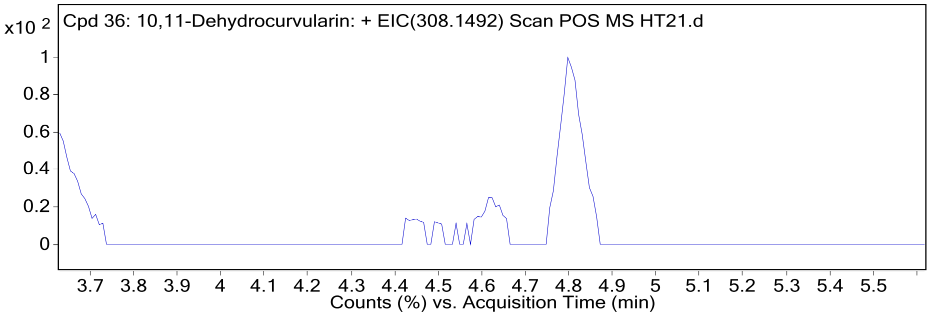

MFE MS Zoomed Spectrum

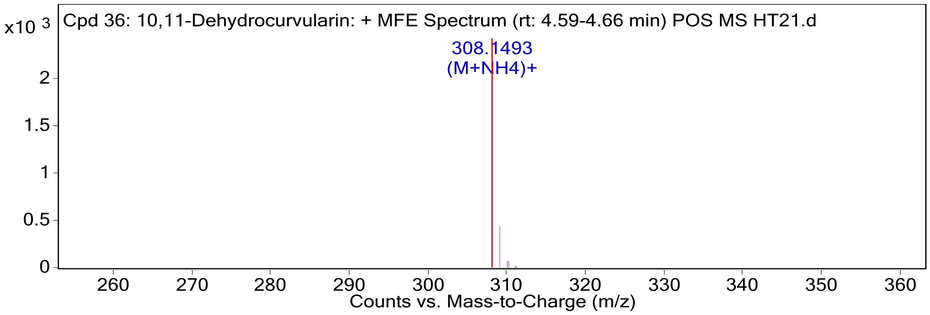

Compound Label	Name	*m/z*	RT	Algorithm	Mass
Cpd 37: 10,11-Dehydrocurvularin	**10,11-Dehydrocurvularin**	308.1495	4.8	Find by Molecular Feature	290.1156

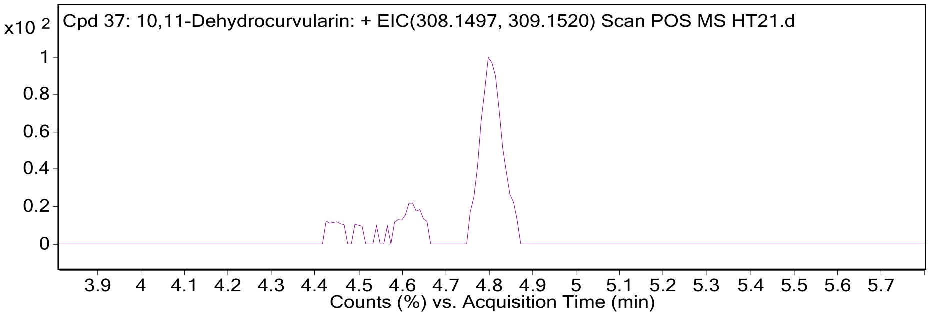

MFE MS Zoomed Spectrum

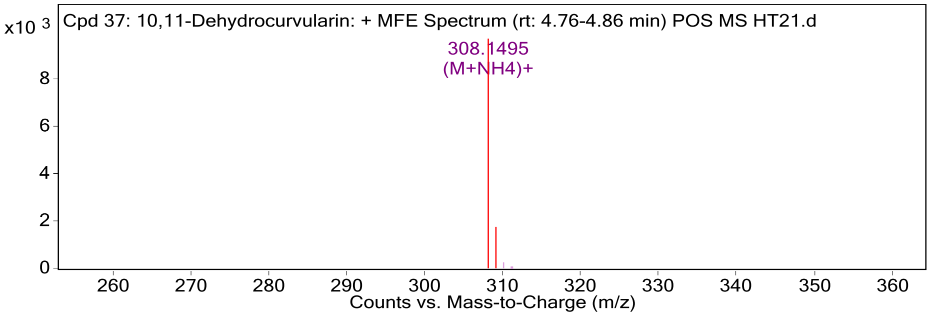

Compound Label	Name	*m/z*	RT	Algorithm	Mass
Cpd 38: Fusaric acid	**Fusaric acid**	197.1286	4.91	Find by Molecular Feature	179.0947

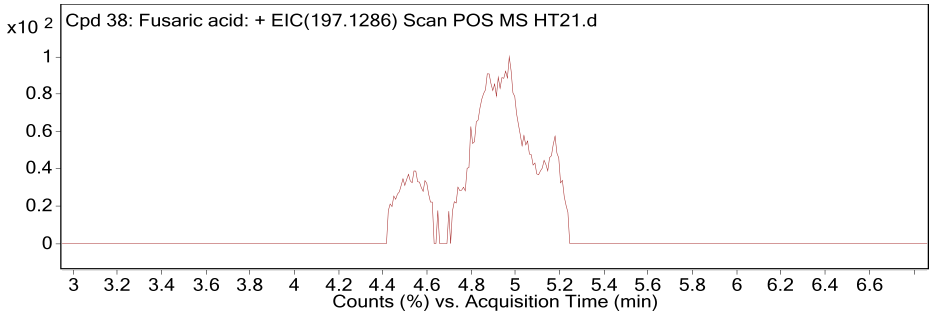

MFE MS Zoomed Spectrum

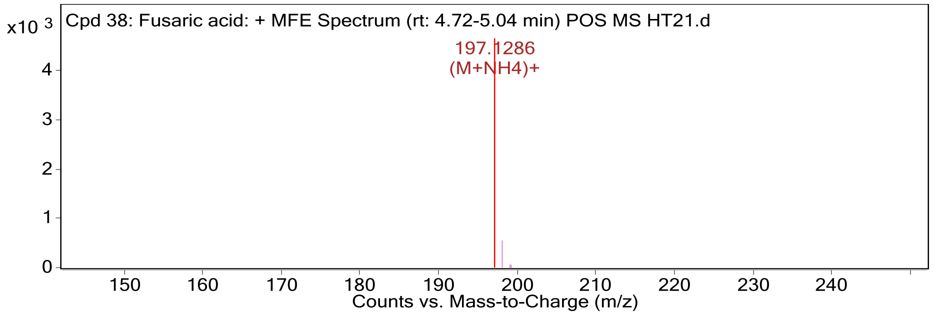

Compound Label	Name	*m/z*	RT	Algorithm	Mass
Cpd 39: Austdiol	**Austdiol**	254.1022	5.09	Find by Molecular Feature	236.0684

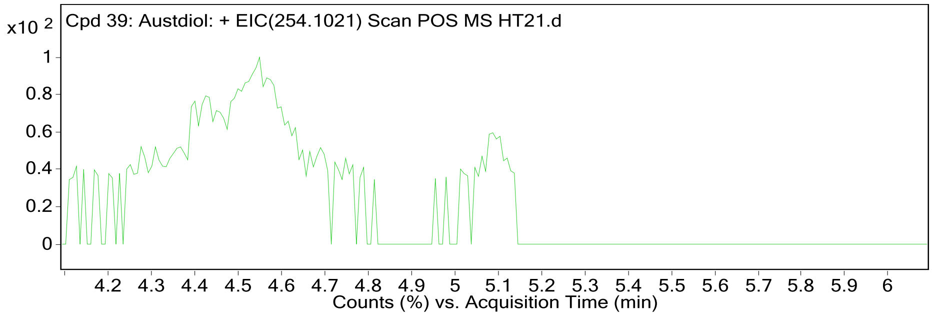

MFE MS Zoomed Spectrum

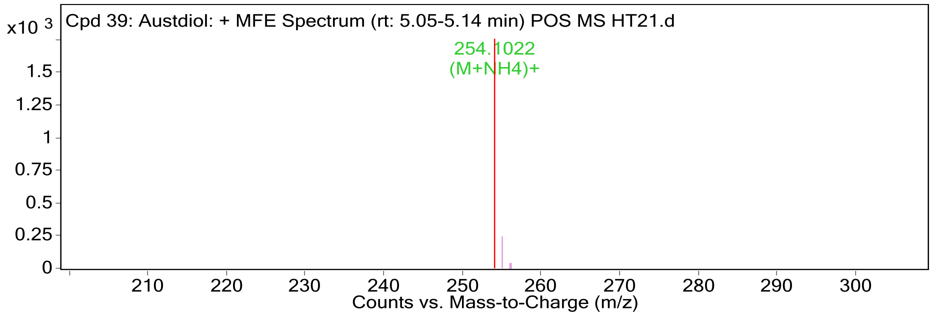

Compound Label	Name	*m/z*	RT	Algorithm	Mass
Cpd 40: Pyrenocine A	**Pyrenocine A**	226.1074	5.09	Find by Molecular Feature	208.0736

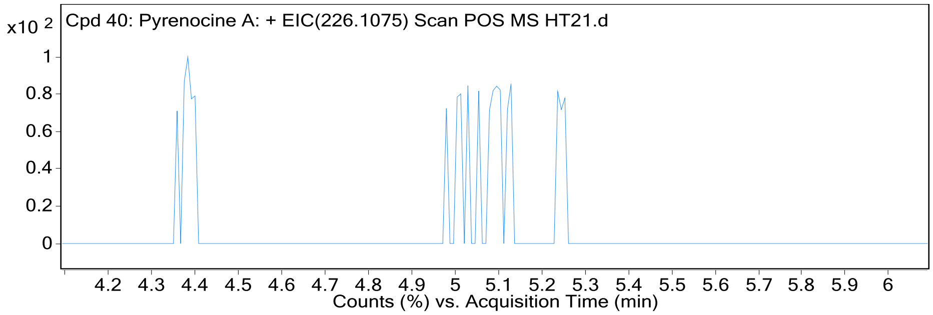

MFE MS Zoomed Spectrum

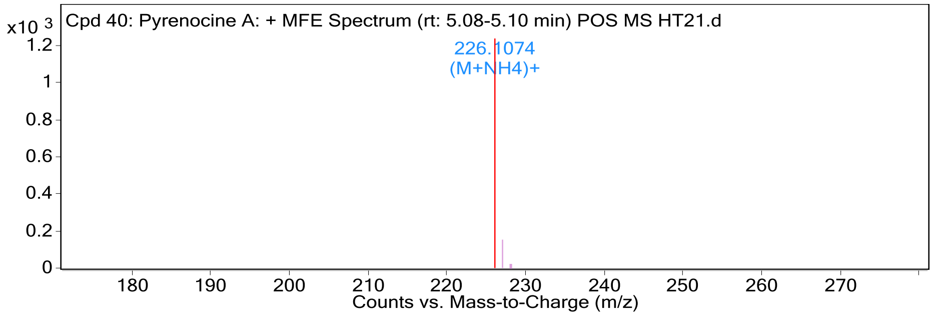

Compound Label	Name	*m/z*	RT	Algorithm	Mass
Cpd 41: Oosporein	**Oosporein**	307.0452	5.13	Find by Molecular Feature	306.0379

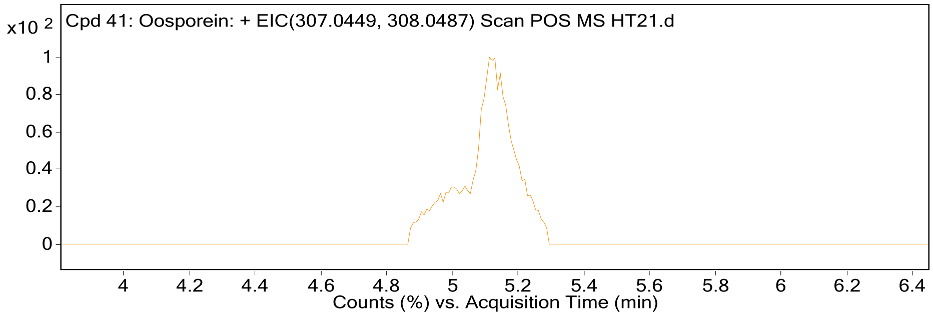

MFE MS Zoomed Spectrum

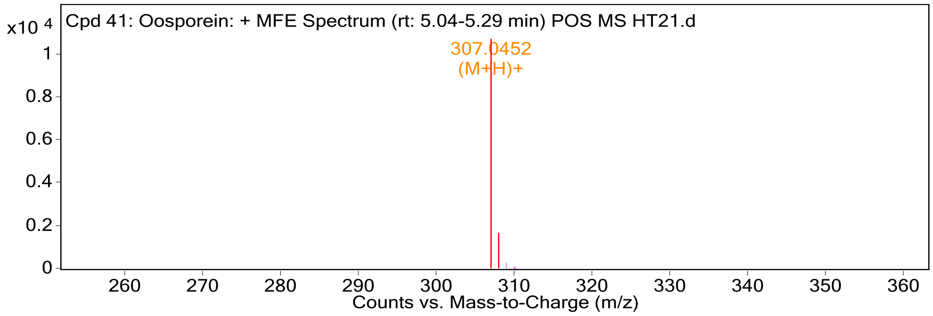

Compound Label	Name	*m/z*	RT	Algorithm	Mass
Cpd 42: Asterric acid	**Asterric acid**	366.1187	5.14	Find by Molecular Feature	348.0849

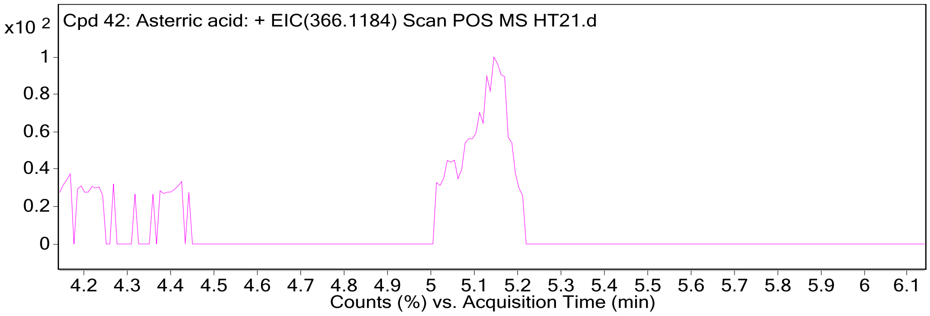

MFE MS Zoomed Spectrum

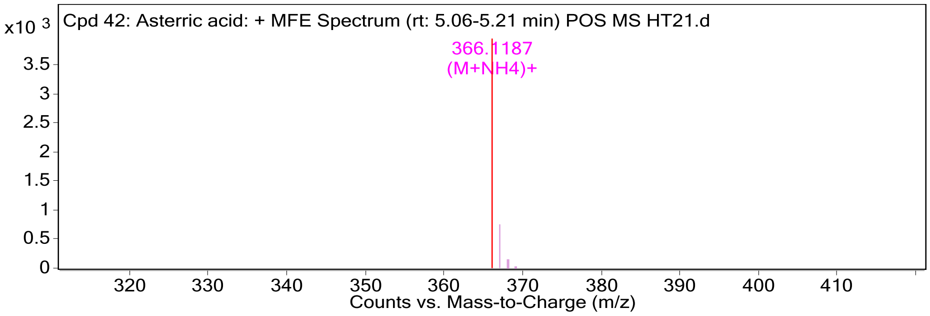

Compound Label	Name	*m/z*	RT	Algorithm	Mass
Cpd 43: Fusaric acid	**Fusaric acid**	197.1287	5.17	Find by Molecular Feature	179.0949

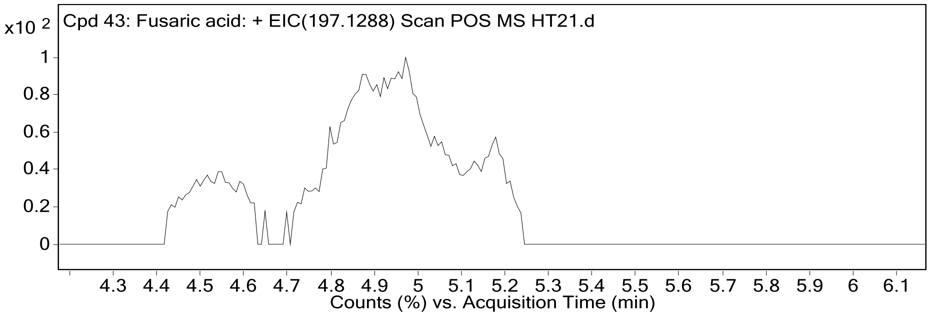

MFE MS Zoomed Spectrum

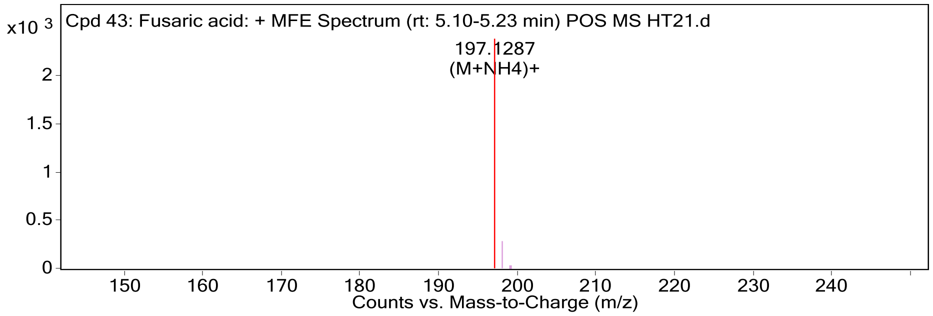

Compound Label	Name	*m/z*	RT	Algorithm	Mass
Cpd 44: Tenuazonic acid	**Tenuazonic acid**	198.1125	5.29	Find by Molecular Feature	197.1052

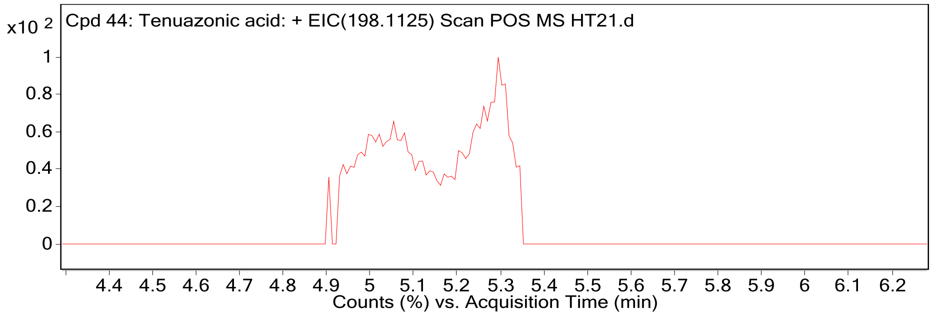

MFE MS Zoomed Spectrum

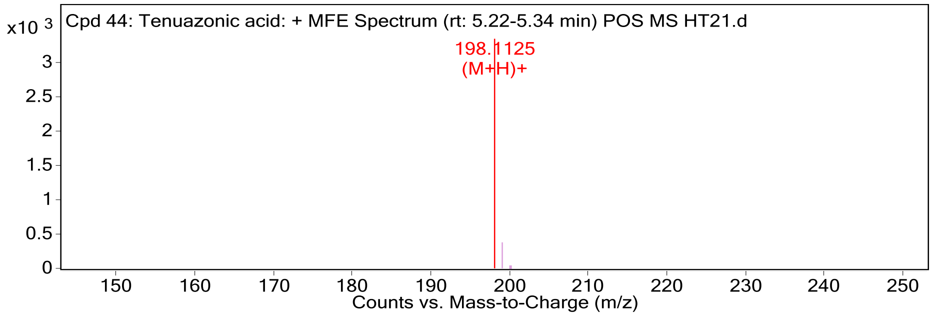

Compound Label	Name	*m/z*	RT	Algorithm	Mass
Cpd 45: Cerulenin	**Cerulenin**	241.1547	5.31	Find by Molecular Feature	223.1209

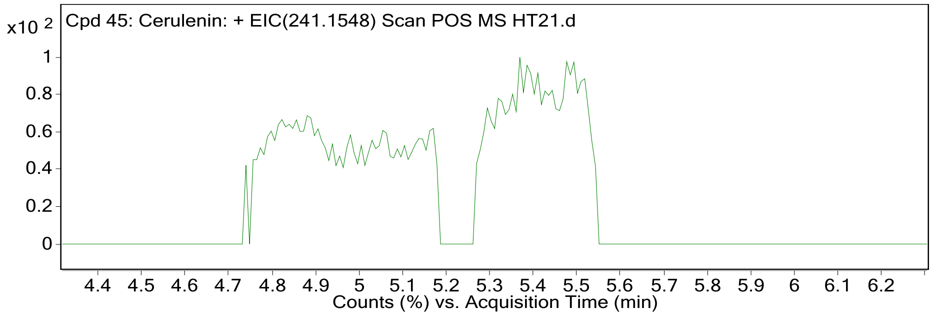

MFE MS Zoomed Spectrum

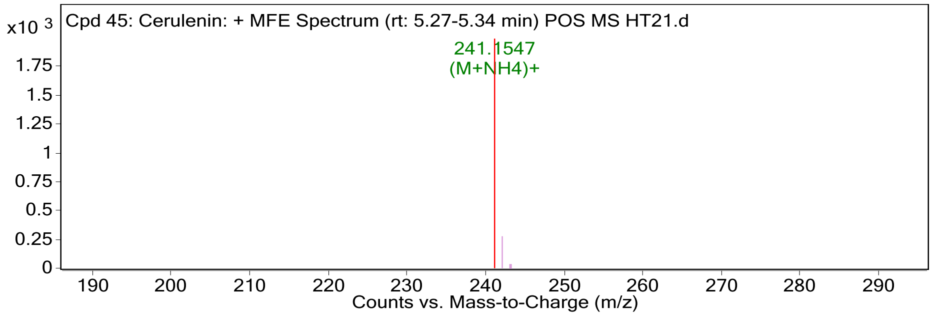

Compound Label	Name	*m/z*	RT	Algorithm	Mass
Cpd 46: Infectopyrone	**Infectopyrone**	282.1335	5.56	Find by Molecular Feature	264.0997

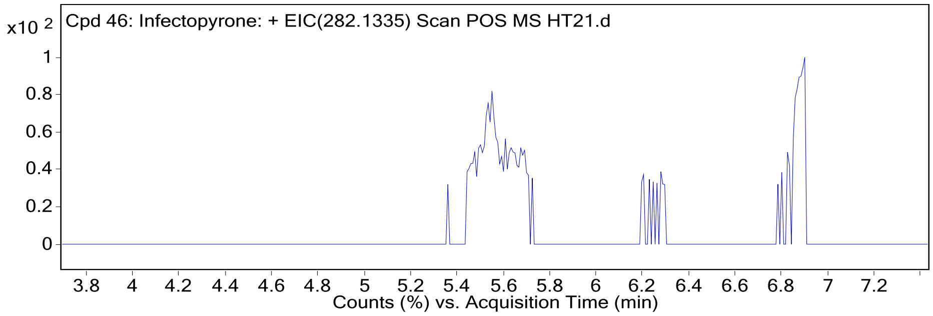

MFE MS Zoomed Spectrum

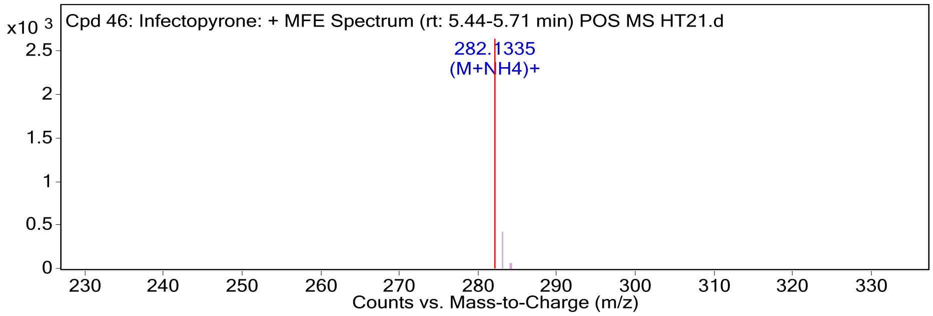

Compound Label	Name	*m/z*	RT	Algorithm	Mass
Cpd 47: Pyrenocine A	**Pyrenocine A**	226.1072	6.3	Find by Molecular Feature	208.0734

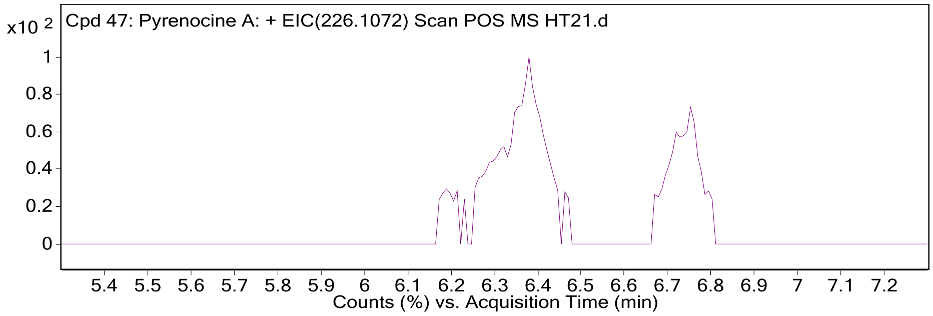

MFE MS Zoomed Spectrum

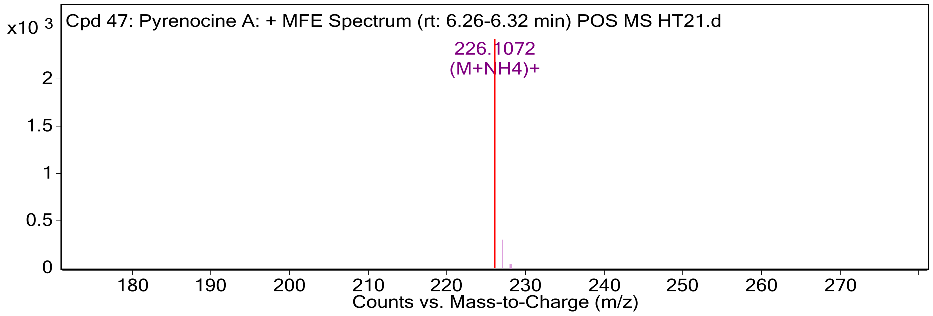

Compound Label	Name	*m/z*	RT	Algorithm	Mass
Cpd 48: Pyrenocine A	**Pyrenocine A**	226.1076	6.38	Find by Molecular Feature	208.0737

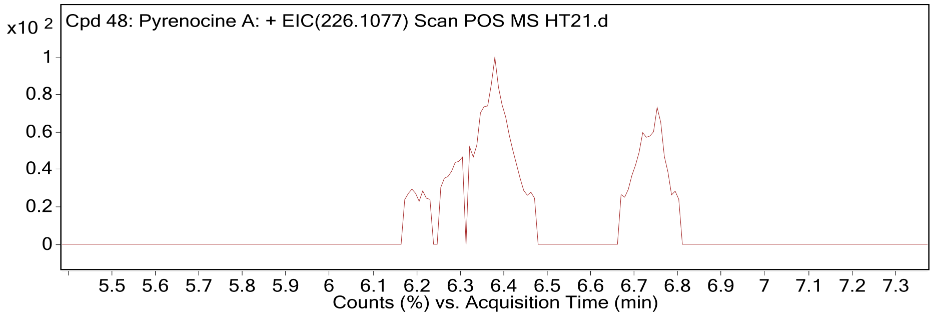

MFE MS Zoomed Spectrum

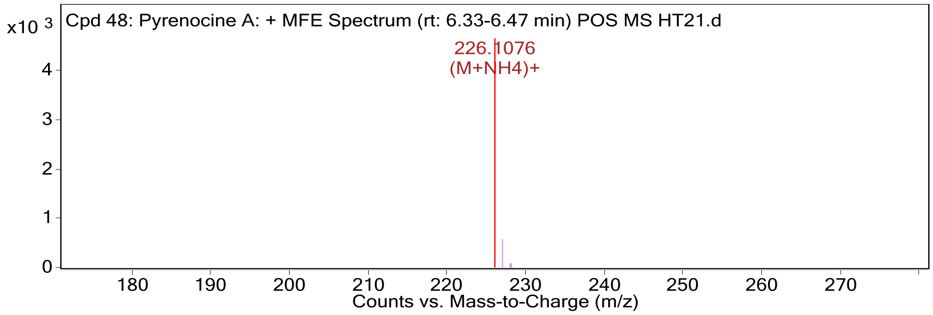

Compound Label	Name	*m/z*	RT	Algorithm	Mass
Cpd 49: Brevianamid F	**Brevianamid F**	284.1395	6.54	Find by Molecular Feature	283.1322

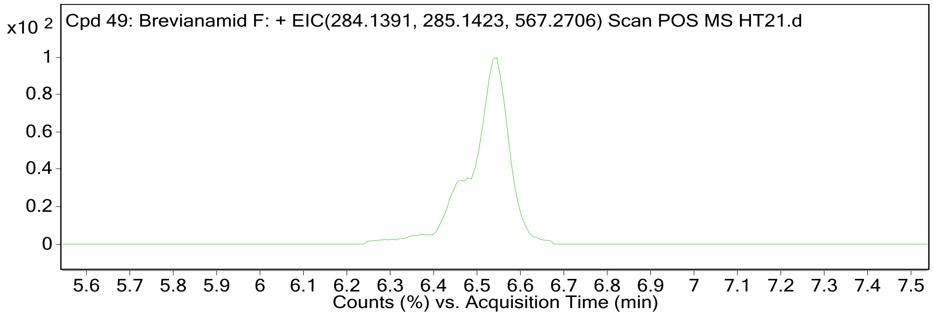

MFE MS Zoomed Spectrum

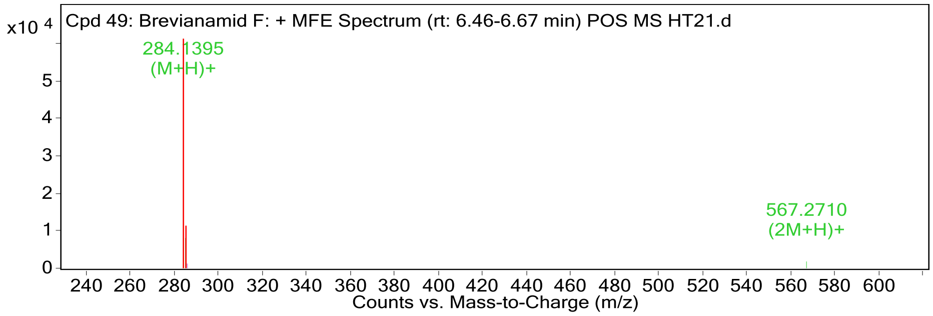

Compound Label	Name	*m/z*	RT	Algorithm	Mass

Cpd 50: Paspalic acid	**Paspalic acid**	286.1548	6.65	Find by Molecular Feature	268.121

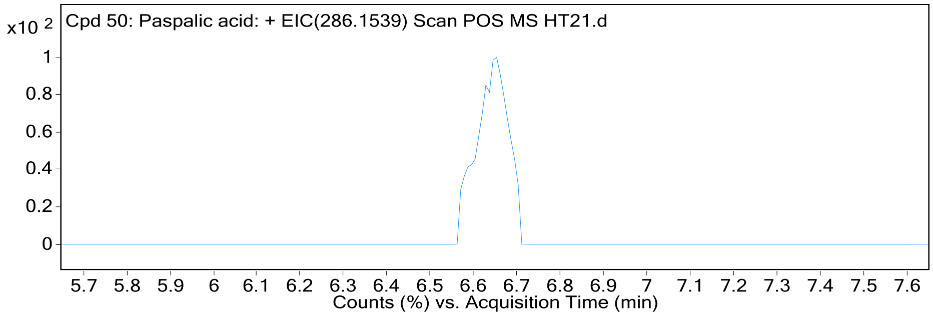

MFE MS Zoomed Spectrum

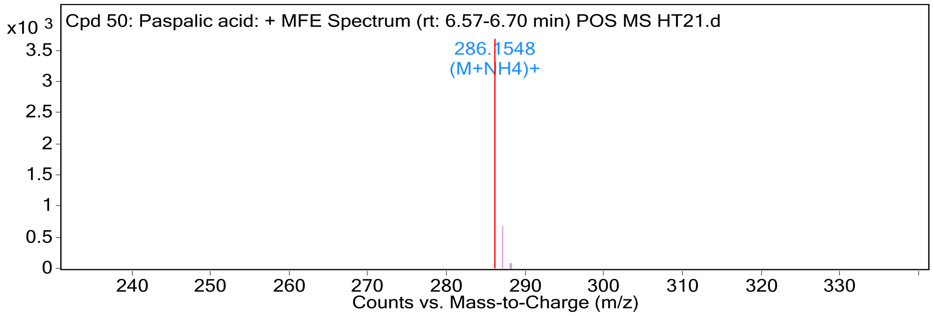

**Compound Label**	**Name**	***m/z***	**RT**	**Algorithm**	**Mass**
Cpd 51: Pyrenocine A	**Pyrenocine A**	226.1075	6.74	Find by Molecular Feature	208.0737

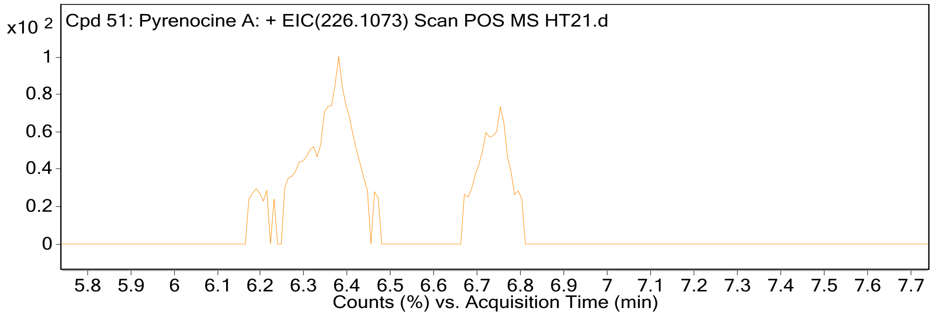

MFE MS Zoomed Spectrum

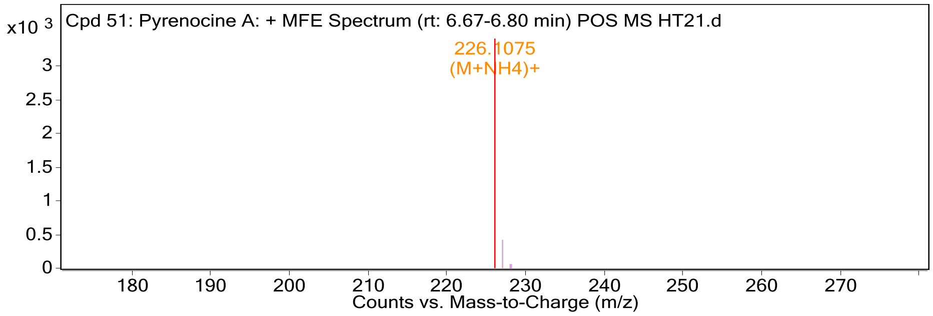

Compound Label	Name	*m/z*	RT	Algorithm	Mass
Cpd 52: Infectopyrone	**Infectopyrone**	282.1345	6.89	Find by Molecular Feature	264.1006

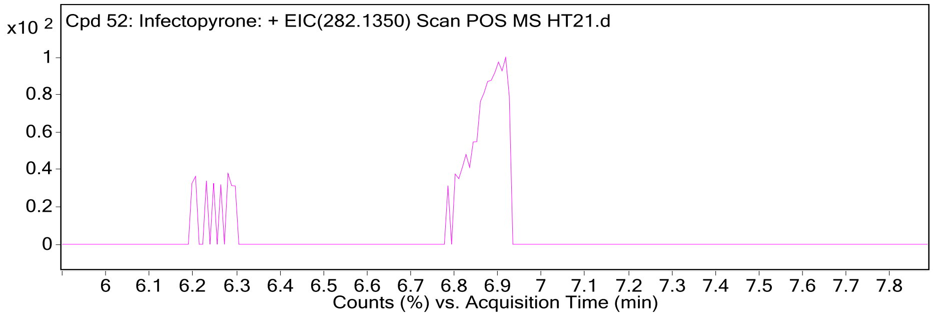

MFE MS Zoomed Spectrum

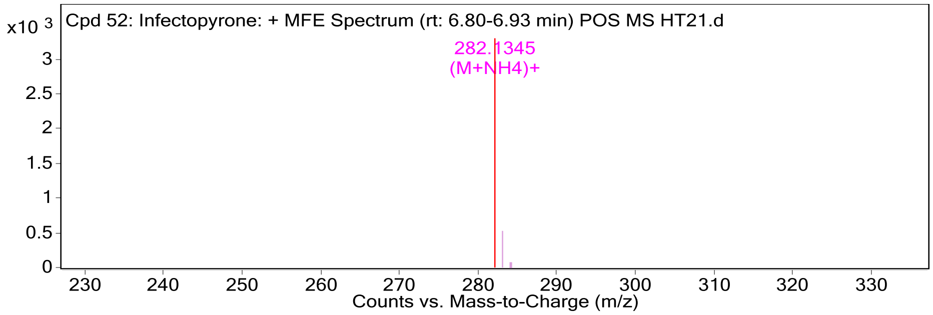

Compound Label	Name	*m/z*	RT	Algorithm	Mass
Cpd 53: Anisomycin	**Anisomycin**	266.1392	6.93	Find by Molecular Feature	265.1319

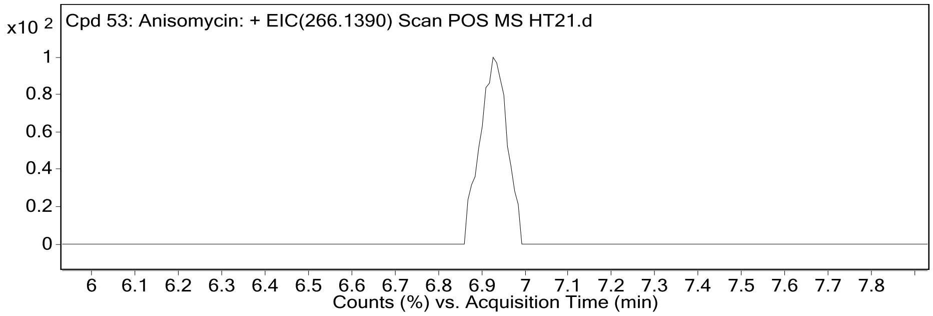

MFE MS Zoomed Spectrum

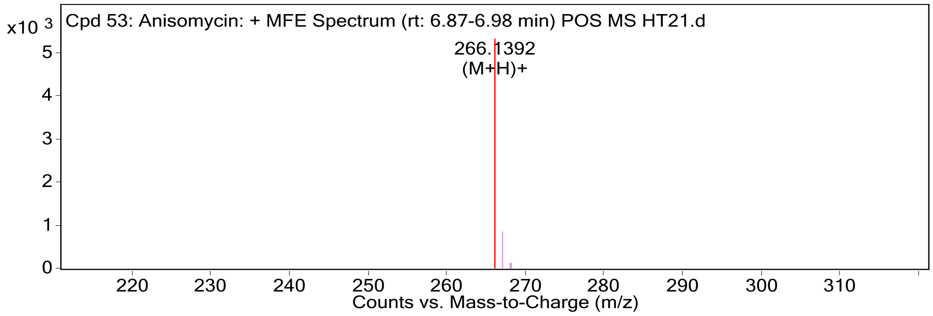

Compound Label	Name	*m/z*	RT	Algorithm	Mass
Cpd 54: Macrosporin	**Macrosporin**	307.0564	7.12	Find by Molecular Feature	284.0672

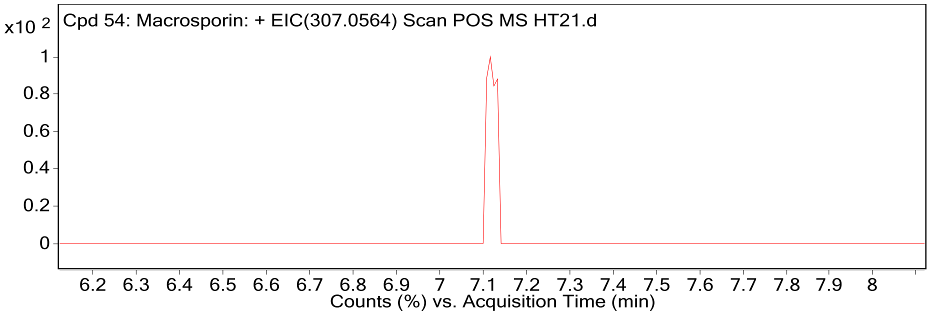

MFE MS Zoomed Spectrum

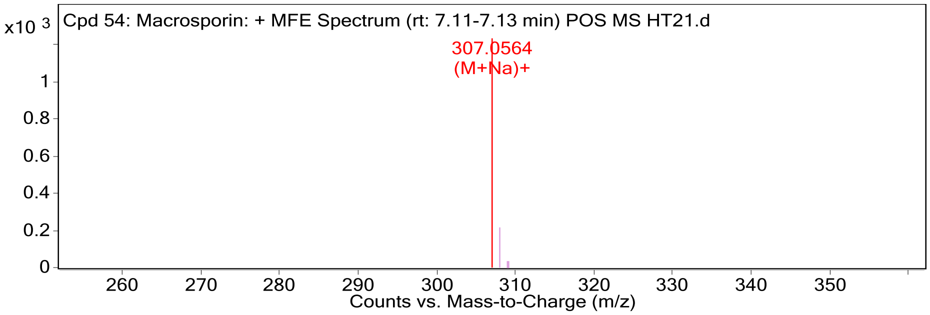

Compound Label	Name	*m/z*	RT	Algorithm	Mass
Cpd 55: Aphidicolin	**Aphidicolin**	377.2072	7.13	Find by Molecular Feature	338.2438

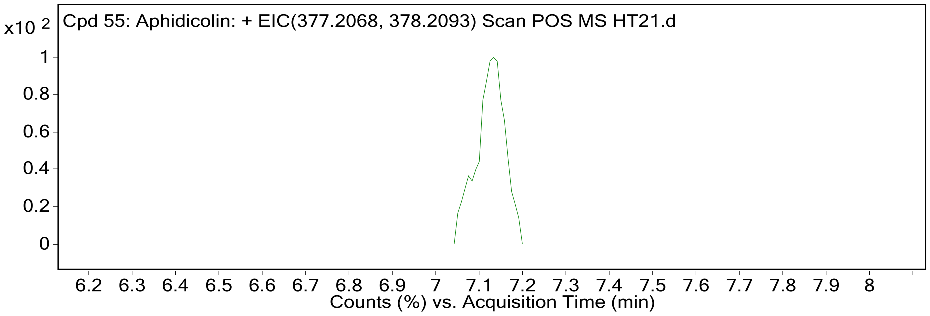

MFE MS Zoomed Spectrum

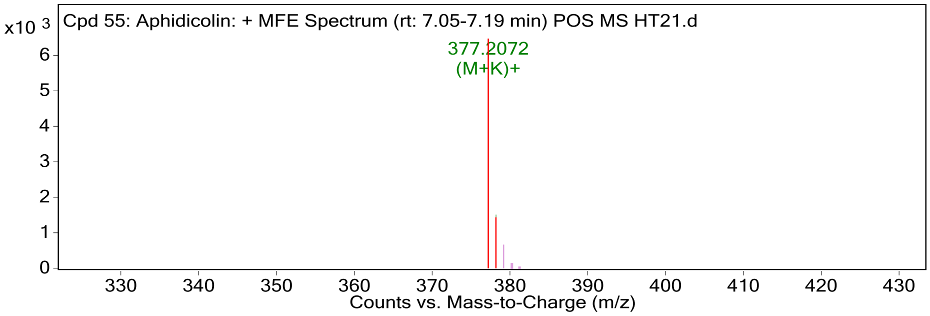

Compound Label	Name	*m/z*	RT	Algorithm	Mass
Cpd 56: DAS / Diacetoxyscirpenol	**DAS / Diacetoxyscirpenol**	384.2002	7.39	Find by Molecular Feature	366.1663

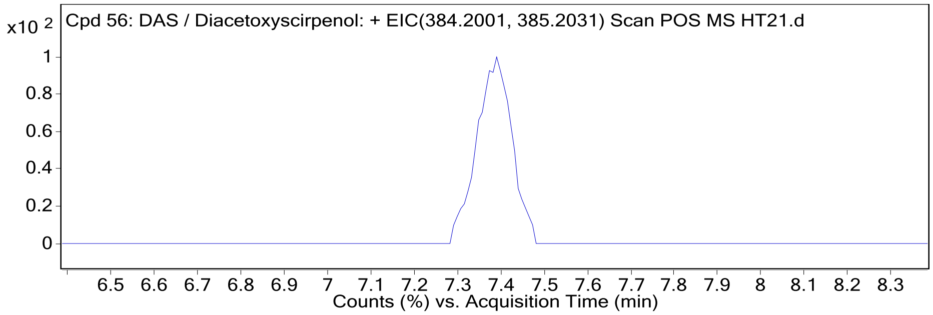

MFE MS Zoomed Spectrum

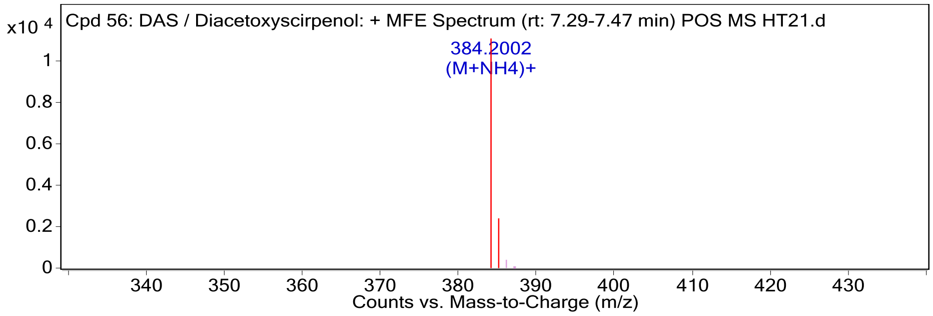

Compound Label	Name	*m/z*	RT	Algorithm	Mass
Cpd 57: Rugulosuvine	**Rugulosuvine**	334.1536	7.41	Find by Molecular Feature	333.1463

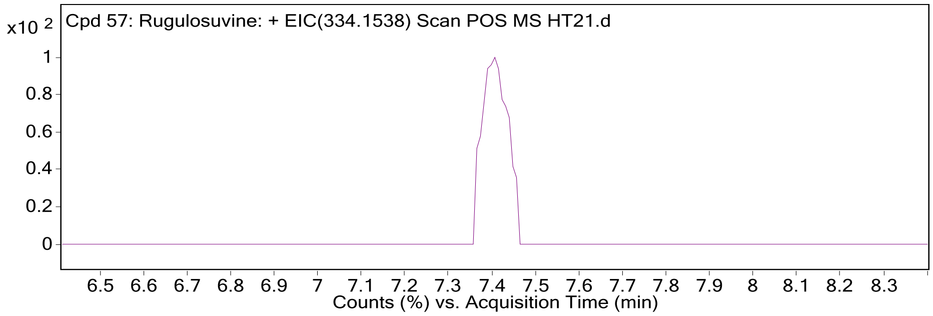

MFE MS Zoomed Spectrum

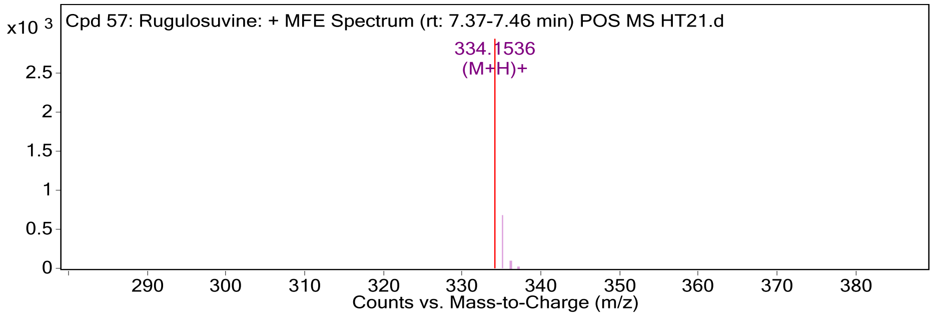

Compound Label	Name	*m/z*	RT	Algorithm	Mass
Cpd 58: Antibiotic L696,474	**Antibiotic L696,474**	516.253	7.44	Find by Molecular Feature	477.2898

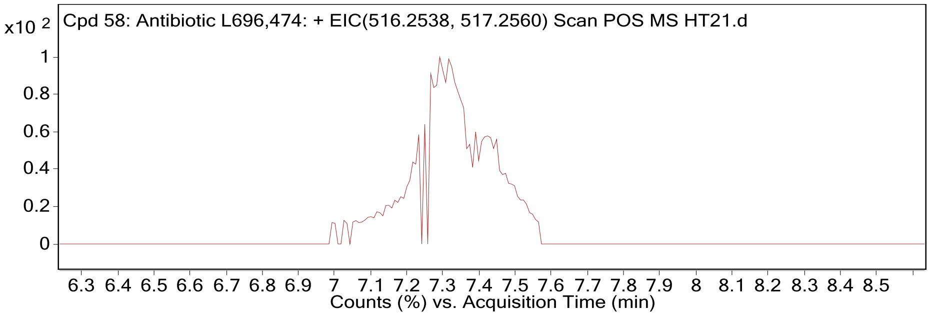

MFE MS Zoomed Spectrum

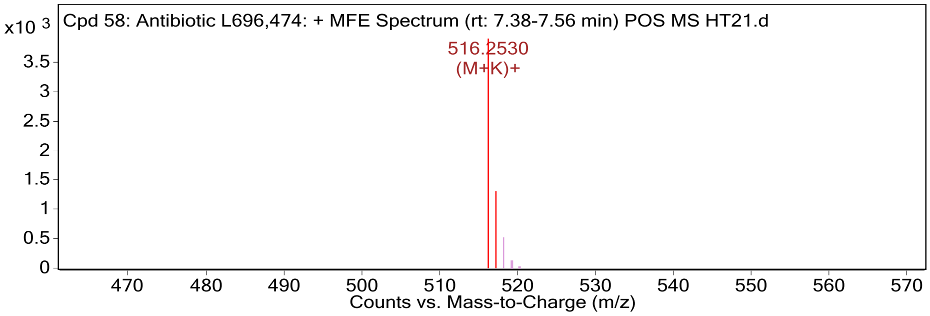

Compound Label	Name	*m/z*	RT	Algorithm	Mass
Cpd 59: beta-Zearalenol	**beta-Zearalenol**	338.1946	7.87	Find by Molecular Feature	320.1607

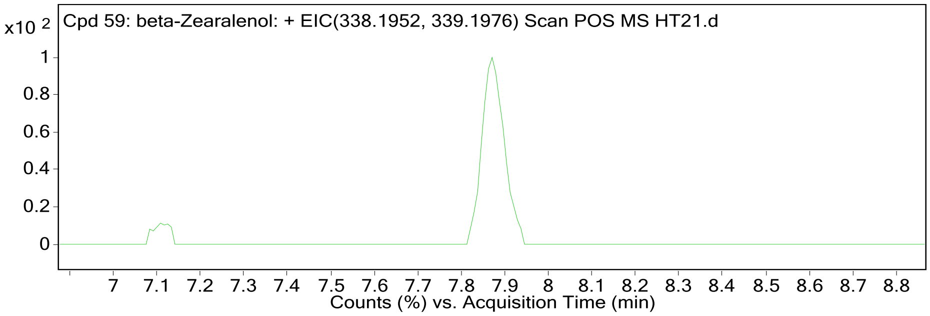

MFE MS Zoomed Spectrum

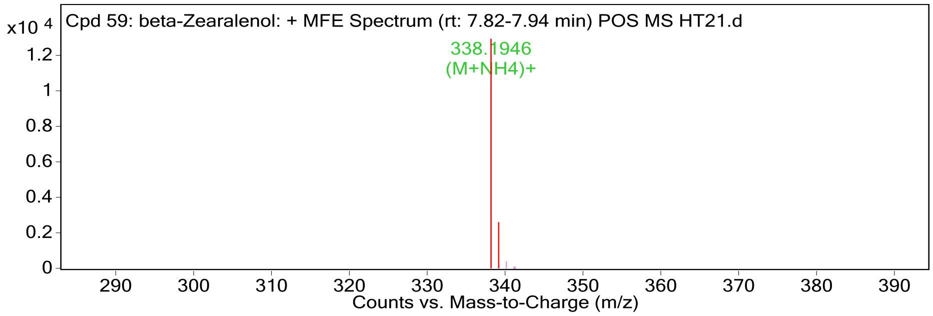

Compound Label	Name	*m/z*	RT	Algorithm	Mass
Cpd 60: Isofusidienol A	**Isofusidienol A**	318.0961	7.98	Find by Molecular Feature	300.0623

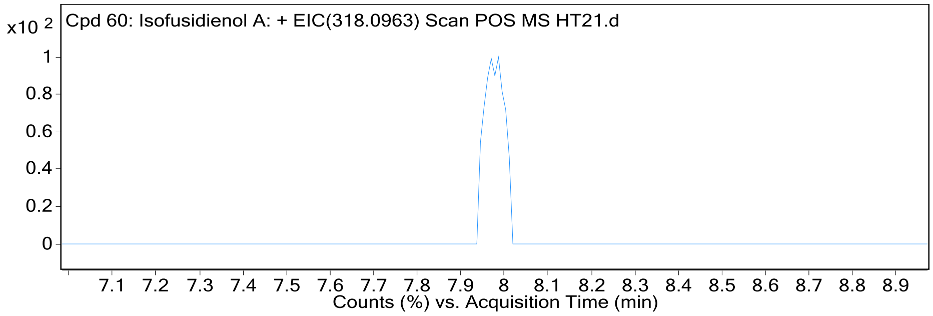

MFE MS Zoomed Spectrum

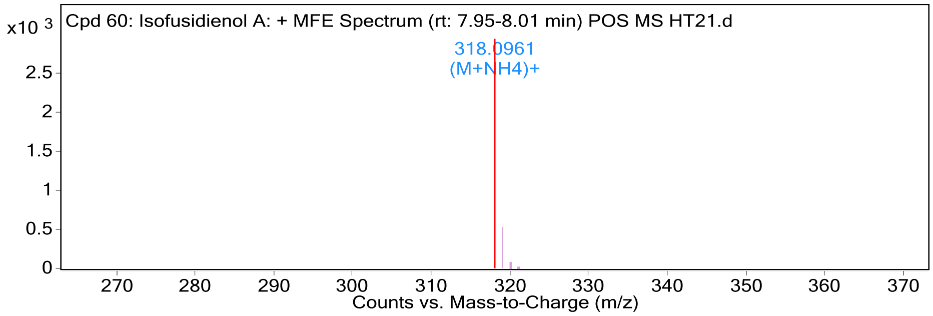

Compound Label	Name	*m/z*	RT	Algorithm	Mass
Cpd 61: [U-13C]-ZEN / Zearalenone	**[U-13C]-ZEN / Zearalenone**	359.1971	8.27	Find by Molecular Feature	336.2079

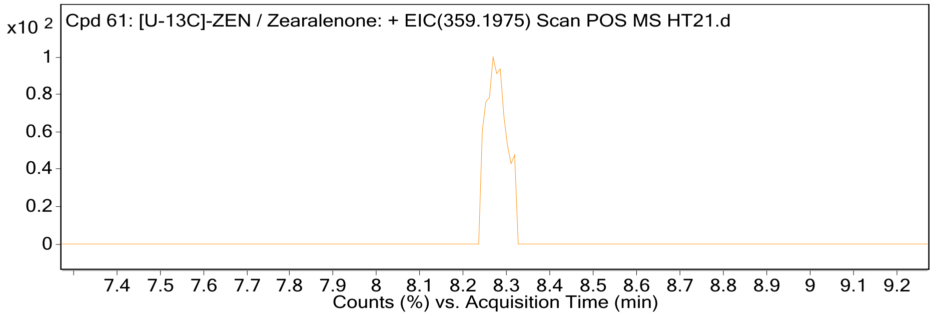

MFE MS Zoomed Spectrum

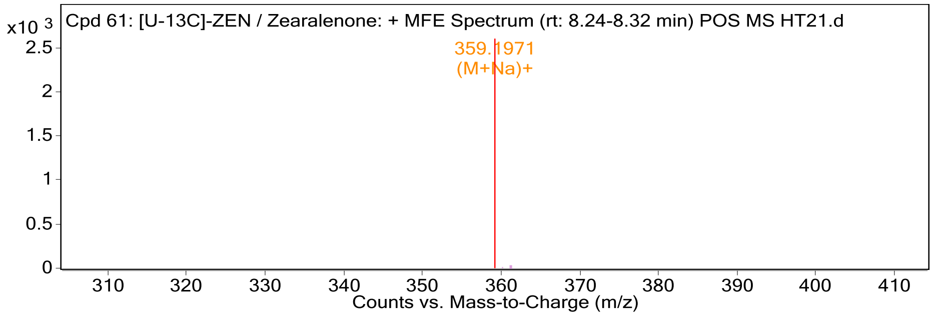

Compound Label	Name	*m/z*	RT	Algorithm	Mass
Cpd 62: Fusaric acid	**Fusaric acid**	180.1011	8.32	Find by Molecular Feature	179.0939

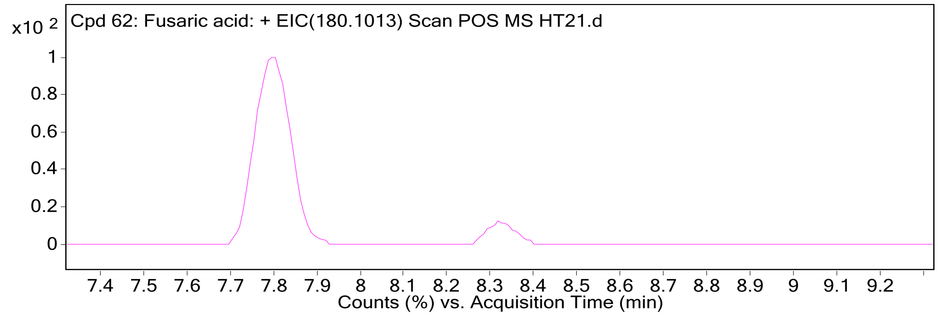

MFE MS Zoomed Spectrum

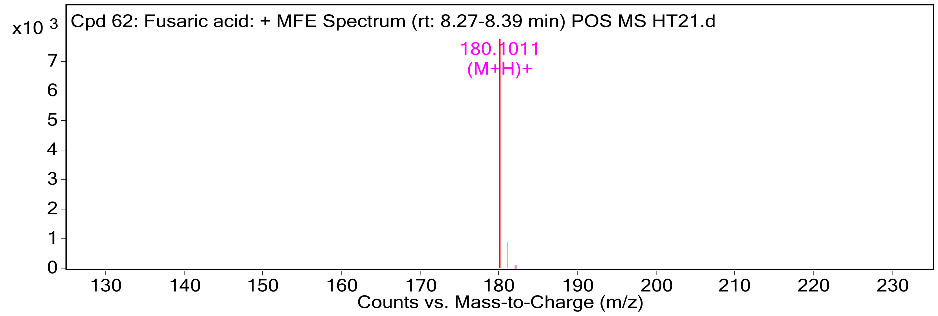

Compound Label	Name	*m/z*	RT	Algorithm	Mass
Cpd 63: Culmorin	**Culmorin**	256.2262	8.45	Find by Molecular Feature	238.1924

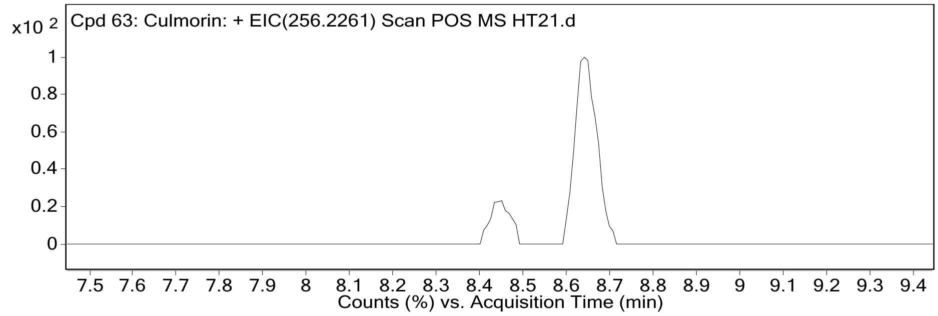

MFE MS Zoomed Spectrum

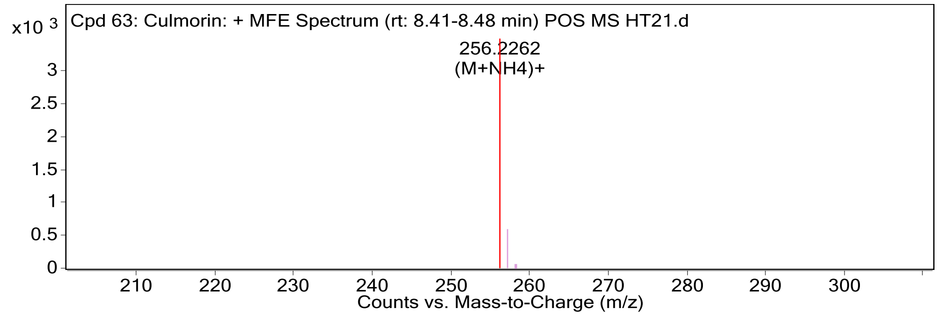

Compound Label	Name	*m/z*	RT	Algorithm	Mass
Cpd 64: Doxycycline	**Doxycycline**	462.1856	8.5	Find by Molecular Feature	444.1518

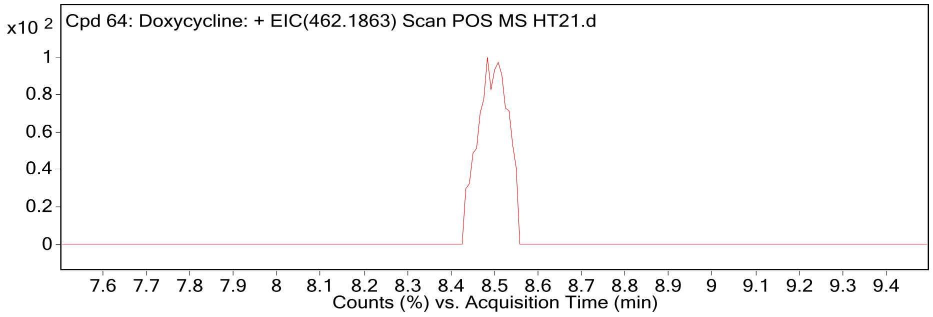

MFE MS Zoomed Spectrum

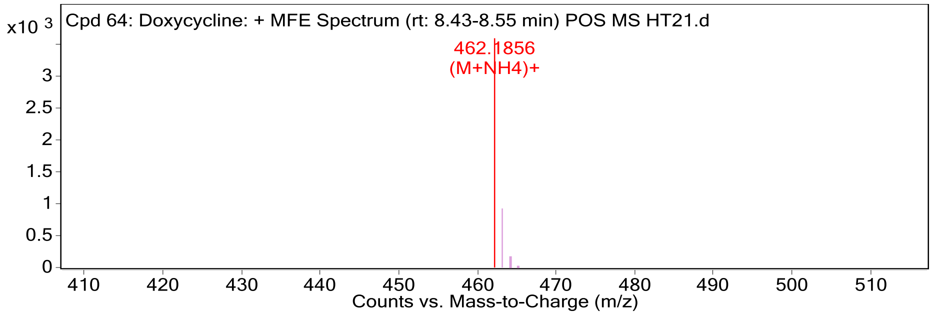

Compound Label	Name	*m/z*	RT	Algorithm	Mass
Cpd 65: Stachybotrylactam	**Stachybotrylactam**	403.2608	9.32	Find by Molecular Feature	385.227

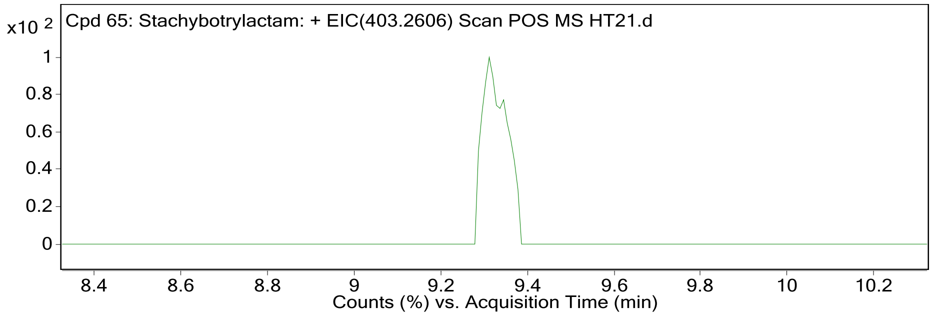

MFE MS Zoomed Spectrum

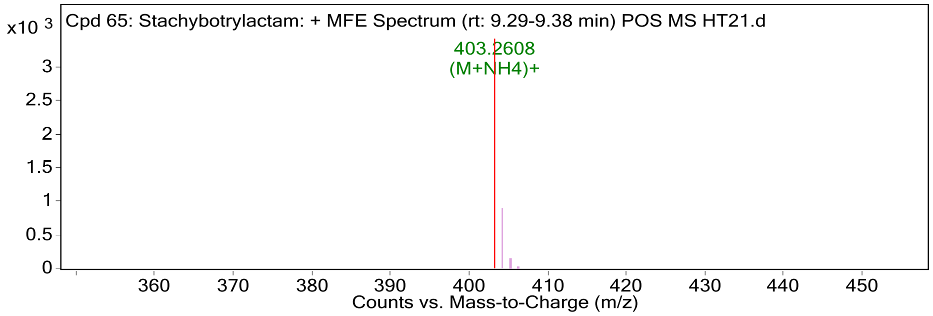

Compound Label	Name	*m/z*	RT	Algorithm	Mass

Cpd 66: FB6 / Fumonisin B6	**FB6 / Fumonisin B6**	722.3972	10.78	Find by Molecular Feature	721.3897

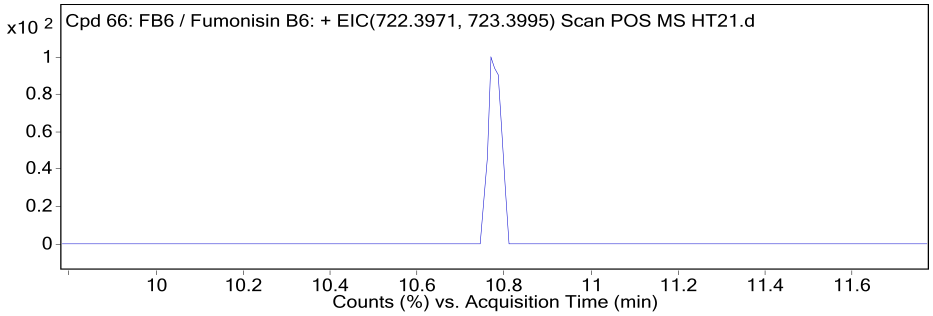

MFE MS Zoomed Spectrum

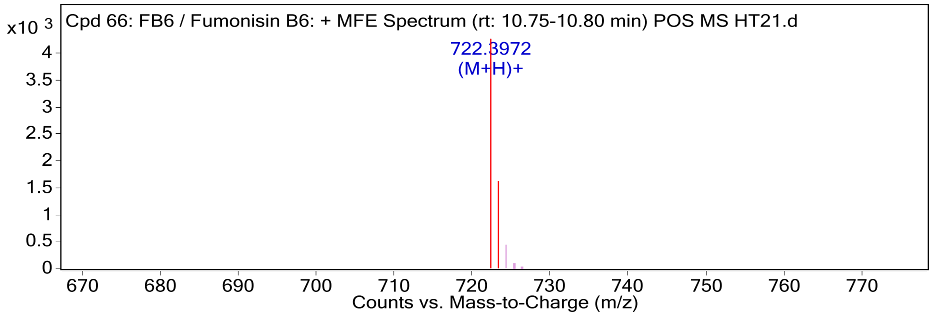

Compound Label	Name	*m/z*	RT	Algorithm	Mass
Cpd 67: Ionomycin	**Ionomycin**	731.5094	10.79	Find by Molecular Feature	708.5202

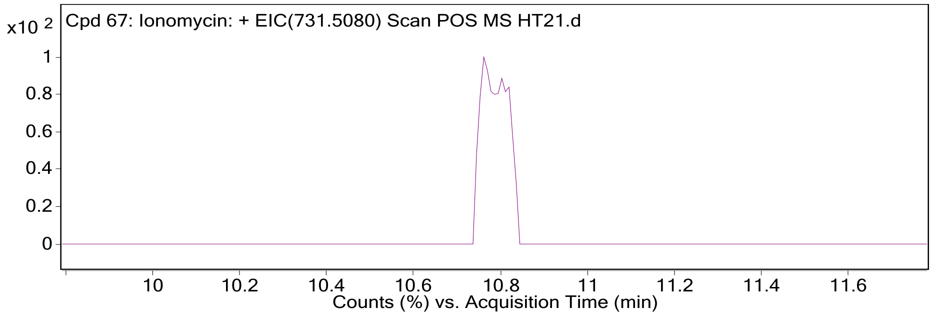

MFE MS Zoomed Spectrum

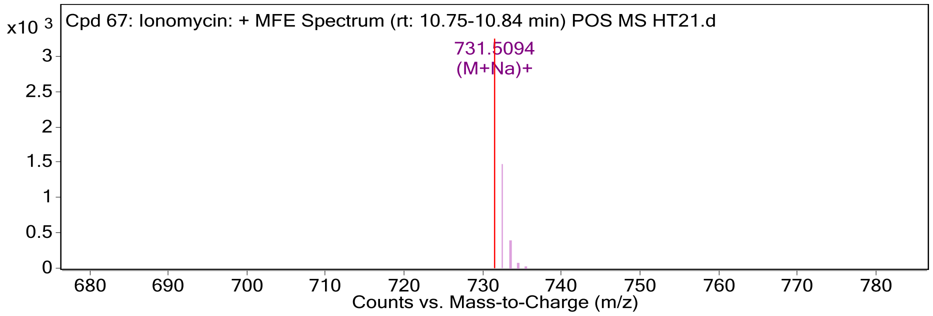

Compound Label	Name	*m/z*	RT	Algorithm	Mass
Cpd 68: Sirolimus (Rapamycin)	**Sirolimus (Rapamycin)**	931.5915	11.32	Find by Molecular Feature	913.5581

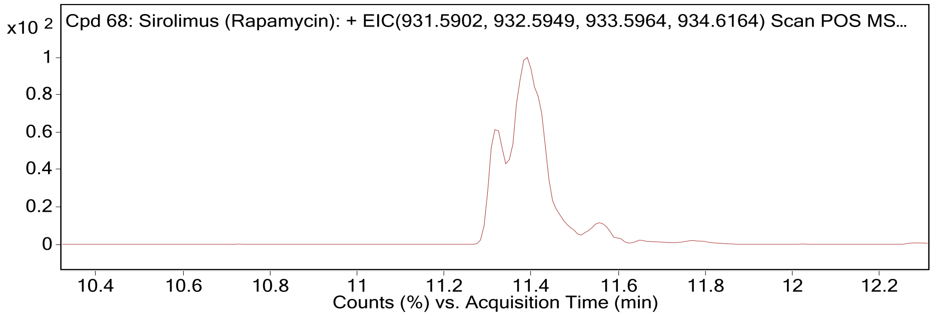

MFE MS Zoomed Spectrum

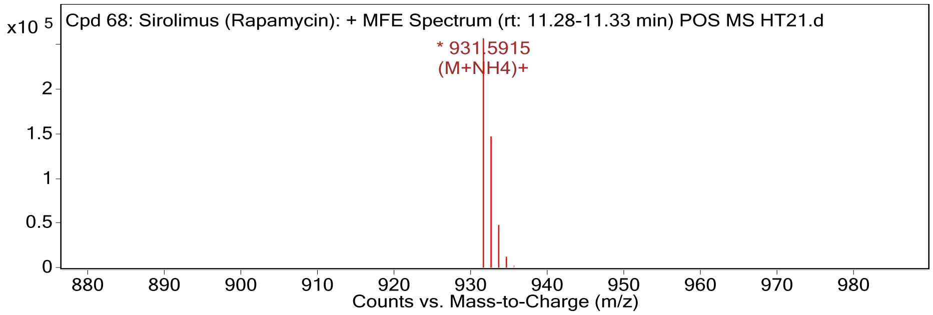

Compound Label	Name	*m/z*	RT	Algorithm	Mass
Cpd 69: Sirolimus (Rapamycin)	**Sirolimus (Rapamycin)**	931.5926	11.4	Find by Molecular Feature	913.5587

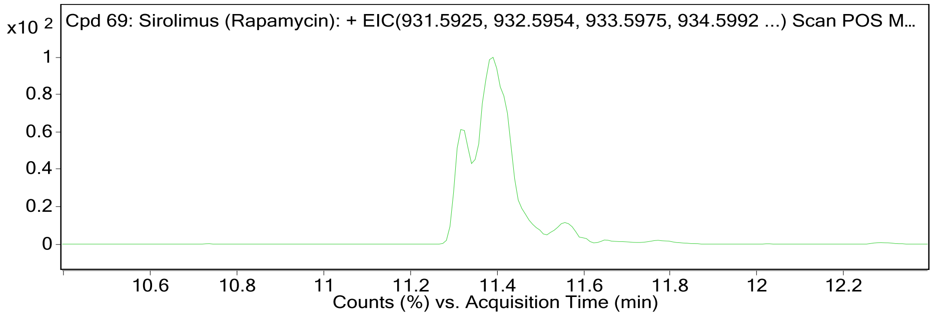

MFE MS Zoomed Spectrum

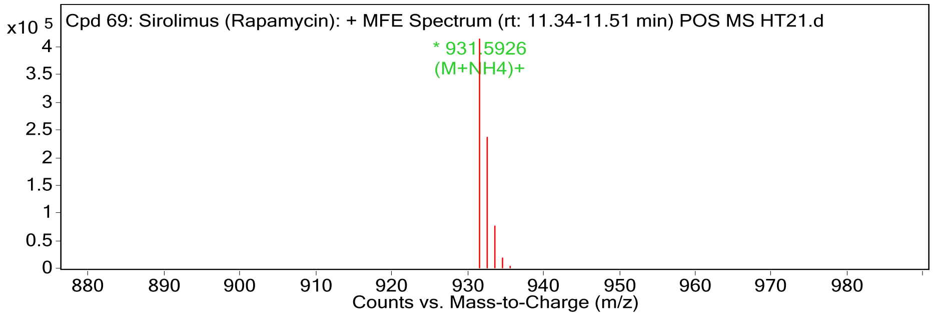

Compound Label	Name	*m/z*	RT	Algorithm	Mass
Cpd 70: Tryptoquialanine	**Tryptoquialanine**	536.2158	11.49	Find by Molecular Feature	518.1811

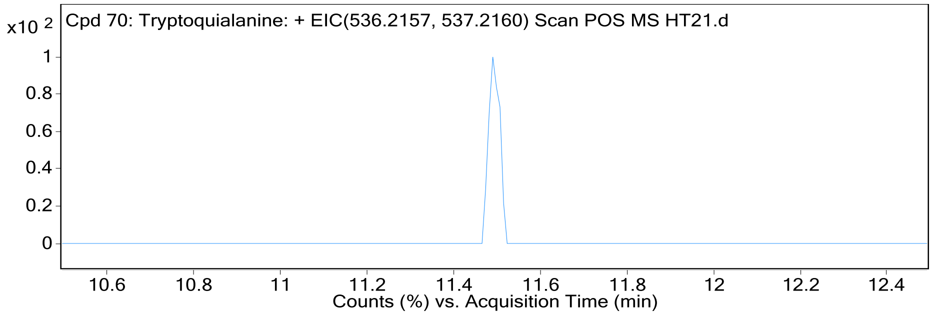

MFE MS Zoomed Spectrum

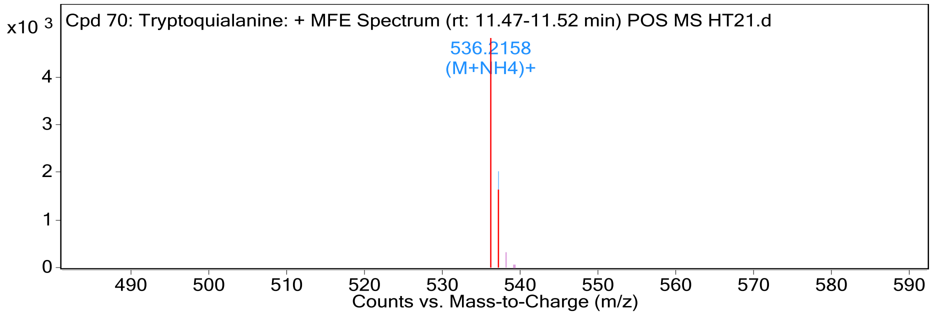

Compound Label	Name	*m/z*	RT	Algorithm	Mass
Cpd 71: Myriocin	**Myriocin**	419.3121	11.51	Find by Molecular Feature	401.2783

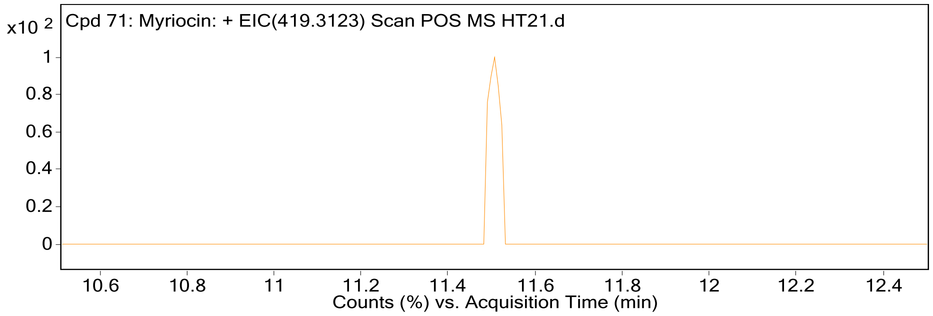

MFE MS Zoomed Spectrum

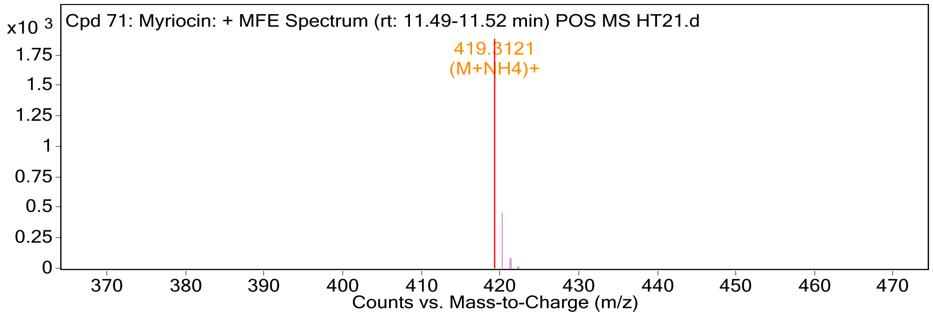

Compound Label	Name	*m/z*	RT	Algorithm	Mass
Cpd 72: Sirolimus (Rapamycin)	**Sirolimus (Rapamycin)**	931.5925	11.56	Find by Molecular Feature	913.5585

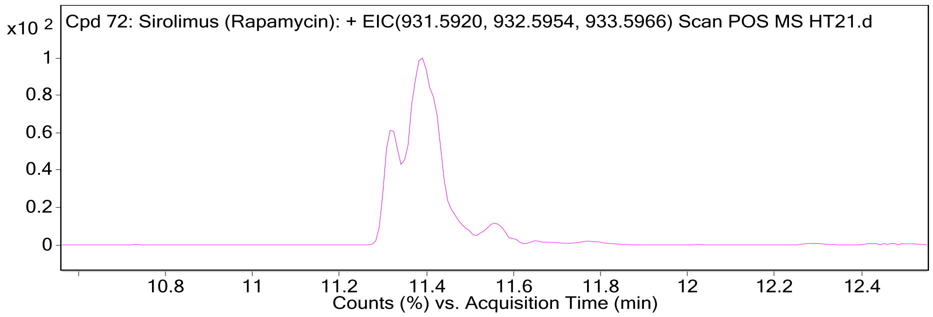

MFE MS Zoomed Spectrum

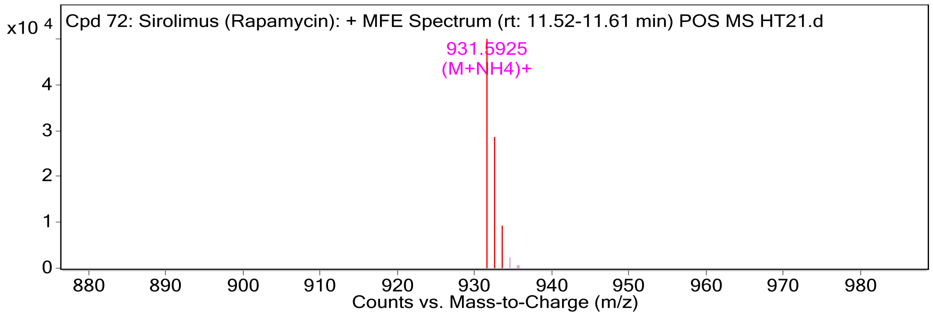

Compound Label	Name	*m/z*	RT	Algorithm	Mass
Cpd 73: [U-13C]-FB1 / Fumonisin B1	**[U-13C]-FB1 / Fumonisin B1**	773.5327	11.65	Find by Molecular Feature	755.4989

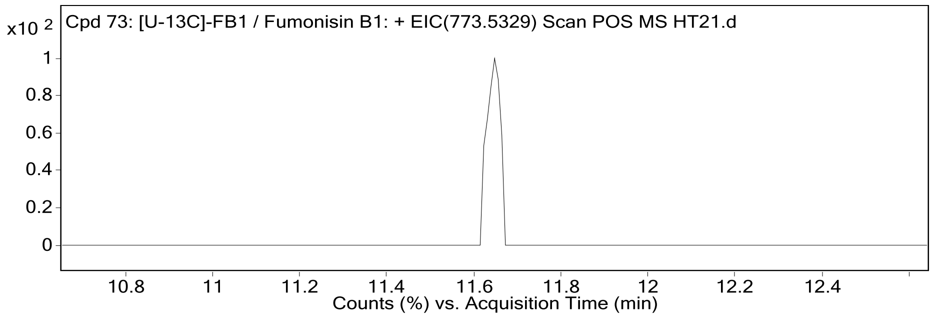

MFE MS Zoomed Spectrum

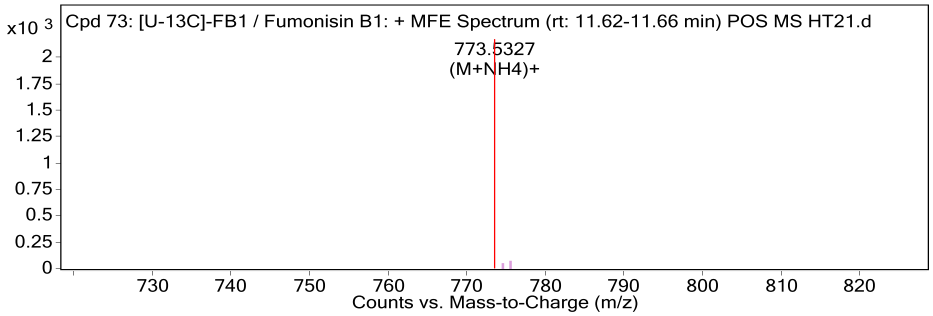

Compound Label	Name	*m/z*	RT	Algorithm	Mass
Cpd 74: Sirolimus (Rapamycin)	**Sirolimus (Rapamycin)**	931.5916	11.66	Find by Molecular Feature	913.5578

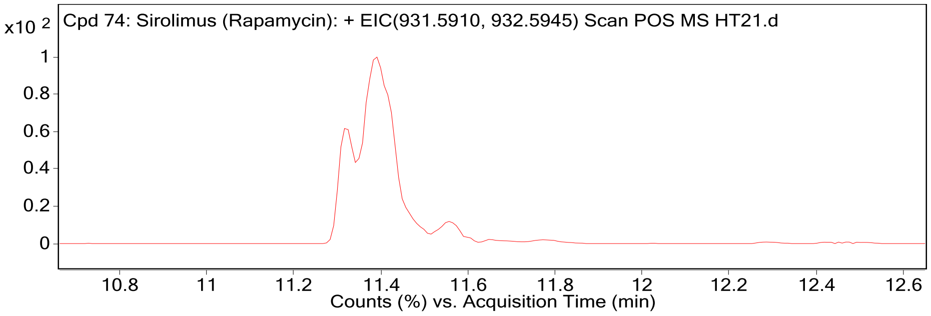

MFE MS Zoomed Spectrum

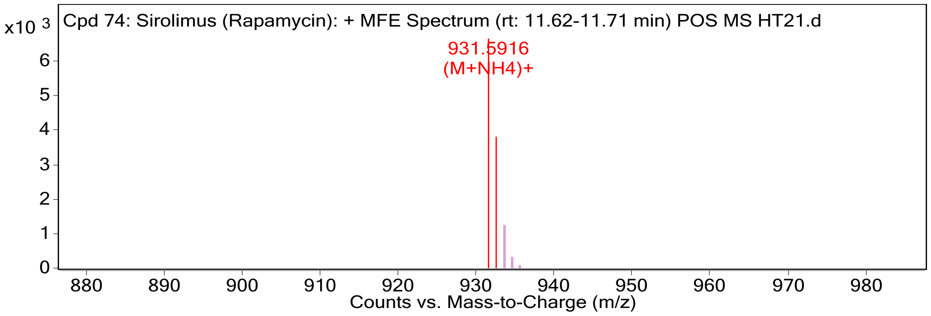

Compound Label	Name	*m/z*	RT	Algorithm	Mass
Cpd 75: Sirolimus (Rapamycin)	**Sirolimus (Rapamycin)**	931.5913	11.78	Find by Molecular Feature	913.5574

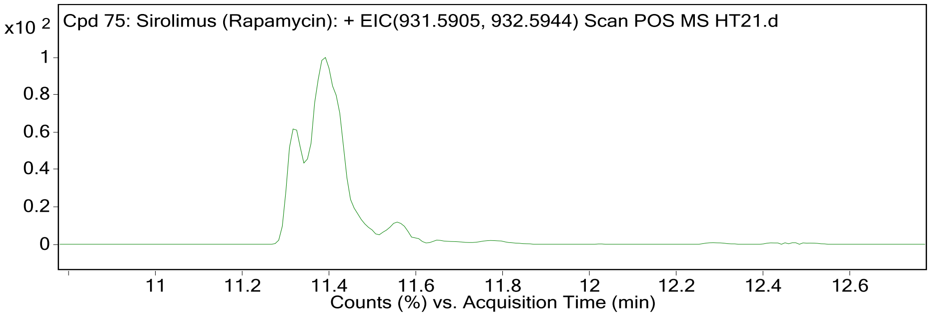

MFE MS Zoomed Spectrum

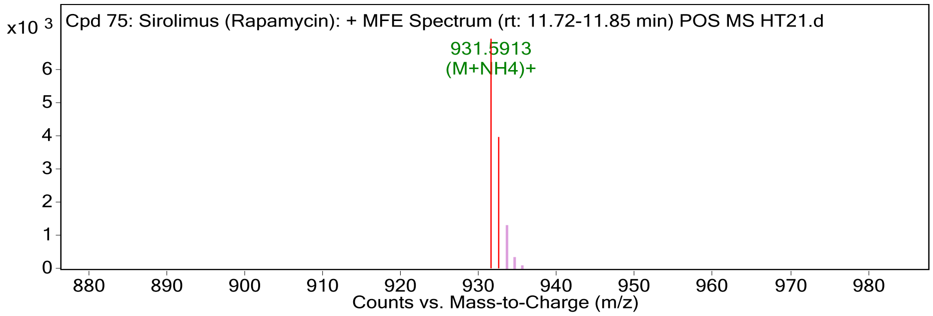

Compound Label	Name	*m/z*	RT	Algorithm	Mass
Cpd 76: [U-13C]-FB2 / Fumonisin B2	**[U-13C]-FB2 / Fumonisin B2**	757.5384	11.78	Find by Molecular Feature	739.5049

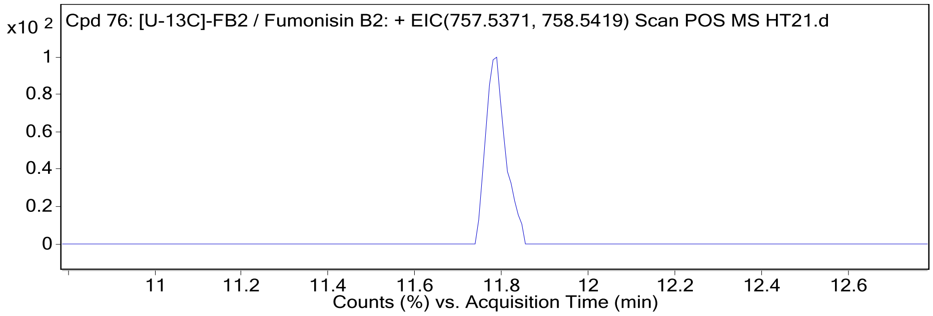

MFE MS Zoomed Spectrum

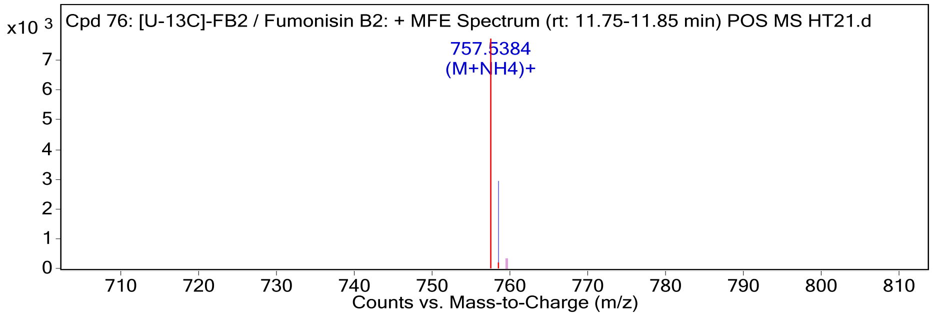

Compound Label	Name	*m/z*	RT	Algorithm	Mass
Cpd 77: Fusapyrone	**Fusapyrone**	607.3863	11.81	Find by Molecular Feature	606.3791

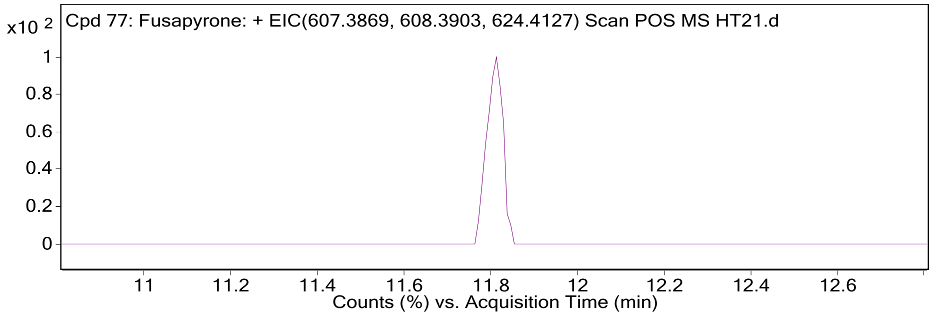

MFE MS Zoomed Spectrum

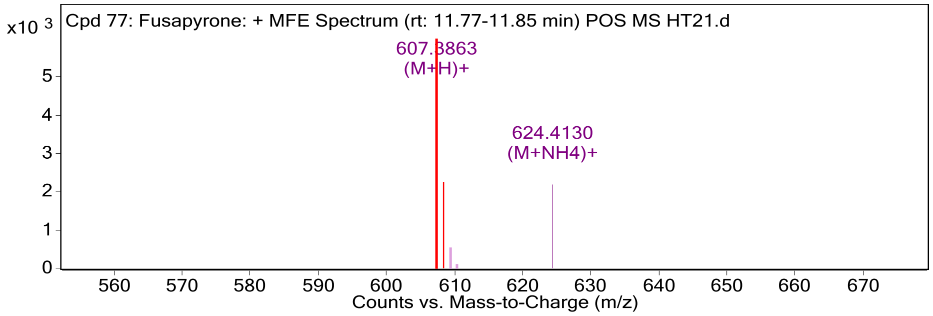

Compound Label	Name	*m/z*	RT	Algorithm	Mass
Cpd 78: Sirolimus (Rapamycin)	**Sirolimus (Rapamycin)**	931.5909	12.29	Find by Molecular Feature	913.5567

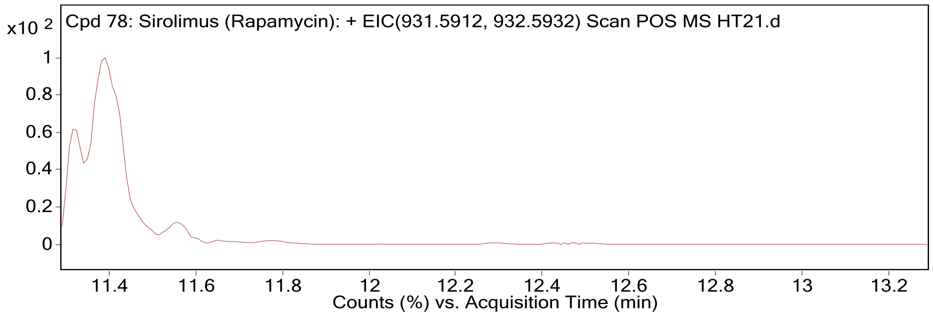

MFE MS Zoomed Spectrum

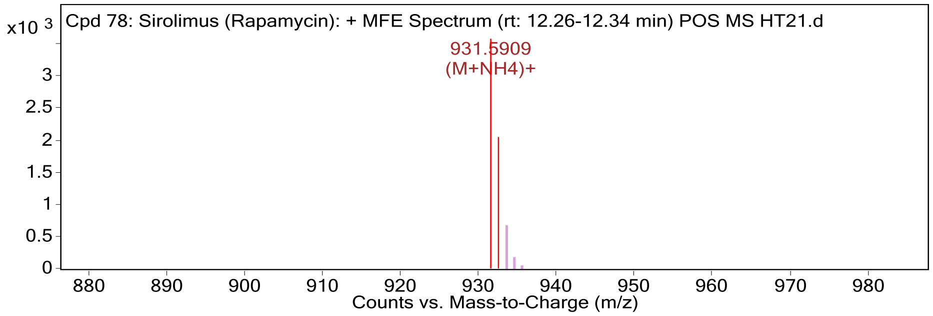

Compound Label	Name	*m/z*	RT	Algorithm	Mass
Cpd 79: Sirolimus (Rapamycin)	**Sirolimus (Rapamycin)**	931.5924	12.46	Find by Molecular Feature	913.5586

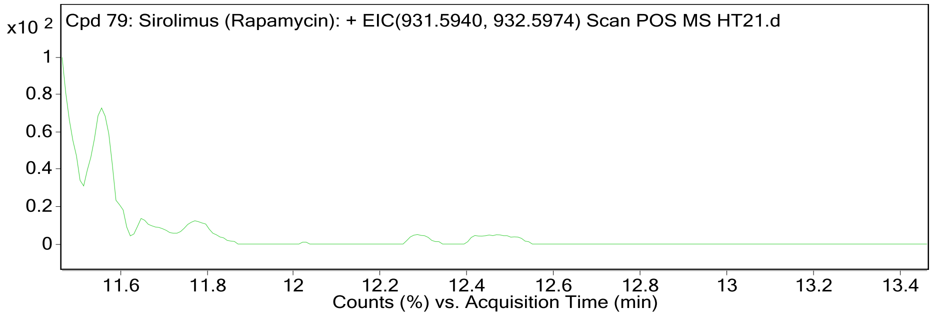

MFE MS Zoomed Spectrum

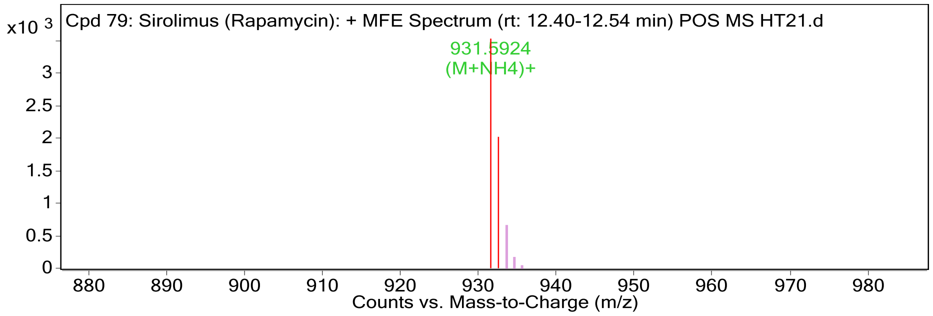

--- End Of Report ---

Qualitative Compound Report

**Data File**	POS MS HT30.d	**Sample Name**	HT30
**Sample Type**	Sample	**Position**	P1-A4
**Instrument Name**	Instrument 1	**User Name**	
**Acq Method**	Mycotoxins POS.m	**Acquired Time**	6/15/2022 12:02:48 PM
**IRM Calibration Status**	Success	**DA Method**	scau default.m
**Comment**			

**Sample Group**		**Info.**	
**Stream Name**	LC 1	**Acquisition SW Version**	6200 series TOF/6500 series Q-TOF B.06.01 (B6157)

Fragmentor Voltage 130 **Collision Energy** 0 **Ionization Mode** ESI

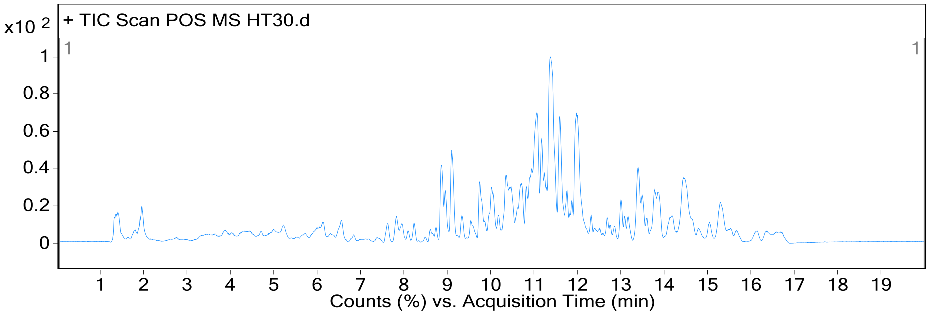

Fragmentor Voltage 130 **Collision Energy** 0 **Ionization Mode** ESI

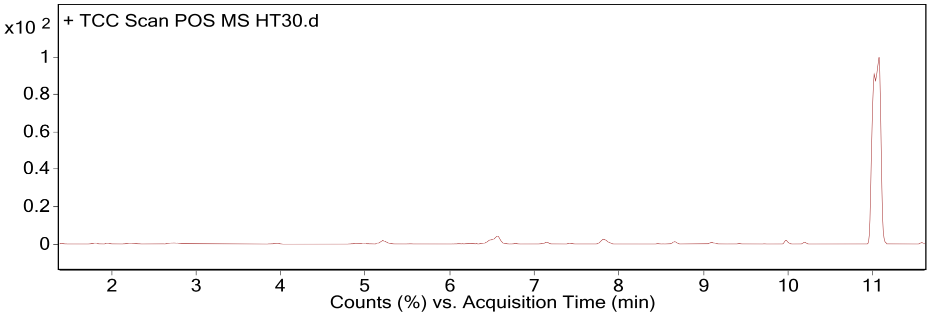

Compound Table

Compound Label	RT	Mass	Name	Formula	Tgt Mass	Diff (ppm)	SignificantIonMz
Cpd 1: Fusarinolic acid	1.42	195.0894	Fusarinolic acid	C10 H13 N O3	195.0895	-0.91	213.1232
Cpd 2: Cerulenin	1.81	223.1207	Cerulenin	C12 H17 N O3	223.1208	-0.46	241.1546
Cpd 3: Fusarinolic acid	1.96	195.0882	Fusarinolic acid	C10 H13 N O3	195.0895	-6.75	196.097
Cpd 4: Decarestrictine D	2.23	216.0996	Decarestrictine D	C10 H16 O5	216.0998	-0.64	234.1335
Cpd 5: Terrein	2.74	154.0629	Terrein	C8 H10 O3	154.063	-0.42	172.0968
Cpd 6: Terrein	3.95	154.0629	Terrein	C8 H10 O3	154.063	-0.71	172.0967
Cpd 7: Fusaric acid	4.87	179.0949	Fusaric acid	C10 H13 N O2	179.0946	1.43	197.1287
Cpd 8: Fusaric acid	4.99	179.0947	Fusaric acid	C10 H13 N O2	179.0946	0.52	197.1285
Cpd 9: OTB / Ochratoxin B	4.99	369.1214	OTB / Ochratoxin B	C20 H19 N O6	369.1212	0.47	387.1552
Cpd 10: Cerulenin	5.17	223.1209	Cerulenin	C12 H17 N O3	223.1208	0.26	241.1547
Cpd 11: Fusaric acid	5.2	179.0948	Fusaric acid	C10 H13 N O2	179.0946	0.73	197.1286
Cpd 12: Oosporein	5.22	306.0376	Oosporein	C14 H10 O8	306.0376	-0.03	329.0268
Cpd 13: Oosporein	5.22	306.0377	Oosporein	C14 H10 O8	306.0376	0.51	307.045
Cpd 14: Cerulenin	5.53	223.121	Cerulenin	C12 H17 N O3	223.1208	0.81	241.1548
Cpd 15: Antimycin	6.11	250.0842	Antimycin	C13 H14 O5	250.0841	0.27	251.0915
Cpd 16: Fusaric acid	6.24	179.0947	Fusaric acid	C10 H13 N O2	179.0946	0.3	180.102
Cpd 17: Pyrenocine A	6.4	208.0736	Pyrenocine A	C11 H12 O4	208.0736	0.42	226.1075
Cpd 18: Brevianamid F	6.55	283.1325	Brevianamid F	C16 H17 N3 O2	283.1321	1.63	284.1398

Cpd 19: Paspalic acid	6.67	268.1211	Paspalic acid	C16 H16 N2 O2	268.1212	-0.2	286.155
Cpd 20: Pyrenocine A	6.78	208.0736	Pyrenocine A	C11 H12 O4	208.0736	0.09	226.1074
Cpd 21: Pestalotin	6.78	214.1204	Pestalotin	C11 H18 O4	214.1205	-0.55	232.1542
Cpd 22: beta-Zearalenol	7.14	320.162	beta-Zearalenol	C18 H24 O5	320.1624	-1.01	338.1959
Cpd 23: Macrosporin	7.14	284.0673	Macrosporin	C16 H12 O5	284.0685	-4.19	307.0565
Cpd 24: Aphidicolin	7.15	338.2438	Aphidicolin	C20 H34 O4	338.2457	-5.73	377.2072
Cpd 25: DAS / Diacetoxyscirpenol	7.41	366.1677	DAS / Diacetoxyscirpenol	C19 H26 O7	366.1679	-0.32	384.2016
Cpd 26: Rugulosuvine	7.42	333.1478	Rugulosuvine	C20 H19 N3 O2	333.1477	0.2	334.1551
Cpd 27: Cladosporin	7.45	292.1309	Cladosporin	C16 H20 O5	292.1311	-0.72	310.1647
Cpd 28: Fusaric acid	7.82	179.0948	Fusaric acid	C10 H13 N O2	179.0946	0.77	180.102
Cpd 29: beta-Zearalenol	7.89	320.1624	beta-Zearalenol	C18 H24 O5	320.1624	0.15	338.1962
Cpd 30: Culmorin	8.46	238.1934	Culmorin	C15 H26 O2	238.1933	0.52	256.2272
Cpd 31: Siccanol	8.53	402.2759	Siccanol	C25 H38 O4	402.277	-2.65	441.2391
Cpd 32: 15-Hydroxyculmorone	8.63	252.1728	15-Hydroxyculmorone	C15 H24 O3	252.1725	1.15	270.2067
Cpd 33: Culmorin	8.66	238.1933	Culmorin	C15 H26 O2	238.1933	0.24	256.2272
Cpd 34: 15-Hydroxyculmorone	8.68	252.1724	15-Hydroxyculmorone	C15 H24 O3	252.1725	-0.39	270.2063
Cpd 35: Deepoxy deoxynivalenol	9.11	280.1304	Deepoxy deoxynivalenol	C15 H20 O5	280.1311	-2.49	281.1377
Cpd 36: Cytochalasin E	9.29	495.2256	Cytochalasin E	C28 H33 N O7	495.2257	-0.27	513.2594
Cpd 37: Fusaproliferin	9.43	444.2863	Fusaproliferin	C27 H40 O5	444.2876	-2.95	483.2494
Cpd 38: 5-Methoxysterigmatocystin	9.73	354.0749	5-Methoxysterigmatocystin	C19 H14 O7	354.074	2.58	393.038
Cpd 39: Paspaline	9.98	421.2973	Paspaline	C28 H39 N O2	421.2981	-1.8	439.3313
Cpd 40: Aphidicolin	10.2	338.2447	Aphidicolin	C20 H34 O4	338.2457	-3.07	356.2786
Cpd 41: Enniatin A2	10.99	681.453	Enniatin A2	C36 H63 N3 O9	681.4564	-5.08	704.4424
Cpd 42: Enniatin A2	11	681.4532	Enniatin A2	C36 H63 N3 O9	681.4564	-4.68	699.487
Cpd 43: Beauvericin	11.06	783.4059	Beauvericin	C45 H57 N3 O9	783.4095	-4.58	801.4397
Cpd 44: Sirolimus (Rapamycin)	11.58	913.5581	Sirolimus (Rapamycin)	C51 H79 N O13	913.5551	3.27	931.5919

Compound Label	Name	*m/z*	RT	Algorithm	Mass
Cpd 1: Fusarinolic acid	**Fusarinolic acid**	213.1232	1.42	Find by Molecular Feature	195.0894

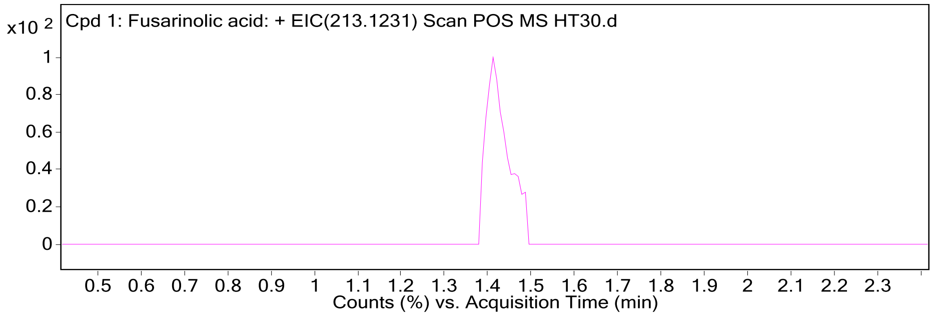

MFE MS Zoomed Spectrum

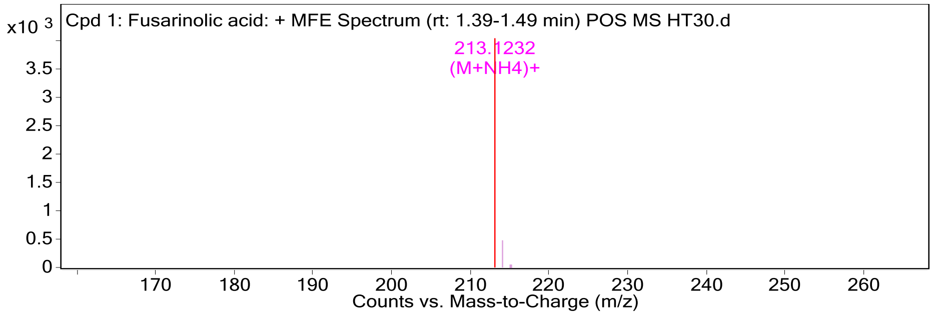

Qualitative Compound Report

Compound Label	Name	*m/z*	RT	Algorithm	Mass
Cpd 2: Cerulenin	**Cerulenin**	241.1546	1.81	Find by Molecular Feature	223.1207

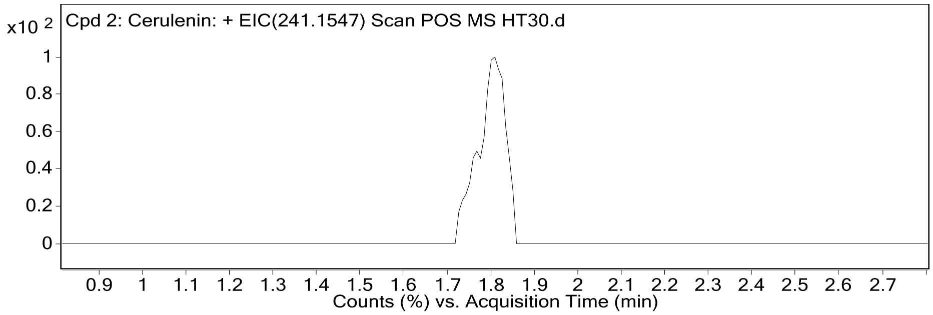

MFE MS Zoomed Spectrum

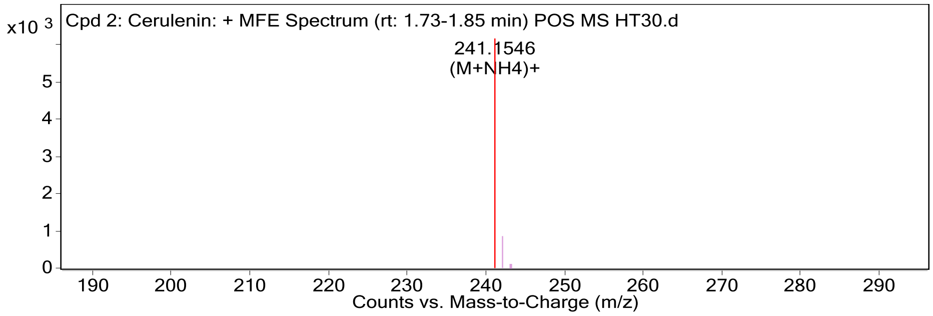

Compound Label	Name	*m/z*	RT	Algorithm	Mass
Cpd 3: Fusarinolic acid	**Fusarinolic acid**	196.097	1.96	Find by Molecular Feature	195.0882

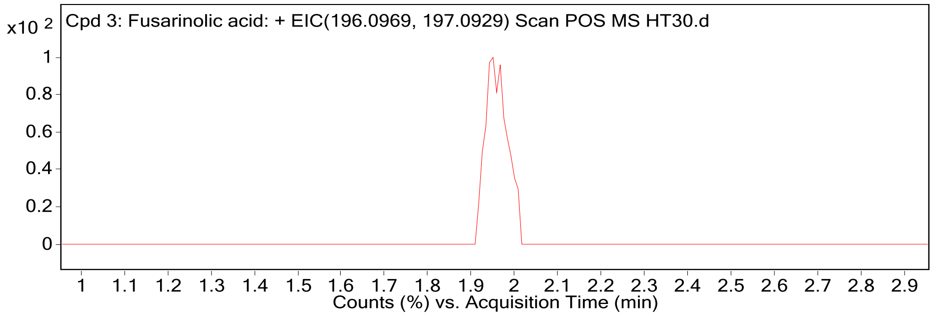

MFE MS Zoomed Spectrum

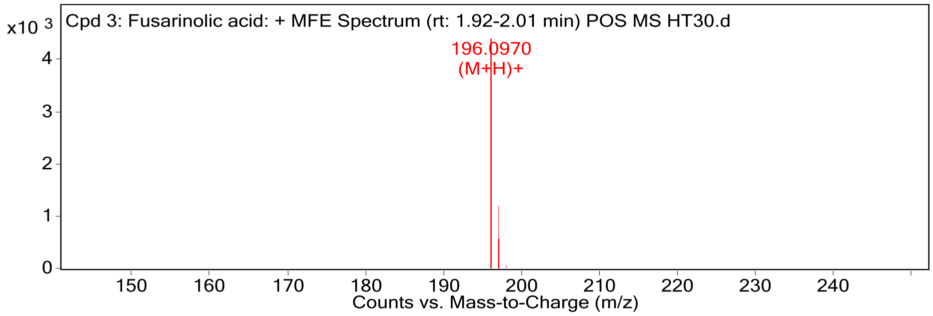

Compound Label	Name	*m/z*	RT	Algorithm	Mass
Cpd 4: Decarestrictine D	**Decarestrictine D**	234.1335	2.23	Find by Molecular Feature	216.0996

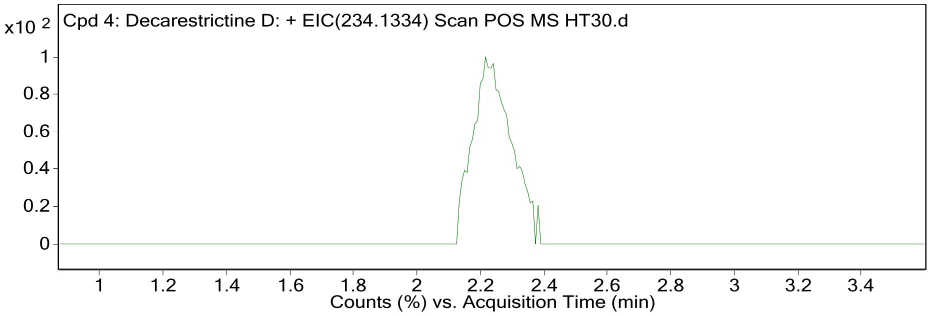

MFE MS Zoomed Spectrum

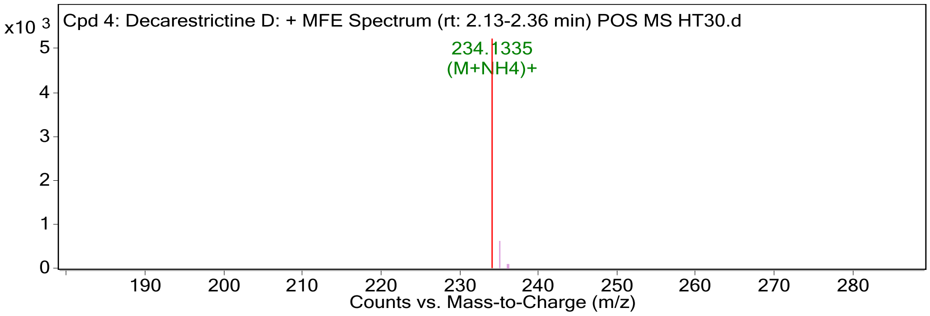

Compound Label	Name	*m/z*	RT	Algorithm	Mass
Cpd 5: Terrein	**Terrein**	172.0968	2.74	Find by Molecular Feature	154.0629

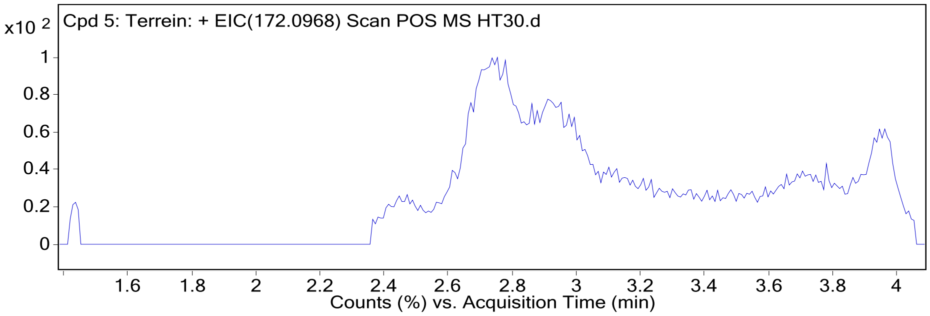

MFE MS Zoomed Spectrum

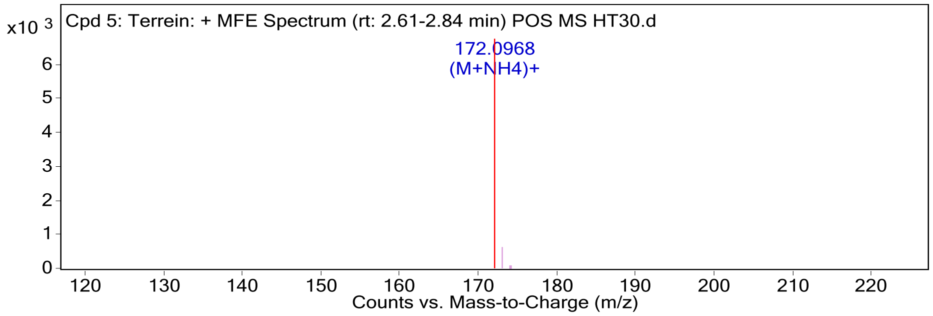

Compound Label	Name	*m/z*	RT	Algorithm	Mass
Cpd 6: Terrein	**Terrein**	172.0967	3.95	Find by Molecular Feature	154.0629

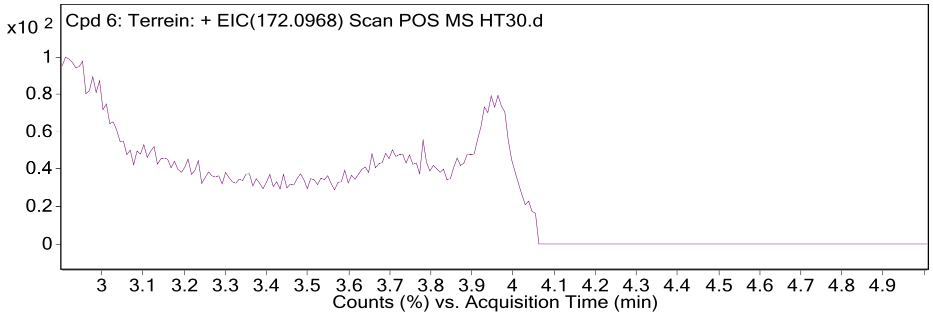

MFE MS Zoomed Spectrum

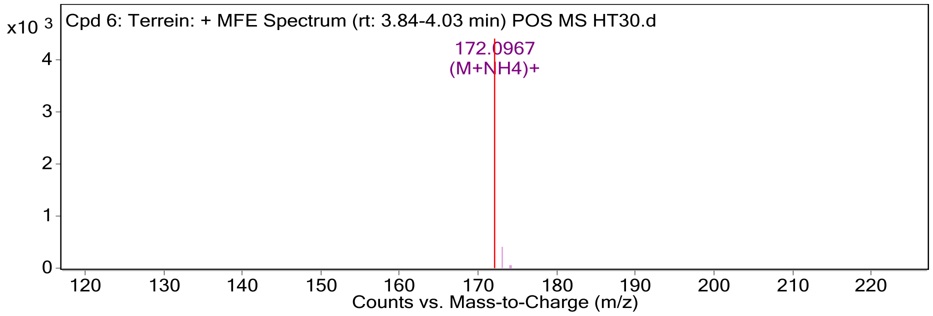

Compound Label	Name	*m/z*	RT	Algorithm	Mass
Cpd 7: Fusaric acid	**Fusaric acid**	197.1287	4.87	Find by Molecular Feature	179.0949

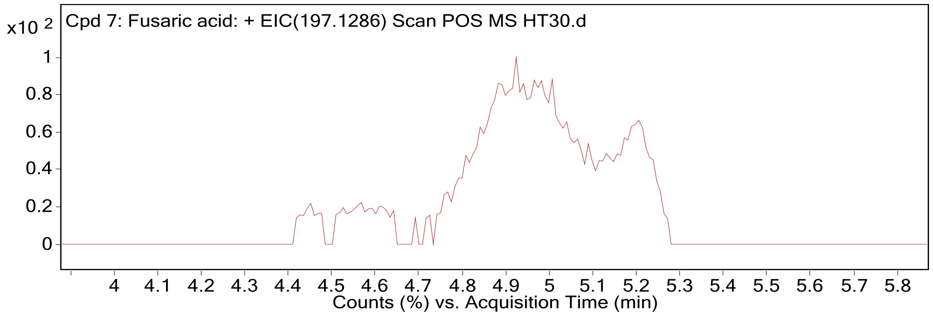

MFE MS Zoomed Spectrum

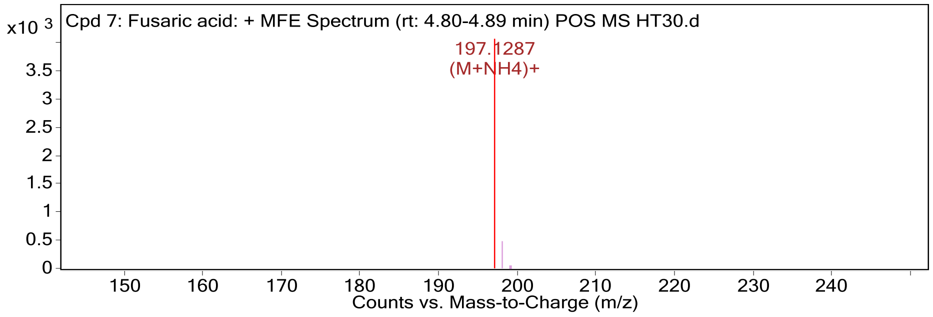

**Compound Label**	**Name**	***m/z***	**RT**	**Algorithm**	**Mass**
Cpd 8: Fusaric acid	**Fusaric acid**	197.1285	4.99	Find by Molecular Feature	179.0947

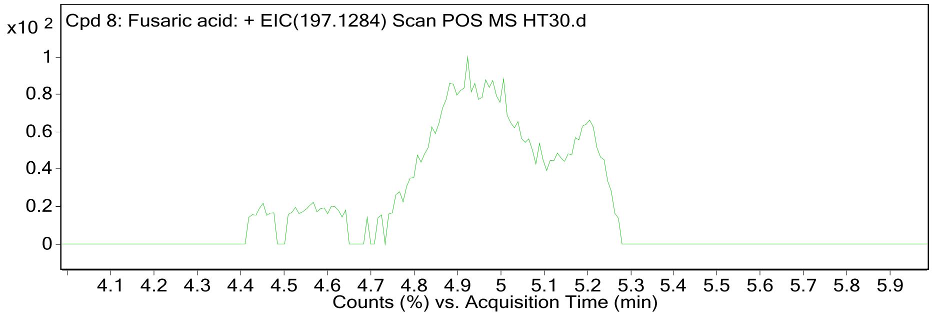

MFE MS Zoomed Spectrum

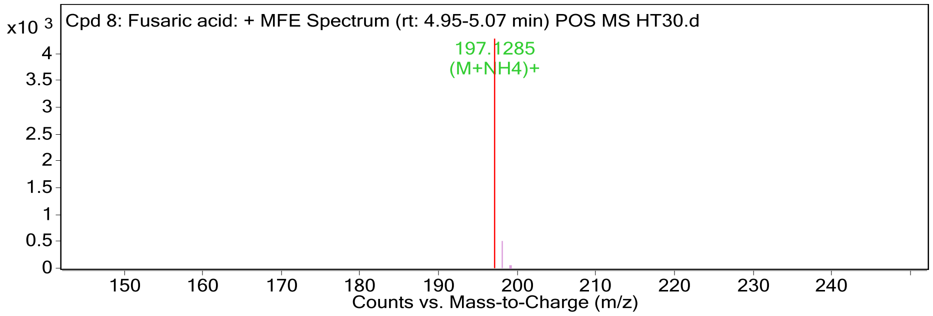

**Compound Label**	**Name**	***m/z***	**RT**	**Algorithm**	**Mass**
Cpd 9: OTB / Ochratoxin B	**OTB / Ochratoxin B**	387.1552	4.99	Find by Molecular Feature	369.1214

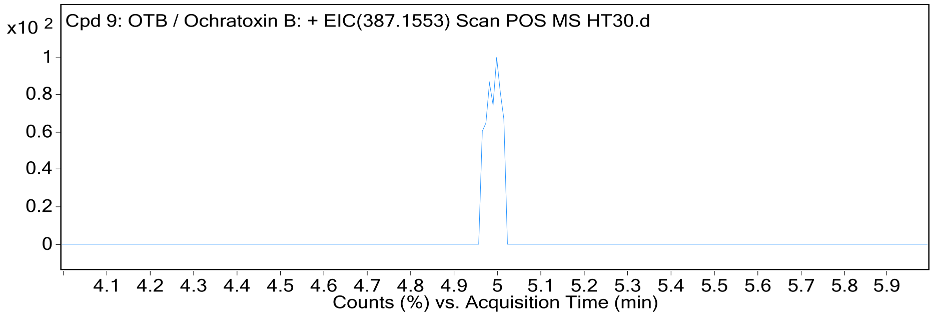

MFE MS Zoomed Spectrum

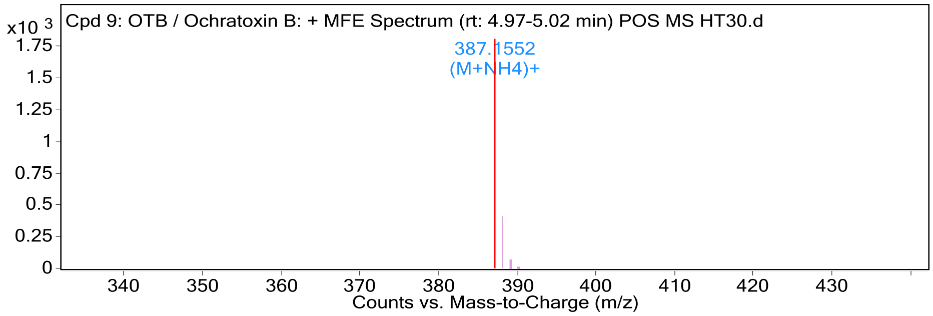

Compound Label	Name	*m/z*	RT	Algorithm	Mass
Cpd 10: Cerulenin	**Cerulenin**	241.1547	5.17	Find by Molecular Feature	223.1209

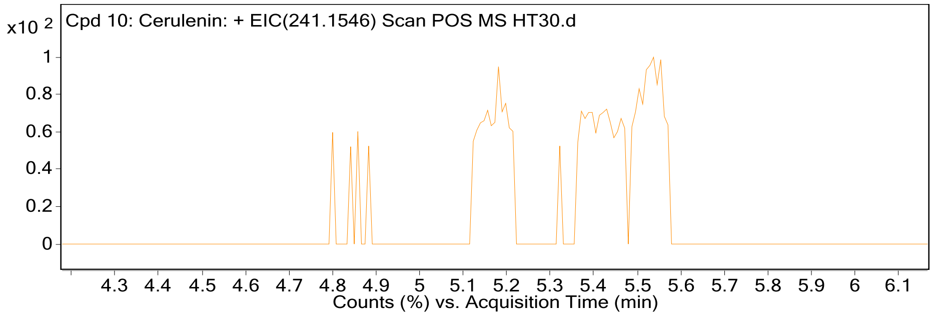

MFE MS Zoomed Spectrum

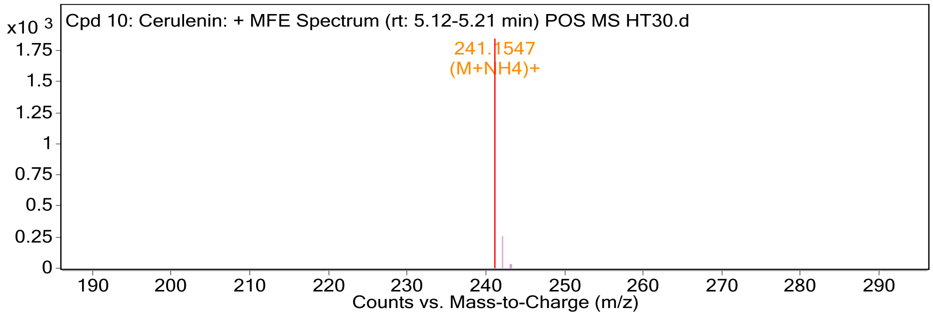

Compound Label	Name	*m/z*	RT	Algorithm	Mass
Cpd 11: Fusaric acid	**Fusaric acid**	197.1286	5.2	Find by Molecular Feature	179.0948

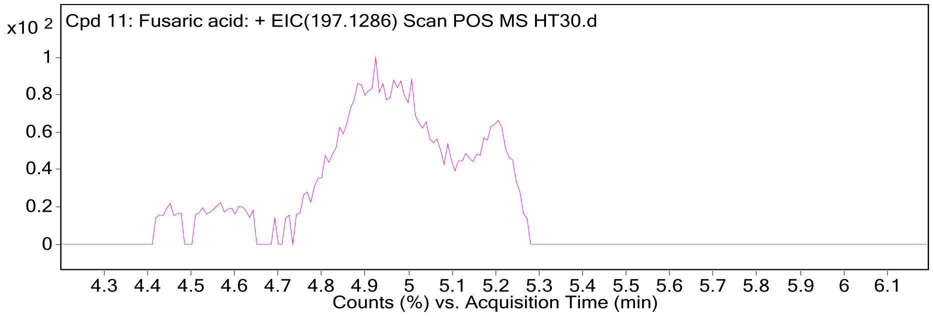

MFE MS Zoomed Spectrum

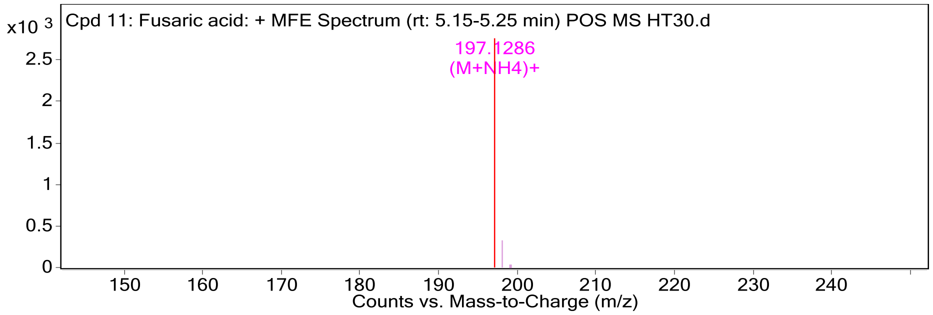

Compound Label	Name	*m/z*	RT	Algorithm	Mass
Cpd 12: Oosporein	**Oosporein**	329.0268	5.22	Find by Molecular Feature	306.0376

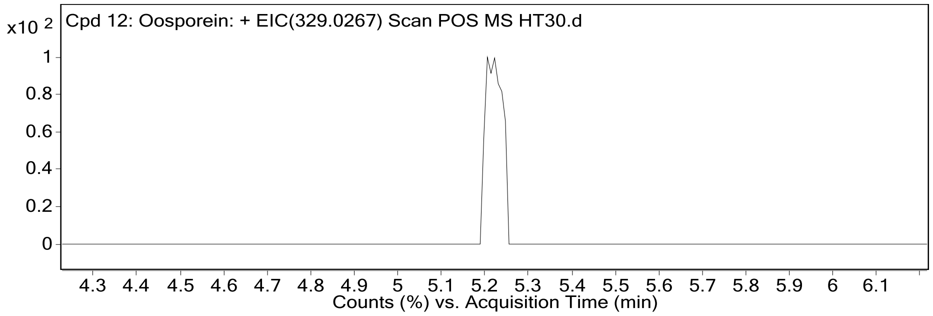

MFE MS Zoomed Spectrum

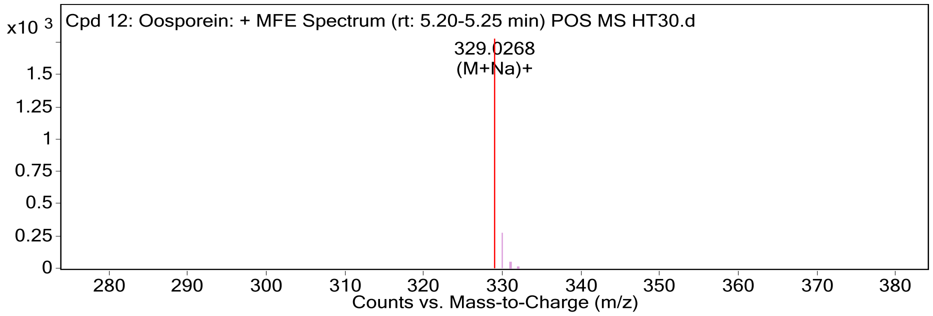

Compound Label	Name	*m/z*	RT	Algorithm	Mass

Cpd 13: Oosporein	**Oosporein**	307.045	5.22	Find by Molecular Feature	306.0377

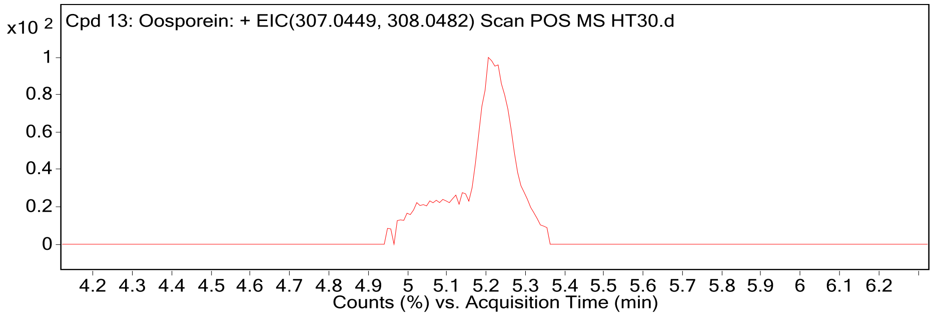

MFE MS Zoomed Spectrum

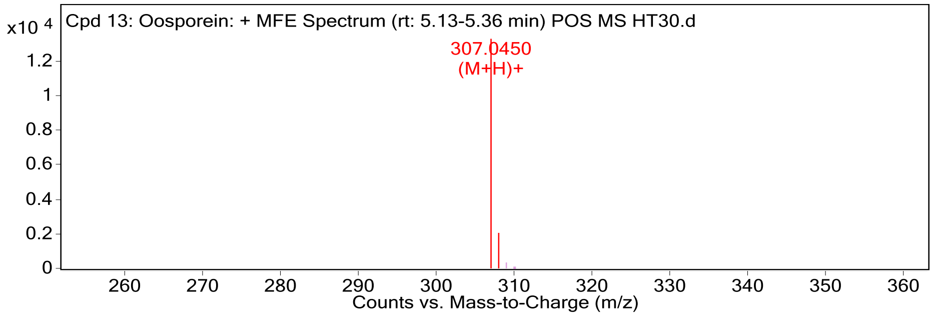

Compound Label	Name	*m/z*	RT	Algorithm	Mass
Cpd 14: Cerulenin	**Cerulenin**	241.1548	5.53	Find by Molecular Feature	223.121

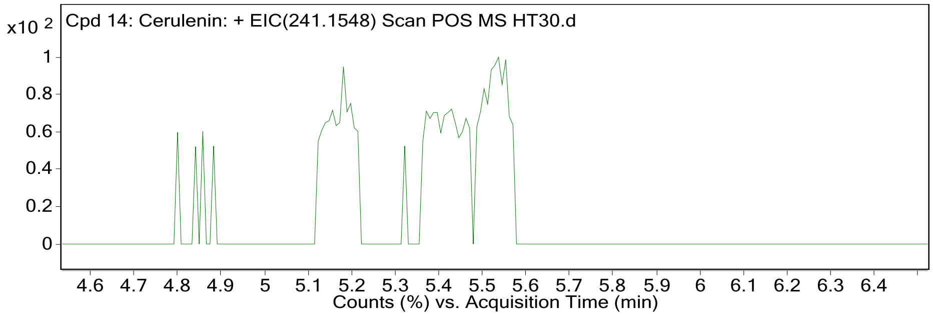

MFE MS Zoomed Spectrum

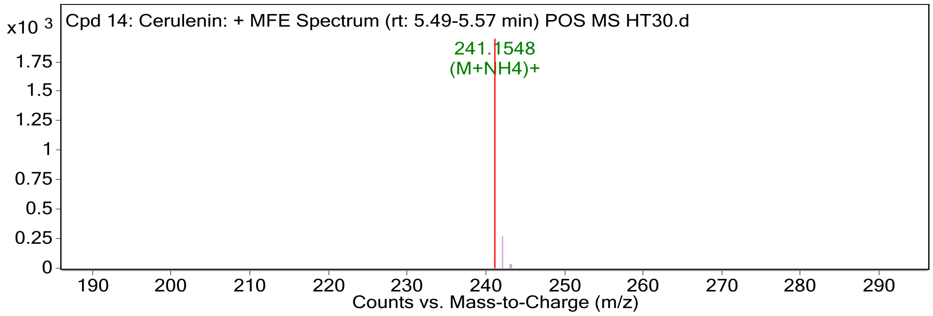

Compound Label	Name	*m/z*	RT	Algorithm	Mass
Cpd 15: Antimycin	**Antimycin**	251.0915	6.11	Find by Molecular Feature	250.0842

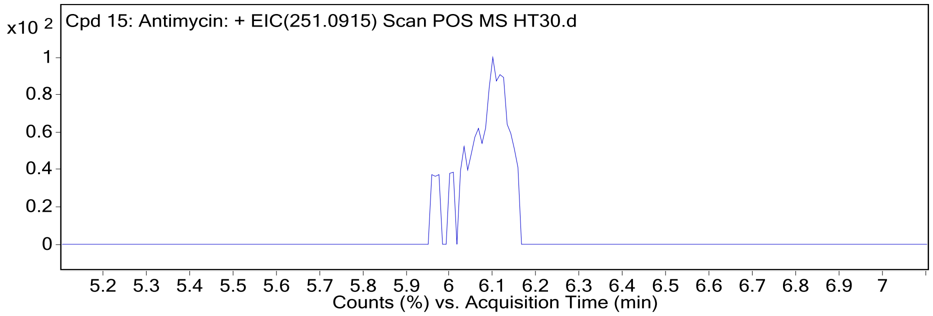

MFE MS Zoomed Spectrum

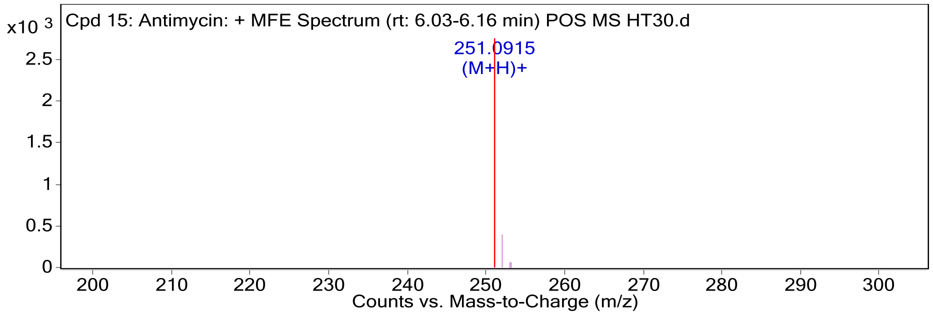

Compound Label	Name	*m/z*	RT	Algorithm	Mass
Cpd 16: Fusaric acid	**Fusaric acid**	180.102	6.24	Find by Molecular Feature	179.0947

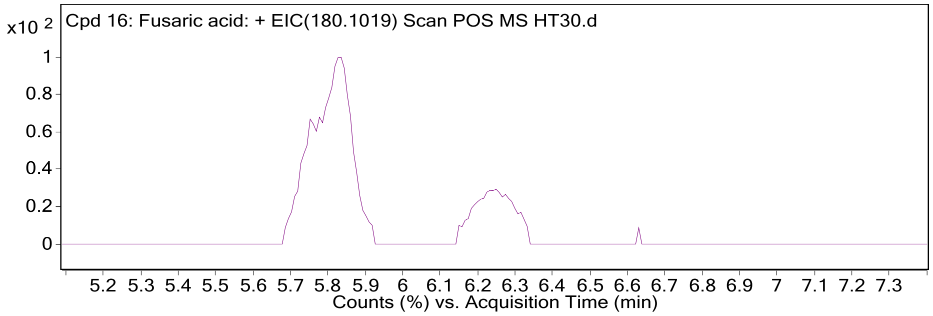

MFE MS Zoomed Spectrum

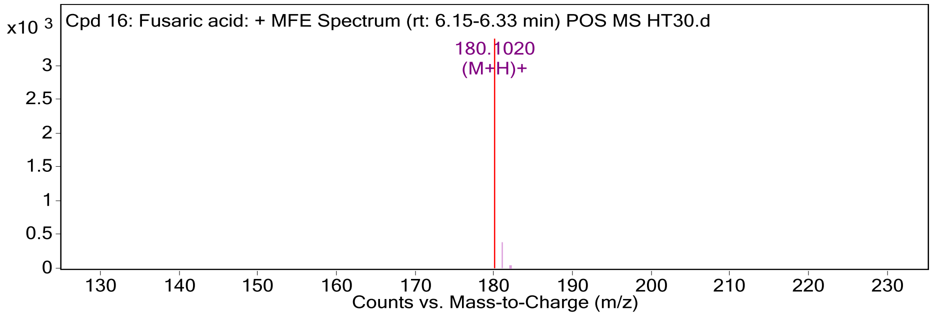

Qualitative Compound Report

Compound Label	Name	*m/z*	RT	Algorithm	Mass
Cpd 17: Pyrenocine A	**Pyrenocine A**	226.1075	6.4	Find by Molecular Feature	208.0736

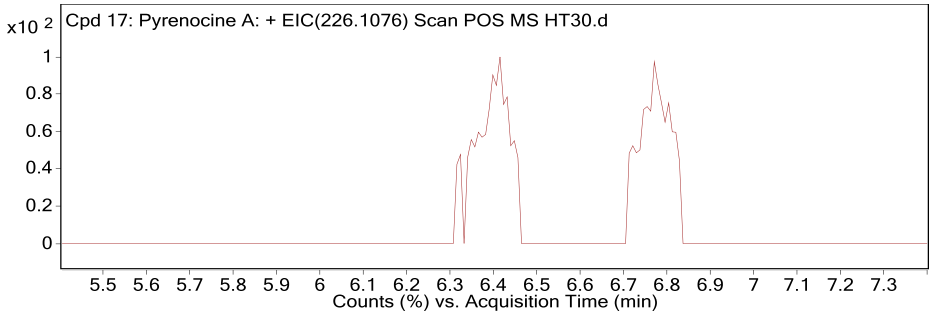

MFE MS Zoomed Spectrum

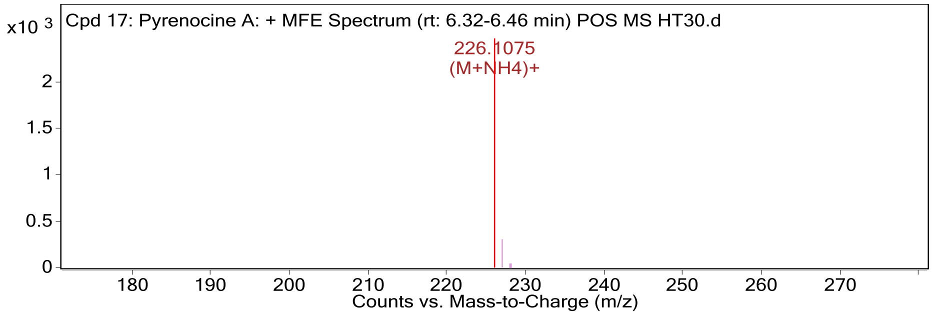

Compound Label	Name	*m/z*	RT	Algorithm	Mass
Cpd 18: Brevianamid F	**Brevianamid F**	284.1398	6.55	Find by Molecular Feature	283.1325

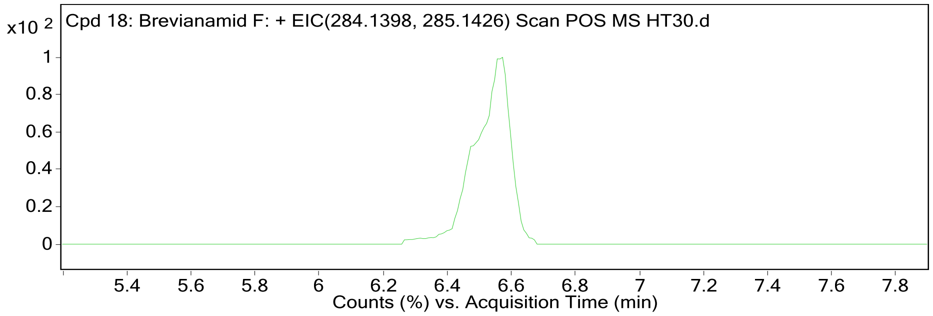

MFE MS Zoomed Spectrum

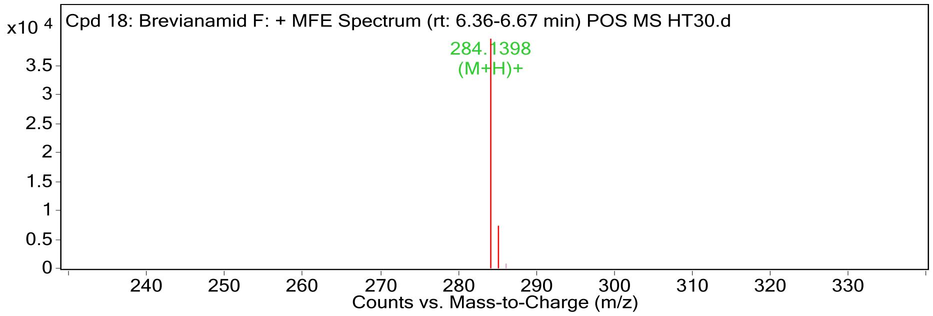

Compound Label	Name	*m/z*	RT	Algorithm	Mass
Cpd 19: Paspalic acid	**Paspalic acid**	286.155	6.67	Find by Molecular Feature	268.1211

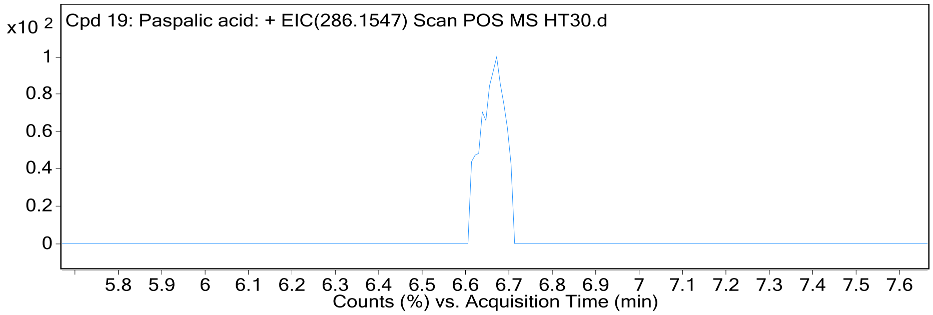

MFE MS Zoomed Spectrum

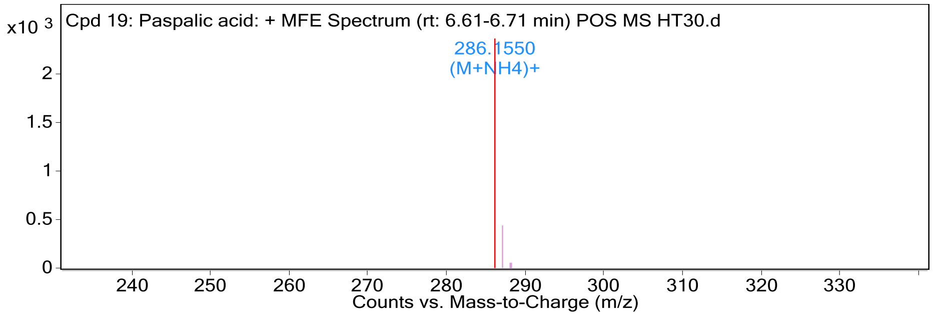

Compound Label	Name	*m/z*	RT	Algorithm	Mass
Cpd 20: Pyrenocine A	**Pyrenocine A**	226.1074	6.78	Find by Molecular Feature	208.0736

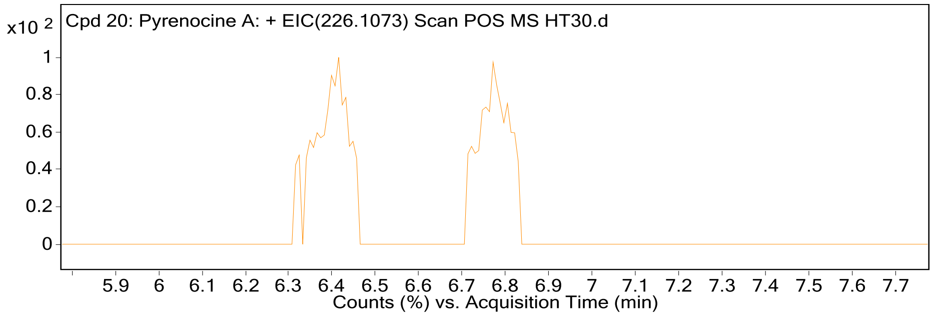

MFE MS Zoomed Spectrum

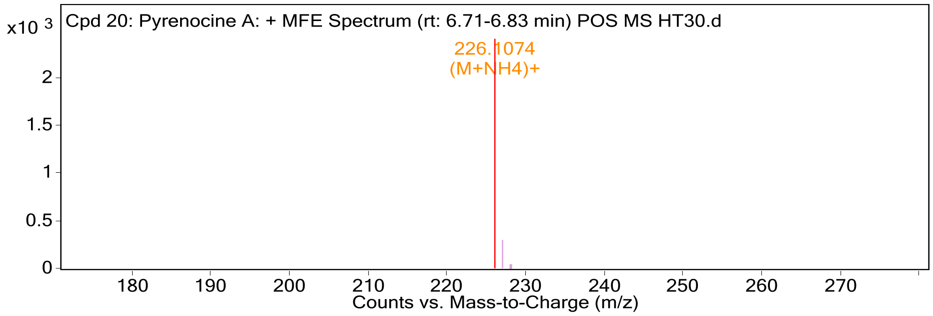

Qualitative Compound Report

Compound Label	Name	*m/z*	RT	Algorithm	Mass
Cpd 21: Pestalotin	**Pestalotin**	232.1542	6.78	Find by Molecular Feature	214.1204

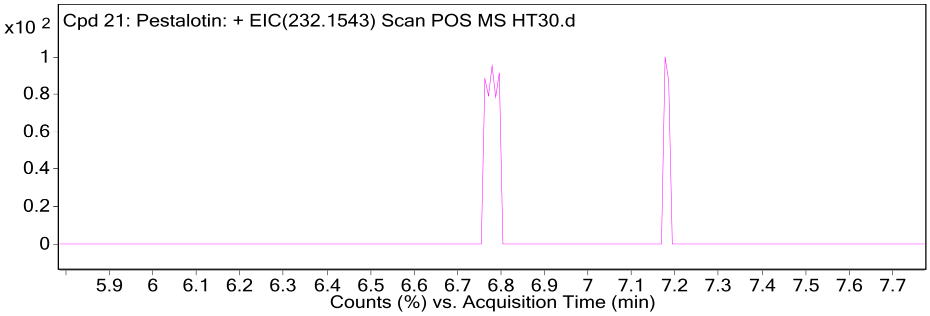

MFE MS Zoomed Spectrum

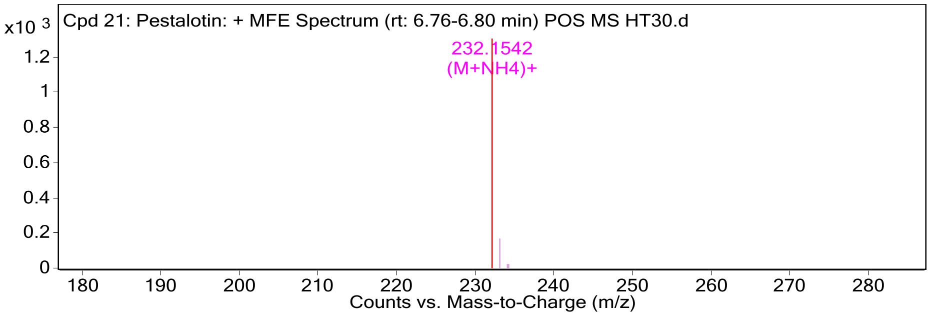

Compound Label	Name	*m/z*	RT	Algorithm	Mass
Cpd 22: beta-Zearalenol	**beta-Zearalenol**	338.1959	7.14	Find by Molecular Feature	320.162

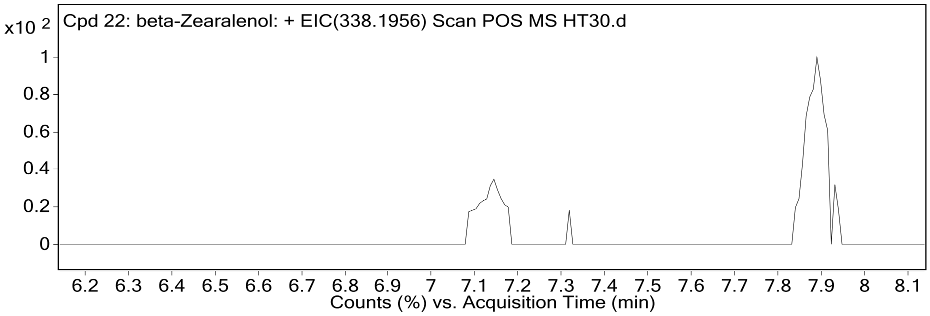

MFE MS Zoomed Spectrum

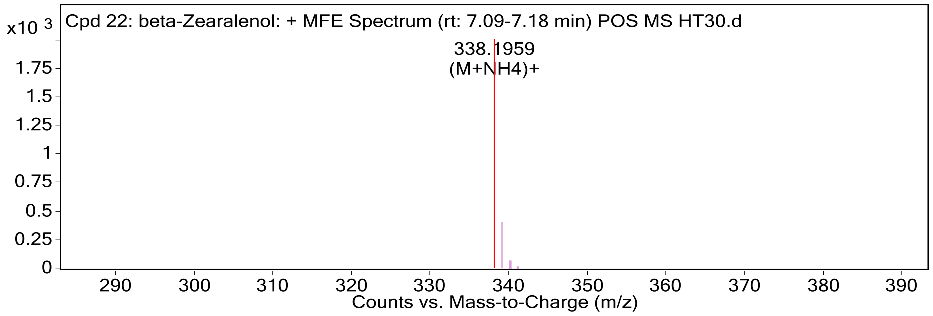

Compound Label	Name	*m/z*	RT	Algorithm	Mass
Cpd 23: Macrosporin	**Macrosporin**	307.0565	7.14	Find by Molecular Feature	284.0673

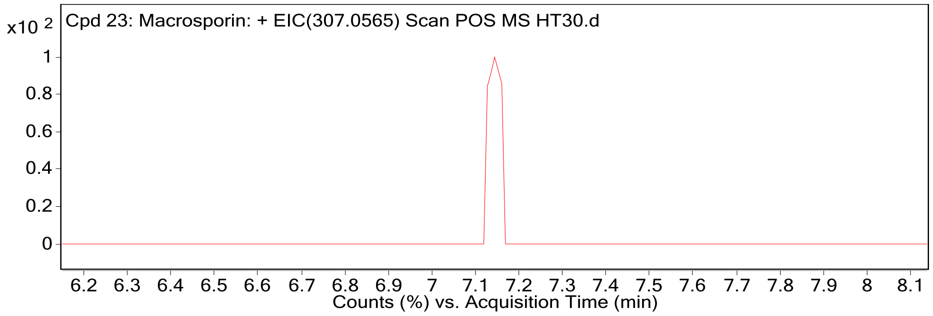

MFE MS Zoomed Spectrum

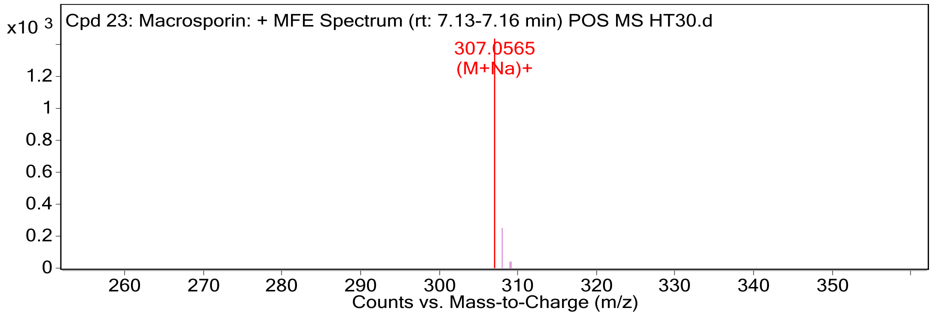

Compound Label	Name	*m/z*	RT	Algorithm	Mass
Cpd 24: Aphidicolin	**Aphidicolin**	377.2072	7.15	Find by Molecular Feature	338.2438

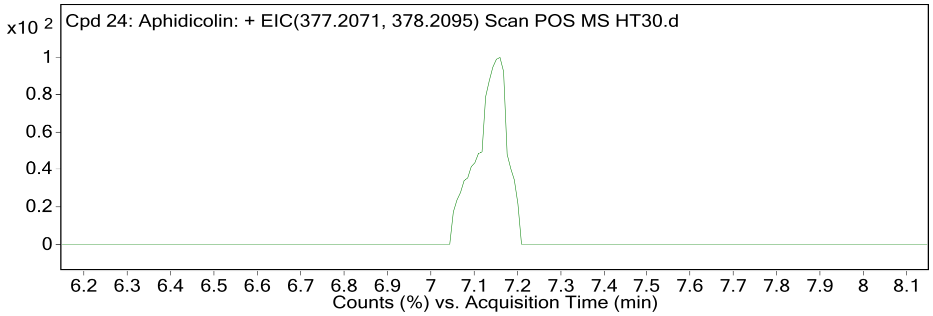

MFE MS Zoomed Spectrum

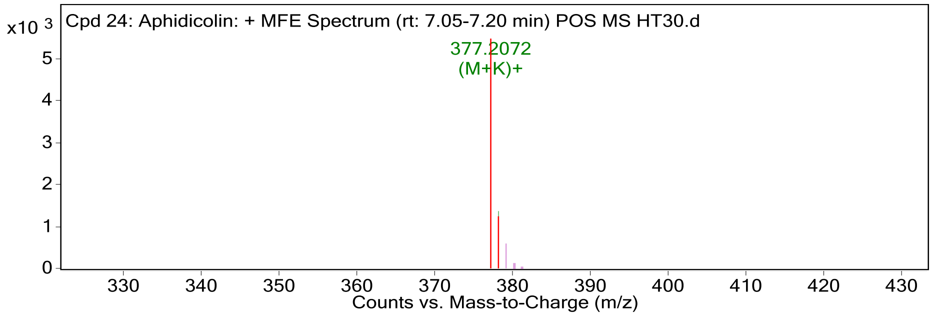

Compound Label	Name	*m/z*	RT	Algorithm	Mass
Cpd 25: DAS / Diacetoxyscirpenol	**DAS / Diacetoxyscirpenol**	384.2016	7.41	Find by Molecular Feature	366.1677

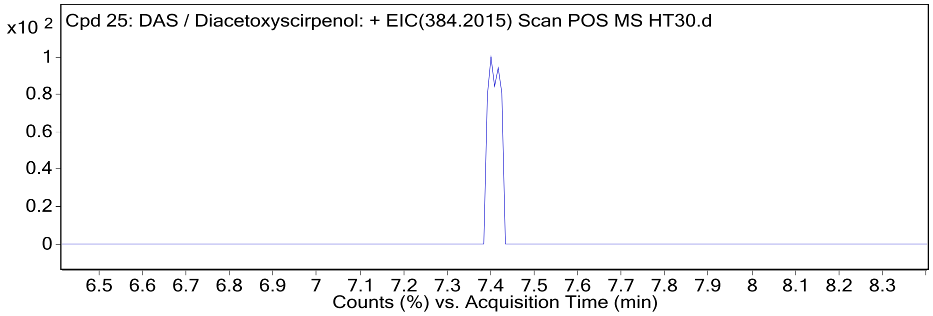

MFE MS Zoomed Spectrum

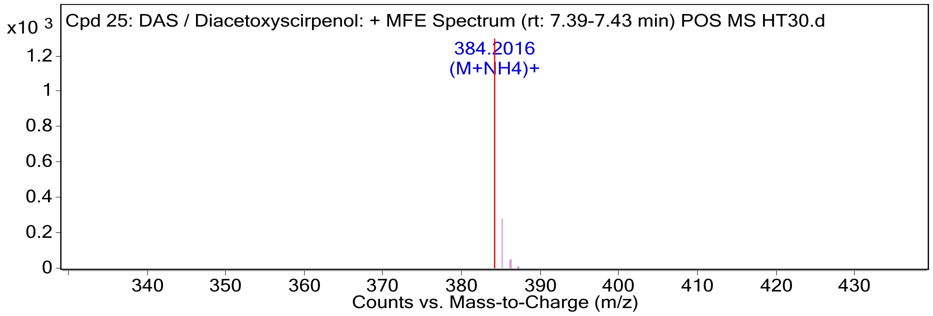

Compound Label	Name	*m/z*	RT	Algorithm	Mass
Cpd 26: Rugulosuvine	**Rugulosuvine**	334.1551	7.42	Find by Molecular Feature	333.1478

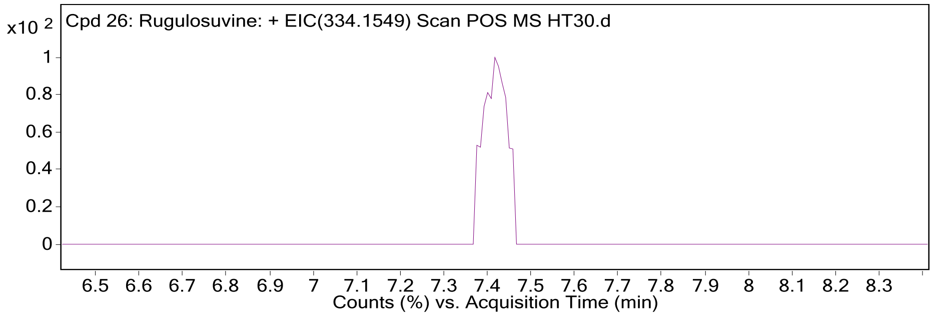

MFE MS Zoomed Spectrum

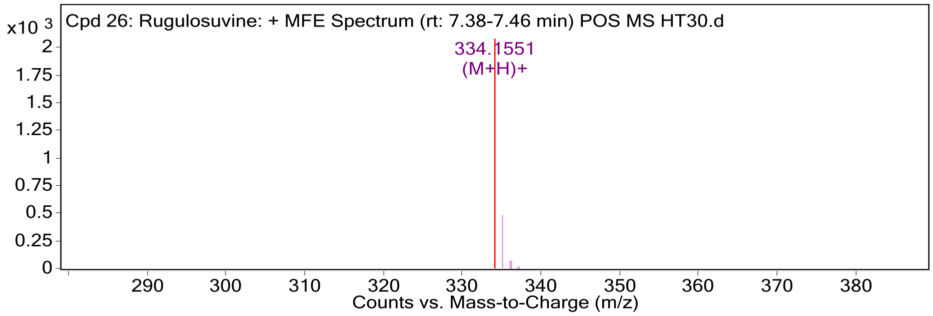

Compound Label	Name	*m/z*	RT	Algorithm	Mass
Cpd 27: Cladosporin	**Cladosporin**	310.1647	7.45	Find by Molecular Feature	292.1309

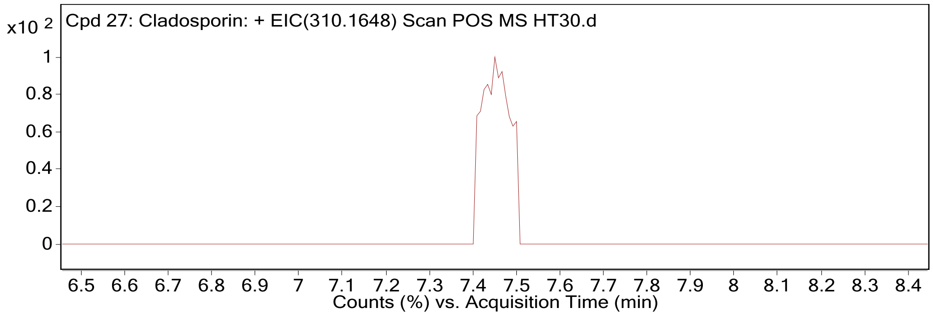

MFE MS Zoomed Spectrum

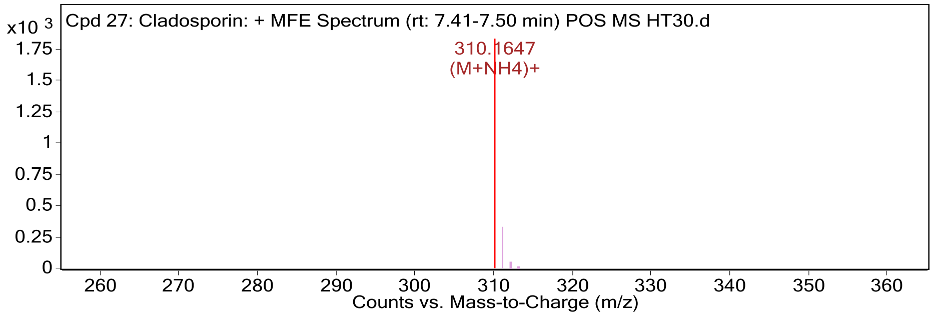

Compound Label	Name	*m/z*	RT	Algorithm	Mass
Cpd 28: Fusaric acid	**Fusaric acid**	180.102	7.82	Find by Molecular Feature	179.0948

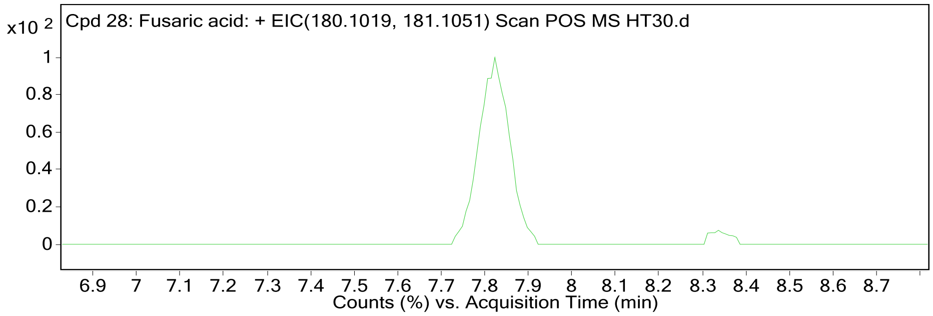

MFE MS Zoomed Spectrum

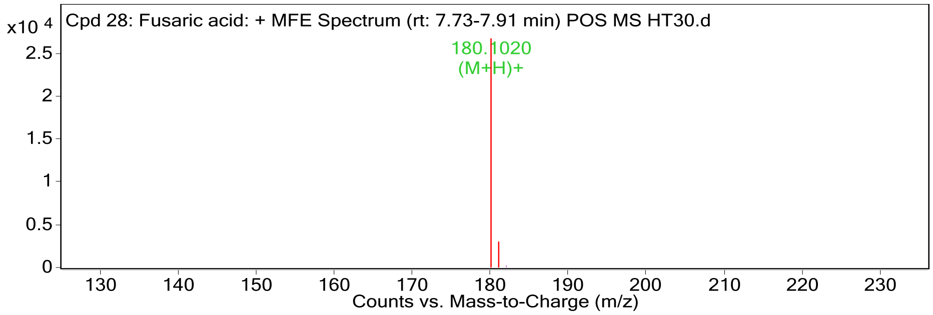

Compound Label	Name	*m/z*	RT	Algorithm	Mass
Cpd 29: beta-Zearalenol	**beta-Zearalenol**	338.1962	7.89	Find by Molecular Feature	320.1624

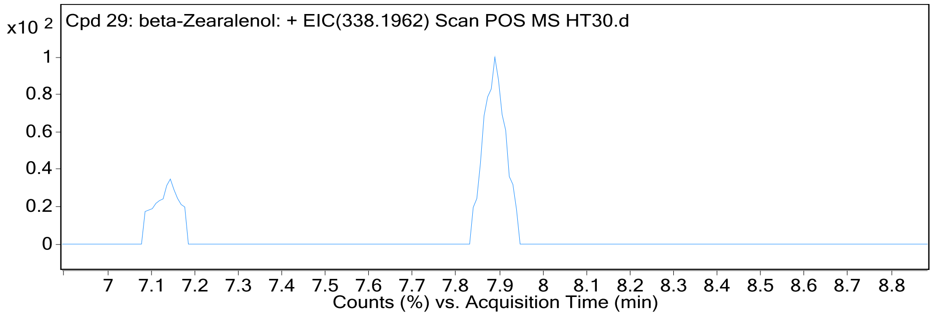

MFE MS Zoomed Spectrum

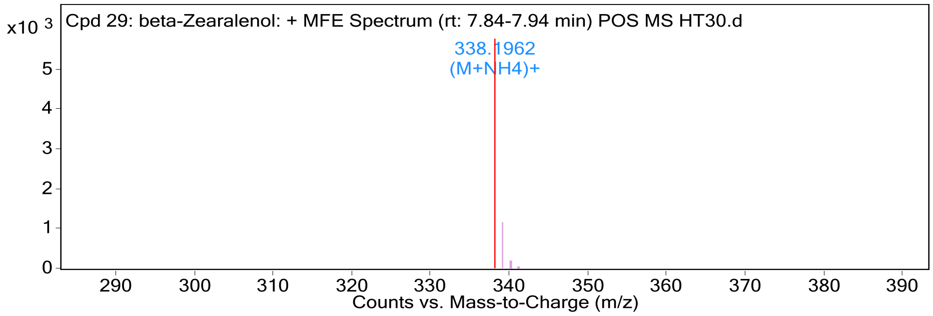

Compound Label	Name	*m/z*	RT	Algorithm	Mass

Cpd 30: Culmorin	**Culmorin**	256.2272	8.46	Find by Molecular Feature	238.1934

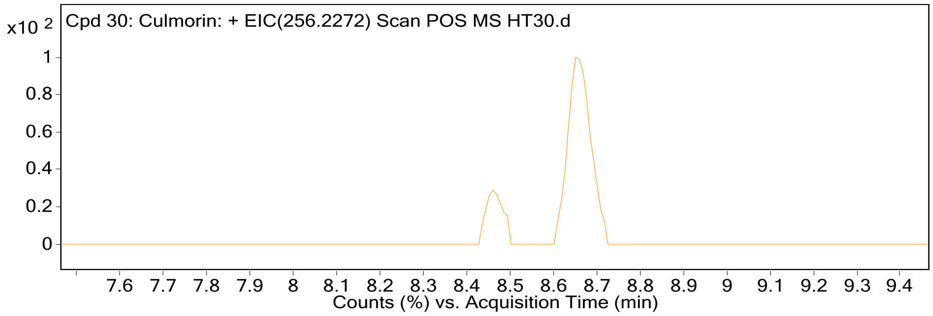

MFE MS Zoomed Spectrum

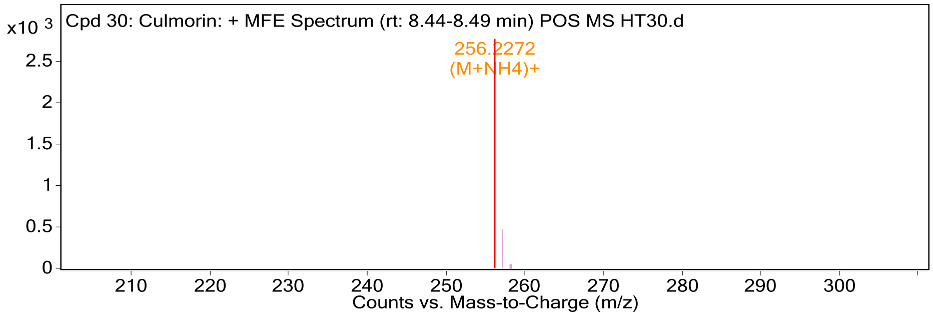

Compound Label	Name	*m/z*	RT	Algorithm	Mass
Cpd 31: Siccanol	**Siccanol**	441.2391	8.53	Find by Molecular Feature	402.2759

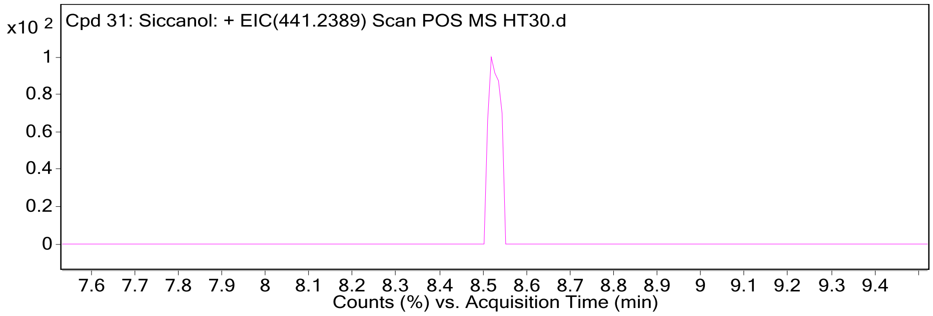

MFE MS Zoomed Spectrum

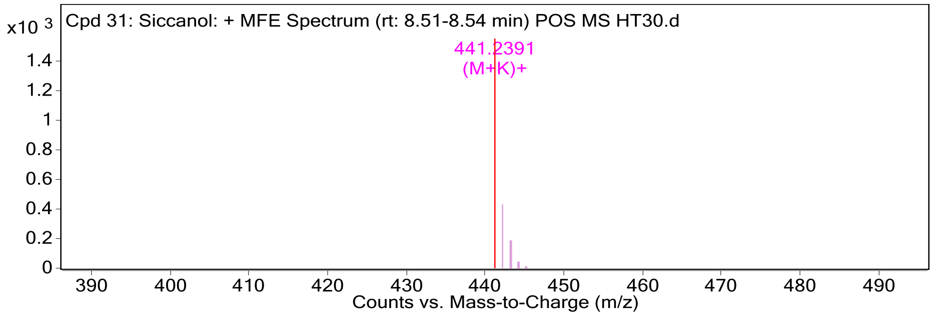

Compound Label	Name	*m/z*	RT	Algorithm	Mass
Cpd 32: 15-Hydroxyculmorone	**15-Hydroxyculmorone**	270.2067	8.63	Find by Molecular Feature	252.1728

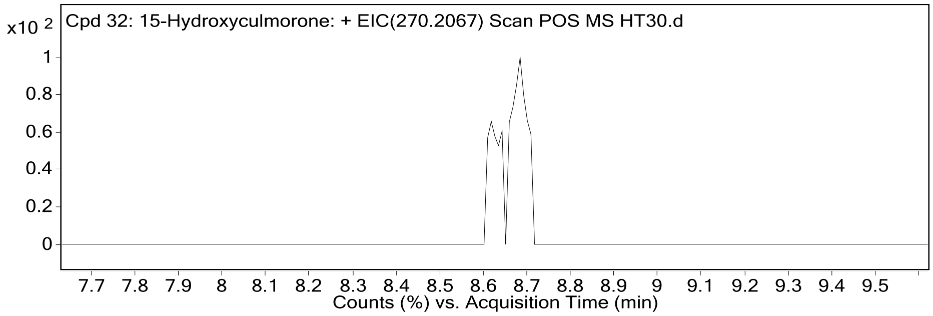

MFE MS Zoomed Spectrum

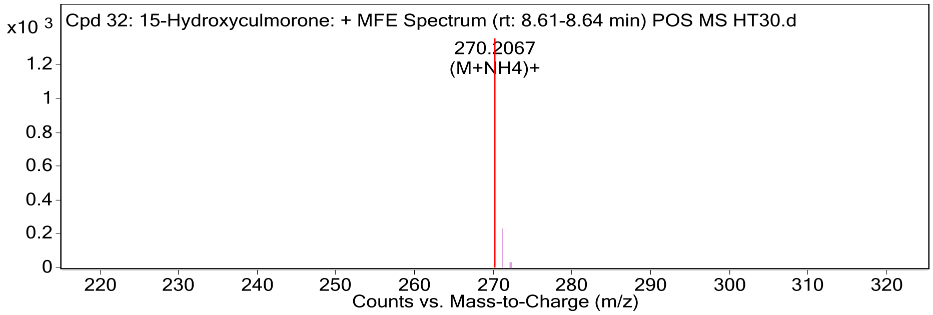

Compound Label	Name	*m/z*	RT	Algorithm	Mass
Cpd 33: Culmorin	**Culmorin**	256.2272	8.66	Find by Molecular Feature	238.1933

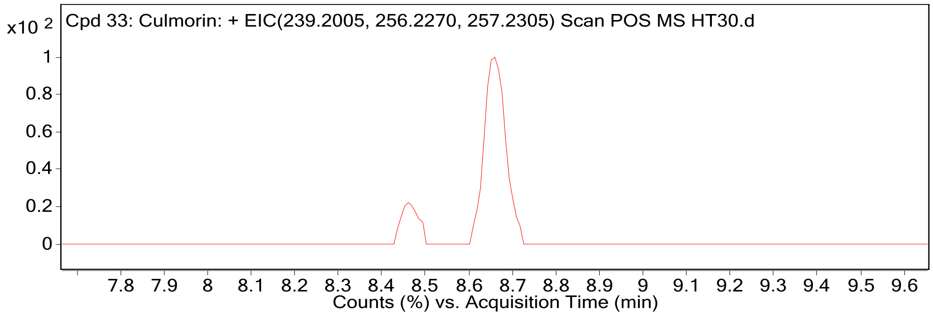

MFE MS Zoomed Spectrum

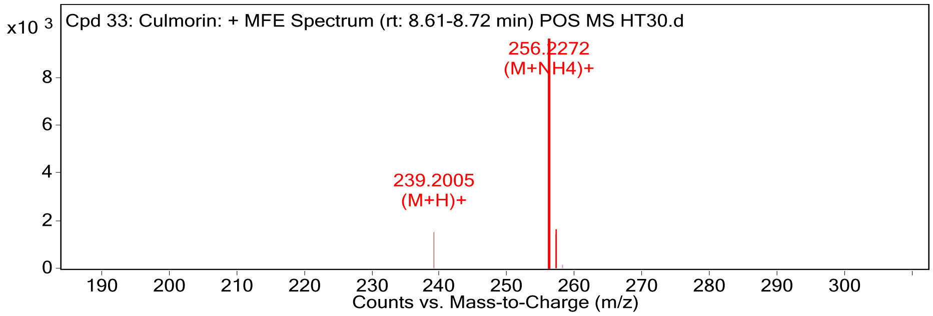

Compound Label	Name	*m/z*	RT	Algorithm	Mass
Cpd 34: 15-Hydroxyculmorone	**15-Hydroxyculmorone**	270.2063	8.68	Find by Molecular Feature	252.1724

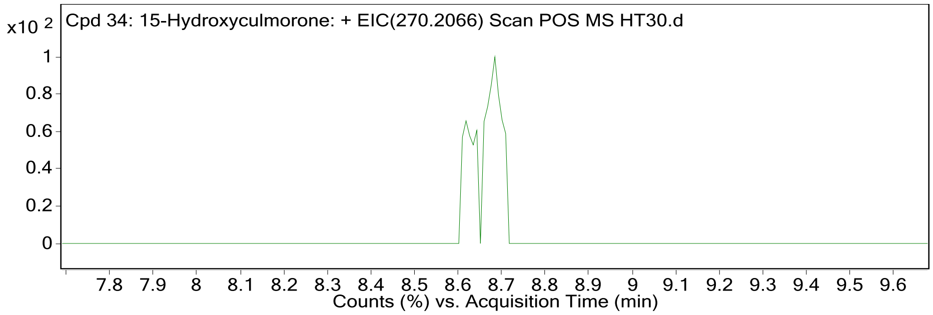

MFE MS Zoomed Spectrum

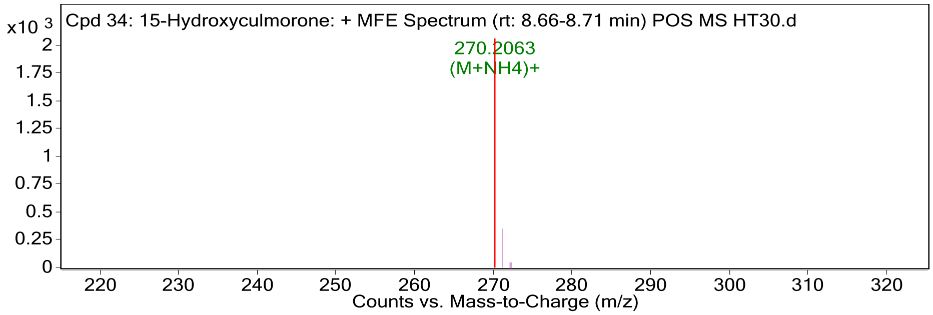

Compound Label	Name	*m/z*	RT	Algorithm	Mass
Cpd 35: Deepoxy deoxynivalenol	**Deepoxy deoxynivalenol**	281.1377	9.11	Find by Molecular Feature	280.1304

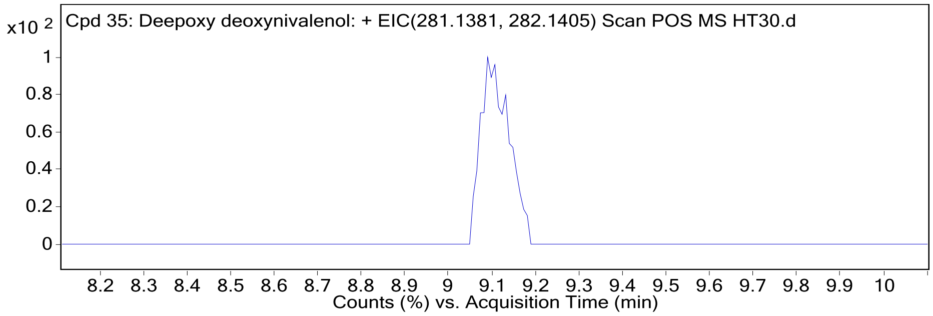

MFE MS Zoomed Spectrum

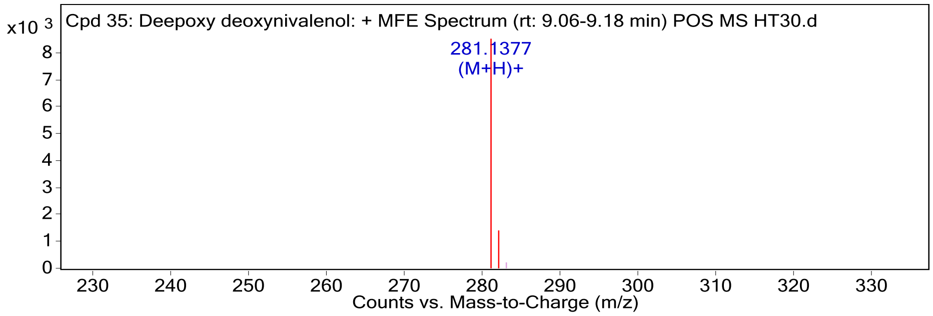

Compound Label	Name	*m/z*	RT	Algorithm	Mass
Cpd 36: Cytochalasin E	**Cytochalasin E**	513.2594	9.29	Find by Molecular Feature	495.2256

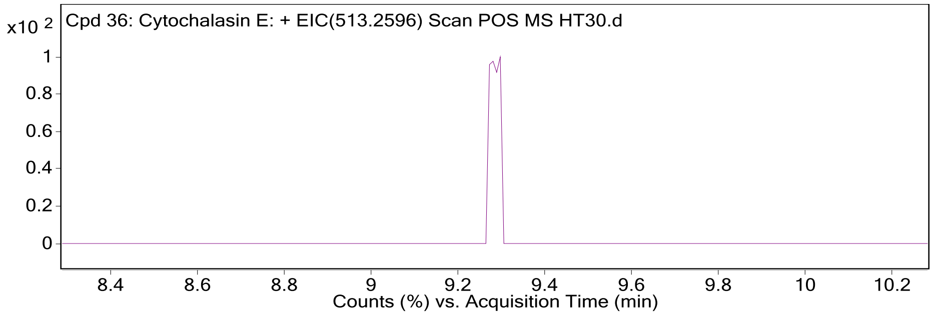

MFE MS Zoomed Spectrum

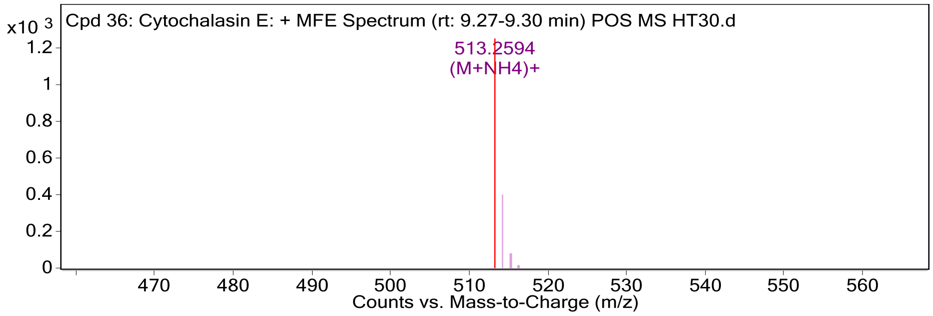

Compound Label	Name	*m/z*	RT	Algorithm	Mass
Cpd 37: Fusaproliferin	**Fusaproliferin**	483.2494	9.43	Find by Molecular Feature	444.2863

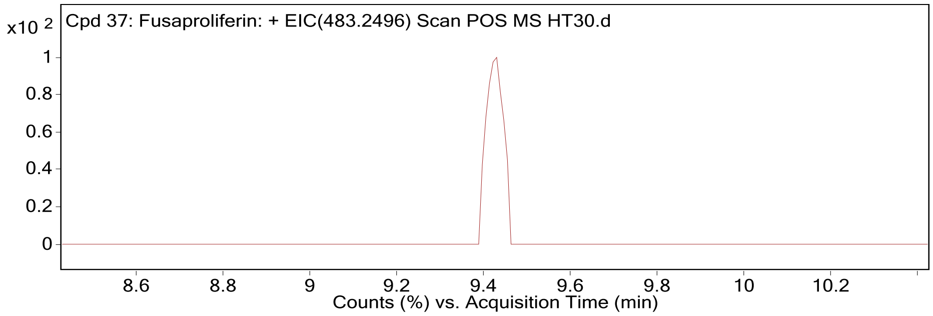

MFE MS Zoomed Spectrum

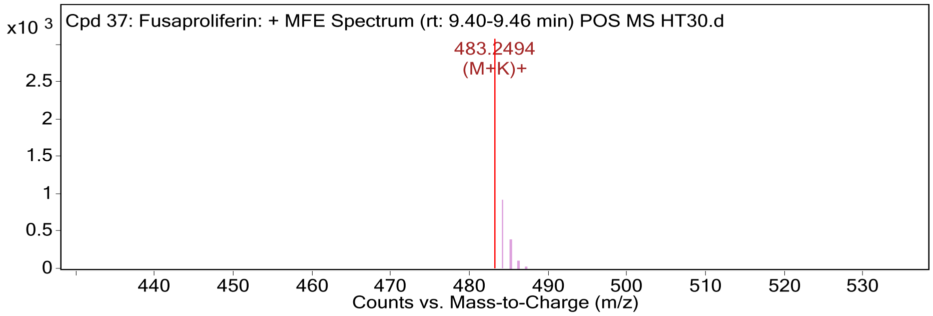

Compound Label	Name	*m/z*	RT	Algorithm	Mass
Cpd 38: 5-Methoxysterigmatocystin	**5-Methoxysterigmatocys**	393.038	9.73	Find by Molecular Feature	354.0749

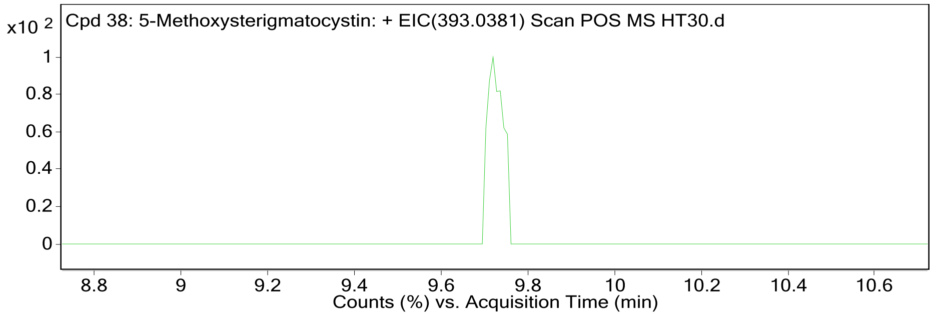

MFE MS Zoomed Spectrum

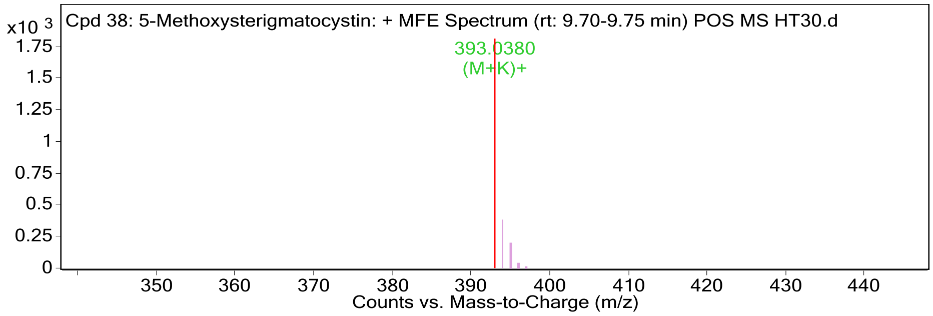

Compound Label	Name	*m/z*	RT	Algorithm	Mass
Cpd 39: Paspaline	**Paspaline**	439.3313	9.98	Find by Molecular Feature	421.2973

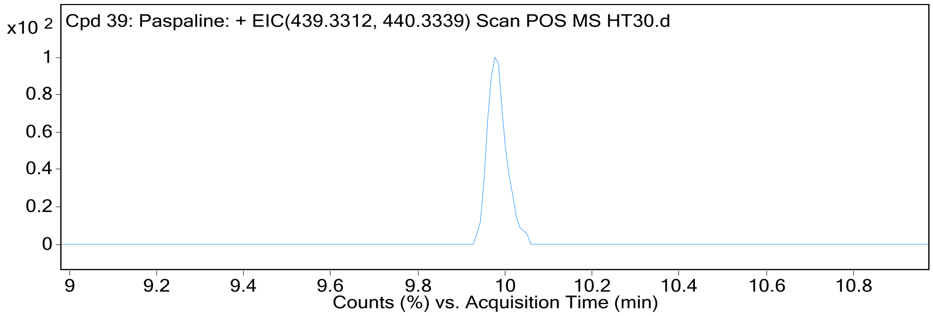

MFE MS Zoomed Spectrum

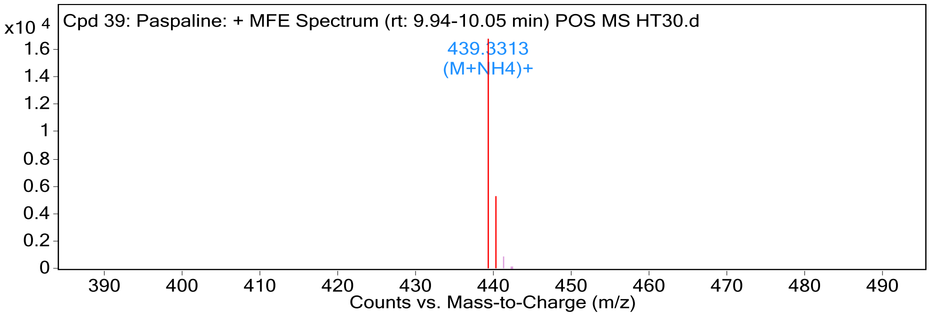

Compound Label	Name	*m/z*	RT	Algorithm	Mass
Cpd 40: Aphidicolin	**Aphidicolin**	356.2786	10.2	Find by Molecular Feature	338.2447

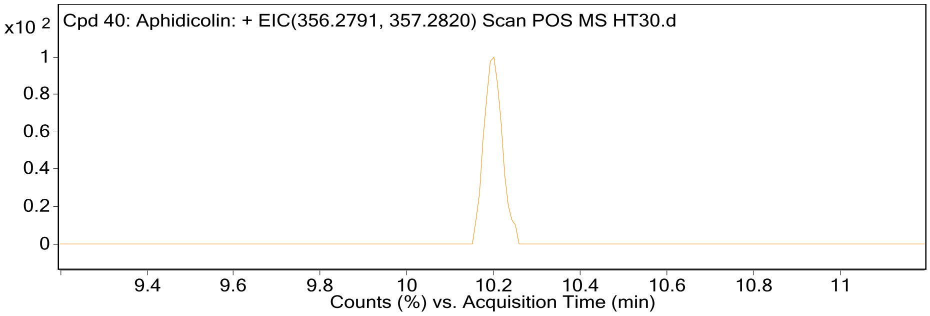

MFE MS Zoomed Spectrum

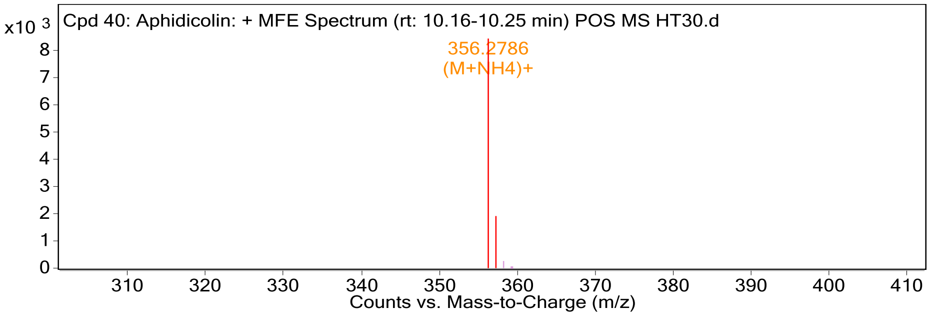

Compound Label	Name	*m/z*	RT	Algorithm	Mass
Cpd 41: Enniatin A2	**Enniatin A2**	704.4424	10.99	Find by Molecular Feature	681.453

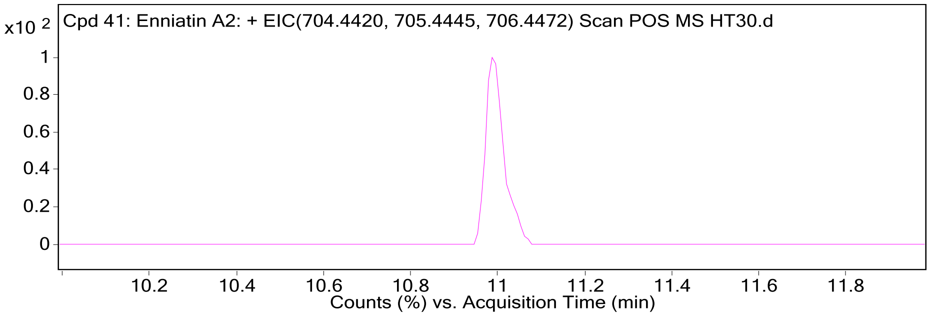

MFE MS Zoomed Spectrum

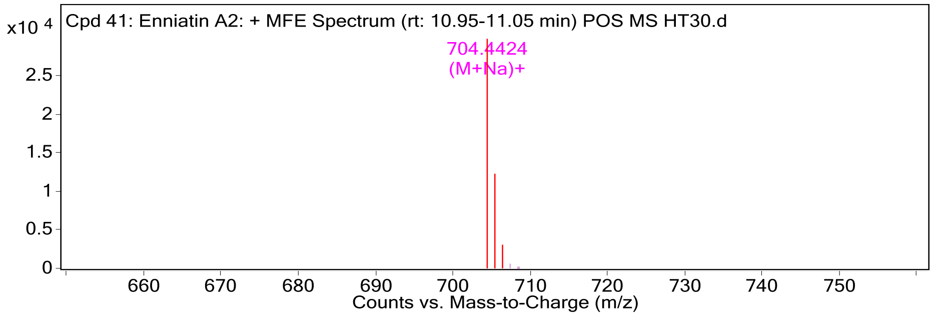

Compound Label	Name	*m/z*	RT	Algorithm	Mass
Cpd 42: Enniatin A2	**Enniatin A2**	699.487	11	Find by Molecular Feature	681.4532

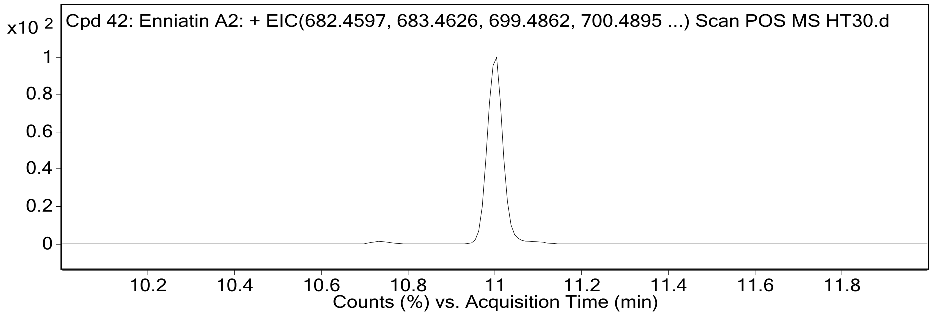

MFE MS Zoomed Spectrum

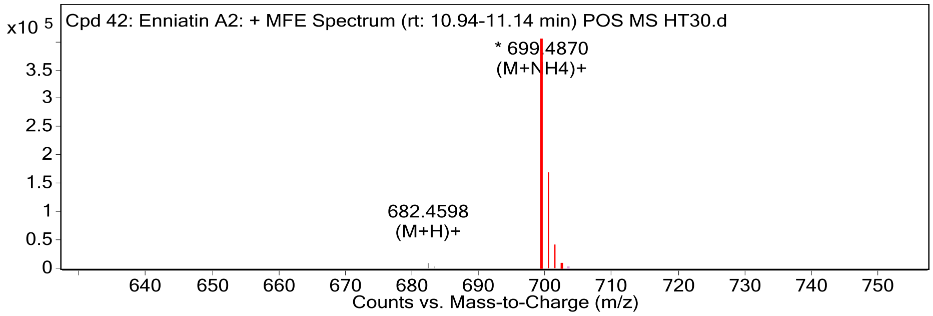

Compound Label	Name	*m/z*	RT	Algorithm	Mass
Cpd 43: Beauvericin	**Beauvericin**	801.4397	11.06	Find by Molecular Feature	783.4059

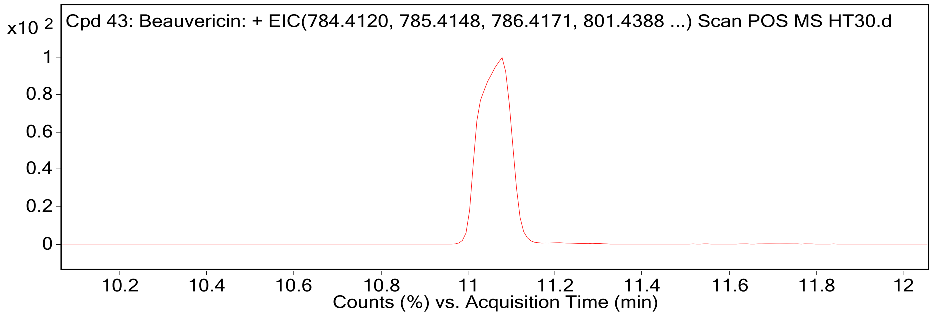

MFE MS Zoomed Spectrum

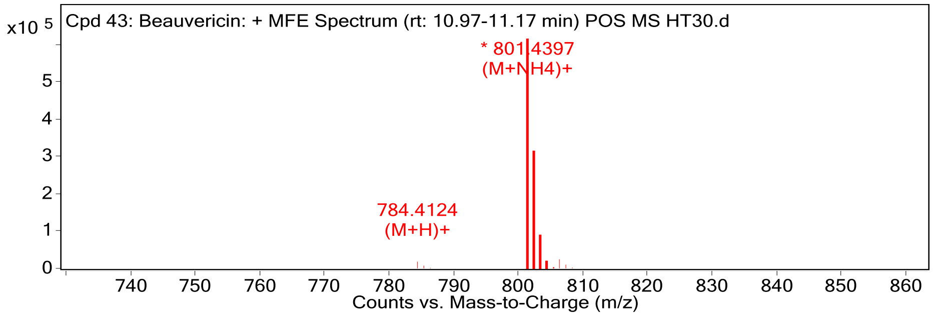

Compound Label	Name	*m/z*	RT	Algorithm	Mass
Cpd 44: Sirolimus (Rapamycin)	**Sirolimus (Rapamycin)**	931.5919	11.58	Find by Molecular Feature	913.5581

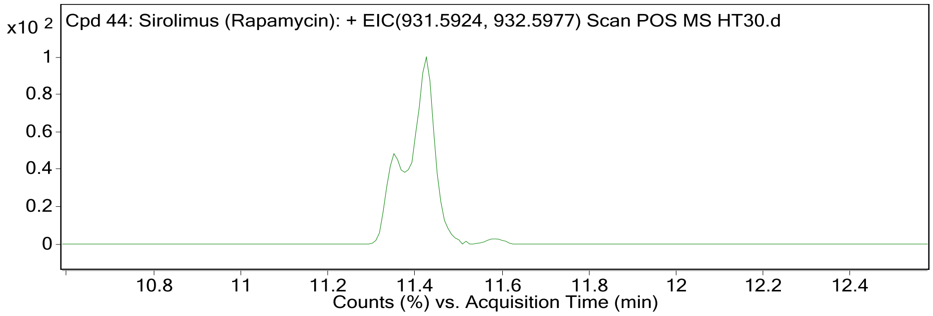

MFE MS Zoomed Spectrum

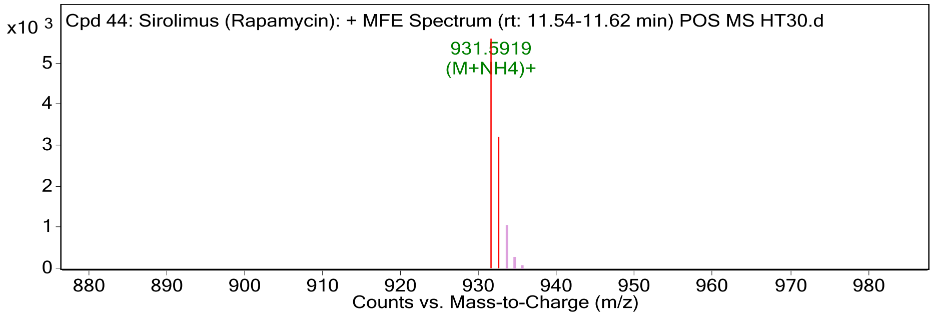

--- End Of Report ---

Qualitative Compound Report

**Data File**	POS MS HT98.d	**Sample Name**	HT98
**Sample Type**	Sample	**Position**	P1-A3
**Instrument Name**	Instrument 1	**User Name**	
**Acq Method**	Mycotoxins POS.m	**Acquired Time**	6/15/2022 11:41:44 AM
**IRM Calibration Status**	Success	**DA Method**	scau default.m
**Comment**			

**Sample Group**		**Info.**	
**Stream Name**	LC 1	**Acquisition SW Version**	6200 series TOF/6500 series Q-TOF B.06.01 (B6157)

Fragmentor Voltage 130 **Collision Energy** 0 **Ionization Mode** ESI

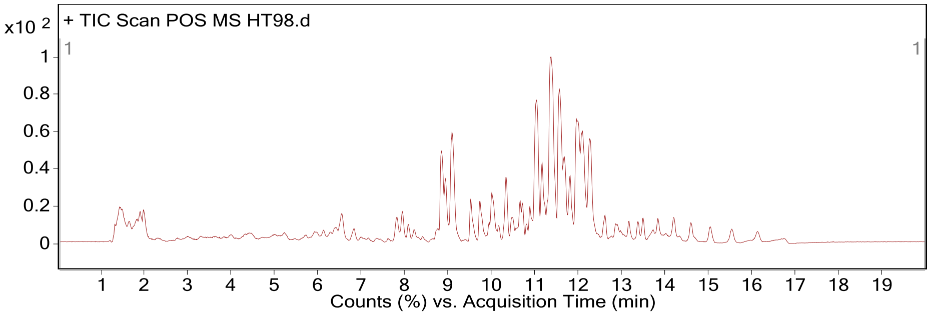

Fragmentor Voltage 130 **Collision Energy** 0 **Ionization Mode** ESI

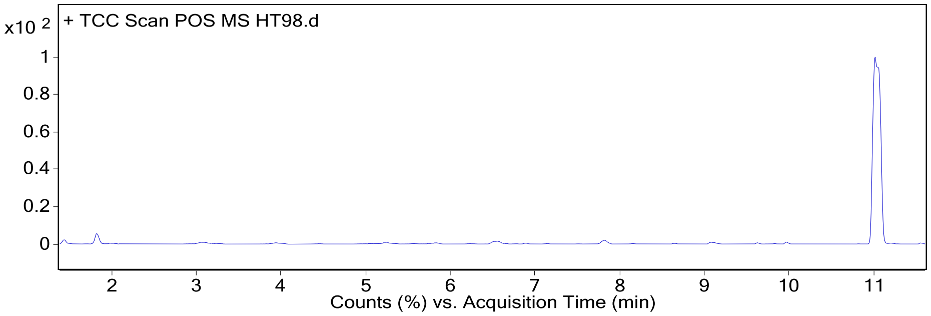

Compound Table

Compound Label	RT	Mass	Name	Formula	Tgt Mass	Diff (ppm)	SignificantIonMz
Cpd 1: Fusarinolic acid	1.44	195.0897	Fusarinolic acid	C10 H13 N O3	195.0895	0.61	213.1234
Cpd 2: Terrein	1.45	154.063	Terrein	C8 H10 O3	154.063	0.03	172.0968
Cpd 3: Chlamydospordiol	1.7	228.0998	Chlamydospordiol	C11 H16 O5	228.0998	-0.07	246.1336
Cpd 4: Fusarinolic acid	1.81	195.0893	Fusarinolic acid	C10 H13 N O3	195.0895	-1.08	213.1232
Cpd 5: Cerulenin	1.83	223.1209	Cerulenin	C12 H17 N O3	223.1208	0.19	241.1547
Cpd 6: Fusaric acid	1.83	179.0946	Fusaric acid	C10 H13 N O2	179.0946	-0.34	197.1284
Cpd 7: Altenuene	1.98	292.095	Altenuene	C15 H16 O6	292.0947	1.08	310.1288
Cpd 8: Fusarinolic acid	2.01	195.0896	Fusarinolic acid	C10 H13 N O3	195.0895	0.35	196.0969
Cpd 9: Pyrenocine A	2.03	208.0738	Pyrenocine A	C11 H12 O4	208.0736	1.39	226.1077
Cpd 10: Terrein	2.9	154.0629	Terrein	C8 H10 O3	154.063	-0.44	172.0967
Cpd 11: Pyrenocine A	3.05	208.0735	Pyrenocine A	C11 H12 O4	208.0736	-0.5	226.1073
Cpd 12: Terrein	3.1	154.063	Terrein	C8 H10 O3	154.063	0.3	172.0969
Cpd 13: Terrein	3.25	154.0629	Terrein	C8 H10 O3	154.063	-0.73	172.0967
Cpd 14: 5-Methyl-mellein	3.94	192.0785	5-Methyl-mellein	C11 H12 O3	192.0786	-0.75	210.1123
Cpd 15: Terrein	3.98	154.0629	Terrein	C8 H10 O3	154.063	-0.53	172.0967
Cpd 16: 5-Methyl-mellein	4.4	192.0785	5-Methyl-mellein	C11 H12 O3	192.0786	-0.49	210.1124
Cpd 17: 5-Methyl-mellein	4.46	192.0788	5-Methyl-mellein	C11 H12 O3	192.0786	0.67	210.1126
Cpd 18: Fusaric acid	4.97	179.0946	Fusaric acid	C10 H13 N O2	179.0946	0.11	197.1285

Cpd 19: Oosporein	5.23	306.0379	Oosporein	C14 H10 O8	306.0376	0.97	307.0451
Cpd 20: Fusaric acid	5.35	179.0947	Fusaric acid	C10 H13 N O2	179.0946	0.27	180.102
Cpd 21: Infectopyrone	5.59	264.0998	Infectopyrone	C14 H16 O5	264.0998	0.03	282.1336
Cpd 22: Fusaric acid	5.82	179.0948	Fusaric acid	C10 H13 N O2	179.0946	0.85	180.1021
Cpd 23: Fusaric acid	6.24	179.0947	Fusaric acid	C10 H13 N O2	179.0946	0.42	180.102
Cpd 24: Pyrenocine A	6.41	208.0737	Pyrenocine A	C11 H12 O4	208.0736	0.9	226.1076
Cpd 25: Enniatin K1	6.53	625.3918	Enniatin K1	C32 H55 N3 O9	625.3938	-3.26	648.3811
Cpd 26: Brevianamid F	6.55	283.1325	Brevianamid F	C16 H17 N3 O2	283.1321	1.46	284.1398
Cpd 27: Paspalic acid	6.67	268.121	Paspalic acid	C16 H16 N2 O2	268.1212	-0.53	286.1549
Cpd 28: Pyrenocine A	6.77	208.0739	Pyrenocine A	C11 H12 O4	208.0736	1.44	226.1077
Cpd 29: Enniatin K1	6.89	625.3912	Enniatin K1	C32 H55 N3 O9	625.3938	-4.15	648.3805
Cpd 30: Aphidicolin	7.14	338.2444	Aphidicolin	C20 H34 O4	338.2457	-3.97	377.2075
Cpd 31: Marcfortine C	7.52	447.2524	Marcfortine C	C27 H33 N3 O3	447.2522	0.54	465.2863
Cpd 32: Fusaric acid	7.82	179.0947	Fusaric acid	C10 H13 N O2	179.0946	0.59	180.102
Cpd 33: beta-Zearalenol	7.88	320.1626	beta-Zearalenol	C18 H24 O5	320.1624	0.67	338.1964
Cpd 34: Epiequisetin	8.15	373.2236	Epiequisetin	C22 H31 N O4	373.2253	-4.71	412.1867
Cpd 35: Culmorin	8.65	238.1935	Culmorin	C15 H26 O2	238.1933	1.08	256.2274
Cpd 36: HFB1 / Hydrolysed Fumonisin B1	9.08	405.3452	HFB1 / Hydrolysed Fumonisin B1	C22 H47 N O5	405.3454	-0.47	406.3525
Cpd 37: Deepoxy deoxynivalenol	9.1	280.1307	Deepoxy deoxynivalenol	C15 H20 O5	280.1311	-1.32	281.138
Cpd 38: HFB3 / Hydrolysed Fumonisin B3	9.63	389.3506	HFB3 / Hydrolysed Fumonisin B3	C22 H47 N O4	389.3505	0.16	390.3578
Cpd 39: Siccanol	9.73	402.2752	Siccanol	C25 H38 O4	402.277	-4.54	441.2383
Cpd 40: 2-Amino-14,16-dimethyloctadecan-3-ol	9.83	313.3343	2-Amino-14,16-dimethyloctadecan-3-ol	C20 H43 N O	313.3345	-0.55	314.3416
Cpd 41: Paspaline	9.97	421.2982	Paspaline	C28 H39 N O2	421.2981	0.25	439.332
Cpd 42: Enniatin A1	10.82	667.4417	Enniatin A1	C35 H61 N3 O9	667.4408	1.36	685.4755
Cpd 43: Enniatin A2	10.99	681.4562	Enniatin A2	C36 H63 N3 O9	681.4564	-0.39	1380.9466
Cpd 44: Enniatin A2	10.99	681.4548	Enniatin A2	C36 H63 N3 O9	681.4564	-2.35	699.4876
Cpd 45: Beauvericin	11.04	783.4109	Beauvericin	C45 H57 N3 O9	783.4095	1.8	801.445
Cpd 46: Beauvericin	11.21	783.4059	Beauvericin	C45 H57 N3 O9	783.4095	-4.63	801.4398
Cpd 47: Sirolimus (Rapamycin)	11.56	913.5576	Sirolimus (Rapamycin)	C51 H79 N O13	913.5551	2.71	931.5915

Compound Label	Name	*m/z*	RT	Algorithm	Mass
Cpd 1: Fusarinolic acid	**Fusarinolic acid**	213.1234	1.44	Find by Molecular Feature	195.0897

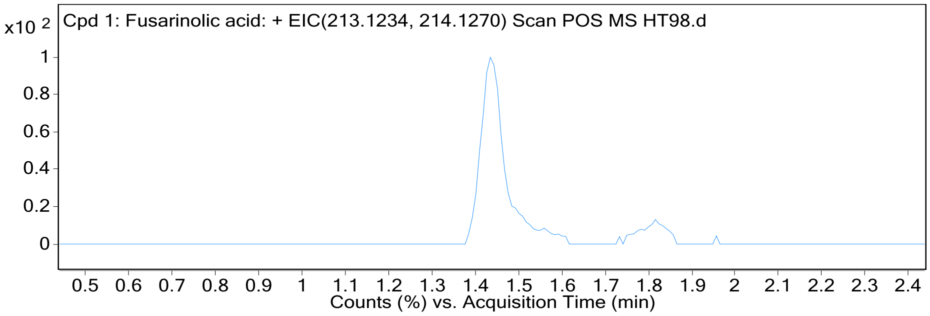

MFE MS Zoomed Spectrum

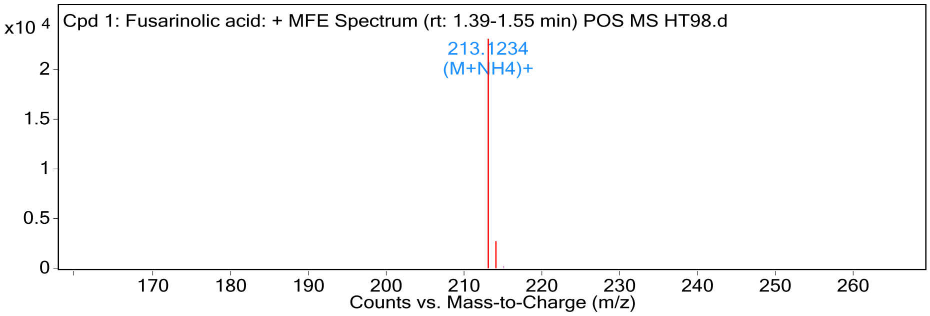

Compound Label	Name	*m/z*	RT	Algorithm	Mass
Cpd 2: Terrein	**Terrein**	172.0968	1.45	Find by Molecular Feature	154.063

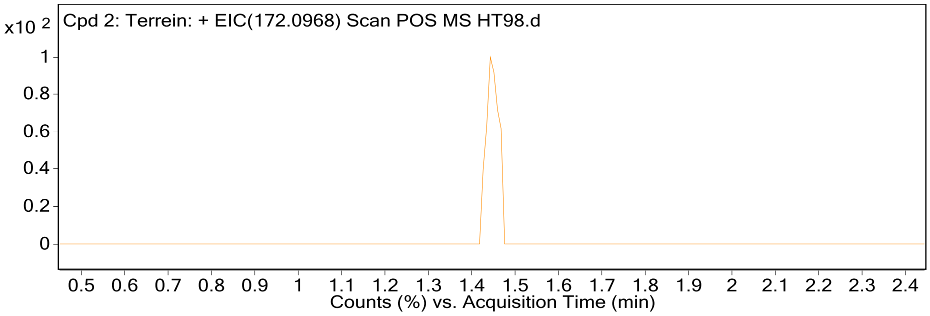

MFE MS Zoomed Spectrum

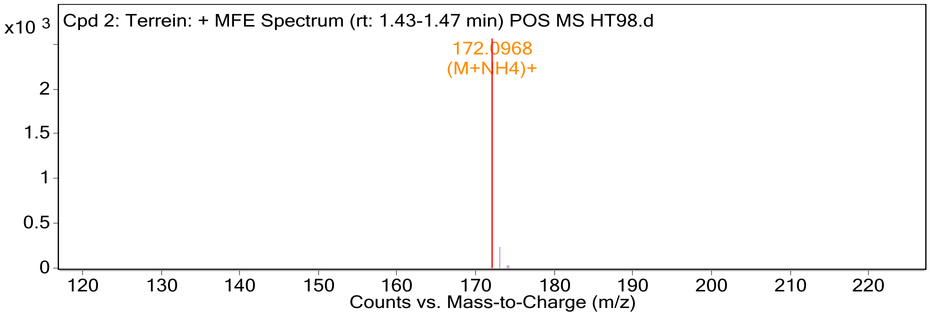

Compound Label	Name	*m/z*	RT	Algorithm	Mass
Cpd 3: Chlamydospordiol	**Chlamydospordiol**	246.1336	1.7	Find by Molecular Feature	228.0998

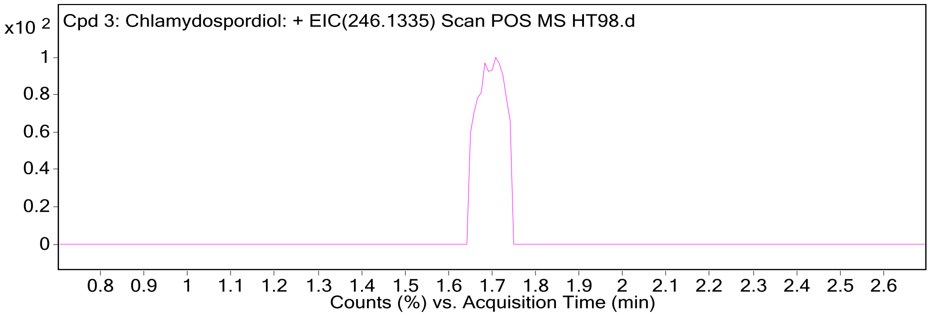

MFE MS Zoomed Spectrum

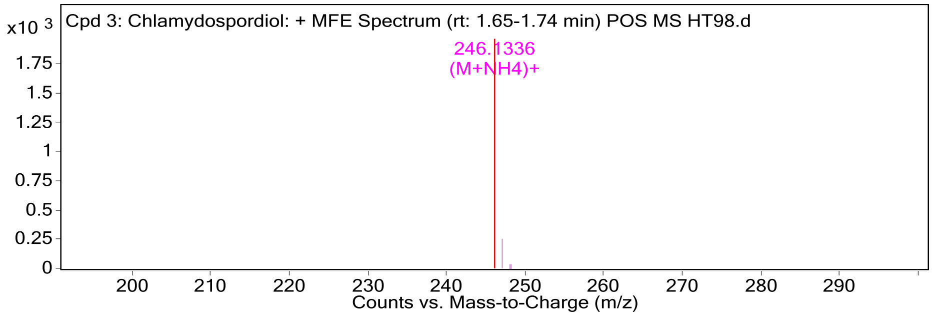

Compound Label	Name	*m/z*	RT	Algorithm	Mass
Cpd 4: Fusarinolic acid	**Fusarinolic acid**	213.1232	1.81	Find by Molecular Feature	195.0893

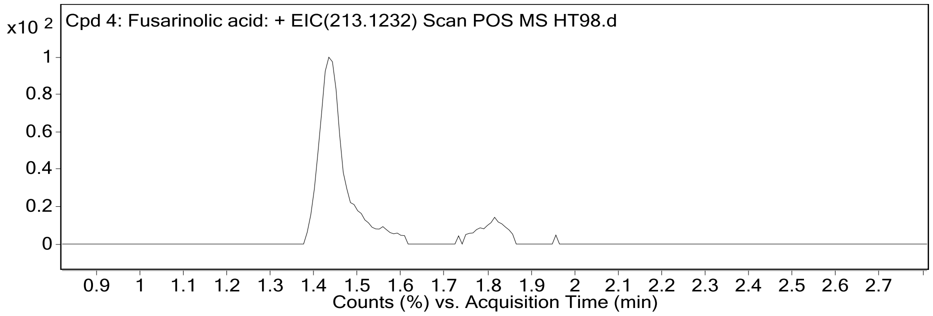

MFE MS Zoomed Spectrum

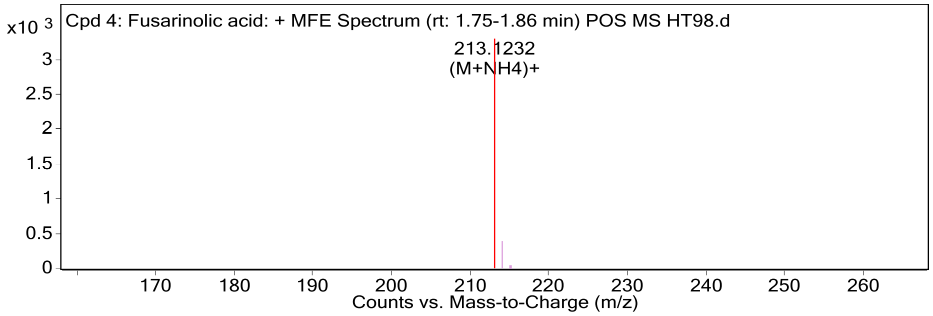

Compound Label	Name	*m/z*	RT	Algorithm	Mass
Cpd 5: Cerulenin	**Cerulenin**	241.1547	1.83	Find by Molecular Feature	223.1209

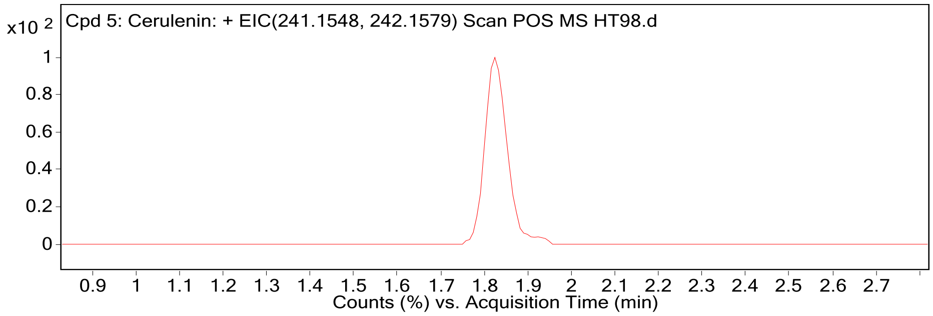

MFE MS Zoomed Spectrum

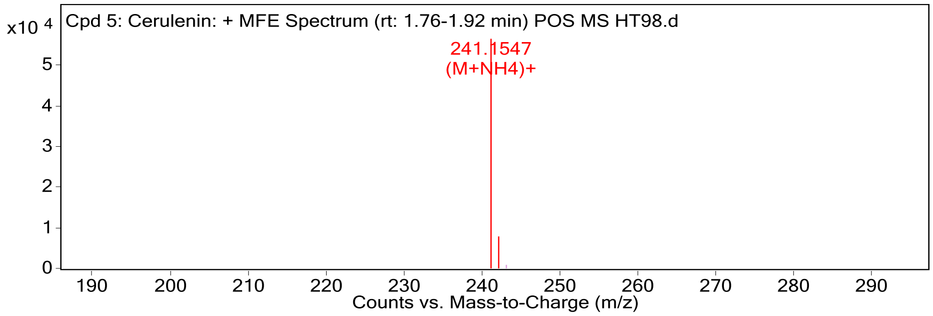

Compound Label	Name	*m/z*	RT	Algorithm	Mass
Cpd 6: Fusaric acid	**Fusaric acid**	197.1284	1.83	Find by Molecular Feature	179.0946

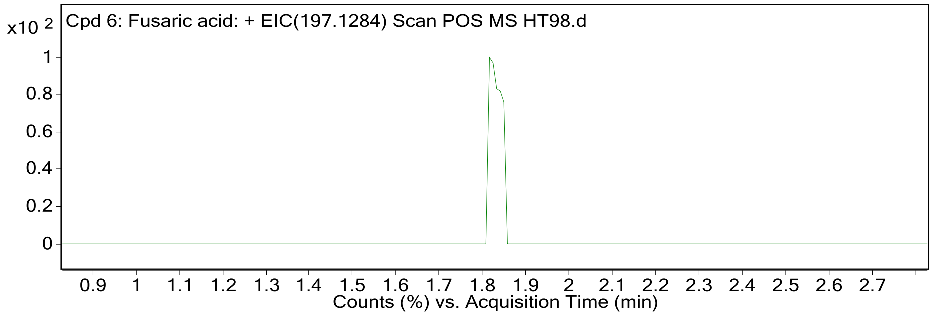

MFE MS Zoomed Spectrum

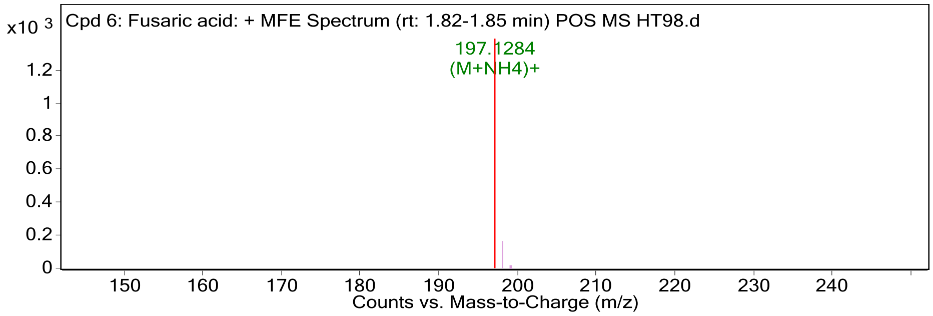

Compound Label	Name	*m/z*	RT	Algorithm	Mass
Cpd 7: Altenuene	**Altenuene**	310.1288	1.98	Find by Molecular Feature	292.095

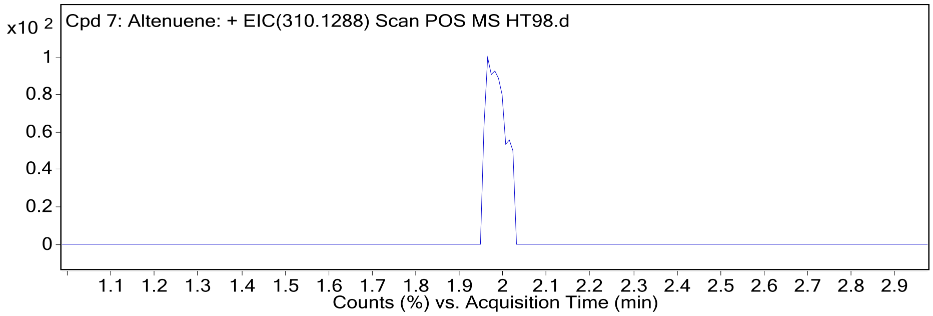

MFE MS Zoomed Spectrum

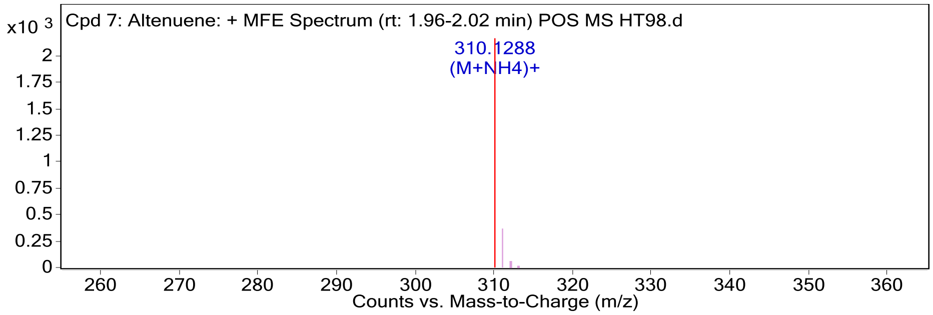

Compound Label	Name	*m/z*	RT	Algorithm	Mass
Cpd 8: Fusarinolic acid	**Fusarinolic acid**	196.0969	2.01	Find by Molecular Feature	195.0896

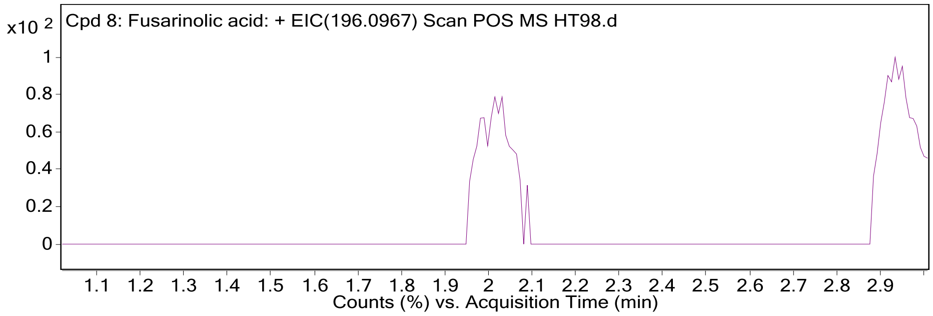

MFE MS Zoomed Spectrum

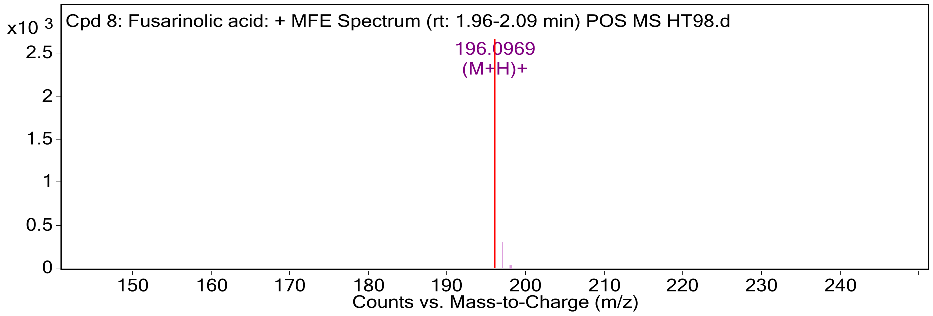

Compound Label	Name	*m/z*	RT	Algorithm	Mass

Cpd 9: Pyrenocine A	**Pyrenocine A**	226.1077	2.03	Find by Molecular Feature	208.0738

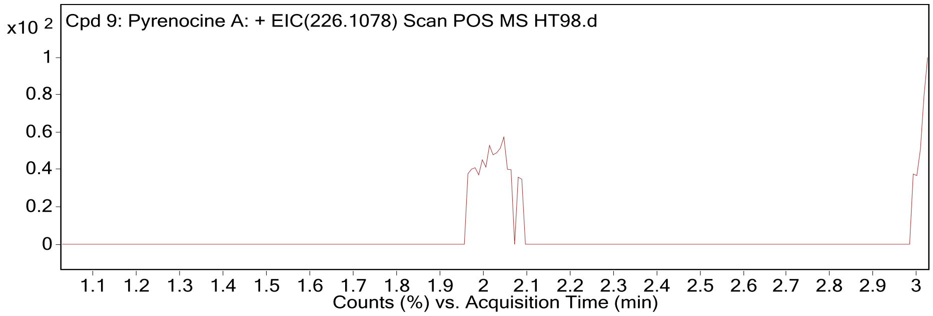

MFE MS Zoomed Spectrum

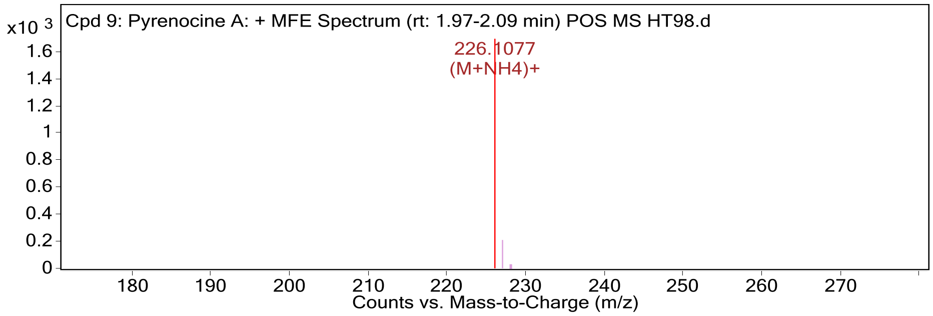

Compound Label	Name	*m/z*	RT	Algorithm	Mass
Cpd 10: Terrein	**Terrein**	172.0967	2.9	Find by Molecular Feature	154.0629

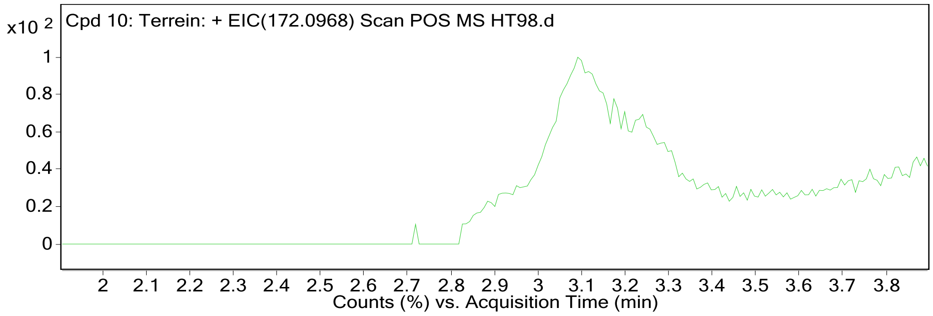

MFE MS Zoomed Spectrum

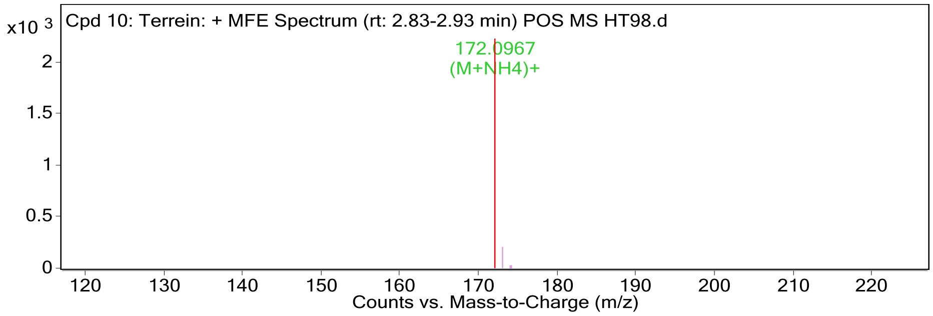

Compound Label	Name	*m/z*	RT	Algorithm	Mass
Cpd 11: Pyrenocine A	**Pyrenocine A**	226.1073	3.05	Find by Molecular Feature	208.0735

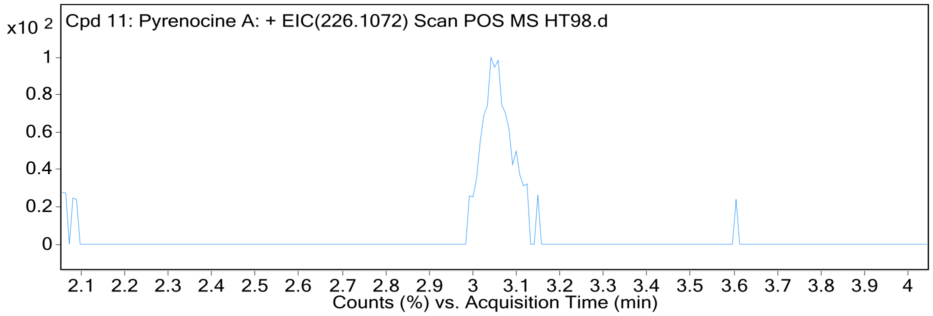

MFE MS Zoomed Spectrum

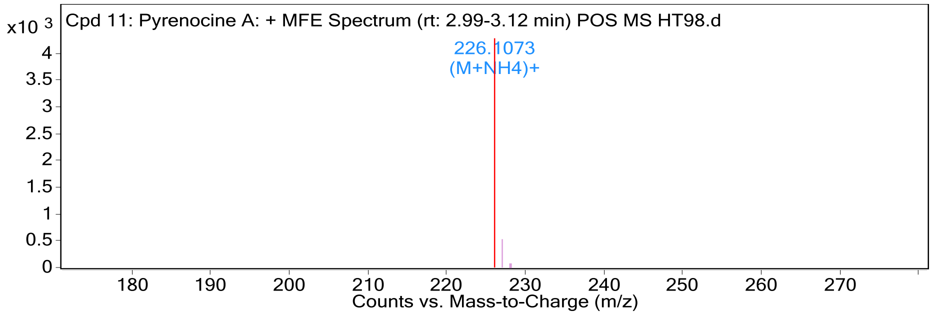

Compound Label	Name	*m/z*	RT	Algorithm	Mass
Cpd 12: Terrein	**Terrein**	172.0969	3.1	Find by Molecular Feature	154.063

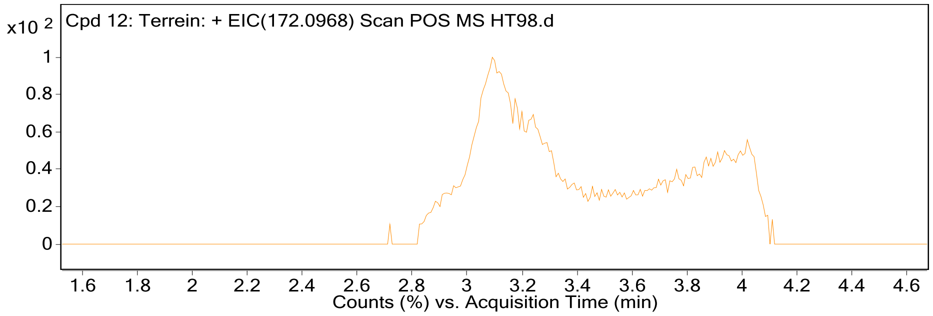

MFE MS Zoomed Spectrum

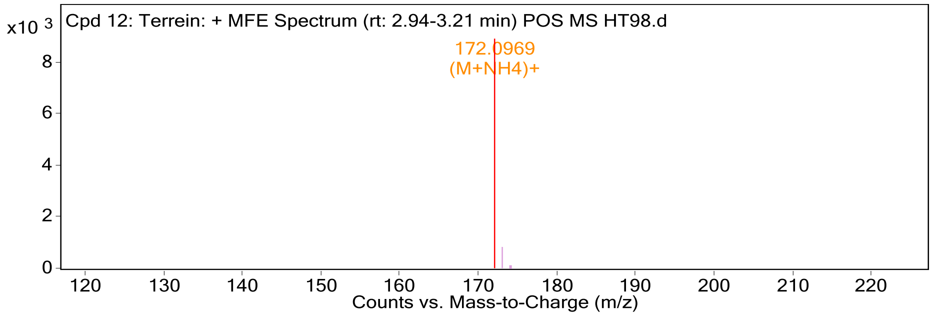

Compound Label	Name	*m/z*	RT	Algorithm	Mass
Cpd 13: Terrein	**Terrein**	172.0967	3.25	Find by Molecular Feature	154.0629

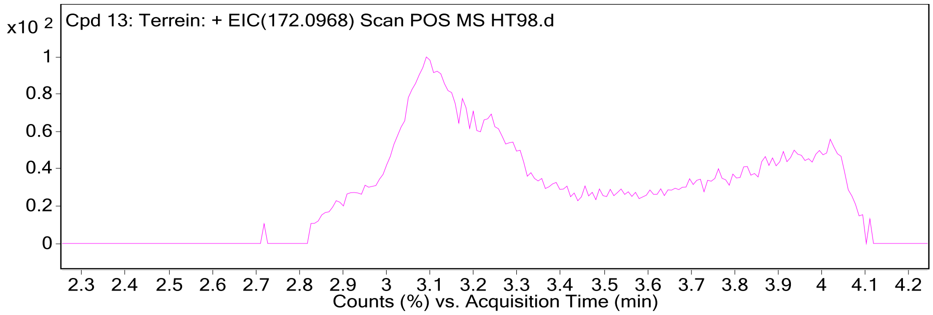

MFE MS Zoomed Spectrum

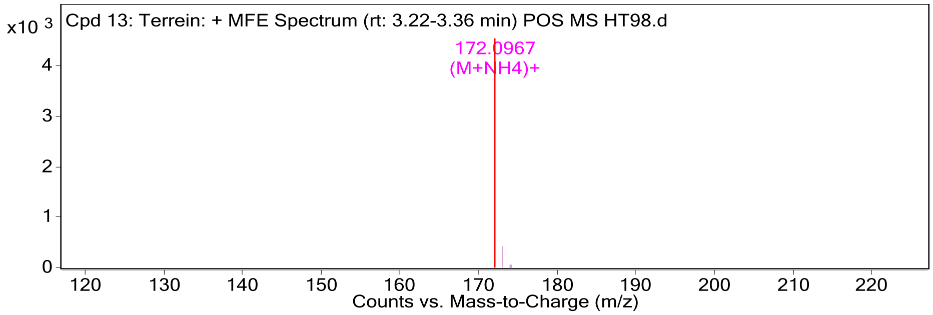

Compound Label	Name	*m/z*	RT	Algorithm	Mass
Cpd 14: 5-Methyl-mellein	**5-Methyl-mellein**	210.1123	3.94	Find by Molecular Feature	192.0785

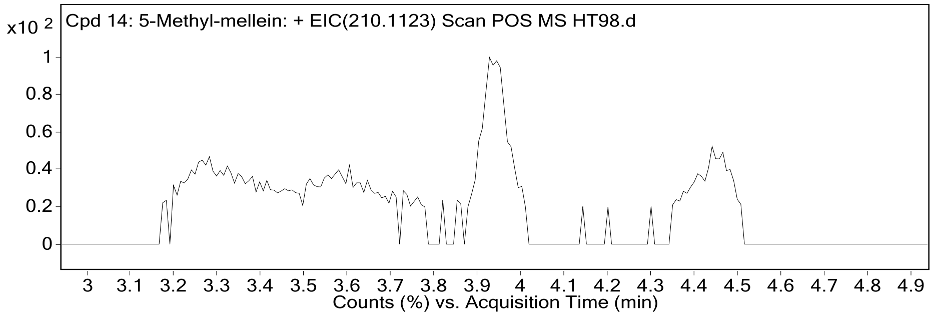

MFE MS Zoomed Spectrum

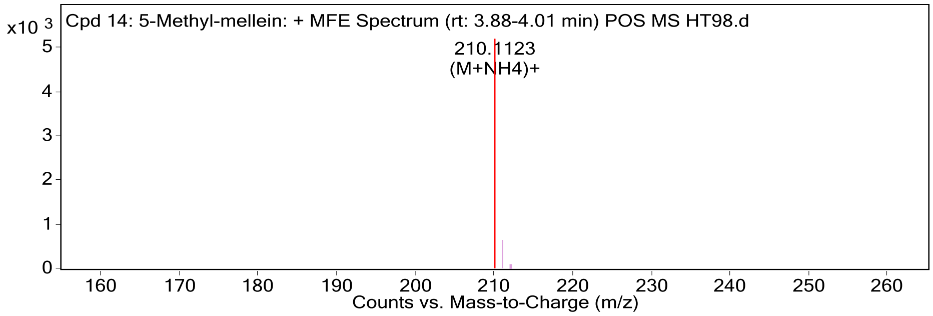

Qualitative Compound Report

Compound Label	Name	*m/z*	RT	Algorithm	Mass
Cpd 15: Terrein	**Terrein**	172.0967	3.98	Find by Molecular Feature	154.0629

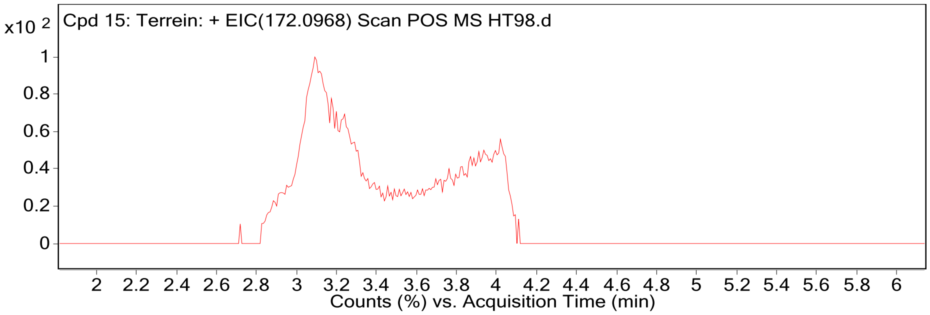

MFE MS Zoomed Spectrum

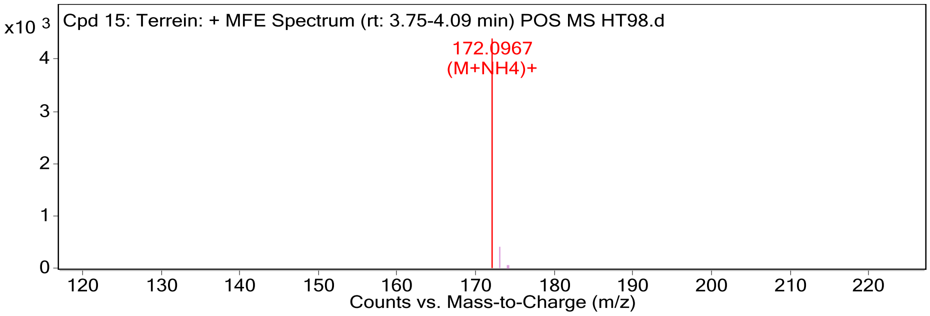

Compound Label	Name	*m/z*	RT	Algorithm	Mass
Cpd 16: 5-Methyl-mellein	**5-Methyl-mellein**	210.1124	4.4	Find by Molecular Feature	192.0785

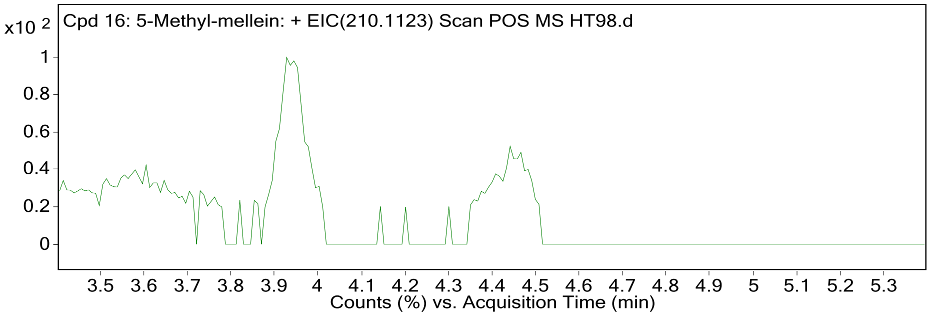

MFE MS Zoomed Spectrum

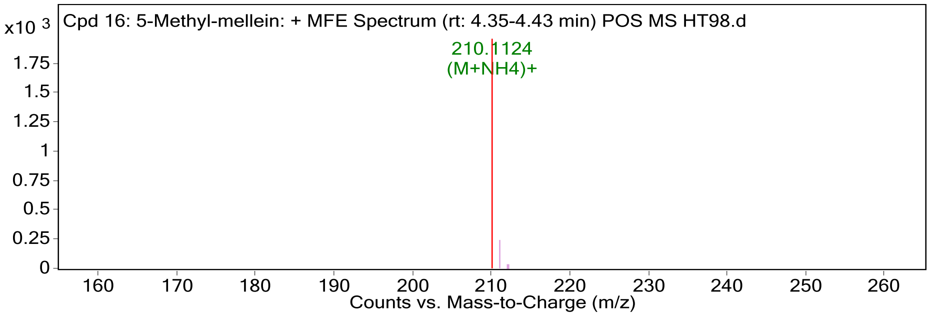

Compound Label	Name	*m/z*	RT	Algorithm	Mass
Cpd 17: 5-Methyl-mellein	**5-Methyl-mellein**	210.1126	4.46	Find by Molecular Feature	192.0788

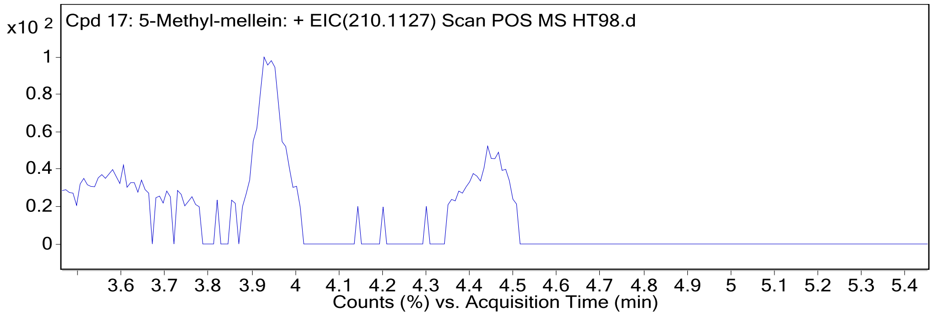

MFE MS Zoomed Spectrum

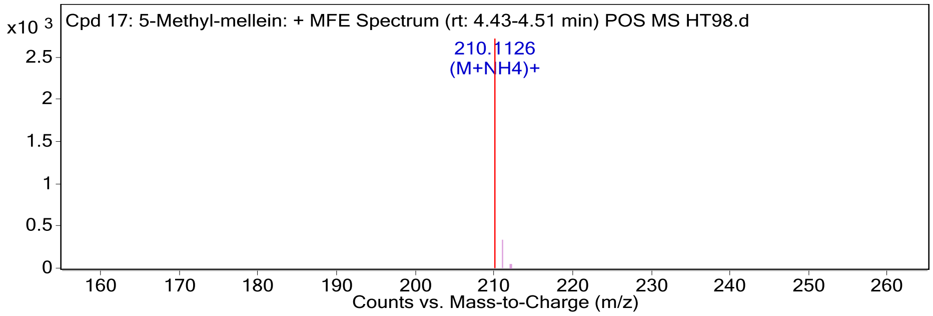

Compound Label	Name	*m/z*	RT	Algorithm	Mass
Cpd 18: Fusaric acid	**Fusaric acid**	197.1285	4.97	Find by Molecular Feature	179.0946

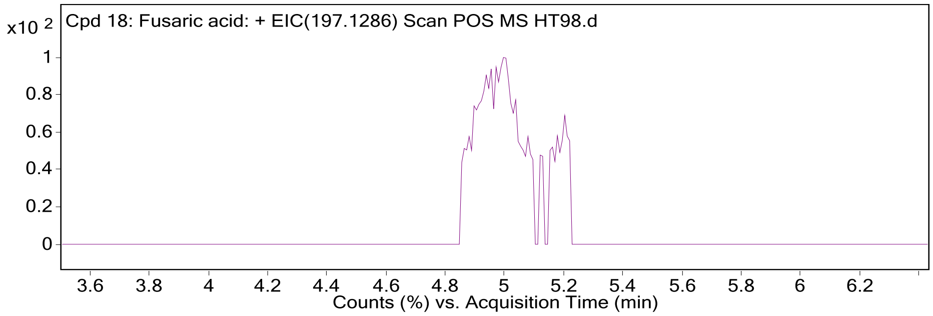

MFE MS Zoomed Spectrum

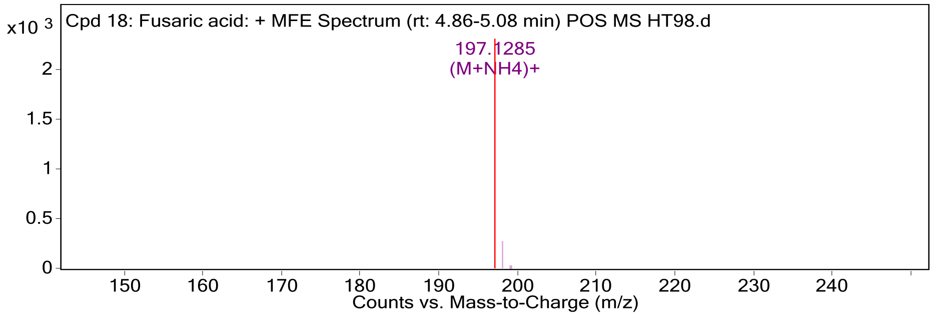

Compound Label	Name	*m/z*	RT	Algorithm	Mass
Cpd 19: Oosporein	**Oosporein**	307.0451	5.23	Find by Molecular Feature	306.0379

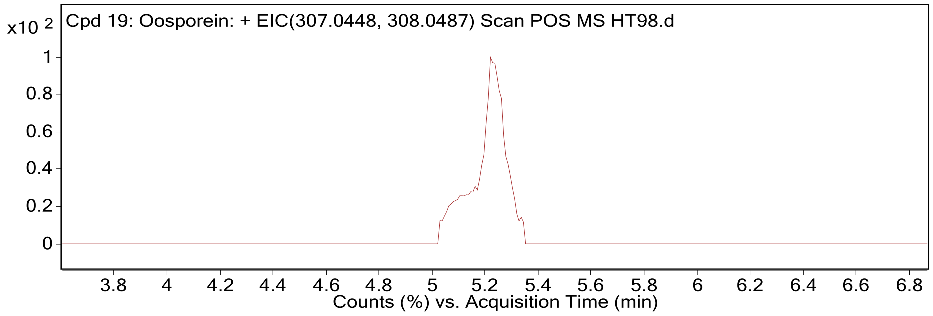

MFE MS Zoomed Spectrum

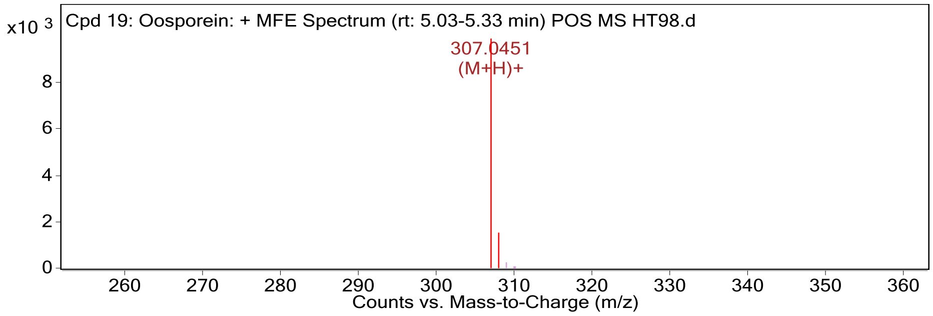

Compound Label	Name	*m/z*	RT	Algorithm	Mass
Cpd 20: Fusaric acid	**Fusaric acid**	180.102	5.35	Find by Molecular Feature	179.0947

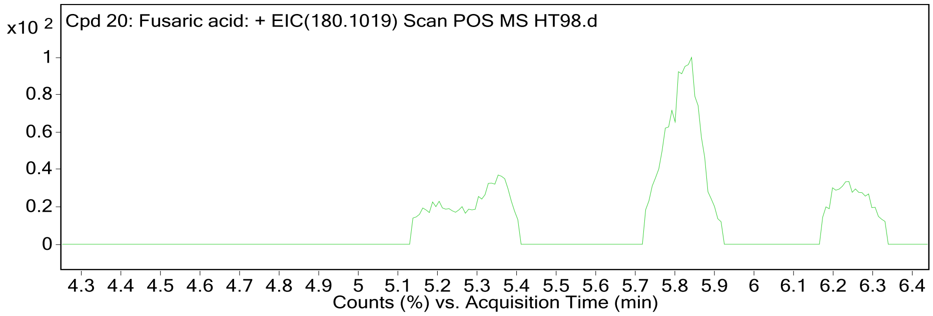

MFE MS Zoomed Spectrum

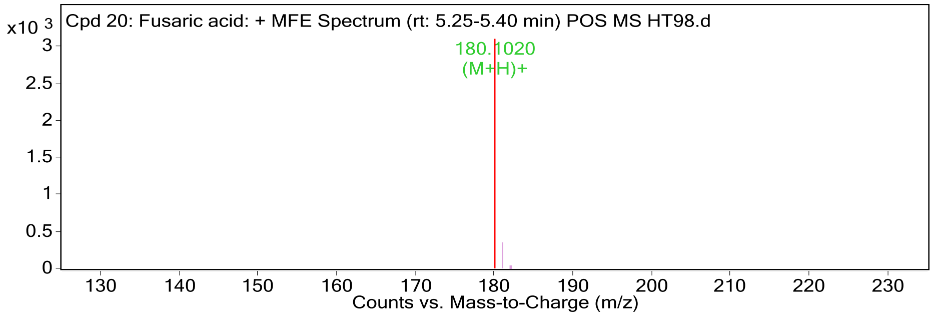

Compound Label	Name	*m/z*	RT	Algorithm	Mass
Cpd 21: Infectopyrone	**Infectopyrone**	282.1336	5.59	Find by Molecular Feature	264.0998

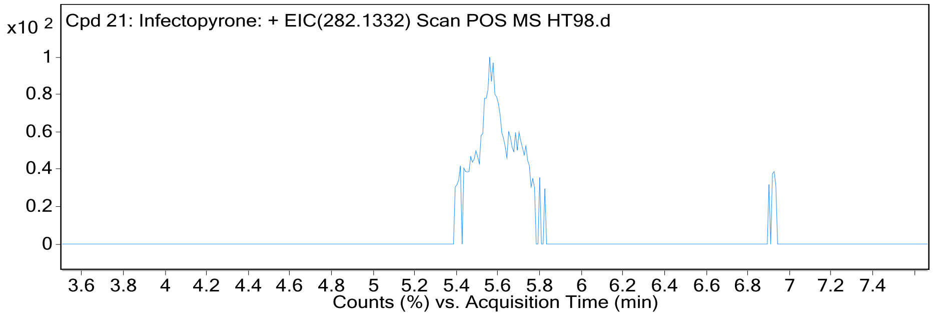

MFE MS Zoomed Spectrum

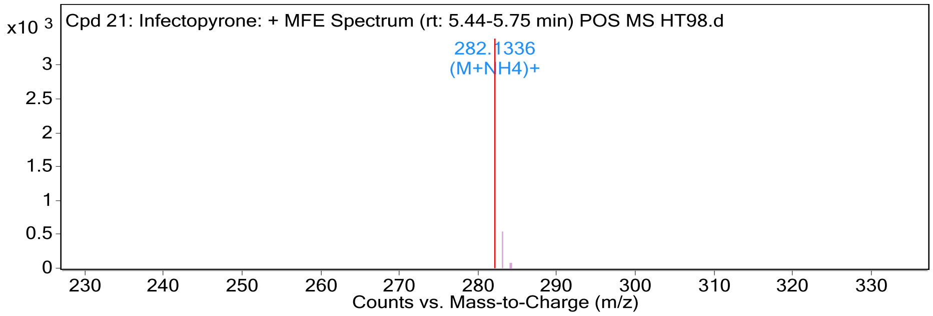

Compound Label	Name	*m/z*	RT	Algorithm	Mass
Cpd 22: Fusaric acid	**Fusaric acid**	180.1021	5.82	Find by Molecular Feature	179.0948

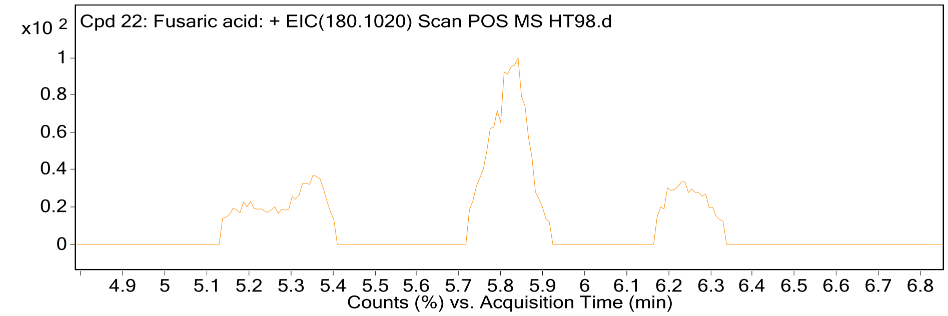

MFE MS Zoomed Spectrum

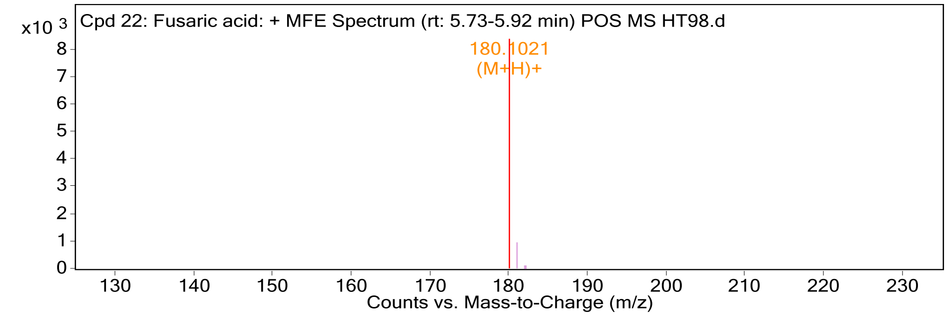

Compound Label	Name	*m/z*	RT	Algorithm	Mass
Cpd 23: Fusaric acid	**Fusaric acid**	180.102	6.24	Find by Molecular Feature	179.0947

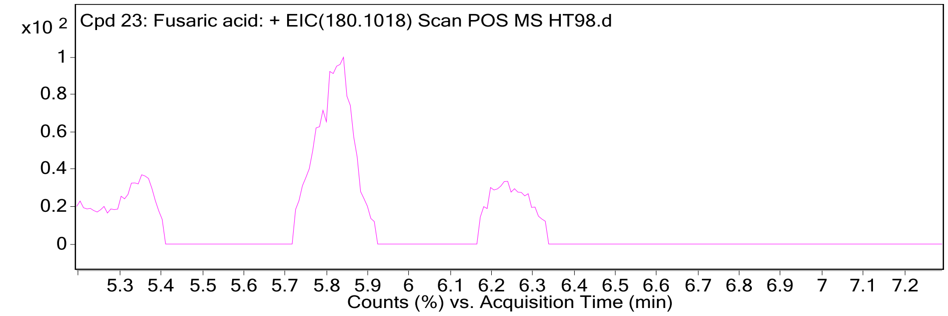

MFE MS Zoomed Spectrum

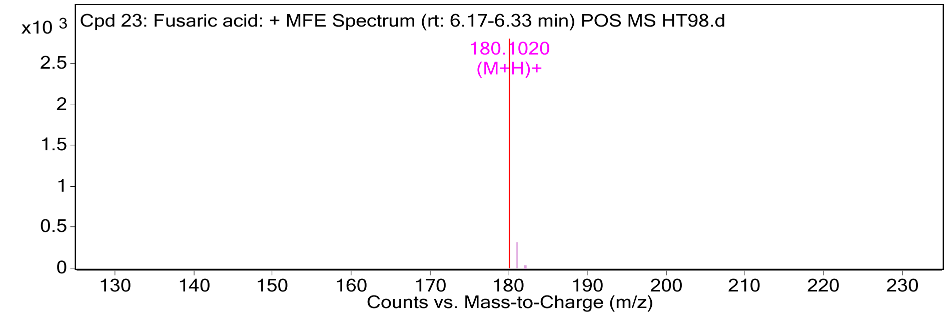

Compound Label	Name	*m/z*	RT	Algorithm	Mass
Cpd 24: Pyrenocine A	**Pyrenocine A**	226.1076	6.41	Find by Molecular Feature	208.0737

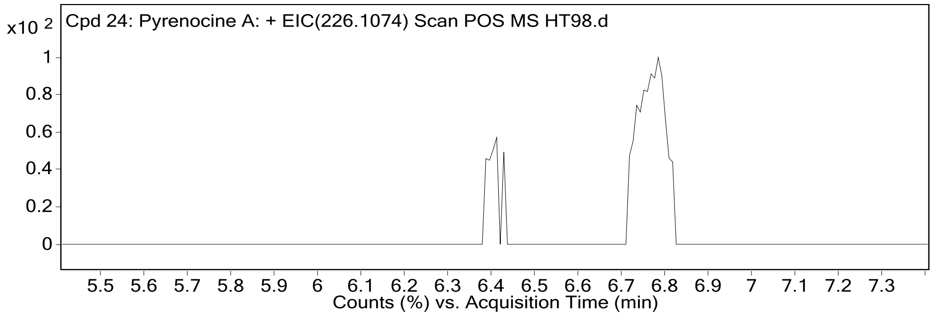

MFE MS Zoomed Spectrum

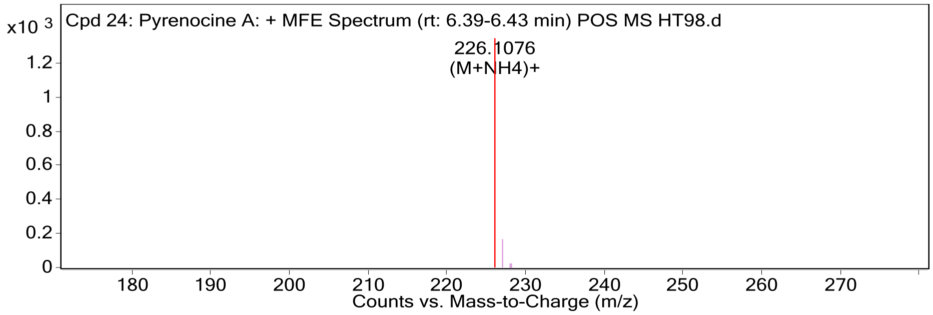

Compound Label	Name	*m/z*	RT	Algorithm	Mass
Cpd 25: Enniatin K1	**Enniatin K1**	648.3811	6.53	Find by Molecular Feature	625.3918

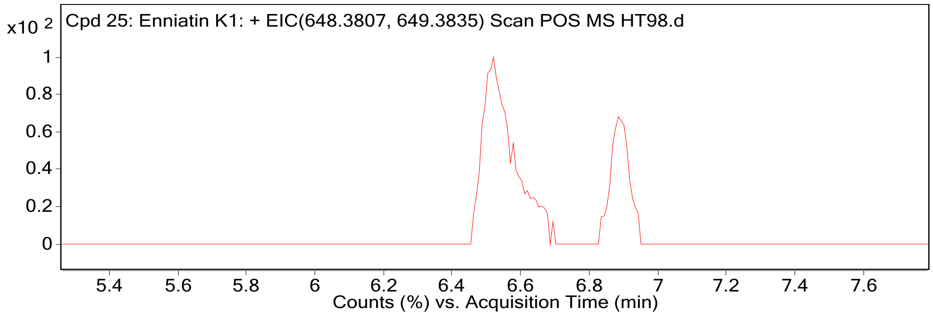

MFE MS Zoomed Spectrum

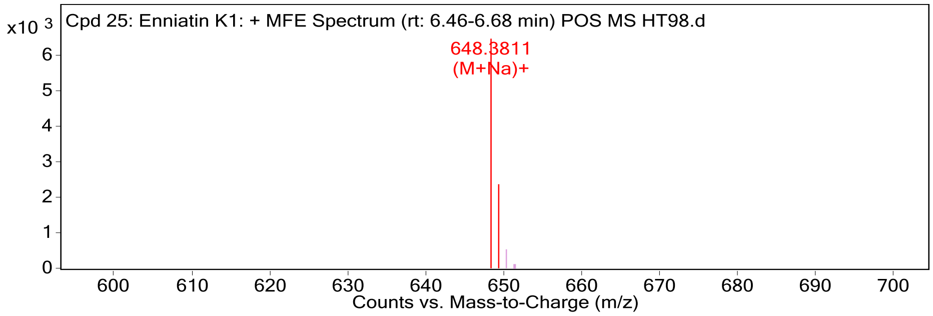

Compound Label	Name	*m/z*	RT	Algorithm	Mass

Cpd 26: Brevianamid F	**Brevianamid F**	284.1398	6.55	Find by Molecular Feature	283.1325

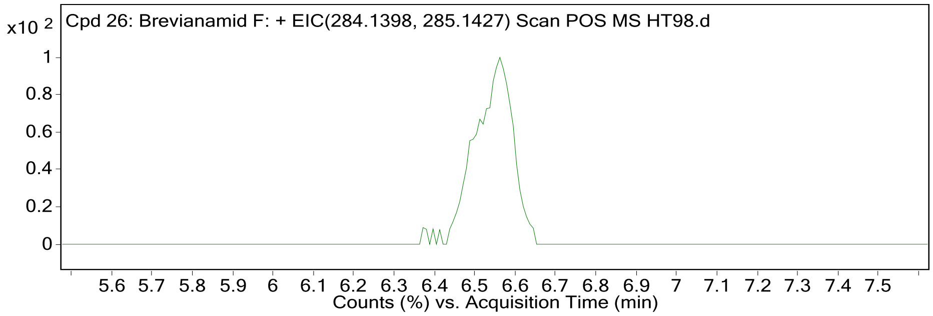

MFE MS Zoomed Spectrum

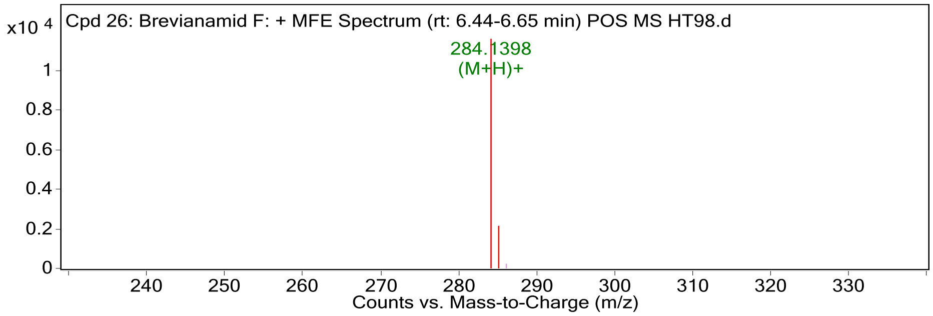

Compound Label	Name	*m/z*	RT	Algorithm	Mass
Cpd 27: Paspalic acid	**Paspalic acid**	286.1549	6.67	Find by Molecular Feature	268.121

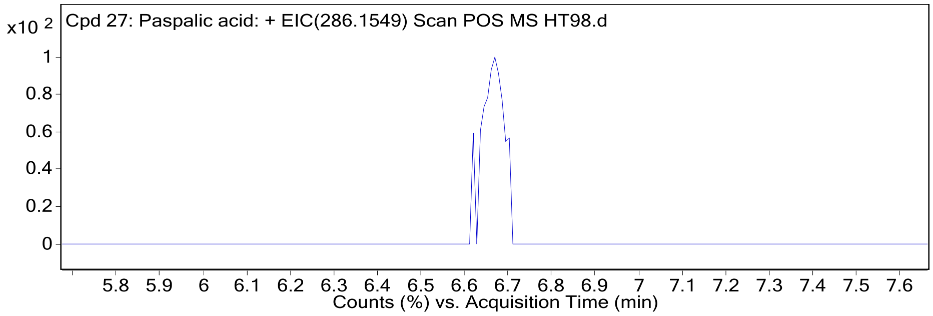

MFE MS Zoomed Spectrum

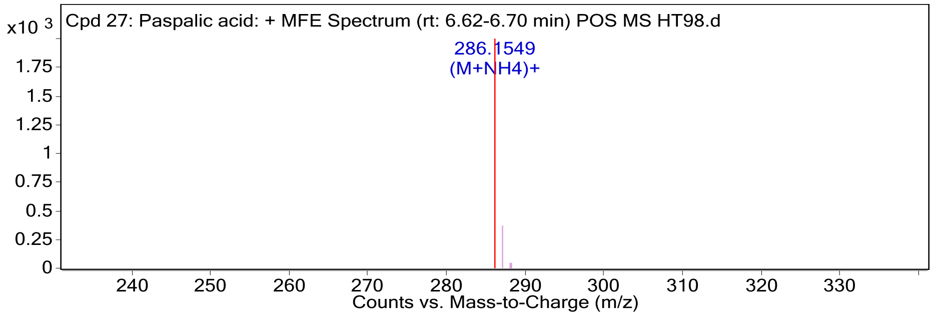

Compound Label	Name	*m/z*	RT	Algorithm	Mass
Cpd 28: Pyrenocine A	**Pyrenocine A**	226.1077	6.77	Find by Molecular Feature	208.0739

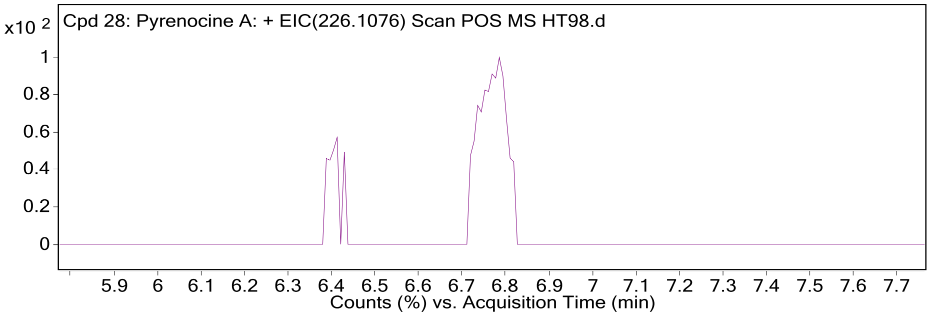

MFE MS Zoomed Spectrum

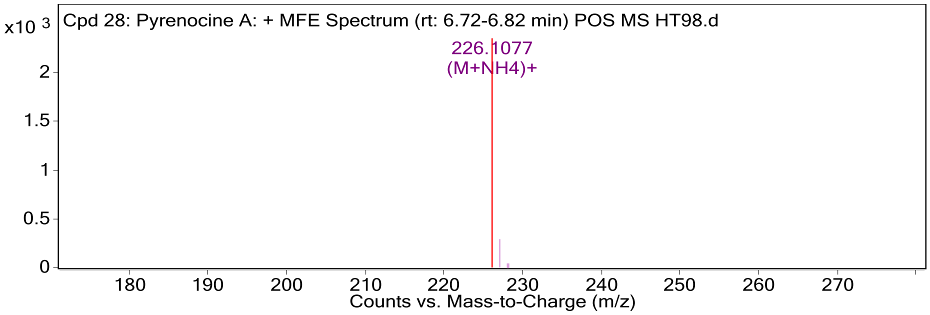

Compound Label	Name	*m/z*	RT	Algorithm	Mass
Cpd 29: Enniatin K1	**Enniatin K1**	648.3805	6.89	Find by Molecular Feature	625.3912

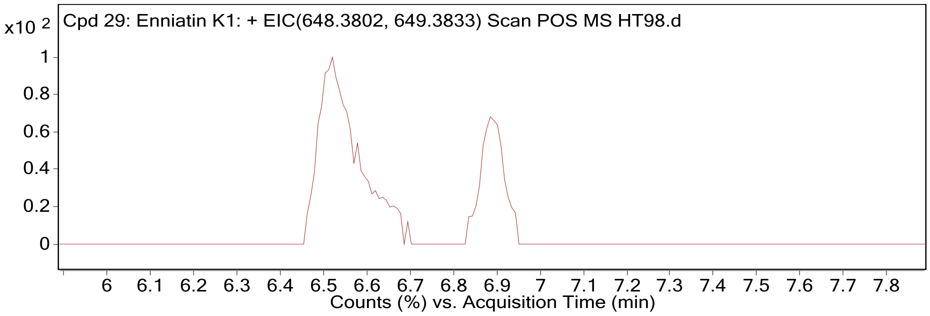

MFE MS Zoomed Spectrum

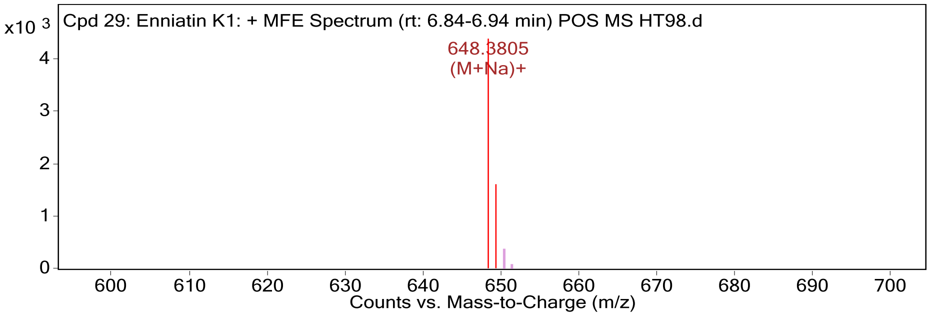

Qualitative Compound Report

Compound Label	Name	*m/z*	RT	Algorithm	Mass
Cpd 30: Aphidicolin	**Aphidicolin**	377.2075	7.14	Find by Molecular Feature	338.2444

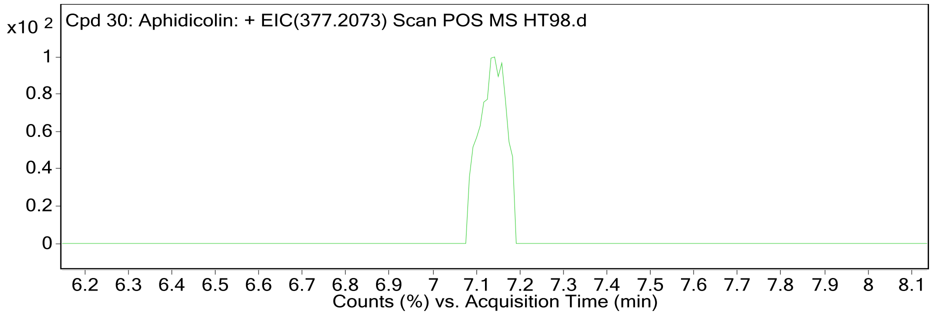

MFE MS Zoomed Spectrum

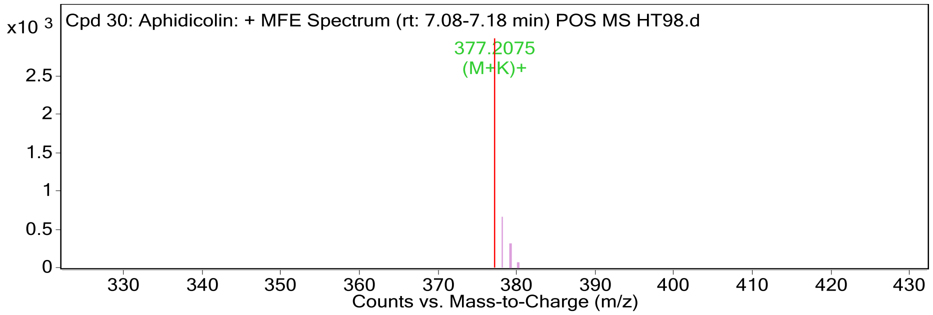

Compound Label	Name	*m/z*	RT	Algorithm	Mass
Cpd 31: Marcfortine C	**Marcfortine C**	465.2863	7.52	Find by Molecular Feature	447.2524

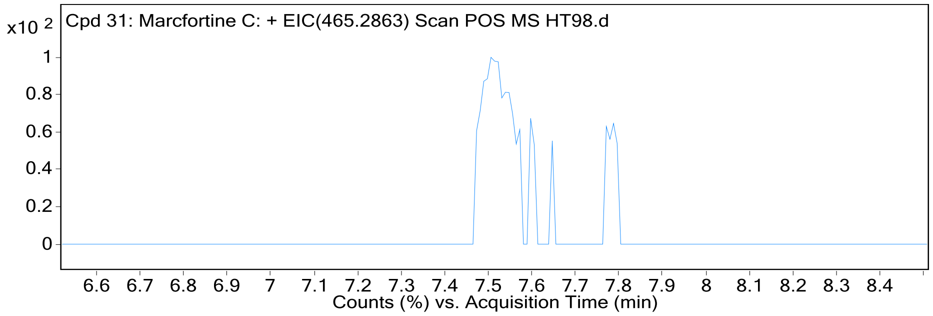

MFE MS Zoomed Spectrum

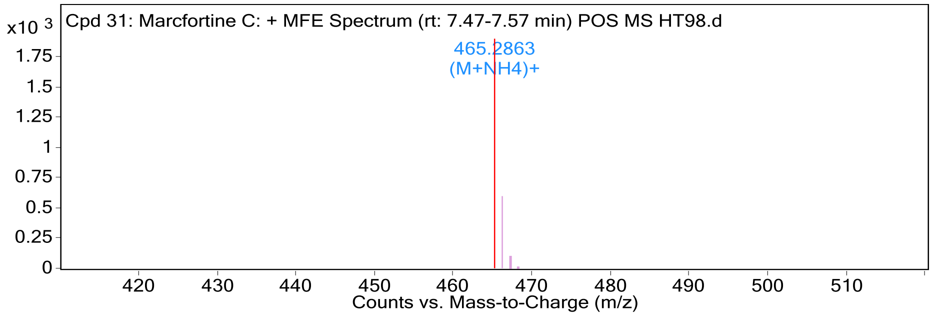

Compound Label	Name	*m/z*	RT	Algorithm	Mass
Cpd 32: Fusaric acid	**Fusaric acid**	180.102	7.82	Find by Molecular Feature	179.0947

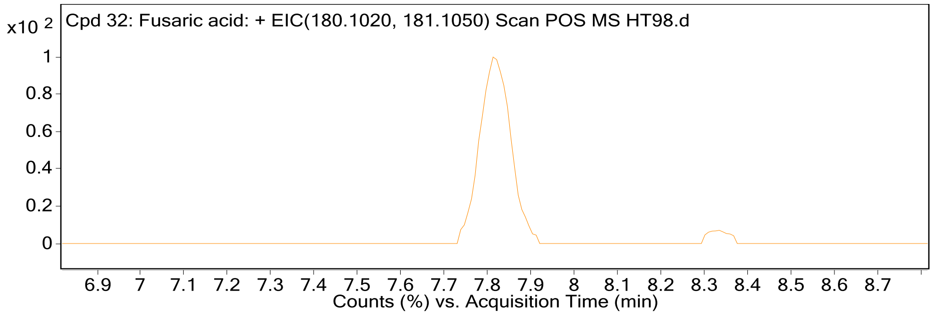

MFE MS Zoomed Spectrum

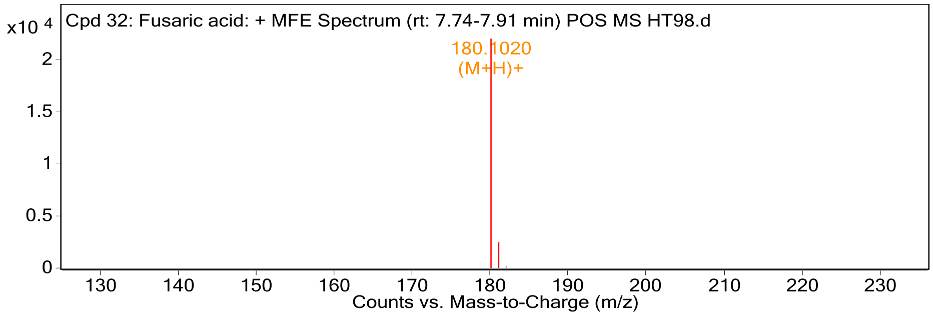

Compound Label	Name	*m/z*	RT	Algorithm	Mass
Cpd 33: beta-Zearalenol	**beta-Zearalenol**	338.1964	7.88	Find by Molecular Feature	320.1626

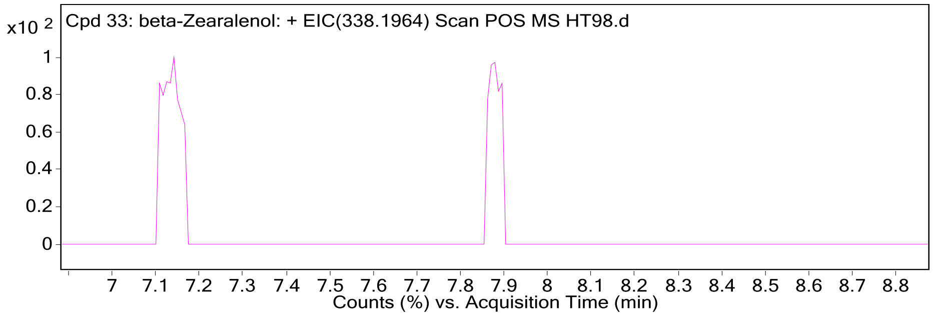

MFE MS Zoomed Spectrum

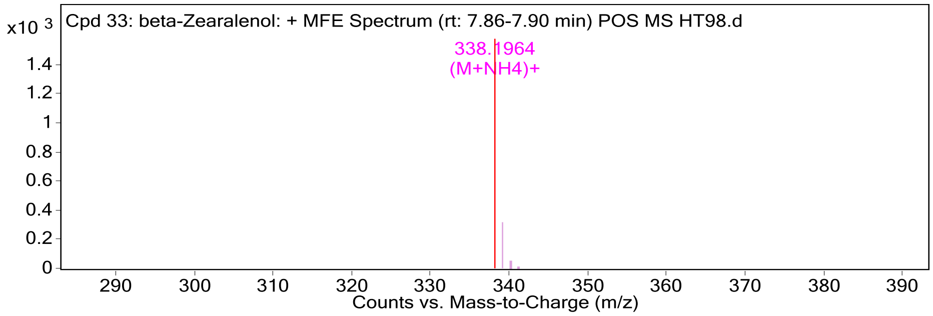

Compound Label	Name	*m/z*	RT	Algorithm	Mass
Cpd 34: Epiequisetin	**Epiequisetin**	412.1867	8.15	Find by Molecular Feature	373.2236

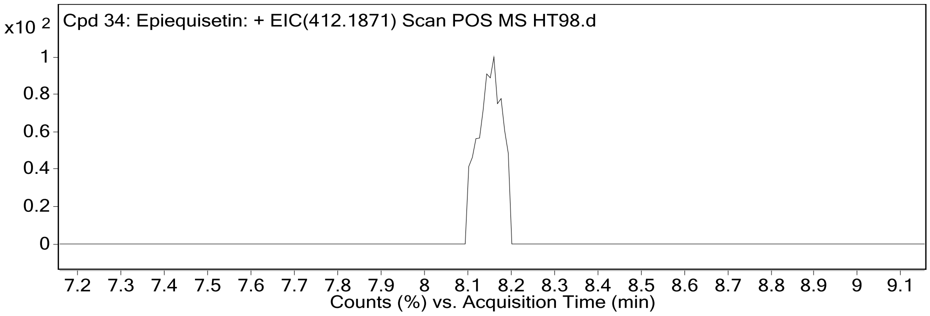

MFE MS Zoomed Spectrum

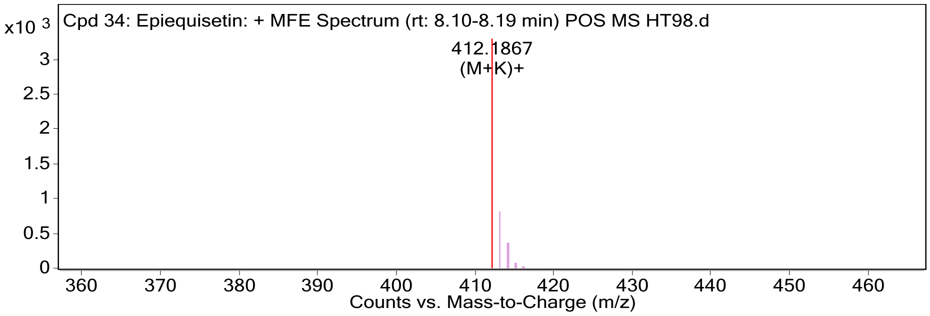

Compound Label	Name	*m/z*	RT	Algorithm	Mass
Cpd 35: Culmorin	**Culmorin**	256.2274	8.65	Find by Molecular Feature	238.1935

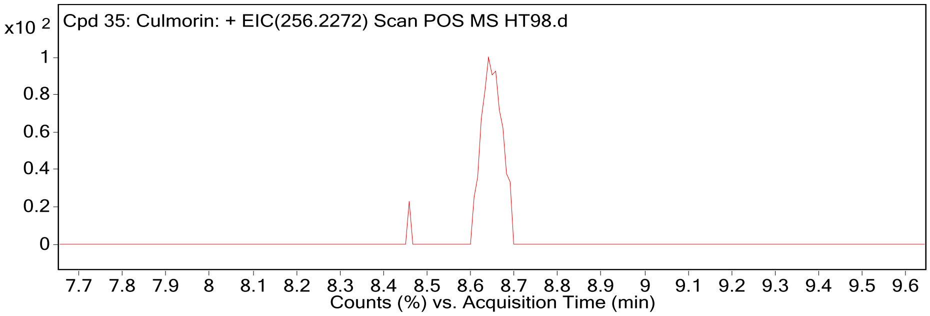

MFE MS Zoomed Spectrum

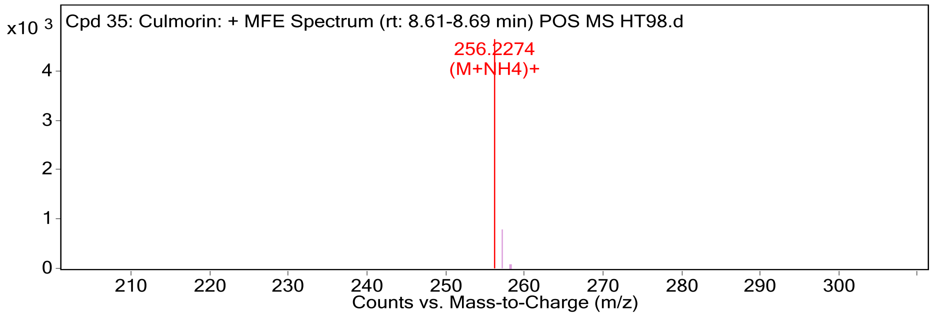

Compound Label	Name	*m/z*	RT	Algorithm	Mass
Cpd 36: HFB1 / Hydrolysed Fumonisin B1	**HFB1 / Hydrolysed Fumonisin B1**	406.3525	9.08	Find by Molecular Feature	405.3452

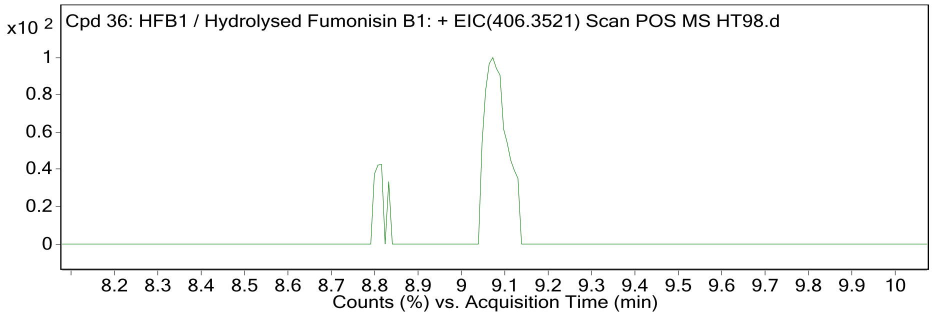

MFE MS Zoomed Spectrum

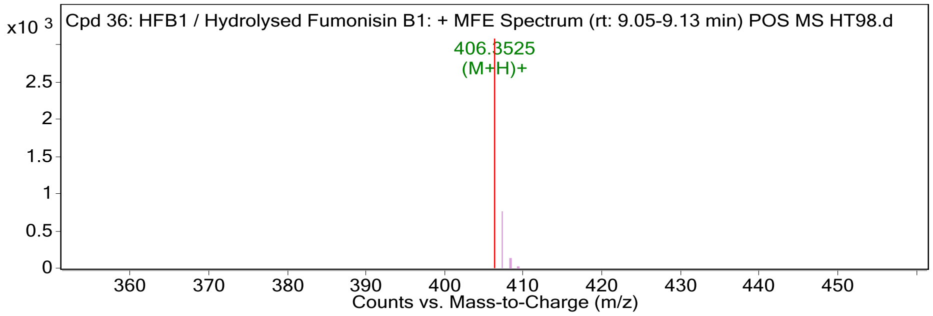

Compound Label	Name	*m/z*	RT	Algorithm	Mass
Cpd 37: Deepoxy deoxynivalenol	**Deepoxy deoxynivalenol**	281.138	9.1	Find by Molecular Feature	280.1307

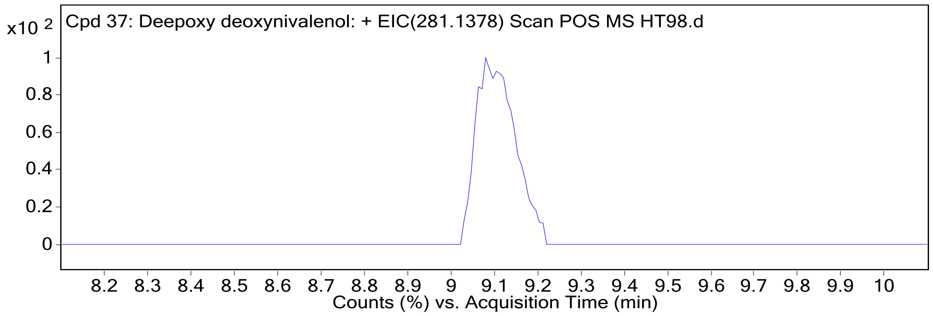

MFE MS Zoomed Spectrum

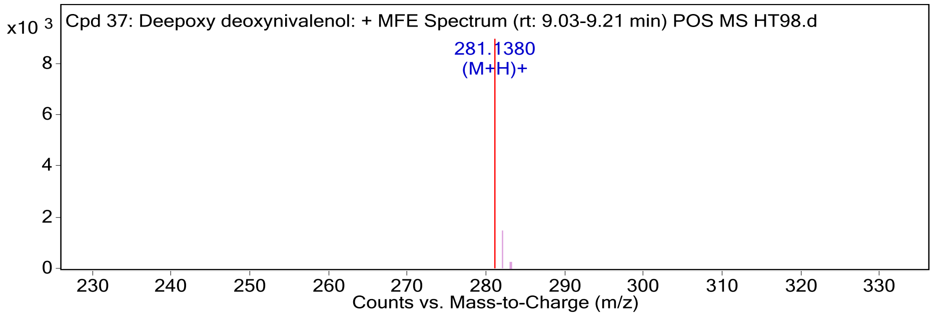

Compound Label	Name	*m/z*	RT	Algorithm	Mass
Cpd 38: HFB3 / Hydrolysed Fumonisin B3	**HFB3 / Hydrolysed Fumonisin B3**	390.3578	9.63	Find by Molecular Feature	389.3506

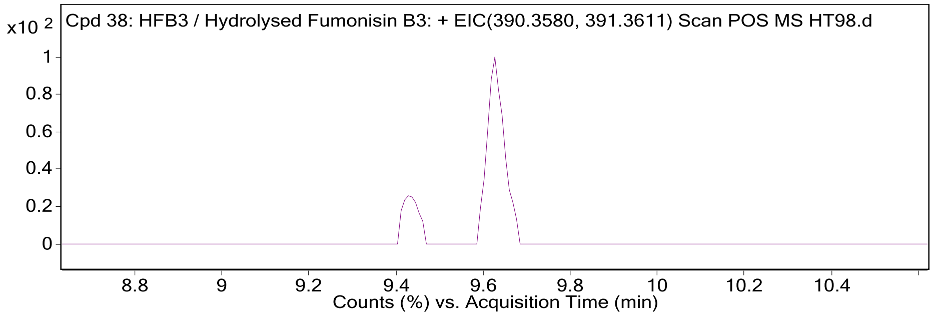

MFE MS Zoomed Spectrum

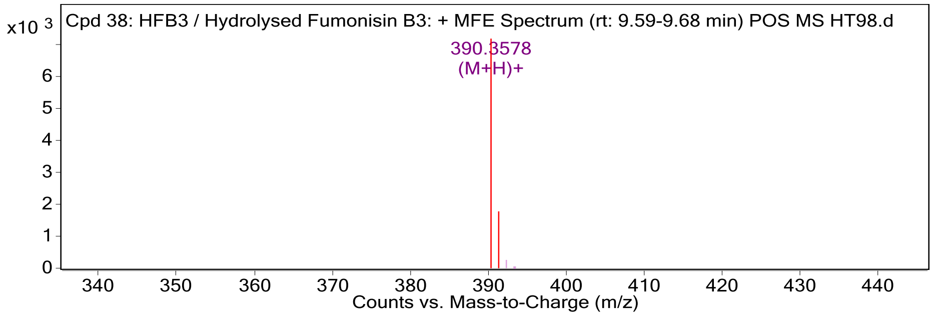

Compound Label	Name	*m/z*	RT	Algorithm	Mass
Cpd 39: Siccanol	**Siccanol**	441.2383	9.73	Find by Molecular Feature	402.2752

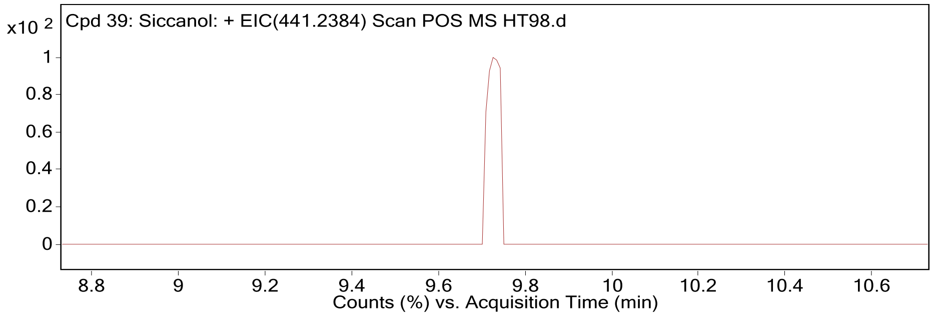

MFE MS Zoomed Spectrum

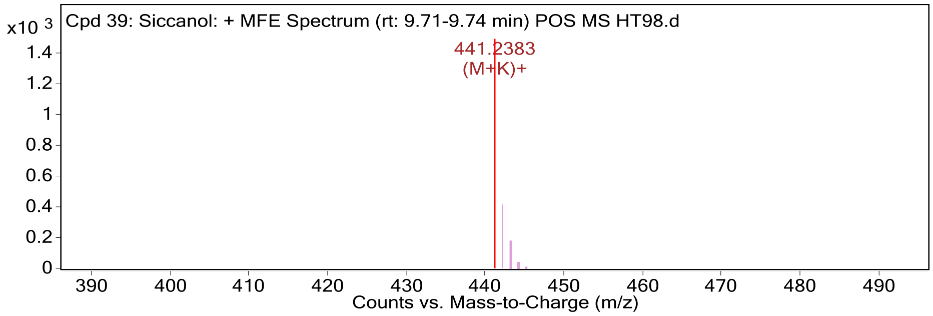

Compound Label	Name	*m/z*	RT	Algorithm	Mass
Cpd 40: 2-Amino-14,16-dimethyloctadecan-3-ol	**2-Amino-14,16-dimethyloctadecan-3-**	314.3416	9.83	Find by Molecular Feature	313.3343

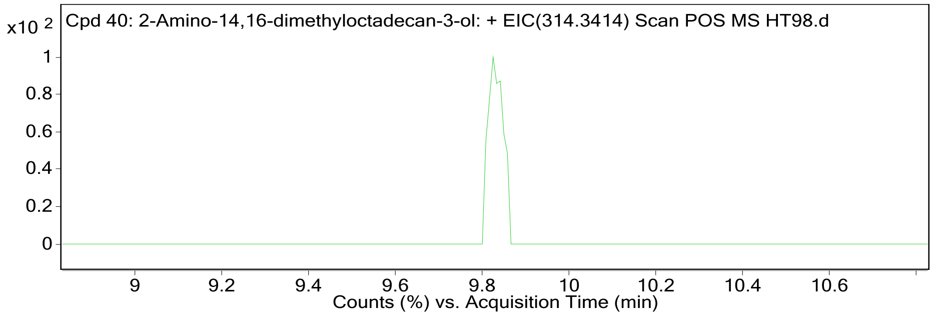

MFE MS Zoomed Spectrum

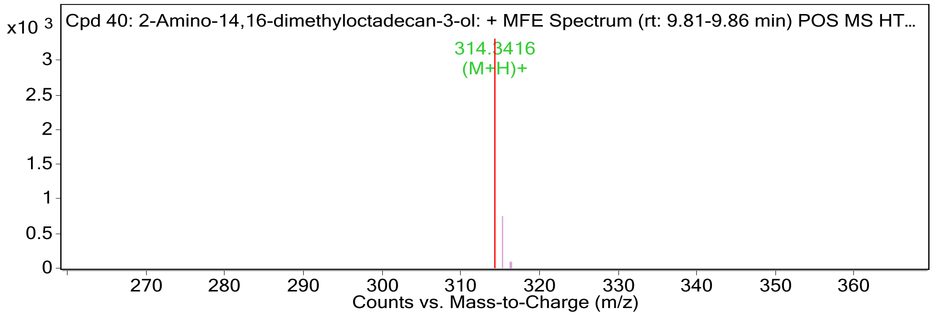

Compound Label	Name	*m/z*	RT	Algorithm	Mass
Cpd 41: Paspaline	**Paspaline**	439.332	9.97	Find by Molecular Feature	421.2982

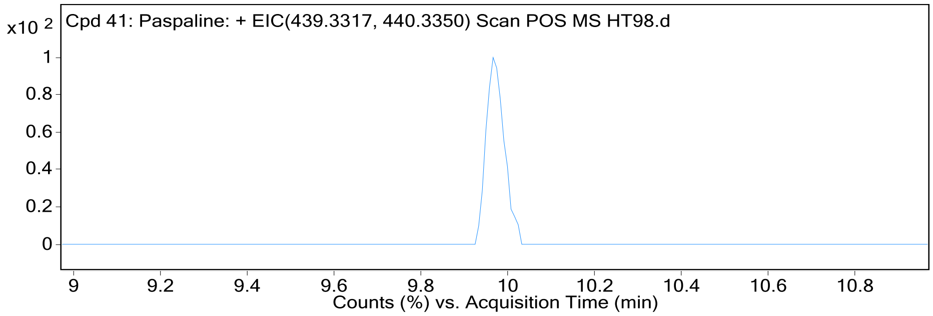

MFE MS Zoomed Spectrum

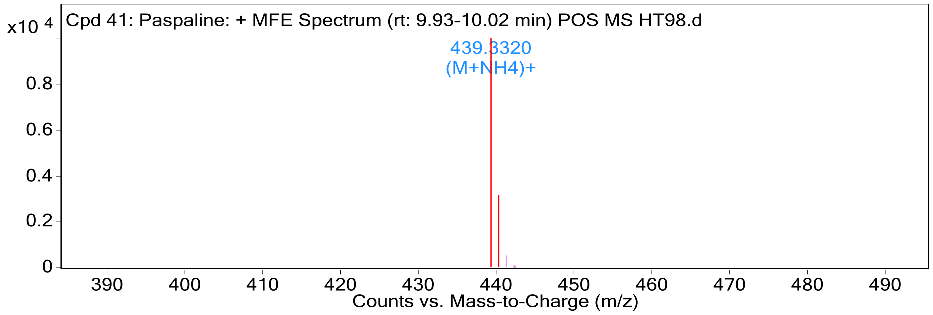

Compound Label	Name	*m/z*	RT	Algorithm	Mass
Cpd 42: Enniatin A1	**Enniatin A1**	685.4755	10.82	Find by Molecular Feature	667.4417

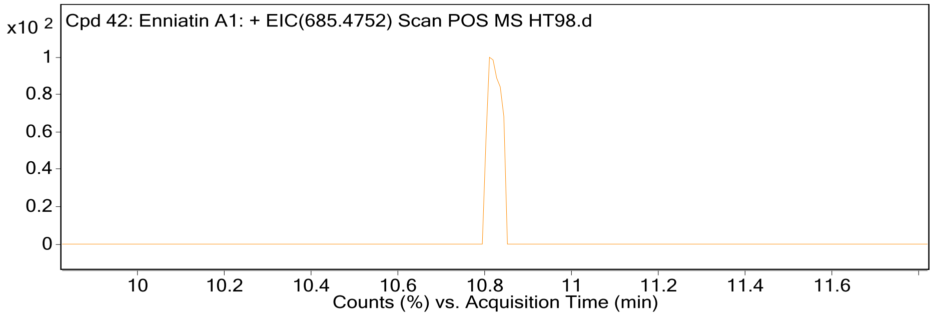

MFE MS Zoomed Spectrum

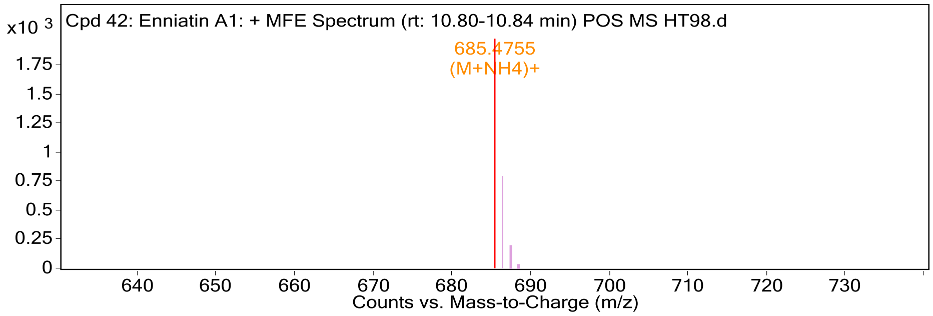

Compound Label	Name	*m/z*	RT	Algorithm	Mass

Cpd 43: Enniatin A2	**Enniatin A2**	1380.9466	10.99	Find by Molecular Feature	681.4562

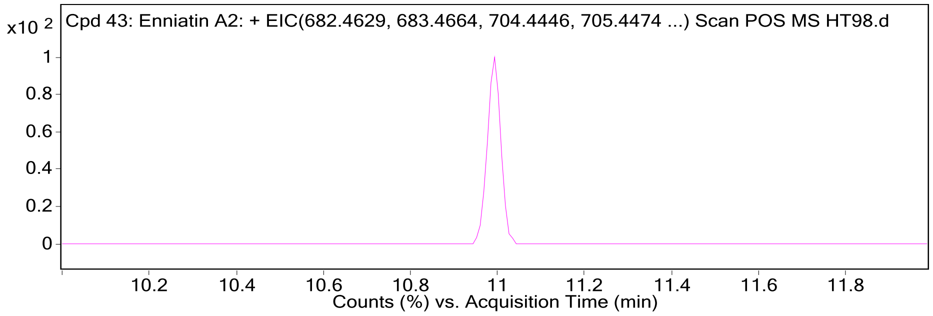

MFE MS Zoomed Spectrum

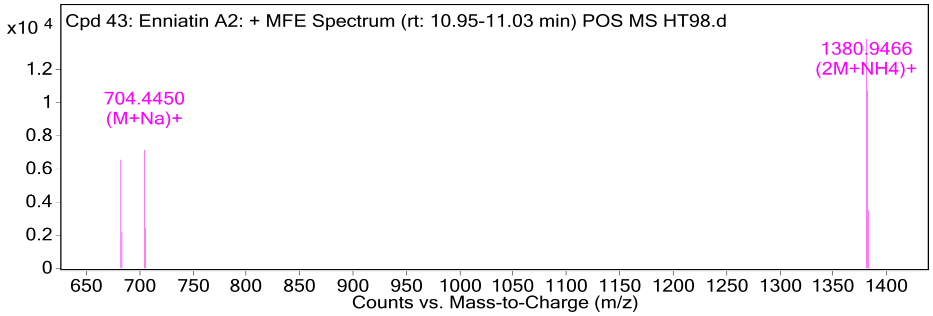

Compound Label	Name	*m/z*	RT	Algorithm	Mass
Cpd 44: Enniatin A2	**Enniatin A2**	699.4876	10.99	Find by Molecular Feature	681.4548

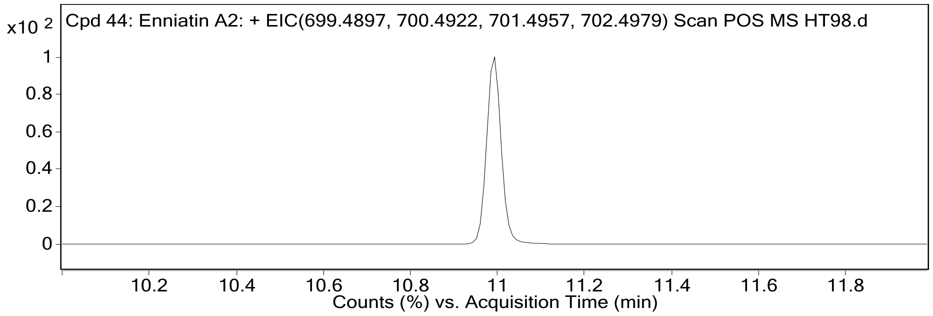

MFE MS Zoomed Spectrum

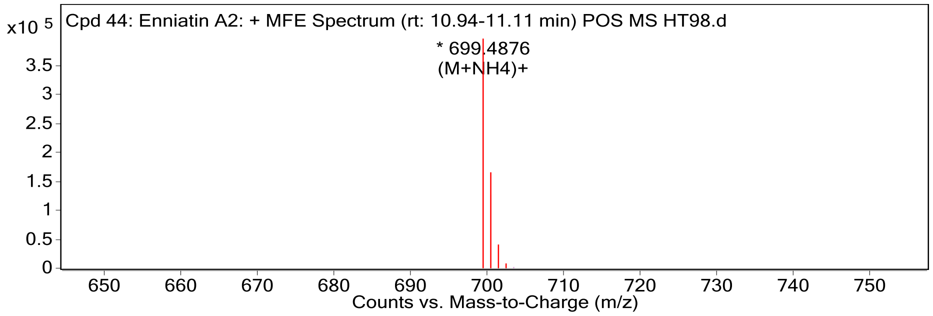

Qualitative Compound Report

Compound Label	Name	*m/z*	RT	Algorithm	Mass
Cpd 45: Beauvericin	**Beauvericin**	801.445	11.04	Find by Molecular Feature	783.4109

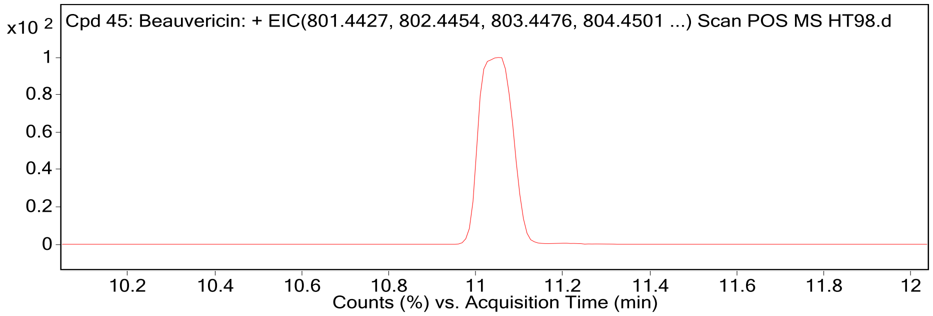

MFE MS Zoomed Spectrum

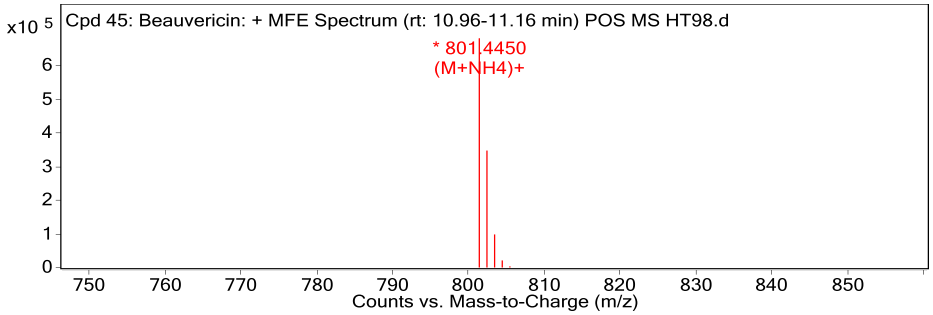

Compound Label	Name	*m/z*	RT	Algorithm	Mass
Cpd 46: Beauvericin	**Beauvericin**	801.4398	11.21	Find by Molecular Feature	783.4059

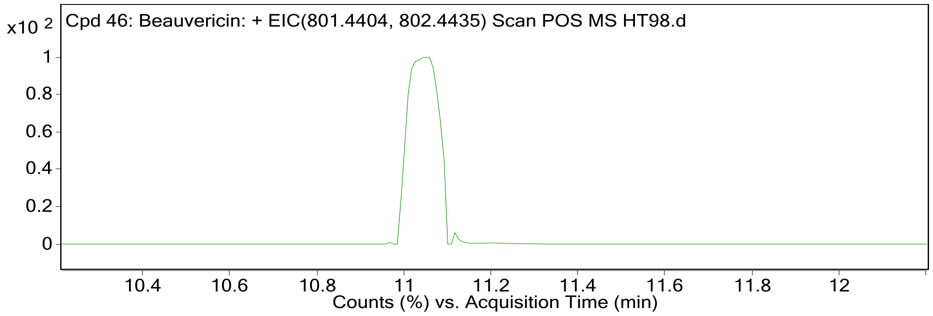

MFE MS Zoomed Spectrum

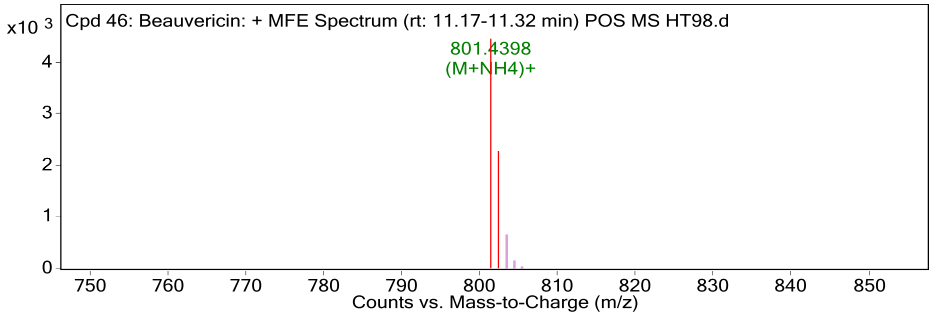

Qualitative Compound Report

Compound Label	Name	*m/z*	RT	Algorithm	Mass
Cpd 47: Sirolimus (Rapamycin)	**Sirolimus (Rapamycin)**	931.5915	11.56	Find by Molecular Feature	913.5576

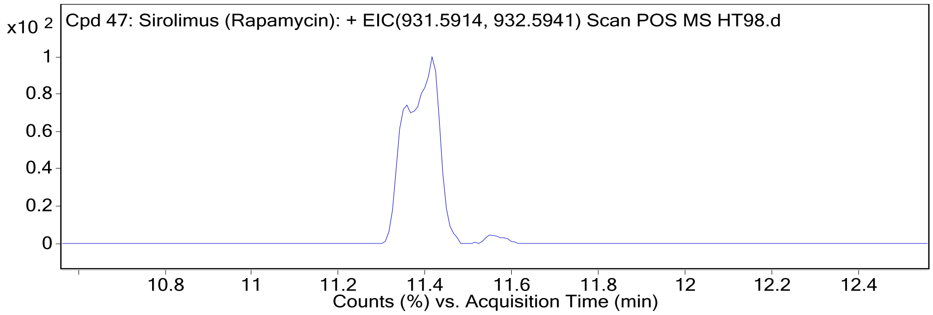

MFE MS Zoomed Spectrum

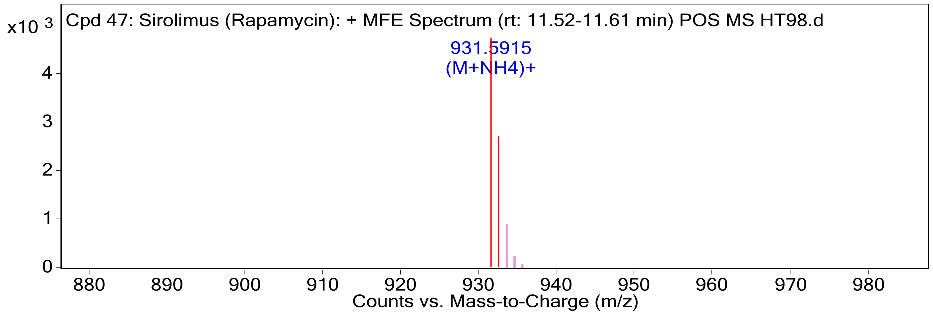

--- End Of Report ---
